# A General Hydrotrifluoromethylation of Unactivated Olefins Enabled by Voltage‐Gated Electrosynthesis

**DOI:** 10.1002/anie.202415218

**Published:** 2024-11-11

**Authors:** Eva M. Alvarez, Jinxiao Li, Christian A. Malapit

**Affiliations:** ^1^ Department of Chemistry Northwestern University 2145N Sheridan Road Evanston IL 60208 USA

**Keywords:** carbanion, electrosynthesis, hydrotrifluoromethylation, olefin functionalization

## Abstract

Here we present the first successful hydrotrifluoromethylation of unactivated olefins under electrochemical conditions. Commercially available trifluoromethyl thianthrenium salt (TT^+^−CF_3_BF_4_^−^, E_p/2_=−0.85 V vs Fc/Fc^+^) undergoes electrochemical reduction to generate CF_3_ radicals which add to olefins with exclusive chemoselectivity. The resulting carbon centered radical undergoes a second cathodic reduction, instead of a classical HAT process, to generate a carbanion that can be terminated by protonation from solvent. The use of MgBr_2_ (+0.20 V onset oxidation potential) plays a key role as an enabling sacrificial reductant for the reaction to operate in an undivided cell. Guided by cyclic voltammetry (CV) studies, fine‐tuning the solvent system, trifluoromethylating reagent's counteranion and careful selection of redox processes, this work led to the development of a voltage‐gated electrosynthesis by pairing two redox processes with a narrow potential difference (ΔE≈1.00 V) allowing the reaction to proceed with two important advances: (a) high reactivity and selectivity towards hydrotrifluoromethylation over undesired dibromination, and (b) an unprecedented functional group tolerance, including aniline, phenols, unprotected alcohol, epoxide, trialkyl amine, and several redox sensitive heterocycles.

## Graphical abstract

This work presents the discovery and development of a regioselective hydrotrifluoromethylation of olefins under electrochemical conditions. Guided by mechanistic studies, a voltage‐gated electrolysis was designed by integrating two redox processes operating within a narrow potential window. This strategy facilitates olefin hydrotrifluoromethylation (over dibromination) that showcases broad functional group tolerance.

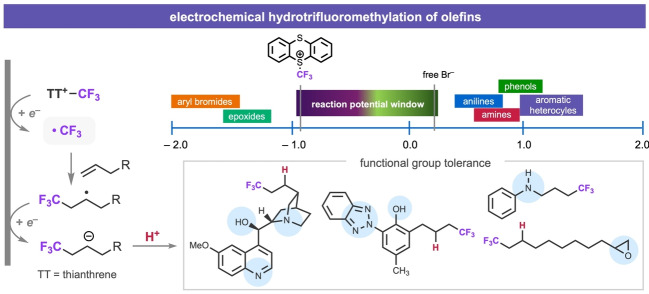

Given the broad interest in the incorporation of trifluoromethyl substituents into organic molecules to improve the pharmacokinetic profile of drug candidates (Figure 1a),[^1^, ^2^] it can be expected that interest in hydrotrifluoromethylation of unactivated alkenes will increase. Significant advances in hydrotrifluoromethylation rely on transition‐metal‐free,^[3]^ transition metal catalysis platforms,^[4]^ and photoredox catalysis using Umemoto or Langlois’ reagent as shown by the pioneering work of Gouverneur^[5]^ and Nicewicz,^[6]^ respectively. Recently, West reported a photoredox strategy that has demonstrated the synthetic advantages of merging earth‐abundant metal catalysis with trifluoroacetic acid (TFA) as a CF_3_ radical source.^[7]^ Despite these advancements, the development of a transition metal‐free strategy with high functional group tolerance remains unaddressed.

The resurgence of electrochemistry for organic synthesis has shown to be a powerful tool to discover and develop selective organic transformations,[^8^, ^9^] being the hydrotrifluoromethylation of alkenes unexplored.[^10^, ^11^, ^12^] Electrochemical anodic oxidation[^13^, ^14^, ^15^] or radical electroreductive^[16]^ strategies are a few examples that deal with introducing fluoroalkyl groups to olefins, however, these methods result in heterodifunctionalization of olefins[^17^, ^18^] (e.g. halotrifluoromethylation, hydroxytrifluoromethylation). The first electrochemical hydrotrifluoromethylation of olefins was observed in Langlois’ work (Figure 1b)^[14]^ where anodic oxidation of CF_3_SO_2_K (*E*_ox_=1.05 V *vs* SCE) generated CF_3_ radicals that added to olefins, yielding three inseparable trifluoromethylated products with hydrotrifluoromethylation as a minor byproduct (8–20 % yield, 3 examples).^[14]^ Alternatively, the use of trifluoroacetic acid (TFA, E_1/2_^ox^>2.4 V *vs* SCE) in an electrophotochemical strategy in presence of thiol as hydrogen‐atom‐transfer (HAT) source was reported in low yields (15 %, one example).^[19]^ Given these limitations and the constrained progress in cathodic reduction‐enabled transformations for olefin functionalization, our group explored electroreductive strategies as shown in our recent work in arene C−H amination^[20]^ through cathodic generation of reactive electrophilic amine radical dicationic intermediates. As such, we envisioned that an electroreductive strategy to generate electrophilic CF_3_ radicals could address synthetic limitations in hydrotrifluoromethylation of olefins (Figure 1c).

Most hydrofunctionalization reactions of alkenes are driven by an HAT process and often require strongly reducing organic photoredox catalyst,^[5]^ the use of stoichiometric amounts of thiol[^3^, ^16^] or the dimeric disulfide generation in the thiol catalytic turnover,^[6]^ which may result in an inefficient process with low functional group tolerance. Of interest is whether electrochemistry could allow the selective generation of carbanion intermediates from unactivated olefins after radical addition enabling protonation as the terminal step rather than the adoption of an HAT process (Figure 1d). The feasibility of a cathodic reduction of carbon radical to carbanion was recently utilized in the difunctionalization of alkenes by Lin and required stabilizing groups such as Ph^[21]^ or Bpin.^[22]^

**Figure 1:**
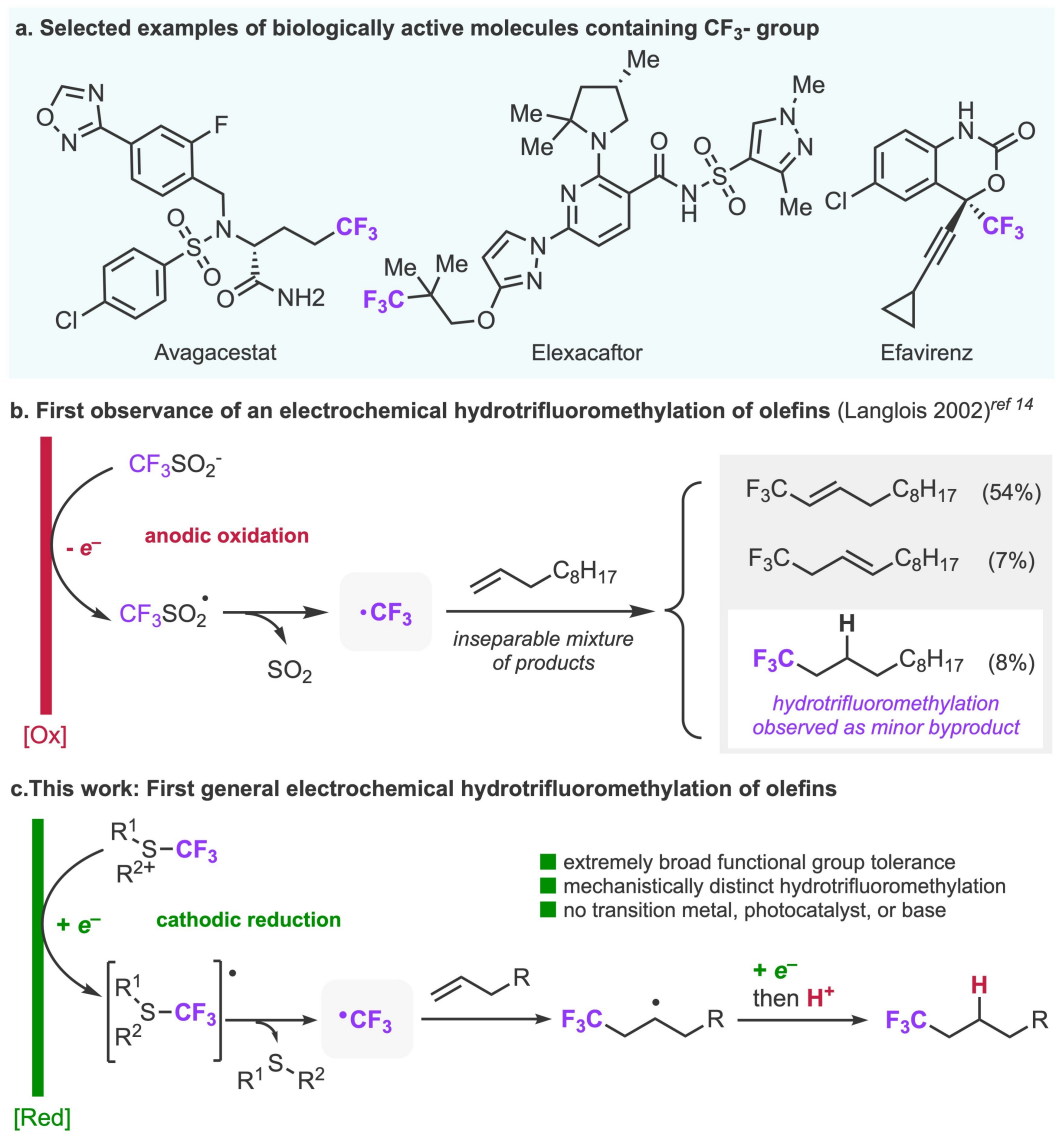
(a) Selected examples of trifluoromethyl‐containing pharmaceuticals. Electrochemical hydrotrifluoromethylation of olefins through (b) anodic oxidation using Langlois′ reagent, and (c) via cathodic reduction of CF_3_‐sulfonium salt, this work.

To design and develop a highly selective and functional group tolerant hydrotrifluoromethylation of olefins, we chose electron‐rich arene 4‐allylanisole as model substrate, which has two irreversible oxidation peaks at +1.55 and +2.10 V vs SCE.^[23]^ Next, the choice of a trifluoromethylating reagent with a low negative reduction potential and a sacrificial reductant that readily undergoes oxidation at the anode are desired to avoid undesirable electroactivity of sensitive functional groups. As such, we selected TT^+^CF_3_BF_4_^−^, among other sulfonium salts, and explored various sacrificial reductants (Figure 2a). Our initial attempts for electroreductive hydrotrifluoromethylation of 4‐allylanisole with TT^+^‐CF_3_BF_4_^−^ **1** **a** (Figure 2c, conditions A) proved to be challenging due to several unproductive synthetic routes that needed to be addressed as outlined in Figure 2a. First, C−H trifluoromethylation of electron‐rich (hetero) arenes could take place preferentially over addition to the olefin,[^24^, ^25^] yet CF_3_ radical addition to aromatic systems is a slow process compared to nucleophilic unactivated olefins based on rate constant values.^[26]^ Second, the CF_3_ radical can undergo H‐atom abstraction to produce fluoroform (HCF_3_)^[27]^ or anionic deprotonation (2 e^−^ process). Third, thianthrene (TT) is known to form stable radical cations (TT^.+^) by both chemical oxidants^[28]^ and electrochemical methods^[29]^ and will potentially undergo thianthrenation of electron rich arenes,^[28]^ olefins^[30]^ or react with reactive functional groups (e.g. alcohols, phenols, amines, etc.).[^31^, ^32^] In our initial attempt (Figure 2c, conditions A) we hoped that the generated thianthrene could act as the sacrificial reductant,^[33]^ however, only traces of product were observed. Nonetheless, we found evidence of thianthrene‐*S*‐oxide (TTO) in our system, suggesting consumption of the TT^+^‐CF_3_ reagent and overoxidation of generated thianthrene towards TTO. Thus, we recognized the need of a sacrificial reductant (X^n^) with a low oxidation potential, which could get oxidized before thianthrene to avoid oxidation of TT into reactive intermediates (Figure 2b).

**Figure 2:**
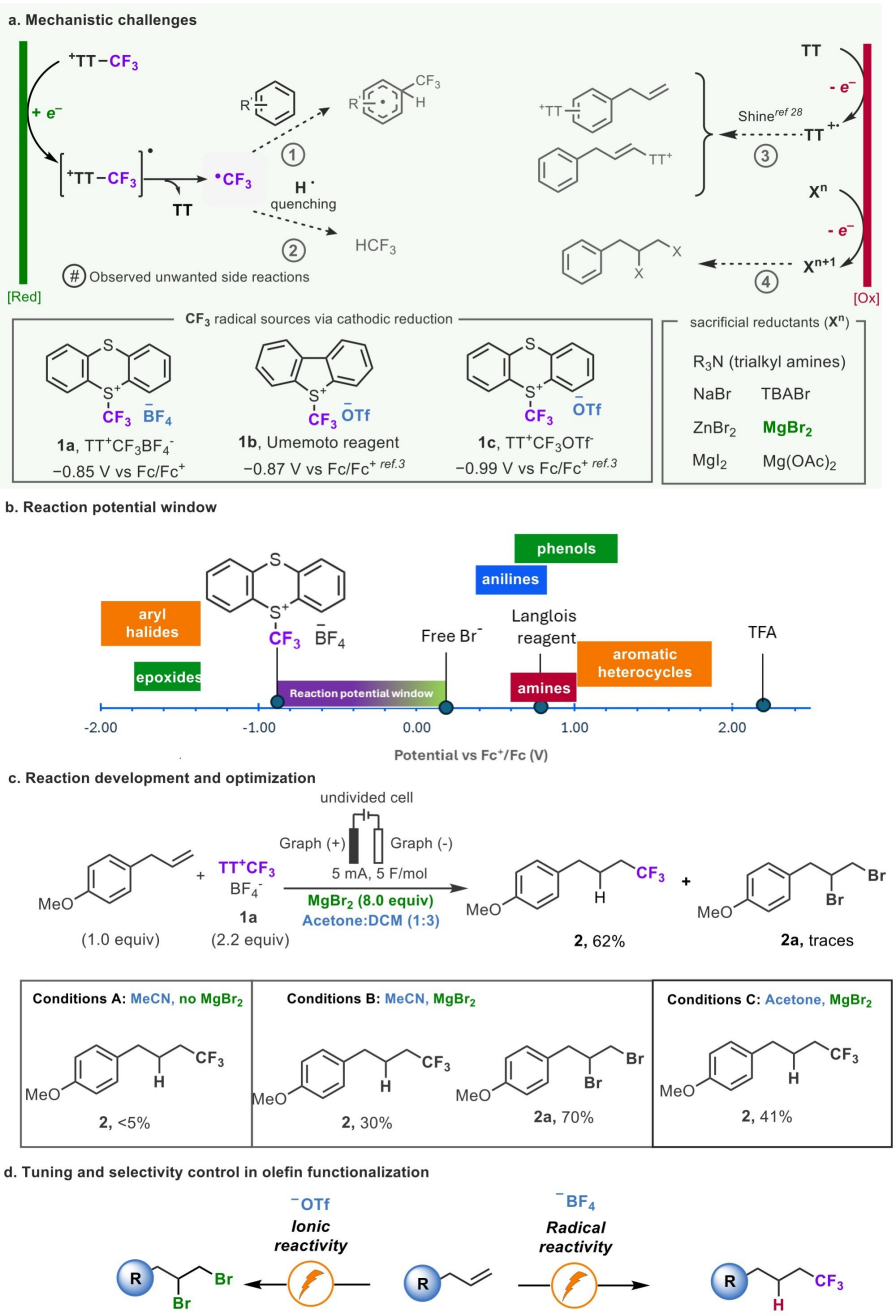
Rational development of electroreductive hydrotrifluoromethylation of olefins: (a) challenges and rational choice of reagents, (b) voltage‐gated electrolysis, (c) reaction development, and (d) tuning reactivity towards hydrotrifluoromethylation over dibromination.

On the basis of reported electrochemical reduction of sulfonium salts,^[34]^ we explored organic amines (DIPEA and Et_3_N) and low oxidation potential bromide salts^[35]^ as sacrificial reductants. While DIPEA showed traces of product due to the incompatibility with our trifluoromethyl thianthrenium salt and reaction conditions, MgBr_2_ gave 30 % yield of hydrotrifluoromethylated product **2**, however, the dibromination of the olefin **2 a** was observed as the major product (Figure 2c, conditions B). The formation of **2 a** results from the reaction of olefin with the oxidized bromide.[^36^, ^37^, ^38^] Due to MgBr_2_’s limited solubility in MeCN, we found that the use of acetone as solvent exclusively gave the desired product **2** and the optimal yield can be obtained when acetone/DCM binary solvent is used. Under the optimized condition, the desired product **2**, unreacted olefin, and trace amount of **2 a** were obtained (Figure 2c, conditions B). Pleasingly, the TT^+^−CF_3_BF_4_^−^ gets completely consumed into thianthrene without any evidence of TTO in the reaction mixture. The use of 2.2 equivalents of TT^+^‐CF_3_BF_4_^−^ is necessary to achieve higher yields of hydrotrifluoromethylation. Screening of other bromide sources (TBABr, NaBr, and ZnBr_2_) and Mg salts as sacrificial reductants led to no desired product. Other screened counter electrodes (RVC, Pt) showed a drastic decrease in the yield, and the use of Mg as sacrificial metal anode resulted in no product formation. Alternative trifluoromethylating chemicals such as Umemoto reagent, gave lower yield and selectivity over dibromination (see the Supporting Information).

Interestingly, replacing TT^+^CF_3_BF_4_^−^ by TT^+^CF_3_OTf^−^ in our optimal reaction conditions, the functionalization of olefins is diverted from a radical mechanism to an ionic dibromination reactivity (Figure 2d). We attributed the differences in the reactivity and selectivity to increased electrode resistance, which leads to an undesired anodic process to compensate the high electrode potential.^[22]^ Despite the high dielectric constant of our binary solvent, MgBr_2_ shows poor solubility and low solution conductivity. Through qualitative analysis surrounding the role of the trifluoromethylating reagent's counteranion in the solubility of our system, we found that MgBr_2_ salt (0.2 M) in presence of TT^+^CF_3_BF_4_^−^ (54 mM) in DCM/Acetone showed a notable increase in solubility, which can likely be attributed to the higher association strength of BF_4_^–^ with Mg^2+^ (ion‐pair formation) relative to OTf^−^,[^39^, ^40^] resulting in an electrochemically active mixture. In contrast, MgBr_2_ (0.2 M) salt in presence of TT^+^CF_3_OTf^−^ (54 mM) in DCM/Acetone produced a cloudy light‐orange solution (See Supporting Information). Previous studies have indicated the crucial role of ion‐pairing^[41]^ on the reaction mechanism and rate of electron transfer, which could explain the reason for this behavior. In line with these observations, a constant current electrolysis experiment was set up to monitor the potential of the working and counter electrodes during the reaction using TT^+^CF_3_OTf^−^. As expected, the potential of the working electrode rapidly rises as a consequence of poor solubility of the system. Once TBABF_4_ (5.0 equiv.) as supporting electrolyte is added, the electrode resistance decreases significantly as the solubility of MgBr_2_ increases and the hydrotrifluoromethylation product is observed. Under similar conditions, a uniform potential distribution at the working electrode was observed using TTCF_3_BF_4_ instead (See Supporting Information).

This carefully designed reaction led to an electrochemical hydrotrifluoromethylation of olefins that displays a high functional group tolerance under milder reaction conditions (Figure 3). For example, our system is compatible with electron‐rich and electron‐deficient allylbenzene derivatives (**2**, **3**, **4**, **12**, and **13**) contrasting the scope limitation in previous protocols, where allylic C−H functionalization of simple olefins is favored.^[42]^ Aliphatic phenethyl‐substituted substrates (**5**, **6** and **7**), and cyclic internal olefin (**15**) are obtained in moderate yields. Unlike conventional strategies,[^3^, ^6^, ^7^] we were able to expand the scope to OH‐containing groups such as phenols (**12** and **13**) and secondary alcohol (**19**) in absence of inorganic/organic bases. Remarkably, epoxide (**8**) is tolerated despite being functionalized through electroreductive approaches.^[43]^ Easily reducible nitrile group (**18**) survived the electroreductive conditions. Redox labile functionalities such as aryl bromide (**4**), aniline (**7**), phthalimide (**9**), amide (**11**), esters (**14**, **22**), aryl chloride (**20**), sulfonamide (**23**) and indole (**22**) are well tolerated. We also explored styrene derivatives (**16**, **17**), which resulted in compatible substrates with our system. We showcased our methodology's application in the functionalization of pharmaceutically active compounds (**18**–**23**) towards the formation of desired hydrotrifluoromethylated products without the need of protecting the hydroxyl group in quinine (**19**), and no observed undesired trifluoromethylation on electron rich arenes such as theobromine (**21**) or indomethacin (**22**) analogues.

**Figure 3:**
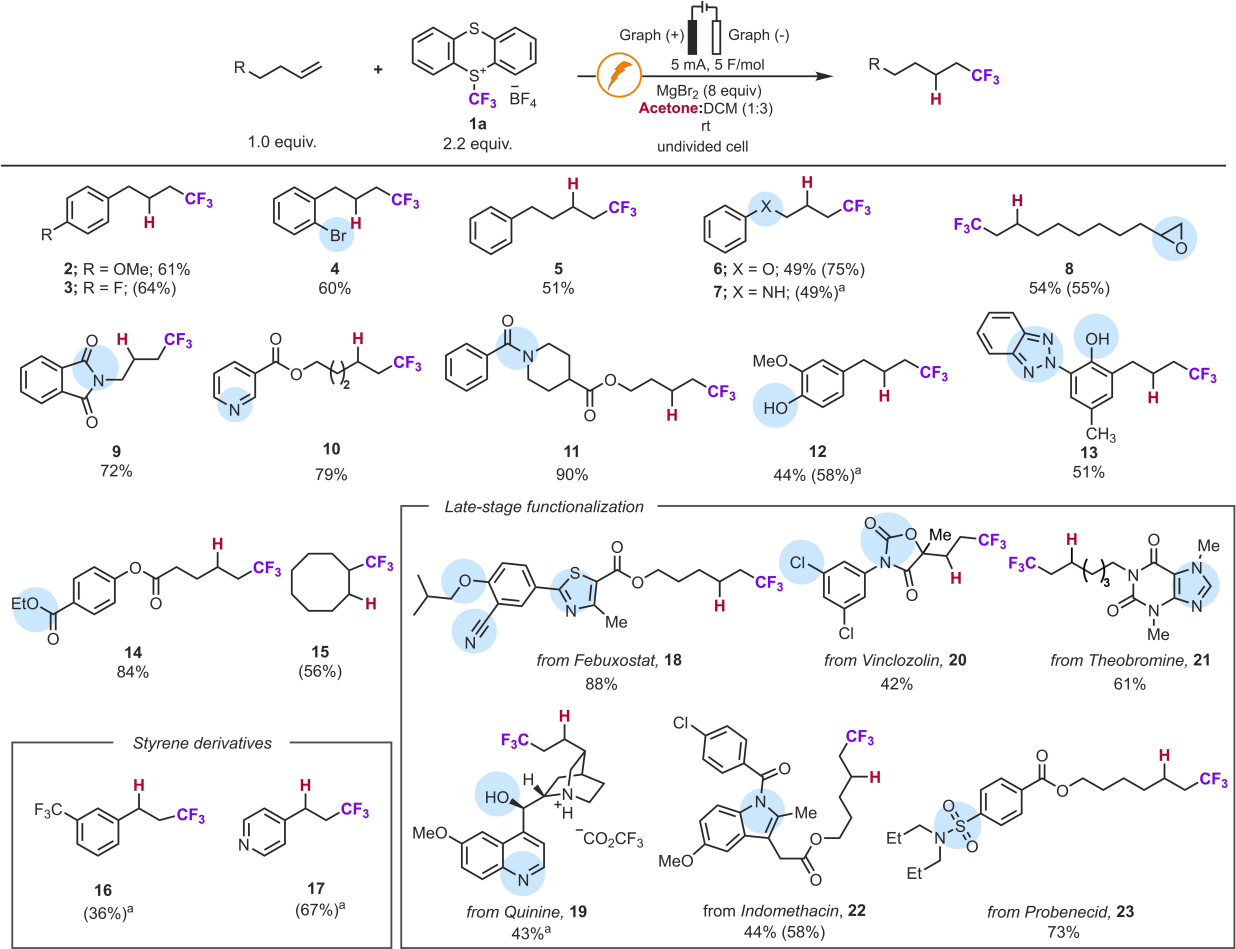
Scope of the electrochemical hydrotrifluoromethylation of unactivated olefins. Yields correspond to isolated, purified products. Due to the volatility of a few products, yields on parenthesis are reported on the basis of ^19^F NMR and ^1^H NMR analysis using PhCF_3_ and mesitylene as internal standards, respectively. The general reaction conditions are as follows: olefin (0.25 mmol, 1.0 equiv.), TT^+^CF_3_BF_4_^−^ (2.0 equiv.–2.2 equiv.), MgBr_2_ (8.0 equiv.), acetone:DCM (1 : 3), rt. Electrolysis parameters: constant current of 5 mA, 5 F/mol, and stirring at 1500 rpm. [a] 10.0 equiv. of TFA was added.

To shed light into the mechanism of the reaction, several experimental mechanistic studies were performed (Figure 4a–d) and the proposed mechanism is summarized in Figure 4e. CV studies (Figure 4a–1) revealed that TT^+^CF_3_BF_4_^−^ (E_p/2_=−0.85 V vs Fc/Fc^+^) undergoes electrochemical reduction,^[33]^ while acetone is inert to cathodic reduction. The oxidative CV profile (Figure 4a–2) showed that MgBr_2_ as sacrificial reductant undergoes productive oxidation on the counter electrode with an onset potential of +0.20 V vs Fc/Fc^+^. Together with TT^+^CF_3_BF_4_^−^, this results in a very narrow net redox operating window of ~1.00 V. As such, functional groups that are susceptible to reduction or oxidation are tolerated. A radical clock experiment (Figure 4b) on diethyl diallylmalonate showed the formation of the cyclized product **24** as a mixture of *cis* and *trans* diastereomers (dr=10 : 1), which is in agreement with CF_3_ radical addition to olefins, followed by a 5 exo‐trig cyclization[^3^, ^5^] (Figure 4b). Consistent with the preceding result, the CF_3_ radical undergoes addition to 4‐allylanisole to form intermediate **A**.

**Figure 4:**
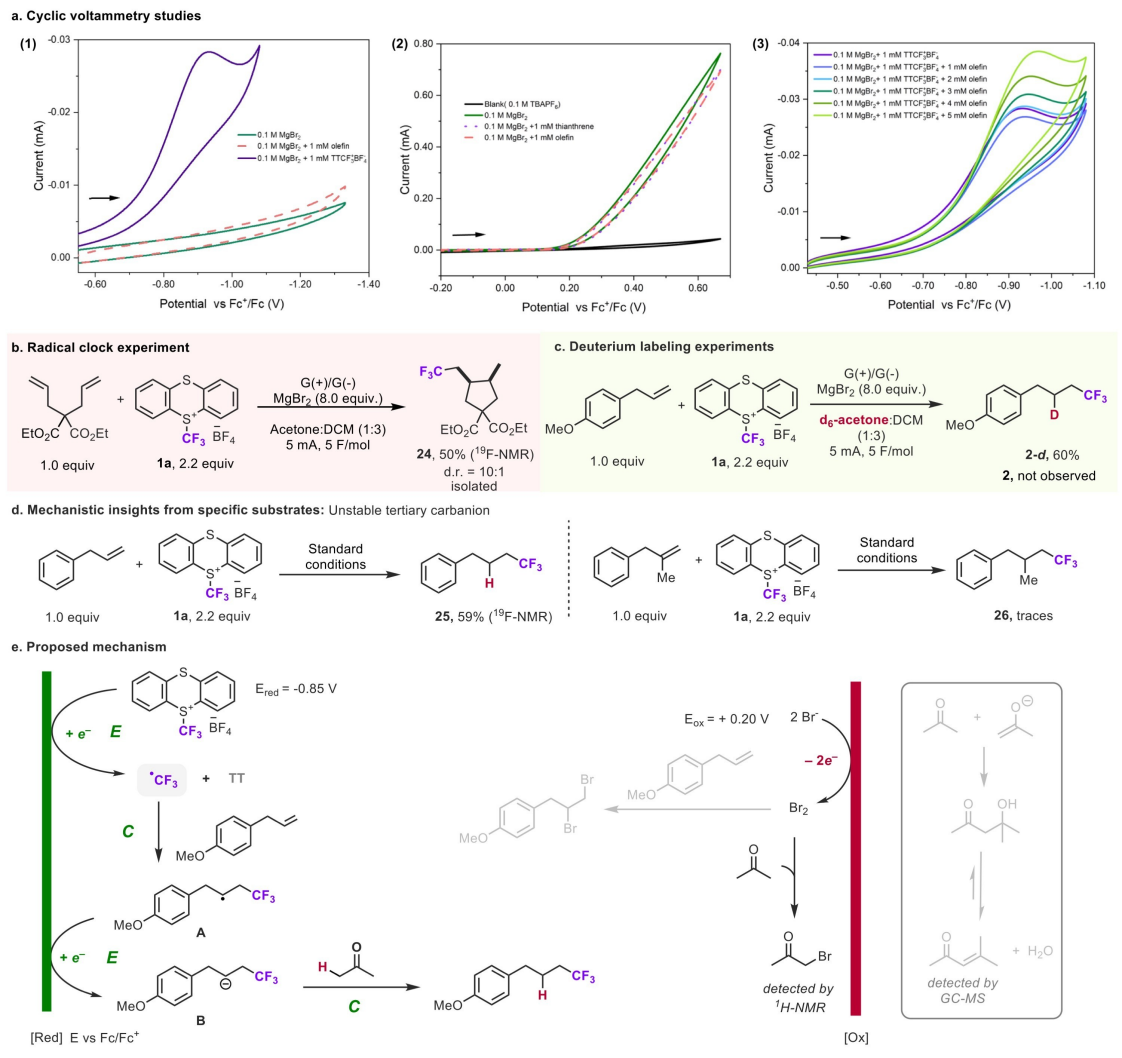
(a) Cyclic voltammetry studies. The black arrows indicate the initial scan direction. (b) Radical clock experiment. (c) Deuterium labeling experiment. (d) Control experiments. (e) Proposed mechanism. See the Supporting Information for more details on experimental mechanistic studies.

To understand the fate of intermediate **A**, deuterium labeling studies (Figure 4c) using acetone‐d_6_ as solvent revealed the exclusive formation of deuteriutrifluoromethylation product (**2**–**d**) denoting that the α‐protons of acetone serve as proton source. Furthermore, CV studies (Figure 4a–3) were performed to a solution of TT^+^CF_3_BF_4_^−^ with increasing amount of olefin concentration. The noticeable increase in the current density and cathodic shift suggests a chemical step and a second reduction.^[44]^ Considering the proton donor is part of the solvent system (acetone), only a single but enhanced first cathodic peak is observed as the production of carbanion is blocked by protonation from acetone.^[44]^ This indicates intermediate **A** undergoes reduction to carbanion **B** at voltages more cathodic than −0.85 V.[^44^, ^45^, ^46^] Additionally, we performed CV and constant potential bulk electrolysis experiment of 25 mM 4‐allylanisole with 55 mM TT^+^CF_3_BF_4_^−^ in a solution of 0.2 M MgBr_2_ in DCM/Acetone to identify a reductive peak potential (E_p_) at −0.90 V vs Fc/Fc^+^ for the reduction of TT^+^CF_3_BF_4_^−^. To perform the constant potential (CP) experiment, the potential of the working electrode (graphite) was set at −1.20 V vs Fc/Fc^+^. Initially, a high current density is observed, which gradually decays over the consumption of **1 a** where the hydrotrifluoromethylation product was obtained in 54 % (^19^F NMR yield) with 18 % (^1^H NMR yield) 4‐allylanisole remaining after 2 h of applied potential at the working electrode (See Supporting Information). Furthermore, the addition of 1.0 equiv. of TEMPO as radical scavenger under standard conditions did not suppress the reactivity, which is in line with a second electron reduction mechanism being favorable over an intermolecular radical electroreductive process. Further evidence to support a carbanion intermediate resulting from the second cathodic reduction is obtained from the GC‐MS analysis of our reaction mixture, which identifies the homoaldol condensation product of acetone (see the Supporting Information). Moreover, 1,1‐disubstituted olefins were not amenable to our methodology presumably due to the challenges associated with formation of an unstable tertiary carbanion (Figure 4d).

Collectively, these data suggest an electrochemical reduction of **1 a** to thianthrenyl radical species facilitated by ion‐pairing interaction between Mg^2+^ and BF_4_^−^ that will eventually decompose into thianthrene and electrophilic CF_3_ radical, which undergoes radical addition to the unactivated olefin in a polarity‐matched process to form intermediate **A**. This is followed by further reduction of the radical intermediate **A** to the corresponding unstabilized carbanion **B**. Subsequently, the reaction with acetone as a sacrificial proton source^[46]^ delivers the desired hydrotrifluoromethylated product, unlike photoredox synthetic approaches where the resulting radical intermediate abstracts a hydrogen atom from solvent^[5]^ or thiol.[^6^, ^7^] Moreover, we propose that magnesium bromide undergoes productive anodic oxidation into Br_2_/Br_3_^−[47]^ which makes possible a broader substrate scope and high functional group tolerance by preventing the oxidation of sensitive functional groups.

In summary, we report a selective and mechanistically distinct hydrotrifluoromethylation of unactivated olefins over undesired dibromination by combining two reduction events. Guided by mechanistic studies and careful choice of redox‐active components, a voltage‐gated strategy was implemented to make this reaction proceed with high functional group tolerance allowing hydrotrifluoromethylation of olefins bearing anilines, phenols, alcohols, epoxides, and several redox‐sensitive heterocycles. Taken together, the results reported here provide a rationale to favor a radical pathway over dominant competitive ionic process (dibromination) that can be implemented in other selective olefin functionalization reactions.

## Conflict of Interests

The authors declare no conflict of interest.

## Supporting information

As a service to our authors and readers, this journal provides supporting information supplied by the authors. Such materials are peer reviewed and may be re‐organized for online delivery, but are not copy‐edited or typeset. Technical support issues arising from supporting information (other than missing files) should be addressed to the authors.

Supporting Information

## Data Availability

The data that support the findings of this study are available in the supplementary material of this article.
