# Supplementary material for: A General Hydrotrifluoromethylation of Unactivated Olefins Enabled by Voltage‐Gated Electrosynthesis
Source: Angew Chem Int Ed Engl. 2024 Nov 11;64(4):e202415218. doi: 10.1002/anie.202415218 (PMC11753607; doi:10.1002/anie.202415218)
Supplement: Supplementary file 1 — Supporting Information [file ANIE-64-e202415218-s001.pdf]

Angewandte Chemie
Eine Zeitschrift der Gesellschaft Deutscher Chemiker

Supporting Information

A General Hydrotrifluoromethylation of Unactivated Olefins Enabled by Voltage-Gated Electrosynthesis

E. M. Alvarez, J. Li, C. A. Malapit**

Supporting Information

A general hydrotrifluoromethylation of unactivated olefins enabled by voltage-gated electrosynthesis

Eva Maria Alvarez[*], Jinxiao Li, Christian A. Malapit*

Department of Chemistry, Northwestern University, Technological Institute, Evanston, Illinois 60208, United States

*Correspondence to: christian.malapit@northwestern.edu

Table of Contents

MATERIALS AND METHODS 6

EXPERIMENTAL DATA 7

Preparation of starting materials 7

Synthesis of olefins 7

Pent-4-en-1-yl nicotinate (S1) 7

General procedure for electroreductive hydrotrifluoromethylation of unactivated olefins 8

Alternative procedure for hydrotrifluoromethylation of unactivated olefins 8

1-Methoxy-4-(4,4,4-trifluorobutyl)benzene (2) 9

1-Methoxy-4-(4,4,4-trifluorobutyl)benzene (2) 10

1-Fluoro-4-(4,4,4-trifluorobutyl)benzene (3) 12

1-Bromo-2-(4,4,4-trifluorobutyl)benzene (4) 13

(5,5,5-Trifluoropentyl)benzene (5) 14

(4,4,4-Trifluorobutoxy)benzene (6) 15

N-(4,4,4-Trifluorobutyl)aniline (7) 16

2-(9,9,9-Trifluorononyl)oxirane (8) 17

2-(4,4,4-Trifluorobutyl)isoindoline-1,3-dione (9) 17

Nicotinic ester hydrotrifluoromethylated derivative (10) 18

5,5,5-Trifluoropentyl 1-benzoylpiperidine-4-carboxylate (11) 19

2-Methoxy-4-(4,4,4-trifluorobutyl)phenol (12) 20

2-(2H-Benzo[d][1,2,3]triazol-2-yl)-4-methyl-6-(4,4,4-trifluorobutyl)phenol (13) 21

Ethyl 4-((6,6,6-trifluorohexanoyl)oxy)benzoate (14) 21

(Trifluoromethyl)cyclooctane (15) 22

1-(Trifluoromethyl)-3-(3,3,3-trifluoropropyl)benzene (16) 24

4-(3,3,3-Trifluoropropyl)pyridine (17) 24

Febuxostat hydrotrifluoromethylated derivative (18) 25

Quinine hydrotrifluoromethylated derivative (19) 25

Vinclozolin hydrotrifluoromethylated derivative (20) 26

Theobromine hydrotrifluoromethylated derivative (21) 27

Indomethacin hydrotrifluoromethylated derivative (22) 28

Probenecid hydrotrifluoromethylated derivative (23) 29

Optimization 29

Mechanistic investigations 32

Control experiments 32

Cyclic Voltammogram studies 39
Constant Potential Experiment 42
Voltage Profile Measurement 44
Redox potential substrates (Functional group tolerance) 46

SPECTROSCOPIC DATA 49

^{1}H NMR of nicotinic ester hydrotrifluoromethylated derivative (S1) 49

^{13}C NMR of nicotinic ester hydrotrifluoromethylated derivative (S1) 50

^{1}H NMR of 1-methoxy-4-(4,4,4-trifluorobutyl)benzene (2) 51

^{13}C NMR of 1-methoxy-4-(4,4,4-trifluorobutyl)benzene (2) 52

^{19}F NMR of 1-methoxy-4-(4,4,4-trifluorobutyl)benzene (2) 53

^{1}H NMR of 1-bromo-2-(4,4,4-trifluorobutyl)benzene (4) 54

^{13}C NMR of 1-bromo-2-(4,4,4-trifluorobutyl)benzene (4) 55

^{19}F NMR of 1-bromo-2-(4,4,4-trifluorobutyl)benzene (4) 56

^{1}H NMR of (5,5,5-trifluoropentyl)benzene (5) 57

^{19}F NMR of (5,5,5-trifluoropentyl)benzene (5) 58

^{1}H NMR of (4,4,4-trifluorobutoxy)benzene (6) 59

^{13}C NMR of (4,4,4-trifluorobutoxy)benzene (6) 60

^{19}F NMR of (4,4,4-trifluorobutoxy)benzene (6) 61

^{1}H NMR of 2-(9,9,9-Trifluorononyl)oxirane (8) 62

^{13}C NMR of 2-(9,9,9-Trifluorononyl)oxirane (8) 63

^{19}F NMR of 2-(9,9,9-Trifluorononyl)oxirane (8) 64

^{1}H NMR of 2-(4,4,4-trifluorobutyl)isoindoline-1,3-dione (9) 65

^{13}C NMR of 2-(4,4,4-trifluorobutyl)isoindoline-1,3-dione (9) 66

^{19}F NMR of 2-(4,4,4-trifluorobutyl)isoindoline-1,3-dione (9) 67

^{1}H NMR of nicotinic ester hydrotrifluoromethylated derivative (10) 68

^{13}C NMR of nicotinic ester hydrotrifluoromethylated derivative (10) 69

^{19}F NMR of nicotinic ester hydrotrifluoromethylated derivative (10) 70

^{1}H NMR of 5,5,5-trifluoropentyl 1-benzoylpiperidine-4-carboxylate (11) 71

^{13}C NMR of 5,5,5-trifluoropentyl 1-benzoylpiperidine-4-carboxylate (11) 72

^{19}F NMR of 5,5,5-trifluoropentyl 1-benzoylpiperidine-4-carboxylate (11)....73
^{1}H NMR of 2-methoxy-4-(4,4,4-trifluorobutyl)phenol (12)....74
^{19}F NMR of 2-methoxy-4-(4,4,4-trifluorobutyl)phenol (12)....75
^{13}C NMR of 2-methoxy-4-(4,4,4-trifluorobutyl)phenol (12)....76
^{1}H NMR of 2-(2H-benzo[d][1,2,3]triazol-2-yl)-4-methyl-6-(4,4,4-trifluorobutyl)phenol (13)....77
^{13}C NMR of 2-(2H-benzo[d][1,2,3]triazol-2-yl)-4-methyl-6-(4,4,4-trifluorobutyl)phenol (13)....78
^{19}F NMR of 2-(2H-benzo[d][1,2,3]triazol-2-yl)-4-methyl-6-(4,4,4-trifluorobutyl)phenol (13)....79
^{1}H NMR of ethyl 4-((6,6,6-trifluorohexanoyl)oxy)benzoate (14)....80
^{13}C NMR of ethyl 4-((6,6,6-trifluorohexanoyl)oxy)benzoate (14)....81
^{19}F NMR of ethyl 4-((6,6,6-trifluorohexanoyl)oxy)benzoate (14)....82
^{1}H NMR of febuxostat hydrotrifluoromethylated derivative (18)....83
^{13}C NMR of febuxostat hydrotrifluoromethylated derivative (18)....84
^{19}F NMR of febuxostat hydrotrifluoromethylated derivative (18)....85
^{1}H NMR of quinine hydrotrifluoromethylated derivative (19)....86
^{13}C NMR of quinine hydrotrifluoromethylated derivative (19)....87
^{19}F NMR of quinine hydrotrifluoromethylated derivative (19)....88
^{1}H NMR of vinclozolin hydrotrifluoromethylated derivative (20)....89
^{13}C NMR of vinclozolin hydrotrifluoromethylated derivative (20)....90
^{19}F NMR of vinclozolin hydrotrifluoromethylated derivative (20)....91
^{1}H NMR of theobromine hydrotrifluoromethylated derivative (21)....92
^{13}C NMR of theobromine hydrotrifluoromethylated derivative (21)....93
^{19}F NMR of theobromine hydrotrifluoromethylated derivative (21)....94
^{1}H NMR of indomethacin hydrotrifluoromethylated derivative (22)....95
^{13}C NMR of indomethacin hydrotrifluoromethylated derivative (22)....96
^{19}F NMR of indomethacin hydrotrifluoromethylated derivative (22)....97
^{1}H NMR of probenecid hydrotrifluoromethylated derivative (23)....98
^{13}C NMR of probenecid hydrotrifluoromethylated derivative (23)....99
^{19}F NMR of probenecid hydrotrifluoromethylated derivative (23)....100

^{1}H NMR of diethyl 3-methyl-4-(2,2,2-trifluoroethyl)cyclopentane-1,1-dicarboxylate (24) 101

^{13}C NMR of diethyl 3-methyl-4-(2,2,2-trifluoroethyl)cyclopentane-1,1-dicarboxylate (24) 102

^{19}F NMR of diethyl 3-methyl-4-(2,2,2-trifluoroethyl)cyclopentane-1,1-dicarboxylate (24) 103

REFERENCES 104

MATERIALS AND METHODS

All reactions were carried out under an ambient atmosphere unless otherwise stated and monitored by thin-layer chromatography (TLC). High-resolution mass spectra were obtained using a 1200 HPLC System (Agilent Technologies) with Direct loop injection (no Column), coupled to an Agilent 6230 time-of-flight (TOF) mass spectrometer with Electrospray Ionization (Agilent Technologies Inc.), utilizing a Dual ESI source. Concentration under reduced pressure was performed by rotary evaporation at 25–40 °C at an appropriate pressure. Yields refer to purified and spectroscopically pure compounds, unless otherwise stated.

Solvents

All deuterated solvents were purchased from Cambridge Isotope Laboratories. Inc. (CIL). Acetone and dichloromethane (DCM) were used directly from the commercial bottle, purchased from Fisher Chemical.

Chromatography

Thin layer chromatography (TLC) was performed using EMD TLC plates pre-coated with 250 µm thickness silica gel 60 F_{254} plates and visualized by fluorescence quenching under UV light (254 nm or 365 nm) or TLC stain (aqueous potassium permanganate solution followed by heating). Flash chromatography was performed using an Isolera purification system (Biotage, LLC) and Flash Purification Columns were purchased from Biotage LLC. Manual flash chromatography was performed using silica gel (40-63 µm particle size).

Electrochemistry

Electrochemical reactions were performed using IKA Electrasyn and working and counter graphite electrodes were purchased from IKA designed for Electrasyn. Cyclic voltammetry was conducted using EC-Lab and a BioLogic SP-50e potentiostat equipped with a glassy carbon disk working electrode (0.07 cm^2, BASi). The reference electrode consisted of an Ag wire immersed in acetone:DCM (1:3) containing 50 mM $AgNO_3$ was used. Pt wire was used as counter electrode.

Spectroscopy and Instruments

NMR spectra were recorded on Bruker Avance III HD 500 spectrometer operating at 500 MHz, 471 MHz, and 126 MHz, for ^1H, ^{19}F, and ^{13}C acquisitions, respectively. Chemical shifts are reported in ppm against tetramethylsilane (TMS, δ = 0.00 ppm) with the solvent residual peak as the internal standard. For ^1H NMR: $CDCl_3$, δ 7.26; CD_3CN, δ 1.96; DMSO-d_6, δ 2.50. For ^{13}C NMR: $CDCl_3$, δ 77.16; CD_3CN, δ 53.84; DMSO-d_6, δ 39.52. Data is reported as follows: s = singlet, d = doublet, dd = doublet of doublets, t = triplet, q = quartet, m = multiplet, bs = broad singlet; coupling constants are reported in Hz. GCMS analyses were recorded on TRACE 1610 (GC)-ISQ 7610 (Single Quadrupole MS) by ThermoFisher Scientific.

Starting materials

All substrates were used as received from commercial suppliers, unless otherwise stated. Starting materials (olefins, including **S1**) were prepared according to the literature.[1,2] $TT^+CF_3BF_4^-$ was prepared according to the

literature.[3] $MgBr_2$ was purchased from Sigma Aldrich.

EXPERIMENTAL DATA

Preparation of starting materials

Synthesis of olefins

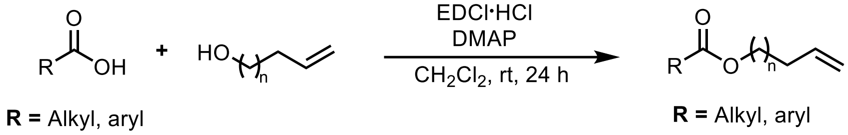

General procedure: A 100 mL round bottom flask equipped with a teflon-coated stirring bar was charged with the carboxylic acid (1.00 equiv) in CH_2Cl_2. Subsequently, the alcohol (1.10 – 1.33 equiv.), 1-ethyl-3-(3 dimethylaminopropyl)carbodiimide hydrochloride (1.00 – 1.33 equiv.), and 4-dimethylaminopyridine (0.40 – 1.00 equiv.) were added at 0 °C. The reaction was stirred for 24 h at room temperature. Then, the reaction mixture was diluted with water and extracted with CH_2Cl_2. The combined organic phases were dried over Na_2SO_4. The solvent was removed in vacuo and the crude product was purified via flash column chromatography to give the corresponding olefin derived product.[1]

Pent-4-en-1-yl nicotinate (S1)

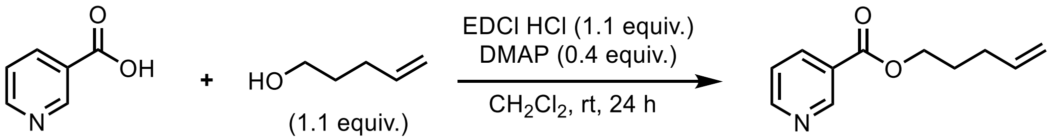

S1

A 100 mL round bottom flask equipped with a teflon-coated stirring bar was charged with nicotinic acid (1.50 g, 12.2 mmol, 1.00 equiv.) in 25 mL CH_2Cl_2. Subsequently, 4-penten-1-ol (1.34 mL, 1.12 g, 13.0 mmol, 1.06 equiv.), 1-ethyl-3-(3 dimethylaminopropyl)carbodiimide hydrochloride (2.50 g, 13.0 mmol, 1.07 equiv.), and 4-dimethylaminopyridine (0.60 g, 4.9 mmol, 0.40 equiv.) were added at 0 °C. The reaction was stirred for 24 h at room temperature. The resulting mixture was subsequently diluted with 25 mL CH_2Cl_2, washed by 1 M HCl (2 × ca. 50 mL), and saturated aqueous $NaHCO_3$ (50 mL). The combined organic phases were dried over Na_2SO_4, filtered, and the solvent was removed in vacuo and the crude product was purified via flash column chromatography (0-10% EtOAc in hexanes) to give the corresponding olefin derived product **S1** as a colorless oil (2.3 g, 12 mmol, 98%).

NMR Spectroscopy:

^{1}H NMR (500 MHz, $CDCl_3$, δ): 9.14 (d, J = 2.1 Hz, 1H), 8.68 (dd, J = 4.9, 1.8 Hz, 1H), 8.20 (dt, J = 8.0, 2.0 Hz, 1H), 7.30 (dd, J = 8.0, 4.8 Hz, 1H), 5.75 (ddt, J = 16.9, 10.2, 6.6 Hz, 1H), 5.06 – 4.89 (m, 2H), 4.28 (t, J = 6.6 Hz, 2H), 2.13 (q, J = 7.2 Hz, 2H), 1.80 (p, J = 6.9 Hz, 2H).

^{13}C NMR (126 MHz, $CDCl_3$, δ): 165.1, 153.3, 150.8, 137.2, 136.9, 126.2, 123.2, 115.4, 64.7, 30.0, 27.7.

HRMS-ESI(m/z) calc'd for $C_{11}H_{13}NO_2^+$ $[M+H]^+$, 192.1019; found, 192.1028; deviation: +4.4 ppm.

General procedure for electroreductive hydrotrifluoromethylation of unactivated olefins

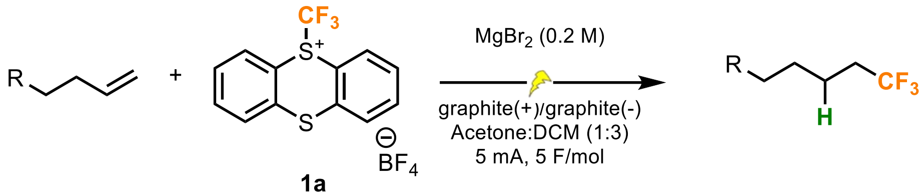

1a

(1.0 equiv) (2.2 equiv)

Under ambient conditions, to a 5 mL ElectraSyn vial equipped with a magnetic stir bar were added olefin (1.00 equiv.) (if solid) S-(Trifluoromethyl)thianthrenium tetrafluoroborate (2.20 equiv.) and magnesium bromide (8.00 equiv.). Subsequently, dichloromethane and acetone (3:1) (c = 0.025 M), olefin (1.00 equiv.) (if liquid), were added at 25 °C. The ElectraSyn vial was equipped with two electrodes: graphite (+) and graphite (-) and sealed with an ElectraSyn septum-cap. The reaction mixture was electrolyzed (electrolysis parameters: constant current: 5 mA, amount of charge: 5 F/mol and stirring: 1500 rpm). After reaction completion, the ElectraSyn vial cap was removed and the electrodes were rinsed with acetone, which was combined with the reaction mixture. The yield of the hydrotrifluoromethylation product was determined by ^{1}H NMR spectroscopy using mesitylene as internal standard and ^{19}F NMR spectroscopy using trifluorotoluene as internal standard. The reaction mixture was subsequently concentrated under reduced pressure and the residue was purified by flash column chromatography on silica gel to afford the title compound.

Note: For some substrates, the dibromination product was observed in <5% as a side product resulting from Br_2 *in situ* production.

Note: After completion of the reaction, bromoacetone was detected by ^{1}H-NMR spectrum. Bromoacetone is known to be a lachrymator, thus the work-up of the reaction was conducted in a well-ventilated fume hood.

Note: The reaction proceeds using technical-grade acetone and dichloromethane (DCM) as solvents.

Note: For some substrates trifluoroacetic acid (TFA) as an additive was added to protect sensitive functional groups or increase the conversion of the olefin.

Alternative procedure for hydrotrifluoromethylation of unactivated olefins

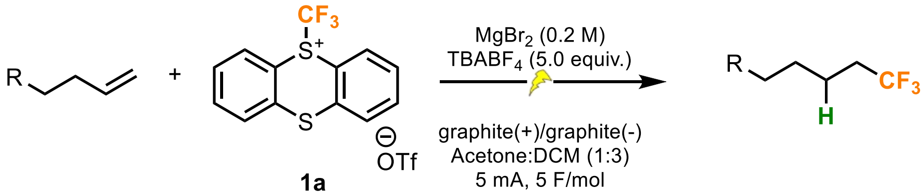

1a

(1.0 equiv) (2.2 equiv)

Under ambient conditions, to a 5 mL ElectraSyn vial equipped with a magnetic stir bar were added olefin (1.00 equiv.) (if solid) S-(Trifluoromethyl)thianthrenium triflate (2.20 equiv.), $TBABF_4$ (5.00 equiv.), magnesium bromide (8.00 equiv.）. Subsequently, dichloromethane and acetone (3:1) (c = 0.025 M), olefin (1.00 equiv.) (if liquid), were added at 25 °C. The ElectraSyn vial was equipped with two electrodes: graphite (+), graphite (-) and sealed with an ElectraSyn septum-cap. The reaction mixture was electrolyzed (electrolysis parameters: constant current: 5 mA, amount of charge: 5 F/mol and stirring: 1500 rpm). After reaction completion, the ElectraSyn vial cap was removed and the electrodes were rinsed with acetone, which was combined with the reaction mixture. The yield of the hydrotrifluoromethylation product was determined by ^1H NMR spectroscopy using mesitylene as internal standard and ^{19}F NMR spectroscopy using trifluorotoluene as internal standard. The reaction mixture was subsequently concentrated under reduced pressure and the residue was purified by flash column chromatography on silica gel to afford the title compound in comparable yields as the general procedure.

1-Methoxy-4-(4,4,4-trifluorobutyl)benzene (2)

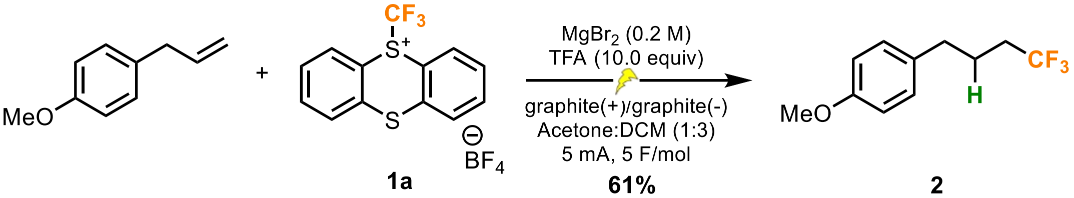

(1.0 equiv) **1a** (2.2 equiv) **2**

Under ambient conditions, to a 10 mL ElectraSyn vial equipped with a magnetic stir bar were added S-(Trifluoromethyl)thianthrenium tetrafluoroborate (0.20 g, 0.54 mmol, 2.2 equiv.) and magnesium bromide (0.37 g, 2.0 mmol, 8.0 equiv.). Subsequently, dichloromethane (7.5 mL) and acetone (2.5 mL) (3:1) (c = 0.025 M), 4-allylanisole (39 µL, 0.25 mmol, 1.0 equiv.), and trifluoroacetic acid (0.19 mL, 2.5 mmol, 10.0 equiv.) were added at 25 °C. The ElectraSyn vial was equipped with two electrodes: graphite (+), graphite (-) and sealed with an ElectraSyn septum-cap. The reaction mixture was electrolyzed (electrolysis parameters: constant current: 5 mA, amount of charge: 5 F/mol and stirring: 1500 rpm). After reaction completion, the ElectraSyn vial cap was removed and the electrodes were rinsed with acetone, which was combined with the reaction mixture in a flask. The yield (85%) of **2** was determined by ^1H NMR spectroscopy using mesitylene as internal standard. And the yield (81%) of **2** was also determined by ^{19}F NMR spectroscopy using trifluorotoluene as internal standard. Saturated sodium bicarbonate solution (10 mL) was added to the flask containing the reaction mixture and the reaction crude was stirred for 10 min. Then, the aqueous phase was washed with dichloromethane (3 × 10 mL), dried over magnesium sulfate and concentrated *in vacuo*. The residue was purified by flash column chromatography on silica gel eluting with hexane/ethyl acetate (100:0 (v/v)) to afford the title compound **2** as a colorless oil (33 mg, 0.15 mmol, 61%).

NMR Spectroscopy:

^{1}H NMR (500 MHz, $CDCl_3$, δ): 7.09 (d, *J* = 8.5 Hz, 2H), 6.85 (d, *J* = 8.6 Hz, 2H), 3.80 (s, 3H), 2.64 (t, *J* = 7.6 Hz, 2H), 2.13 – 2.01 (m, 2H), 1.86 (p, *J* = 7.6 Hz, 2H).

^{13}C NMR (126 MHz, $CDCl_3$, δ): 158.2, 132.9, 129.4, 127.4 (q, *J* = 276.2 Hz), 114.1, 55.4, 33.9, 33.2 (q, *J* = 28.4 Hz), 23.9 (q, *J* = 2.8 Hz).

^{19}F NMR (470 MHz, $CDCl_3$, δ): -66.18 (t, *J* = 10.9 Hz).

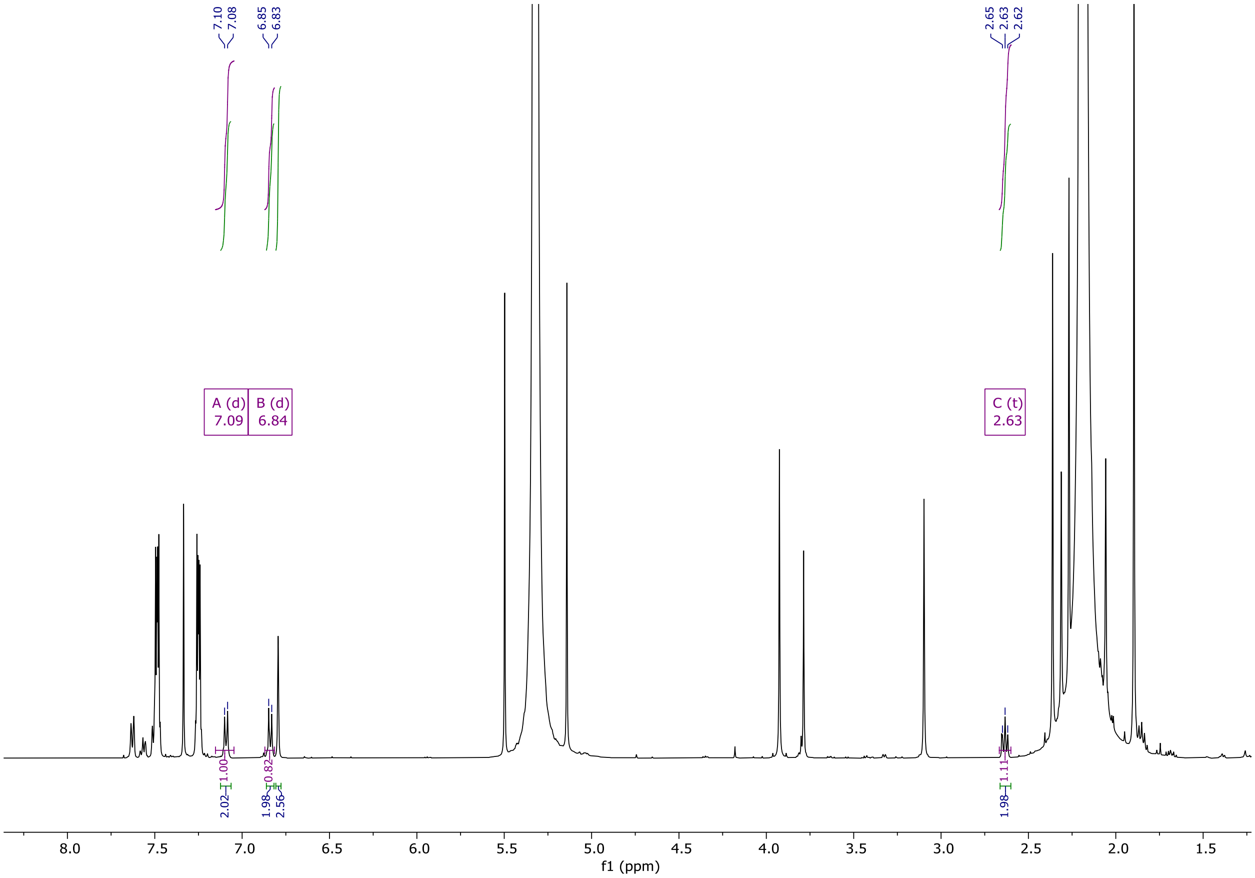

Figure S1: ^{1}H-NMR of reaction mixture of electrochemical hydrotrifluoromethylation of 4-methoxyallylbenzene using mesitylene as internal standard ($CDCl_3$, RT).

Protocol in absence of TFA

1-Methoxy-4-(4,4,4-trifluorobutyl)benzene (2)

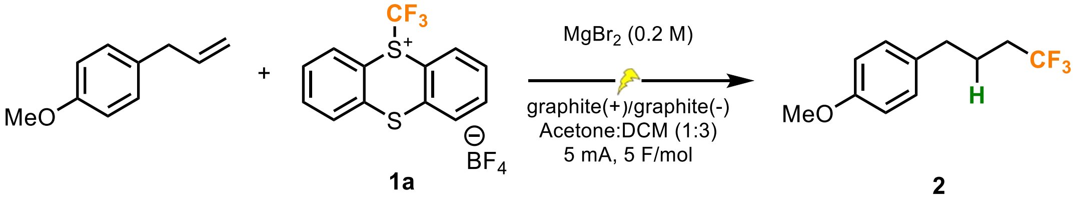

(1.0 equiv) **1a** (2.2 equiv) **2**

Under ambient conditions, to a 5 mL ElectraSyn vial equipped with a magnetic stir bar were added S-(Trifluoromethyl)thianthrenium tetrafluoroborate (81.0 mg, 0.220 mmol, 2.2 equiv.) and magnesium bromide (0.15 g, 0.80 mmol, 8.0 equiv.). Subsequently, dichloromethane (3.0 mL), acetone (1.0 mL) (3:1) (c = 0.025 M), and 4-allylanisole (15 µL, 0.10 mmol, 1.0 equiv.) were added at 25 °C. The ElectraSyn vial was equipped with two electrodes: graphite (+), graphite (-) and sealed with an ElectraSyn septum-cap. The reaction mixture was electrolyzed (electrolysis parameters: constant current: 5 mA, amount of charge: 5 F/mol and stirring: 1500 rpm). After reaction completion, the ElectraSyn vial cap was removed and the electrodes were rinsed with acetone, which was combined with the reaction mixture in a flask. The yield (64%) of **2** was determined by ^{19}F NMR spectroscopy using 10 µL of trifluorotoluene as internal standard alongside remaining starting material, 4-allylanisole (18%) determined by ^{1}H NMR spectroscopy using 10 µL of mesitylene as internal standard.

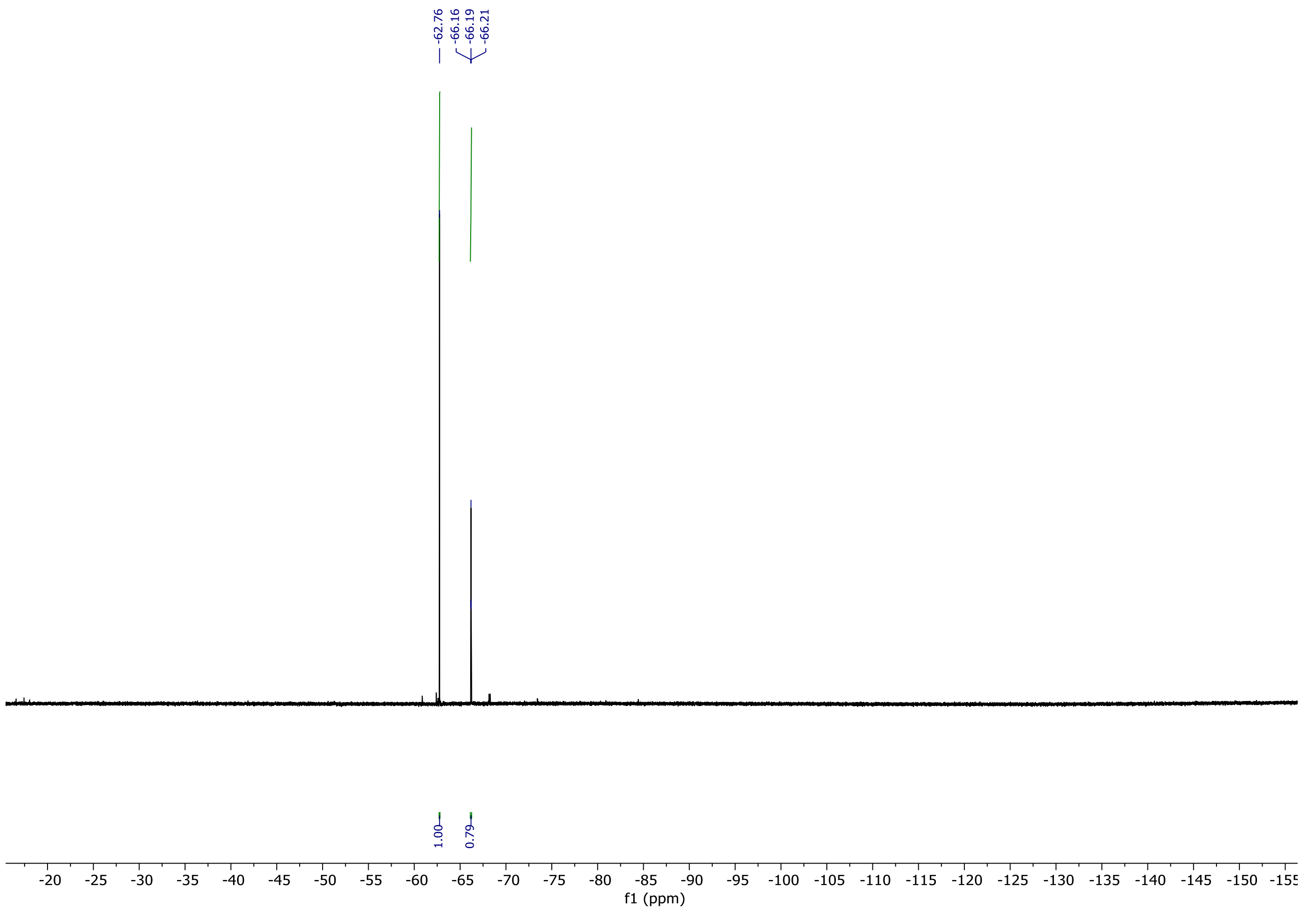

Figure S2: ^{19}F -NMR of reaction mixture of electrochemical hydrotrifluoromethylation of 4-methoxyallylbenzene using trifluorotoluene as internal standard ($CDCl_3$, RT).

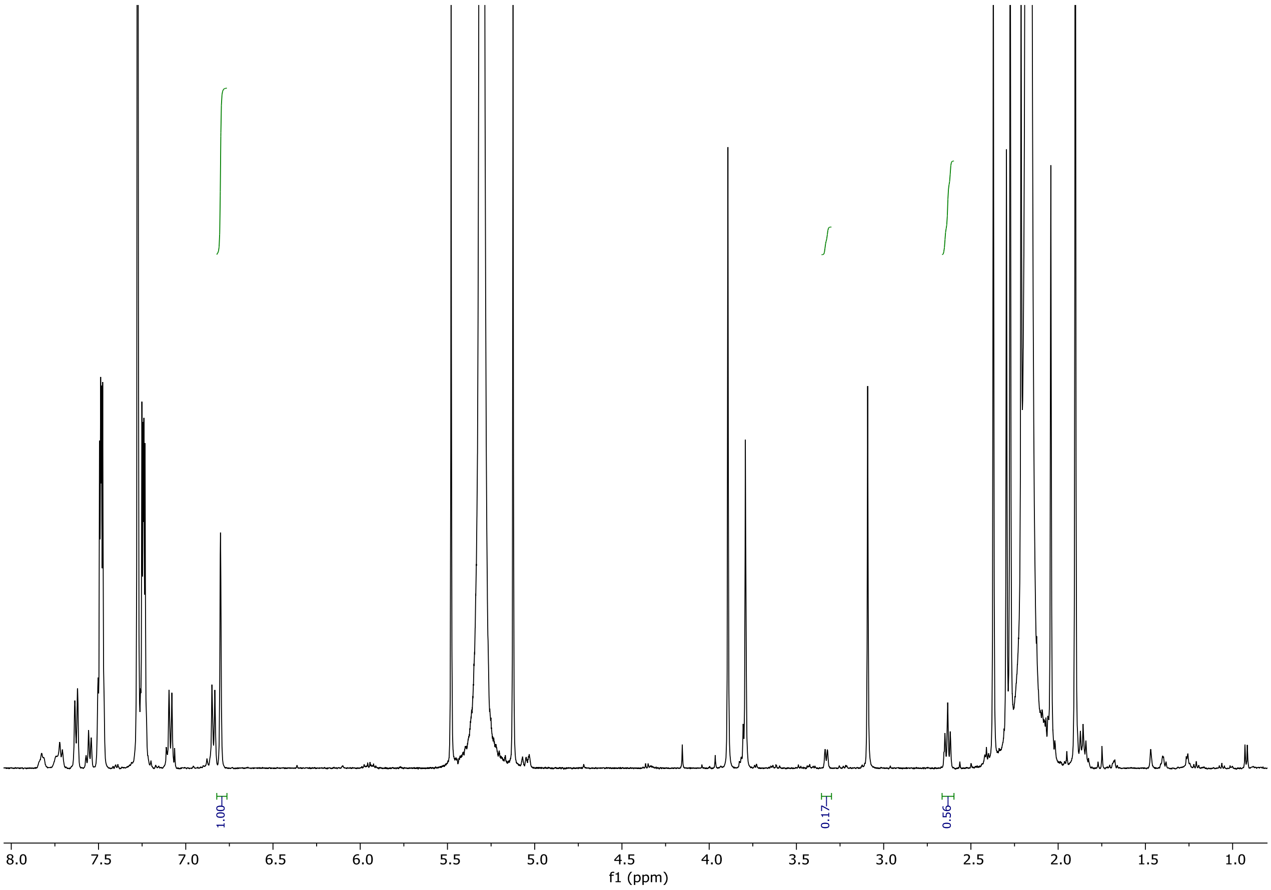

Figure S3: ^{1}H-NMR of reaction mixture of electrochemical hydrotrifluoromethylation of 4-methoxyallylbenzene using mesitylene as internal standard ($CDCl_3$, RT).

1-Fluoro-4-(4,4,4-trifluorobutyl)benzene (3)

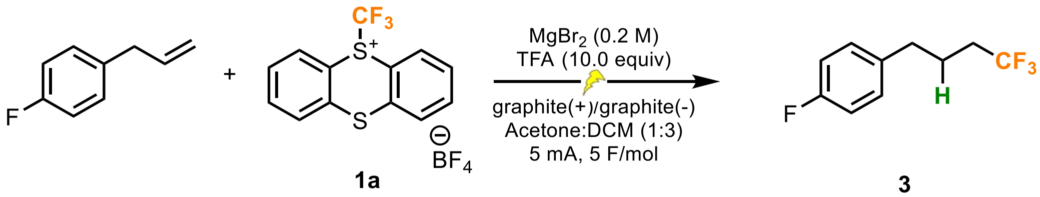

Under ambient conditions, to a 10 mL ElectraSyn vial equipped with a magnetic stir bar were added S-(Trifluoromethyl)thianthrenium tetrafluoroborate (0.20 g, 0.54 mmol, 2.2 equiv.) and magnesium bromide (0.37 g, 2.00 mmol, 8.0 equiv.). Subsequently, dichloromethane (7.5 mL) and acetone (2.5 mL) (3:1) (c = 0.025 M), 4-allylfluorobenzene (34 µL, 0.25 mmol, 1.0 equiv.), and trifluoroacetic acid (0.19 mL, 2.5 mmol, 10.0 equiv.) were added at 25 °C. The ElectraSyn vial was equipped with two electrodes: graphite (+), graphite (-) and sealed with an ElectraSyn septum-cap. The reaction mixture was electrolyzed (electrolysis parameters: constant current: 5 mA, amount of charge: 5 F/mol and stirring: 1500 rpm). After reaction completion, the yield

(64%) of **3** was determined by ^{19}F NMR spectroscopy using trifluorotoluene as internal standard at δ -66.37 (t, *J* = 10.8 Hz).

Note: The compound is volatile and only ^{19}F- NMR yield was reported.

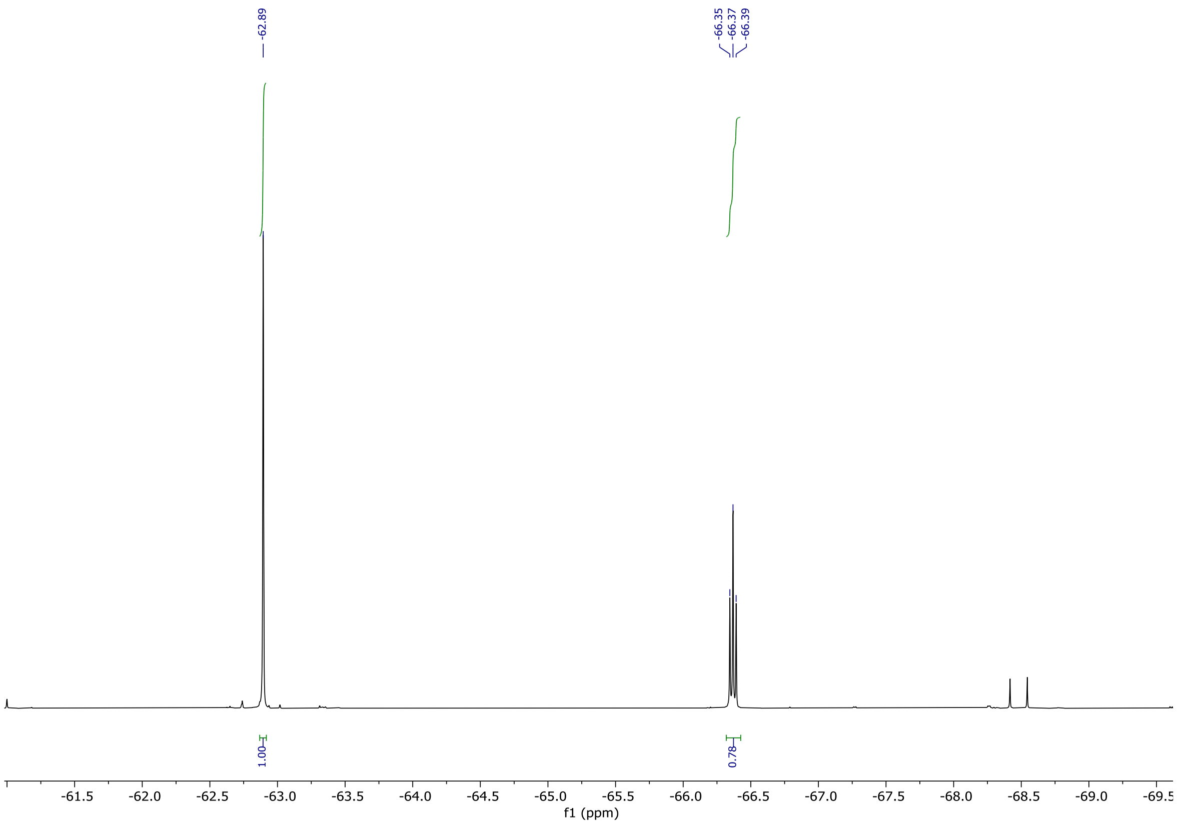

Figure S4: Quantitative ^{19}F -NMR of reaction mixture of electrochemical hydrotrifluoromethylation of 4-fluoroallylbenzene using trifluorotoluene as internal standard ($CDCl_3$, RT).

1-Bromo-2-(4,4,4-trifluorobutyl)benzene (4)

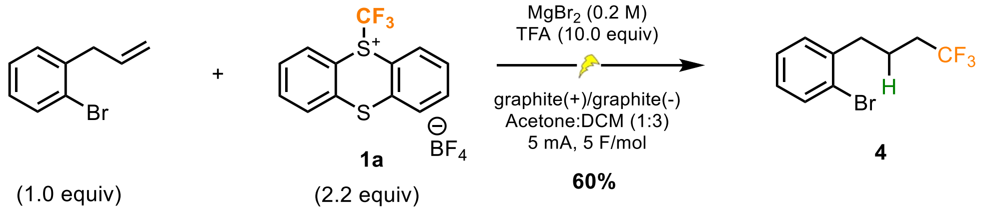

Under ambient conditions, to a 10 mL ElectraSyn vial equipped with a magnetic stir bar were added S-(Trifluoromethyl)thianthrenium tetrafluoroborate (0.20 g, 0.55 mmol, 2.2 equiv.) and magnesium bromide (0.37 g, 2.0 mmol, 8.0 equiv.). Subsequently, dichloromethane (7.5 mL), acetone (2.5 mL) (3:1) (c = 0.025 M), 1-allyl-2-bromobenzene (38 µL, 0.25 mmol, 1.0 equiv.), and trifluoroacetic acid (0.19 mL, 2.5 mmol, 10 equiv.) were added at 25 °C. The ElectraSyn vial was equipped with two electrodes: graphite (+), graphite (-) and sealed with an ElectraSyn septum-cap. The reaction mixture was electrolyzed (electrolysis parameters:

constant current: 5 mA, amount of charge: 5 F/mol and stirring: 1500 rpm). After reaction completion, the ElectraSyn vial cap was removed and the electrodes were rinsed with acetone, which was combined with the reaction mixture in a flask. The yield (64%) of **4** was determined by ^{1}H NMR spectroscopy using mesitylene as internal standard. Saturated sodium bicarbonate solution (10 mL) was added to the flask containing the reaction mixture and the reaction crude was stirred for 10 min. Then, the aqueous phase was washed with dichloromethane (3 × 10 mL), dried over magnesium sulfate and concentrated *in vacuo*. The residue was purified by flash column chromatography on silica gel eluting with hexane/ethyl acetate (100:0 (v/v)) to afford the title compound **4** as a colorless liquid (40 mg, 0.15 mmol, 60%).

NMR Spectroscopy:

^{1}H NMR (500 MHz, $CDCl_3$, δ): 7.55 (dd, J = 8.0, 1.3 Hz, 1H), 7.26 (td, J = 7.4, 1.3 Hz, 1H), 7.21 (dd, J = 7.6, 1.9 Hz, 1H), 7.09 (td, J = 7.6, 1.9 Hz, 1H), 2.82 (t, J = 7.8 Hz, 2H), 2.23 – 2.05 (m, 2H), 1.99 – 1.85 (m, 2H).

^{13}C NMR (126 MHz, $CDCl_3$, δ): 140.2, 133.2, 130.4, 128.2, 127.7, 127.2 (q, *J* = 276.3 Hz), 124.6, 35.1, 33.3 (q, *J* = 28.6 Hz), 22.3 (q, *J* = 2.9 Hz).

^{19}F NMR (470 MHz, $CDCl_3$, δ): -66.17 (t, J = 10.8 Hz).

(5,5,5-Trifluoropentyl)benzene (5)

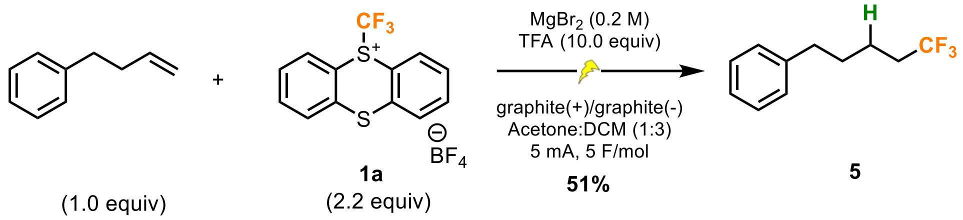

Under ambient conditions, to a 10 mL ElectraSyn vial equipped with a magnetic stir bar were added S-(Trifluoromethyl)thianthrenium tetrafluoroborate (0.20 g, 0.55 mmol, 2.2 equiv.) and magnesium bromide (0.37 g, 2.0 mmol, 8.0 equiv.). Subsequently, dichloromethane (7.5 mL), acetone (2.5 mL) (3:1) (c = 0.025 M), 3-butenylbenzene (38 µL, 0.25 mmol, 1.0 equiv.), and trifluoroacetic acid (0.19 mL, 2.5 mmol, 10 equiv.) were added at 25 °C. The ElectraSyn vial was equipped with two electrodes: graphite (+), graphite (-) and sealed with an ElectraSyn septum-cap. The reaction mixture was electrolyzed (electrolysis parameters: constant current: 5 mA, amount of charge: 5 F/mol and stirring: 1500 rpm). After reaction completion, the ElectraSyn vial cap was removed and the electrodes were rinsed with acetone, which was combined with the reaction mixture in a flask. The yield (64%) of **5** was determined by ^{1}H NMR spectroscopy using mesitylene as internal standard. Saturated sodium bicarbonate solution (10 mL) was added to the flask containing the reaction mixture and the reaction crude was stirred for 10 min. Then, the aqueous phase was washed with dichloromethane (3 × 10 mL), dried over magnesium sulfate and concentrated *in vacuo*. The residue was purified by flash column chromatography on silica gel eluting with hexane/ethyl acetate (100:0 (v/v)) to afford the title compound **5** as a colorless liquid (26 mg, 0.13 mmol, 51%). The product was isolated as an inseparable 35:1 mixture of

regioisomers.

NMR Spectroscopy:

^{1}H NMR (500 MHz, $CDCl_3$, δ): 7.29 (t, *J* = 7.5 Hz, 2H), 7.22 – 7.12 (m, 3H), 2.64 (t, *J* = 7.6 Hz, 2H), 2.17 – 2.01 (m, 2H), 1.71 (p, *J* = 7.4 Hz, 2H), 1.66 – 1.56 (m, 2H).

^{19}F NMR (470 MHz, $CDCl_3$, δ): -66.30 (t, *J* = 11.1 Hz).

The spectroscopic data for this compound were identical to those reported in the literature.[4]

(4,4,4-Trifluorobutoxy)benzene (6)

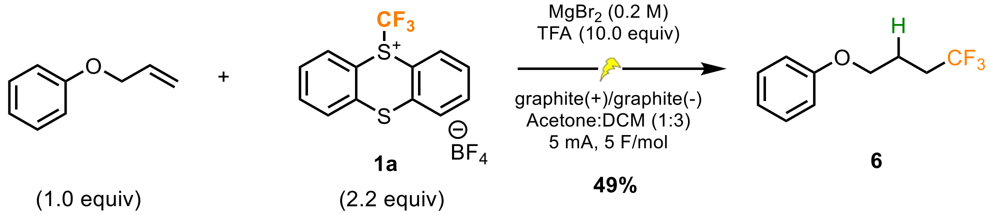

(1.0 equiv) | **1a** (2.2 equiv) | **49%** | **6**

Under ambient conditions, to a 10 mL ElectraSyn vial equipped with a magnetic stir bar were added S-(Trifluoromethyl)thianthrenium tetrafluoroborate (0.20 g, 0.55 mmol, 2.2 equiv.) and magnesium bromide (0.37 g, 2.0 mmol, 8.0 equiv.). Subsequently, dichloromethane (7.5 mL), acetone (2.5 mL) (3:1) (c = 0.025 M), allylphenylether (34 µL, 0.25 mmol, 1.0 equiv.), and trifluoroacetic acid (0.19 mL, 2.5 mmol, 10 equiv.) were added at 25 °C. The ElectraSyn vial was equipped with two electrodes: graphite (+), graphite (-) and sealed with an ElectraSyn septum-cap. The reaction mixture was electrolyzed (electrolysis parameters: constant current: 5 mA, amount of charge: 5 F/mol and stirring: 1500 rpm). After reaction completion, the ElectraSyn vial cap was removed and the electrodes were rinsed with acetone, which was combined with the reaction mixture in a flask. The yield (75%) of **6** was determined by ^{19}F NMR spectroscopy using trifluorotoluene as internal standard. Saturated sodium bicarbonate solution (10 mL) was added to the flask containing the reaction mixture and the reaction crude was stirred for 10 min. Then the aqueous phase was washed with dichloromethane (3 × 10 mL), dried over magnesium sulfate and concentrated *in vacuo*. The residue was purified by flash column chromatography on silica gel eluting with hexane/ethyl acetate (100:0 (v/v)) to afford the title compound **6** as a colorless oil (25 mg, 0.12 mmol, 49%). The product was isolated as a single regioisomer, however the crude NMR shows a >40:1 mixture of regioisomers.

NMR Spectroscopy:

^{1}H NMR (500 MHz, $CDCl_3$, δ): 7.30 (dd, *J* = 8.7, 7.3 Hz, 2H), 6.97 (t, *J* = 7.3 Hz, 1H), 6.90 (d, *J* = 8.1 Hz, 2H), 4.02 (t, *J* = 6.0 Hz, 2H), 2.42 – 2.26 (m, 2H), 2.14 – 2.00 (m, 2H).

^{13}C NMR (126 MHz, $CDCl_3$, δ): 158.7, 129.7, 127.3 (q, *J* = 276.1 Hz), 121.2, 114.6, 66.0, 30.9 (q, *J* = 29.1 Hz), 22.4 (q, *J* = 3.0 Hz).

^{19}F NMR (470 MHz, $CDCl_3$, δ): -66.37 (t, *J* = 10.9 Hz).

N-(4,4,4-Trifluorobutyl)aniline (7)

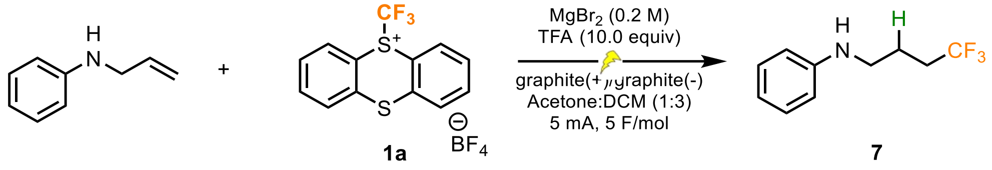

Under ambient conditions, to a 5 mL ElectraSyn vial equipped with a magnetic stir bar were added S-(Trifluoromethyl)thianthrenium tetrafluoroborate (81.0 mg, 0.22 mmol, 2.2 equiv.) and magnesium bromide (0.15 g, 2.0 mmol, 0.80 equiv.). Subsequently, dichloromethane (3.0 mL), acetone (1.0 mL) (3:1) (c = 0.025 M), N-allyl-N-phenylamine (14 µL, 0.10 mmol, 1.0 equiv.), and trifluoroacetic acid (0.19 mL, 2.5 mmol, 10 equiv.) were added at 25 °C. The ElectraSyn vial was equipped with two electrodes: graphite (+), graphite (-) and sealed with an ElectraSyn septum-cap. After reaction completion, the reaction mixture was electrolyzed (electrolysis parameters: constant current: 5 mA, amount of charge: 5 F/mol and stirring: 1500 rpm). The yield (49%) of **7** was determined by ^{19}F NMR spectroscopy using trifluorotoluene as internal standard.

Note: The compound is known in the literature and only ^{19}F- NMR yield was reported at -66.00 (t, J = 10.0 Hz).

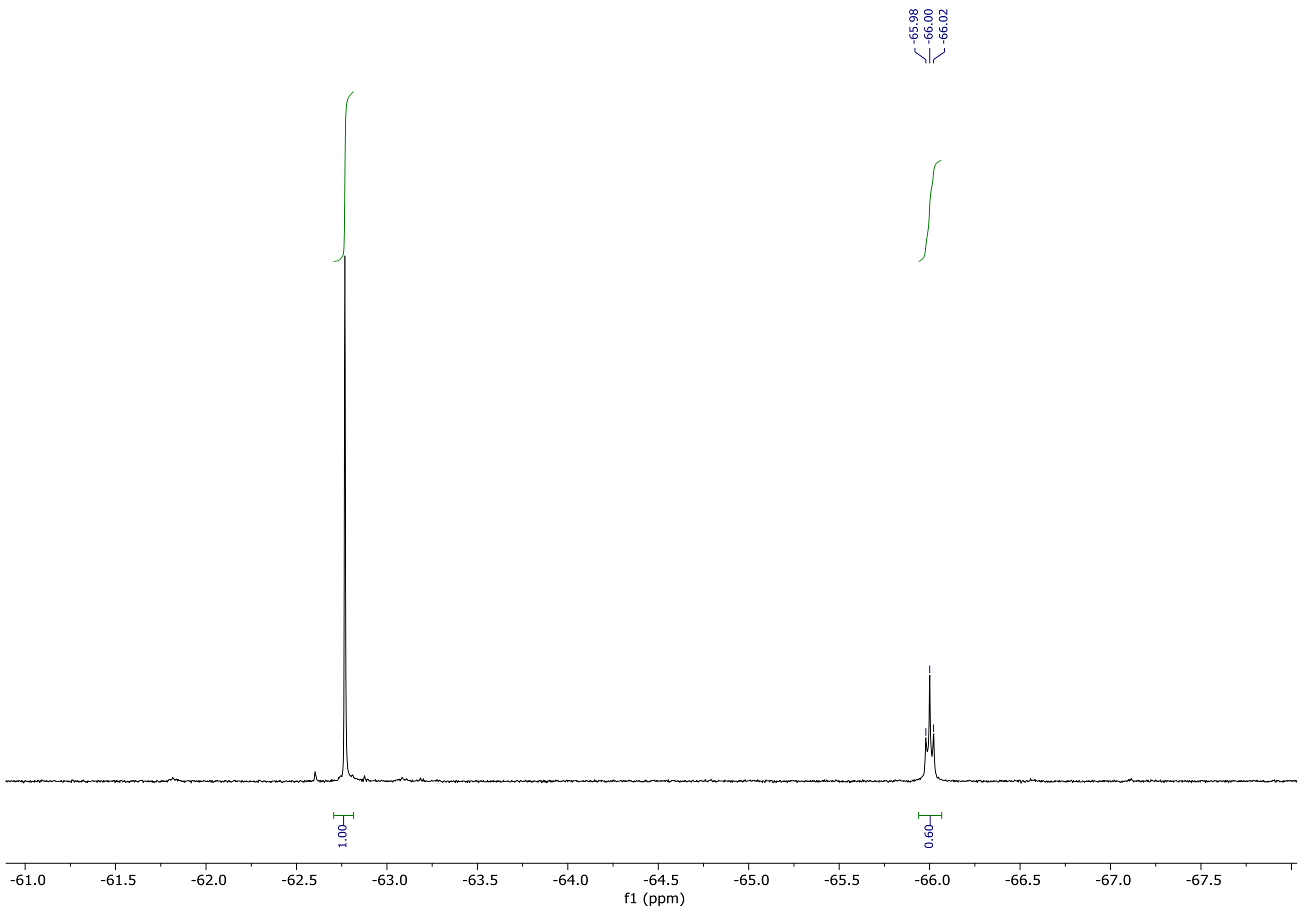

Figure S5: Quantitative ^{19}F -NMR of reaction mixture of electrochemical hydrotrifluoromethylation of N-allyl-

N-phenylamine using trifluorotoluene as internal standard ($CDCl_3$, RT).

2-(9,9,9-Trifluorononyl)oxirane (8)

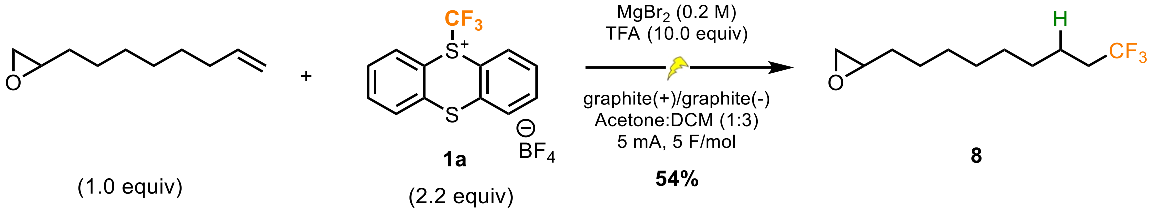

Under ambient conditions, to a 10 mL ElectraSyn vial equipped with a magnetic stir bar were added, S-(Trifluoromethyl)thianthrenium tetrafluoroborate (0.20 g, 0.54 mmol, 2.2 equiv.) and magnesium bromide (0.37 g, 2.0 mmol, 8.0 equiv.). Subsequently, dichloromethane (7.5 mL), acetone (2.5 mL) (3:1) (c = 0.025 M), 1,2-epoxy-9-decene (46 µL, 0.25 mmol, 1.0 equiv.), and trifluoroacetic acid (0.19 mL, 2.5 mmol, 10 equiv.) were added at 25 °C. The ElectraSyn vial was equipped with two electrodes: graphite (+), graphite (-) and sealed with an ElectraSyn septum-cap. The reaction mixture was electrolyzed (electrolysis parameters: constant current: 5 mA, amount of charge: 5 F/mol and stirring: 1500 rpm). After reaction completion, the ElectraSyn vial cap was removed and the electrodes were rinsed with acetone, which was combined with the reaction mixture in a flask. Saturated sodium bicarbonate solution (10 mL) was added to the flask containing the reaction mixture and the reaction crude was stirred for 10 min. Then, the aqueous phase was washed with dichloromethane (3 × 10 mL), dried over magnesium sulfate and concentrated *in vacuo*. The residue was purified by flash column chromatography on silica gel eluting with hexanes to afford the title compound **8** as a colorless oil (30 mg, 0.13 mmol, 54%). The product was isolated as an inseparable 34:1 mixture of regioisomers.

NMR Spectroscopy:

^{1}H NMR (500 MHz, $CDCl_3$, δ): 3.84 – 3.70 (m, 1H), 3.54 (dd, J = 10.3, 3.2 Hz, 1H), 3.38 (dd, J = 10.3, 7.1 Hz, 1H), 2.13 – 2.00 (m, 2H), 1.67 – 1.50 (m, 4H), 1.47 – 1.27 (m, 10H).

^{13}C NMR (126 MHz, $CDCl_3$, δ): 127.4 (q, J = 276.4 Hz), 71.2, 40.8, 35.2, 33.9 (q, J = 28.3 Hz), 29.5, 29.3, 29.2, 28.8, 25.7, 22.0 (q, J = 2.9 Hz).

^{19}F NMR (470 MHz, $CDCl_3$, δ): -66.40 (t, J = 11.0 Hz).

GCMS-EI(m/z) calc'd for $C_{11}H_{19}F_3O^+$ [M]$^+$, 224.1383; found, 224.1384; deviation: +0.4 ppm.

2-(4,4,4-Trifluorobutyl)isoindoline-1,3-dione (9)

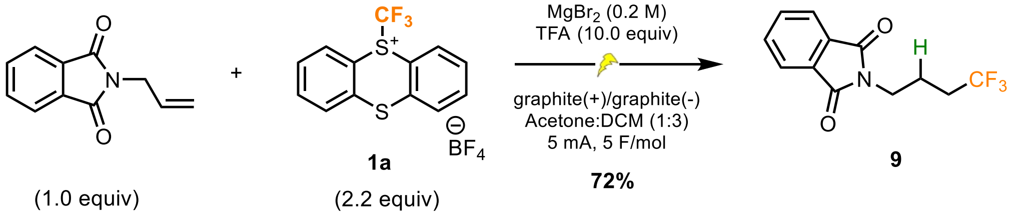

Under ambient conditions, to a 10 mL ElectraSyn vial equipped with a magnetic stir bar were added N-allylphthalimide (46.8 mg, 0.250 mmol, 1.00 equiv.), S-(Trifluoromethyl)thianthrenium tetrafluoroborate (0.20 g, 0.55 mmol, 2.2 equiv.) and magnesium bromide (0.37 g, 2.0 mmol, 8.0 equiv.). Subsequently, dichloromethane (7.5 mL), acetone (2.5 mL) (3:1) (c = 0.025 M), and trifluoroacetic acid (0.19 mL, 2.5 mmol, 10 equiv.) were added at 25 °C. The ElectraSyn vial was equipped with two electrodes: graphite (+), graphite (-) and sealed with an ElectraSyn septum-cap. The reaction mixture was electrolyzed (electrolysis parameters: constant current: 5 mA, amount of charge: 5 F/mol and stirring: 1500 rpm). After reaction completion, the ElectraSyn vial cap was removed and the electrodes were rinsed with acetone, which was combined with the reaction mixture in a flask. Saturated sodium bicarbonate solution (10 mL) was added to the flask containing the reaction mixture and the reaction crude was stirred for 10 min. Then, the aqueous phase was washed with dichloromethane (3 × 10 mL), dried over magnesium sulfate and concentrated *in vacuo*. The residue was purified by flash column chromatography on silica gel eluting with hexane/ethyl acetate (100:0 (v/v)) to afford the title compound **9** as a white solid (46 mg, 0.18 mmol, 72%). The product was isolated as a single regioisomer, however the crude NMR shows 44:1 mixture of regioisomers.

NMR Spectroscopy:

^{1}**H NMR** (500 MHz, CD_3CN, δ): 7.86 – 7.80 (m, 2H), 7.80 – 7.74 (m, 2H), 3.69 (t, *J* = 6.9 Hz, 2H), 2.31 – 2.18 (m, 2H), 1.93 – 1.86 (m, 2H).

^{13}**C NMR** (126 MHz, CD_3CN, δ): 169.3, 135.2, 133.2, 128.5 (q, *J* = 275.4 Hz), 123.8, 37.5, 31.7 (q, *J* = 28.8 Hz), 22.1 (q, *J* = 3.1 Hz).

^{19}**F NMR** (470 MHz, CD_3CN, δ): -67.00 (t, *J* = 11.2 Hz).

Nicotinic ester hydrotrifluoromethylated derivative (10)

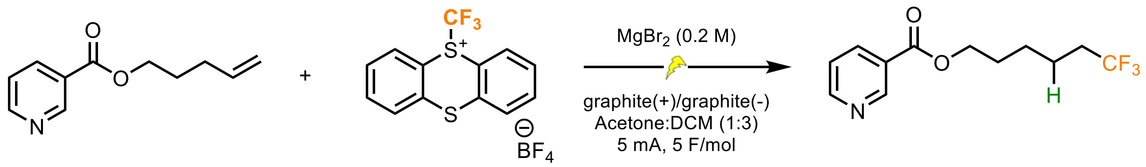

S1 (1.0 equiv) **1a** (2.2 equiv) **79%** **10**

Under ambient conditions, to a 10 mL ElectraSyn vial equipped with a magnetic stir bar were added nicotinic ester derived olefin (47.1 mg, 0.246 mmol, 1.00 equiv.), S-(Trifluoromethyl)thianthrenium tetrafluoroborate (0.20 g, 0.54 mmol, 2.2 equiv.) and magnesium bromide (0.35 g, 1.90 mmol, 7.7 equiv.). Subsequently, dichloromethane (7.5 mL) and acetone (2.5 mL) (3:1) (c = 0.025 M) were added at 25 °C. The ElectraSyn vial was equipped with two electrodes: graphite (+), graphite (-) and sealed with an ElectraSyn septum-cap. The reaction mixture was electrolyzed (electrolysis parameters: constant current: 5 mA, amount of charge: 5 F/mol and stirring: 1500 rpm). After reaction completion, the ElectraSyn vial cap was removed and the electrodes were rinsed with acetone, which was combined with the reaction mixture in a round bottom flask. The reaction mixture was subsequently concentrated under reduced pressure and the residue was purified by flash column

chromatography on silica gel eluting with Et_3N/hexane/ethyl acetate (3/97/0 to 3/57/40 (v/v)) to afford the title compound **10** as a colorless oil (51.0 mg, 0.195 mmol, 79%). The product was isolated as an inseparable 14:1 mixture of regioisomers.

NMR Spectroscopy:

^{1}H NMR (500 MHz, CD_3CN, δ): 9.14 (d, *J* = 1.7 Hz, 1H), 8.76 (dd, *J* = 4.9, 1.7 Hz, 1H), 8.28 (dt, *J* = 7.9, 2.0 Hz, 1H), 7.46 (dd, *J* = 7.7, 4.8 Hz, 1H), 4.33 (t, *J* = 6.5 Hz, 2H), 2.26 – 2.12 (m, 2H), 1.84 – 1.75 (m, 2H), 1.67 – 1.57 (m, 2H), 1.57 – 1.48 (m, 2H).

^{13}C NMR (126 MHz, CD_3CN, δ): 166.2, 154.5, 151.4, 137.7, 128.8 (q, *J* = 275.4 Hz), 127.3, 124.5, 65.9, 33.9 (q, *J* = 28.0 Hz), 28.9, 25.8, 22.3 (q, *J* = 3.1 Hz).

^{19}F NMR (470 MHz, $CDCl_3$, δ): -66.97 (t, *J* = 11.5 Hz)

HRMS-ESI(m/z) calc'd for $C_{12}H_{14}F_3NO_2^+$ $[M+H]^+$, 262.1049; found, 262.1060; deviation: +4.2 ppm.

5,5,5-Trifluoropentyl 1-benzoylpiperidine-4-carboxylate (11)

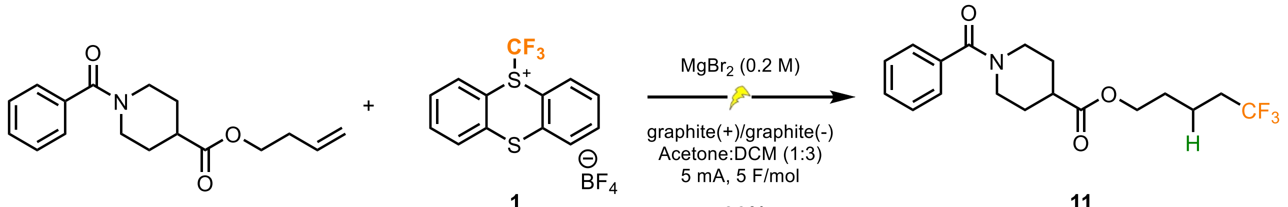

(1.0 equiv) + **1** (2.2 equiv) → **90%** **11**

Under ambient conditions, to a 10 mL ElectraSyn vial equipped with a magnetic stir bar were added piperidine-derived alkene (71.8 mg, 0.250 mmol, 1.00 equiv.), S-(Trifluoromethyl)thianthrenium tetrafluoroborate (0.20 g, 0.55 mmol, 2.2 equiv.) and magnesium bromide (0.37 g, 2.0 mmol, 8.0 equiv.). Subsequently, dichloromethane (7.5 mL) and acetone (2.5 mL) (3:1) (c = 0.025 M) were added at 25 °C. The ElectraSyn vial was equipped with two electrodes: graphite (+), graphite (-) and sealed with an ElectraSyn septum-cap. The reaction mixture was electrolyzed (electrolysis parameters: constant current: 5 mA, amount of charge: 5 F/mol and stirring: 1500 rpm). After reaction completion, the ElectraSyn vial cap was removed and the electrodes were rinsed with acetone, which was combined with the reaction mixture in a flask. The reaction mixture was subsequently concentrated under reduced pressure and the residue was purified by flash column chromatography on silica gel eluting with hexane/ethyl acetate (100:0 to 4:1 (v/v)) to afford the title compound **11** as a colorless liquid (81 mg, 0.23 mmol, 90%). The product was isolated as an inseparable 36:1 mixture of regioisomers.

NMR Spectroscopy:

^{1}H NMR (500 MHz, $CDCl_3$, δ): 7.42 – 7.34 (m, 5H), 4.54 (s, 1H), 4.10 (t, *J* = 6.3 Hz, 2H), 3.74 (s, 1H), 3.03 (s, 2H), 2.57 (tt, *J* = 10.8, 4.1 Hz, 1H), 2.19 – 2.03 (m, 2H), 2.04 – 1.52 (m, 8H).

^{13}C NMR (126 MHz, $CDCl_3$, δ): 174.2, 170.5, 136.1, 129.7, 128.6, 127.0 (q, *J* = 276.3 Hz), 126.9, 63.9, 47.0, 41.5, 41.2, 33.4 (q, *J* = 28.7 Hz), 28.6, 28.1, 27.7, 18.7 (q, *J* = 3.1 Hz).

^{19}**F NMR** (470 MHz, $CDCl_3$, δ): -66.39 (t, J = 10.8 Hz).

HRMS-ESI(m/z) calc'd for $C_{18}H_{22}F_3NO_3^+$ $[M+H]^+$, 358.1624; found, 358.1631; deviation: +1.8 ppm.

2-Methoxy-4-(4,4,4-trifluorobutyl)phenol (12)

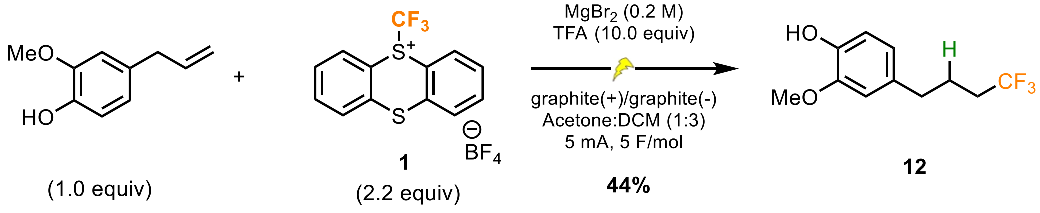

Under ambient conditions, to a 10 mL ElectraSyn vial equipped with a magnetic stir bar were added S-(Trifluoromethyl)thianthrenium tetrafluoroborate (0.20 g, 0.55 mmol, 2.2 equiv.) and magnesium bromide (0.37 g, 2.0 mmol, 8.0 equiv.). Subsequently, dichloromethane (7.5 mL), acetone (2.5 mL) (3:1) (c = 0.025 M), eugenol (39 µL, 0.25 mmol, 1.0 equiv.), and trifluoroacetic acid (0.19 mL, 2.5 mmol, 10 equiv.) were added at 25 °C. The ElectraSyn vial was equipped with two electrodes: graphite (+), graphite (-) and sealed with an ElectraSyn septum-cap. The reaction mixture was electrolyzed (electrolysis parameters: constant current: 5 mA, amount of charge: 5 F/mol and stirring: 1500 rpm). After reaction completion, the ElectraSyn vial cap was removed and the electrodes were rinsed with acetone, which was combined with the reaction mixture in a flask. The yield (58%) of **12** was determined by ^{19}F NMR spectroscopy using trifluorotoluene as internal standard. Saturated sodium bicarbonate solution (10 mL) was added to the flask containing the reaction mixture and the reaction crude was stirred for 10 min. Then, the aqueous phase was washed with dichloromethane (3 × 10 mL), dried over magnesium sulfate and concentrated *in vacuo*. The residue was purified by flash column chromatography on silica gel eluting with hexane/ethyl acetate (100:0 to 99:1 (v/v)) to afford the title compound **12** as a colorless oil (26 mg, 0.11 mmol, 44%). The product was isolated as an inseparable >40:1 mixture of regioisomers.

NMR Spectroscopy:

^{1}**H NMR** (500 MHz, $CDCl_3$, δ): 6.85 (d, J = 8.4 Hz, 1H), 6.67 (dd, J = 4.2, 2.4 Hz, 2H), 5.50 (s, 1H), 3.89 (s, 3H), 2.62 (t, J = 7.6 Hz, 2H), 2.16 – 2.02 (m, 2H), 1.87 (p, J = 7.7 Hz, 2H).

^{13}**C NMR** (126 MHz, $CDCl_3$, δ): 146.7, 144.2, 132.7, 127.4 (q, J = 276.4 Hz), 121.1, 114.5, 110.9, 56.0 (d, J = 2.0 Hz), 34.5, 33.2 (q, J = 28.5 Hz), 23.9 (d, J = 2.7 Hz).

^{19}**F NMR** (470 MHz, CD_3CN, δ): -66.84 (t, J = 11.3 Hz).

2-(2H-Benzo[d][1,2,3]triazol-2-yl)-4-methyl-6-(4,4,4-trifluorobutyl)phenol (13)

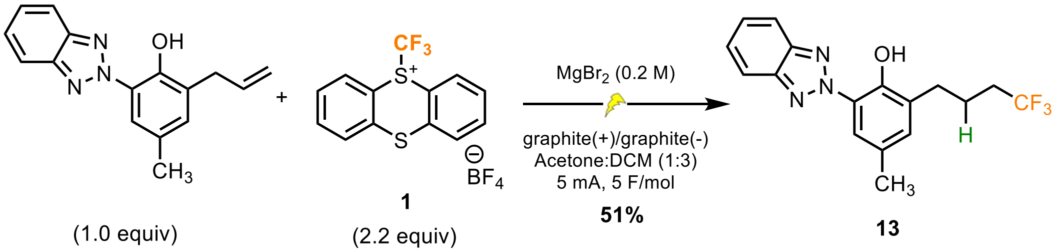

Under ambient conditions, to a 10 mL ElectraSyn vial equipped with a magnetic stir bar were added 2-(3-allyl-2-hydroxy-5-methylphenyl)-2*H*-benzotriazole (66.4 mg, 0.250 mmol, 1.00 equiv.), S-(Trifluoromethyl)thianthrenium tetrafluoroborate (0.21 g, 0.56 mmol, 2.2 equiv.) and magnesium bromide (0.37 g, 2.0 mmol, 8.0 equiv.). Subsequently, dichloromethane (7.5 mL) and acetone (2.5 mL) (3:1) (c = 0.025 M), and trifluoroacetic acid (0.19 mL, 2.5 mmol, 10 equiv.) were added at 25 °C. The ElectraSyn vial was equipped with two electrodes: graphite (+), graphite (-) and sealed with an ElectraSyn septum-cap. The reaction mixture was electrolyzed (electrolysis parameters: constant current: 5 mA, amount of charge: 5 F/mol and stirring: 1500 rpm). After reaction completion, the ElectraSyn vial cap was removed and the electrodes were rinsed with acetone, which was combined with the reaction mixture in a round bottom flask. The reaction mixture was subsequently concentrated under reduced pressure and the residue was purified by flash column chromatography on silica gel eluting with hexane/ethyl acetate (100:0 to 95:5 (v/v)) to afford the title compound **13** as a white solid (43 mg, 0.13 mmol, 51%). The product was isolated as an inseparable 30:1 mixture of regioisomers.

NMR Spectroscopy:

^{1}H NMR (500 MHz, $CDCl_3$, δ): 11.38 (s, 1H), 8.11 (d, *J* = 2.1 Hz, 1H), 7.94 (dd, *J* = 6.5, 3.1 Hz, 2H), 7.49 (dd, *J* = 6.6, 3.1 Hz, 2H), 7.03 (d, *J* = 2.2 Hz, 1H), 2.83 (t, *J* = 7.6 Hz, 2H), 2.39 (s, 3H), 2.29 – 2.12 (m, 2H), 1.98 (p, *J* = 7.8 Hz, 2H).

^{13}C NMR (126 MHz, $CDCl_3$, δ): 145.8, 143.0, 131.9, 130.6, 129.2, 127.8, 127.4 (d, *J* = 276.3 Hz), 125.0, 119.7, 117.8, 33.5 (q, *J* = 28.4 Hz), 29.5, 22.1 (d, *J* = 2.8 Hz), 20.7.

^{19}F NMR (470 MHz, $CDCl_3$, δ): -66.16 (t, *J* = 11.2 Hz).

HRMS-ESI(m/z) calc'd for $C_{17}H_{16}N_3OF_3^+$ $[M+H]^+$, 336.1318; found, 336.1325; deviation: +2.4 ppm.

Ethyl 4-((6,6,6-trifluorohexanoyl)oxy)benzoate (14)

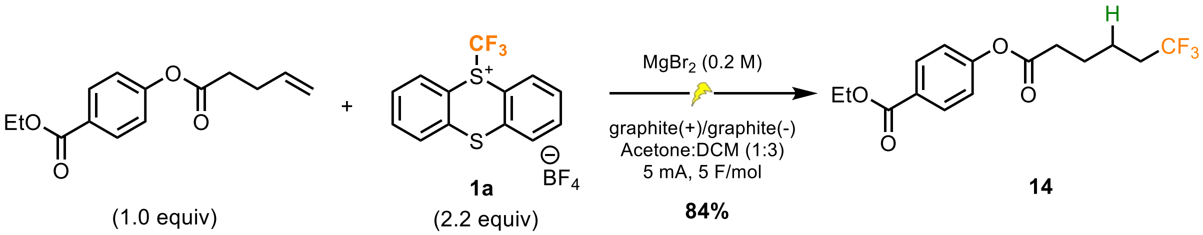

Under ambient conditions, to a 10 mL ElectraSyn vial equipped with a magnetic stir bar were added ethyl 4-(pent-4-enoyloxy)benzoate (62.1 mg, 0.250 mmol, 1.00 equiv.), S-(Trifluoromethyl)thianthrenium tetrafluoroborate (0.21 g, 0.56 mmol, 2.2 equiv.) and magnesium bromide (0.37 g, 2.0 mmol, 8.0 equiv.). Subsequently, dichloromethane (7.5 mL) and acetone (2.5 mL) (3:1) (c = 0.025 M) were added at 25 °C. The ElectraSyn vial was equipped with two electrodes: graphite (+), graphite (-) and sealed with an ElectraSyn septum-cap. The reaction mixture was electrolyzed (electrolysis parameters: constant current: 5 mA, amount of charge: 5 F/mol and stirring: 1500 rpm). After reaction completion, the ElectraSyn vial cap was removed and the electrodes were rinsed with acetone, which was combined with the reaction mixture in a round bottom flask. The reaction mixture was subsequently concentrated under reduced pressure and the residue was purified by flash column chromatography on silica gel eluting with hexane/ethyl acetate (100:0 to 95:5 (v/v)) to afford the title compound **14** as a colorless oil (67 mg, 0.21 mmol, 84%). The product was isolated as an inseparable >30:1 mixture of regioisomers.

NMR Spectroscopy:

^{1}H NMR (500 MHz, $CDCl_3$, δ): 8.07 (d, *J* = 8.7 Hz, 2H), 7.15 (d, *J* = 8.7 Hz, 2H), 4.37 (q, *J* = 7.2 Hz, 2H), 2.61 (t, *J* = 7.3 Hz, 2H), 2.22 – 2.04 (m, 2H), 1.83 (p, *J* = 7.4 Hz, 2H), 1.72 – 1.59 (m, 2H), 1.38 (t, *J* = 7.2 Hz, 3H).

^{13}C NMR (126 MHz, $CDCl_3$, δ): 171.0, 165.9, 154.2, 131.2, 128.2, 127.1 (q, *J* = 276.3 Hz), 121.6, 61.2, 33.9, 33.6 (q, *J* = 28.6 Hz), 23.9, 21.5 (q, *J* = 3.0 Hz), 14.4.

^{19}F NMR (470 MHz, $CDCl_3$, δ): -66.34 (t, *J* = 10.8 Hz).

HRMS-ESI(m/z) calc'd for $C_{15}H_{17}O_4F_3Na^+$[M+Na]$^+$, 341.0971; found, 341.0976; deviation: +1.3 ppm.

(Trifluoromethyl)cyclooctane (15)

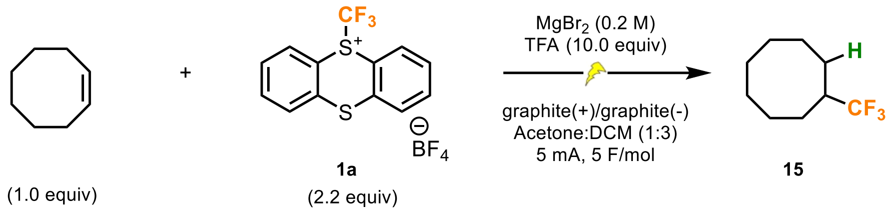

Under ambient conditions, to a 10 mL ElectraSyn vial equipped with a magnetic stir bar were added, S-(Trifluoromethyl)thianthrenium tetrafluoroborate (0.20 g, 0.55 mmol, 2.2 equiv.) and magnesium bromide (0.37 g, 2.0 mmol, 8.0 equiv.). Subsequently, dichloromethane (7.5 mL) and acetone (2.5 mL) (3:1) (c = 0.025 M), cyclooctene (33 µL, 0.25 mmol, 1.0 equiv.), and trifluoroacetic acid (0.19 mL, 2.5 mmol, 10 equiv.) were added at 25 °C. The ElectraSyn vial was equipped with two electrodes: graphite (+), graphite (-) and sealed with an ElectraSyn septum-cap. The reaction mixture was electrolyzed (electrolysis parameters: constant current: 5 mA, amount of charge: 5 F/mol and stirring: 1500 rpm). After reaction completion, the yield (55%) of **15** was determined by ^{1}H NMR spectroscopy using mesitylene as internal standard. The yield (56%) of **15** was also determined by ^{19}F NMR spectroscopy using trifluorotoluene as internal standard at -73.43 (d, *J* = 10.1 Hz).

Note: The compound is extremely volatile and only ^{19}F- and ^{1}H- NMR yields were reported.

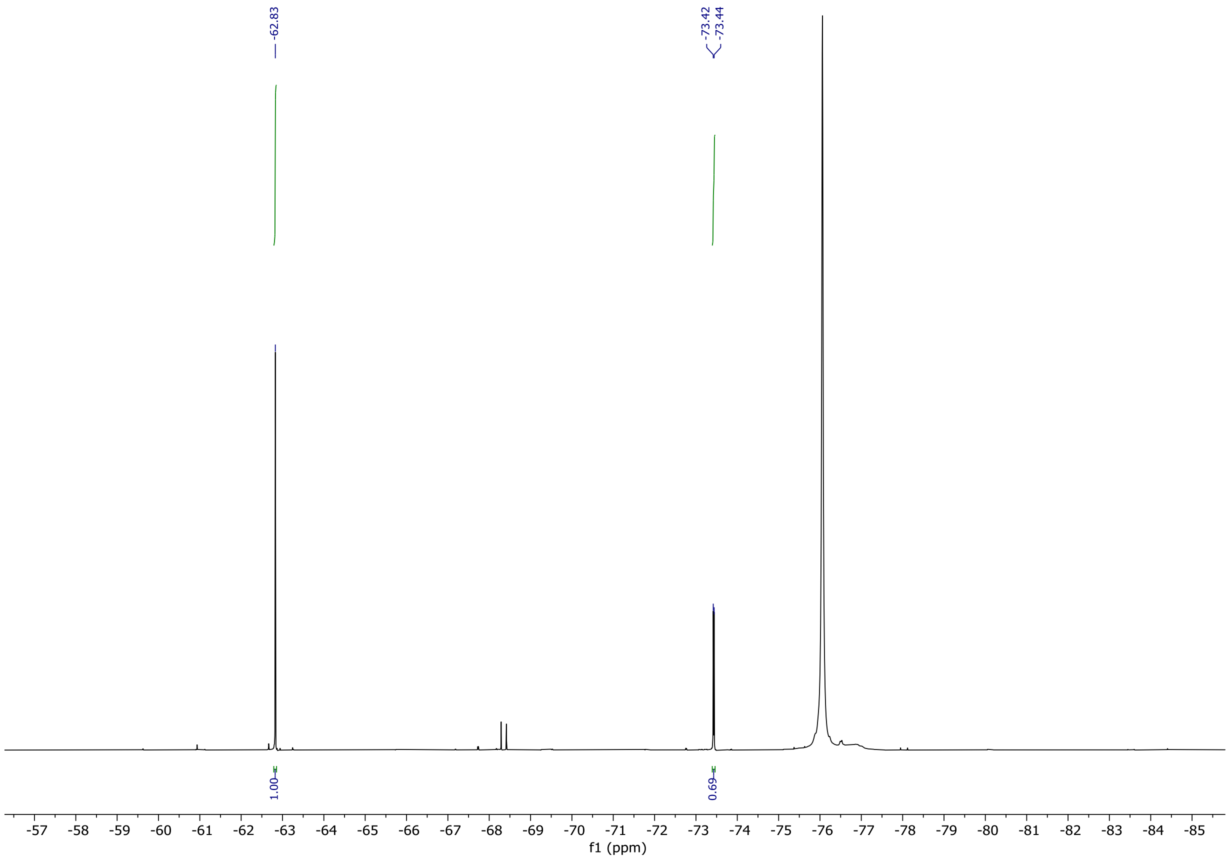

Figure S6: Quantitative ^{19}F -NMR of reaction mixture of electrochemical hydrotrifluoromethylation of cis-cyclooctene using trifluorotoluene as internal standard ($CDCl_3$, RT).

Note: Alternative internal olefins (cycloheptene and cyclohexene) were explored under the standard reaction conditions and the yields drop dramatically showing a potential limitation of this protocol for a general hydrotrifluoromethylation of internal olefins. The yields of volatile hydrotrifluoromethylation products are reported based on ^{19}F NMR integration of reaction mixtures with internal standard Ph–CF_3.

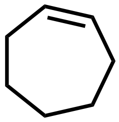

17% (^{19}F-NMR)

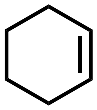

22% (^{19}F-NMR)

1-(Trifluoromethyl)-3-(3,3,3-trifluoropropyl)benzene (16)

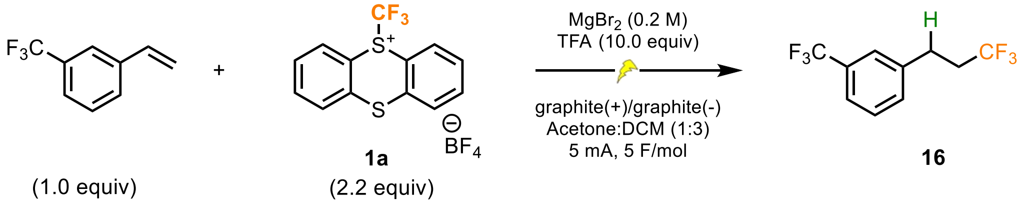

Under ambient conditions, to a 10 mL ElectraSyn vial equipped with a magnetic stir bar were added S-(Trifluoromethyl)thianthrenium tetrafluoroborate (0.20 g, 0.55 mmol, 2.2 equiv.) and magnesium bromide (0.37 g, 2.0 mmol, 8.0 equiv.). Subsequently, dichloromethane (7.5 mL) and acetone (2.5 mL) (3:1) (c = 0.025 M), 3-(trifluoromethyl)styrene (37 µL, 0.25 mmol, 1.0 equiv.), and trifluoroacetic acid (0.19 mL, 2.5 mmol, 10.0 equiv.) were added at 25 °C. The ElectraSyn vial was equipped with two electrodes: graphite (+), graphite (-) and sealed with an ElectraSyn septum-cap. The reaction mixture was electrolyzed (electrolysis parameters: constant current: 5 mA, amount of charge: 5 F/mol and stirring: 1500 rpm). After reaction completion, the yield (36%) of **16** was determined by ^{1}H NMR spectroscopy using mesitylene as internal standard.

Note: The compound is extremely volatile and only ^{1}H- NMR yield was reported.

4-(3,3,3-Trifluoropropyl)pyridine (17)

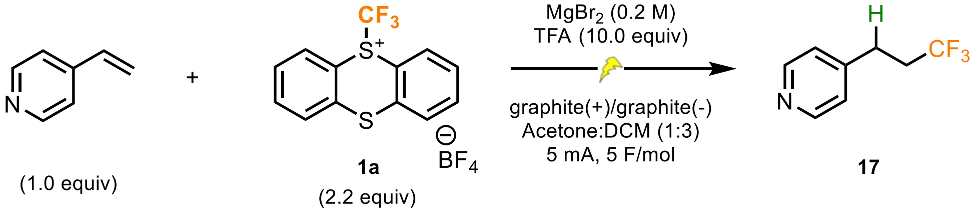

Under ambient conditions, to a 10 mL ElectraSyn vial equipped with a magnetic stir bar were added S-(Trifluoromethyl)thianthrenium tetrafluoroborate (0.20 g, 0.55 mmol, 2.2 equiv.) and magnesium bromide (0.37 g, 2.0 mmol, 8.0 equiv.). Subsequently, dichloromethane (7.5 mL), acetone (2.5 mL) (3:1) (c = 0.025 M), 4-vinylpyridine (27 µL, 0.25 mmol, 1.0 equiv.), and trifluoroacetic acid (0.19 mL, 2.5 mmol, 10 equiv.) were added at 25 °C. The ElectraSyn vial was equipped with two electrodes: graphite and graphite and sealed with an ElectraSyn septum-cap. The reaction mixture was electrolyzed (electrolysis parameters: constant current: 5 mA, amount of charge: 5 F/mol and stirring: 1500 rpm). After reaction completion, the yield (67%) of **17** was determined by ^{19}F NMR spectroscopy using trifluorotoluene as internal standard.

Note: The compound is extremely volatile and only ^{19}F-NMR yield was reported.

Febuxostat hydrotrifluoromethylated derivative (18)

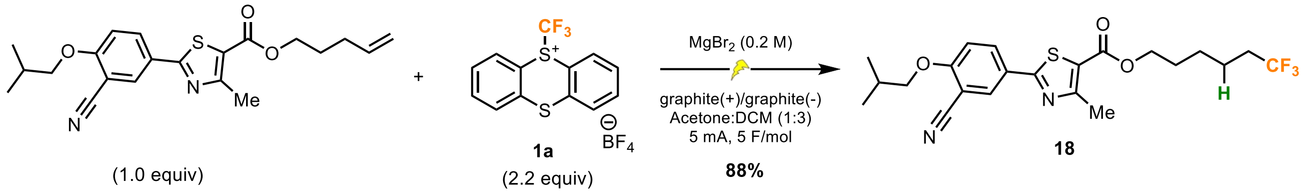

Under ambient conditions, to a 10 mL ElectraSyn vial equipped with a magnetic stir bar were added febuxostat derived olefin (96.1 mg, 0.250 mmol, 1.00 equiv.), S-(Trifluoromethyl)thianthrenium tetrafluoroborate (0.20 g, 0.54 mmol, 2.2 equiv.) and magnesium bromide (0.37 g, 2.0 mmol, 8.0 equiv.). Subsequently, dichloromethane (7.5 mL) and acetone (2.5 mL) (3:1) (c = 0.025 M) were added at 25 °C. The ElectraSyn vial was equipped with two electrodes: graphite (+), graphite (-) and sealed with an ElectraSyn septum-cap. The reaction mixture was electrolyzed (electrolysis parameters: constant current: 5 mA, amount of charge: 5 F/mol and stirring: 1500 rpm). After reaction completion, the ElectraSyn vial cap was removed and the electrodes were rinsed with acetone, which was combined with the reaction mixture in a flask. The reaction mixture was subsequently concentrated under reduced pressure and the residue was purified by flash column chromatography on silica gel eluting with hexane/ethyl acetate (100:0 to 4:1 (v/v)) to afford the title compound **18** as a white solid (99.6 mg, 0.219 mmol, 88%). The product was isolated as an inseparable 33:1 mixture of regioisomers.

NMR Spectroscopy:

^{1}H NMR (500 MHz, $CDCl_3$, δ): 8.15 (d, *J* = 2.2 Hz, 1H), 8.07 (dd, *J* = 8.8, 2.3 Hz, 1H), 7.00 (d, *J* = 8.9 Hz, 1H), 4.29 (t, *J* = 6.5 Hz, 2H), 3.88 (d, *J* = 6.4 Hz, 2H), 2.74 (s, 3H), 2.23 – 2.16 (m, 1H), 2.15 – 2.03 (m, 2H), 1.81 – 1.73 (m, 2H), 1.63 (p, *J* = 7.6 Hz, 2H), 1.51 (q, *J* = 8.3 Hz, 2H), 1.07 (d, *J* = 6.7 Hz, 6H).

^{13}C NMR (126 MHz, $CDCl_3$, δ): 167.4, 162.7, 162.2, 161.4, 132.7, 132.3, 129.4 (d, *J* = 276.3 Hz), 126.1, 121.8, 115.5, 112.8, 103.2, 75.9, 65.1, 33.8 (q, *J* = 28.5 Hz), 28.5, 28.3, 25.4, 21.8 (t, *J* = 3.0 Hz), 19.2, 17.6.

^{19}F NMR (470 MHz, CD_3CN, δ): -66.92 (t, *J* = 11.3 Hz).

HRMS-ESI(m/z) calc'd for $C_{22}H_{25}F_3N_2O_3S^+$ $[M+H]^+$, 455.1618; found, 455.1611; deviation: +1.5 ppm.

Quinine hydrotrifluoromethylated derivative (19)

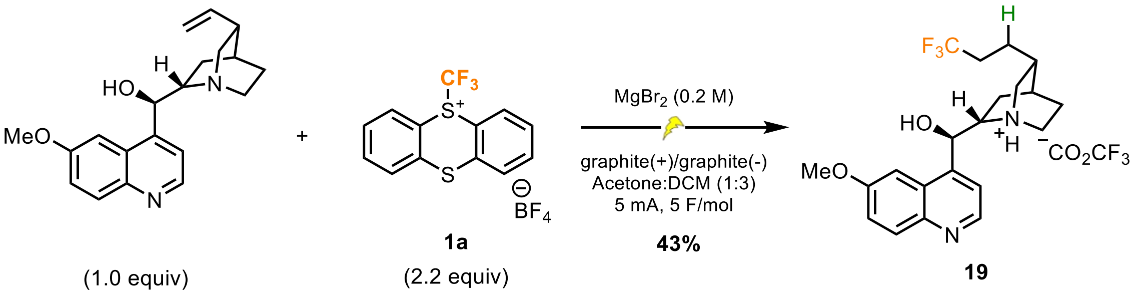

Under ambient conditions, to a 10 mL ElectraSyn vial equipped with a magnetic stir bar were added quinine

(81.1 mg, 0.250 mmol, 1.00 equiv.), S-(Trifluoromethyl)thianthrenium tetrafluoroborate (0.20 g, 0.55 mmol, 2.2 equiv.) and magnesium bromide (0.37 g, 2.0 mmol, 8.0 equiv.). Subsequently, dichloromethane (7.5 mL), acetone (2.5 mL) (3:1) (c = 0.025 M), and trifluoroacetic acid (0.19 mL, 2.5 mmol, 10 equiv.) were added at 25 °C. The ElectraSyn vial was equipped with two electrodes: graphite (+), graphite (-) and sealed with an ElectraSyn septum-cap. The reaction mixture was electrolyzed (electrolysis parameters: constant current: 5 mA, amount of charge: 5 F/mol and stirring: 1500 rpm). After reaction completion, the ElectraSyn vial cap was removed and the electrodes were rinsed with acetone, which was combined with the reaction mixture in a flask. Saturated sodium bicarbonate solution (10 mL) was added to the flask containing the reaction mixture and the reaction crude was stirred for 10 min. Then, the aqueous phase was washed with dichloromethane (3 × 10 mL), dried over magnesium sulfate and concentrated *in vacuo*. The residue was purified by flash column chromatography on silica gel eluting with methanol/ethyl acetate (4:96 (v/v)) to afford the title compound **19** as a colorless solid (53 mg, 0.11 mmol, 43%).

NMR Spectroscopy:

^{1}H NMR (500 MHz, $CDCl_3$, δ): 8.62 (d, *J* = 4.4 Hz, 1H), 7.71 (d, *J* = 9.2 Hz, 1H), 7.60 (d, *J* = 4.5 Hz, 1H), 7.05 (dd, *J* = 9.3, 2.5 Hz, 1H), 6.85 (d, *J* = 2.7 Hz, 1H), 6.25 (s, 1H), 4.32 (t, *J* = 12.3 Hz, 1H), 3.67 (s, 3H), 3.48 (dd, *J* = 13.3, 10.6 Hz, 1H), 3.24 (dd, *J* = 10.5, 7.3 Hz, 1H), 3.02 (td, *J* = 11.7, 5.8 Hz, 1H), 2.59 (ddd, *J* = 13.6, 6.0, 2.5 Hz, 1H), 2.30 – 2.10 (m, 2H), 1.96 (qd, *J* = 8.2, 3.4 Hz, 4H), 1.78 – 1.65 (m, 1H), 1.45 (q, *J* = 7.8 Hz, 2H), 1.29 – 1.21 (m, 2H).

^{13}C NMR (126 MHz, $CDCl_3$, δ): 163.2 (q, *J* = 35.2 Hz), 158.4, 147.1, 144.4, 143.8, 131.5, 128.7 (q, *J* = 276.3 Hz), 125.4, 122.3, 118.7, 116.8 (d, *J* = 292.7 Hz), 99.6, 66.5, 60.3, 56.5, 56.4, 43.9, 33.0, 31.4 (q, *J* = 29.2 Hz), 26.1 (d, *J* = 2.9 Hz), 25.0 (d, *J* = 7.3 Hz), 18.0.

^{19}F NMR (470 MHz, CD_3CN, δ): -66.22 (t, *J* = 10.5 Hz), -75.29.

Vinclozolin hydrotrifluoromethylated derivative (20)

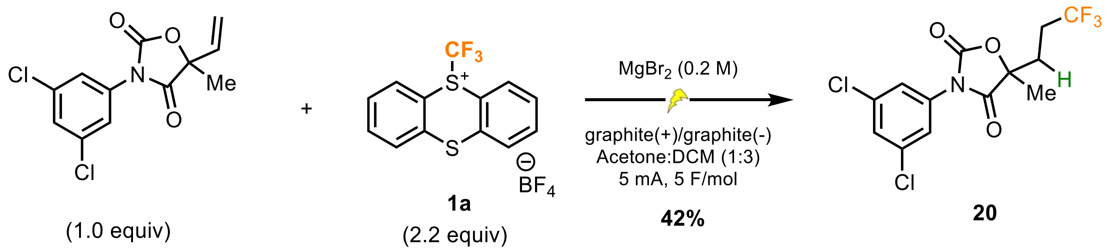

Under ambient conditions, to a 10 mL ElectraSyn vial equipped with a magnetic stir bar were added vinclozolin (71.5 mg, 0.250 mmol, 1.00 equiv.), S-(Trifluoromethyl)thianthrenium tetrafluoroborate (0.20 g, 0.55 mmol, 2.2 equiv.) and magnesium bromide (0.37 g, 2.0 mmol, 8.0 equiv.). Subsequently, dichloromethane (7.5 mL) and acetone (2.5 mL) (3:1) (c = 0.025 M) were added at 25 °C. The ElectraSyn vial was equipped with two electrodes: graphite (+), graphite (-) and sealed with an ElectraSyn septum-cap. The reaction mixture was electrolyzed (electrolysis parameters: constant current: 5 mA, amount of charge: 5 F/mol and stirring: 1500

rpm). After reaction completion, the ElectraSyn vial cap was removed and the electrodes were rinsed with acetone, which was combined with the reaction mixture in a flask. The yield (54%) of **20** was determined by ^{19}F NMR spectroscopy using trifluorotoluene as internal standard. The reaction mixture was subsequently concentrated under reduced pressure and the residue was purified by flash column chromatography on silica gel eluting with hexane/ethyl acetate (100:0 (v/v)) to afford the title compound **20** as a colorless liquid (36 mg, 0.11 mmol, 42%).

NMR Spectroscopy:

^{1}H NMR (500 MHz, $CDCl_3$, δ): 7.46 – 7.42 (m, 3H), 2.40 – 2.27 (m, 1H), 2.28 – 2.13 (m, 3H), 1.71 (s, 3H).

^{13}C NMR (126 MHz, $CDCl_3$, δ): 172.8, 151.9, 135.8, 132.4, 129.4, 126.2 (q, J = 276.2 Hz), 123.8, 84.2, 29.5 (q, J = 3.2 Hz), 28.4 (q, J = 30.4 Hz), 22.4.

^{19}F NMR (470 MHz, $CDCl_3$, δ): -66.40 (t, J = 9.6 Hz).

Theobromine hydrotrifluoromethylated derivative (21)

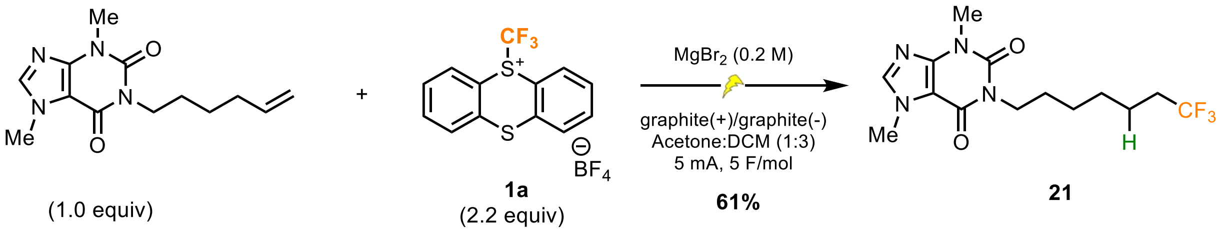

Under ambient conditions, to a 10 mL ElectraSyn vial equipped with a magnetic stir bar were added theobromine derived olefin (65.6 mg, 0.250 mmol, 1.00 equiv.), S-(Trifluoromethyl)thianthrenium tetrafluoroborate (0.20 g, 0.54 mmol, 2.2 equiv.) and magnesium bromide (0.37 g, 2.0 mmol, 8.0 equiv.). Subsequently, dichloromethane (7.5 mL) and acetone (2.5 mL) (3:1) (c = 0.025 M) were added at 25 °C. The ElectraSyn vial was equipped with two electrodes: graphite (+), graphite (-) and sealed with an ElectraSyn septum-cap. The reaction mixture was electrolyzed (electrolysis parameters: constant current: 5 mA, amount of charge: 5 F/mol and stirring: 1500 rpm). After reaction completion, the ElectraSyn vial cap was removed and the electrodes were rinsed with acetone, which was combined with the reaction mixture in a flask. The reaction mixture was subsequently concentrated under reduced pressure and the residue was purified by flash column chromatography on silica gel eluting with hexane/ethyl acetate (100:0 to 0:100 (v/v)) to afford the title compound **21** as a white solid (51 mg, 0.15 mmol, 61%). The product was isolated as an inseparable 50:1 mixture of regioisomers.

NMR Spectroscopy:

^{1}H NMR (500 MHz, $CDCl_3$, δ): 7.50 (s, 1H), 4.05 – 3.96 (m, 5H), 3.57 (s, 3H), 2.17 – 1.97 (m, 2H), 1.73 – 1.62 (m, 2H), 1.60 – 1.52 (m, 2H), 1.45 – 1.36 (m, 4H).

^{13}C NMR (126 MHz, $CDCl_3$, δ): 155.5, 151.6, 148.9, 141.5, 127.4 (q, J = 276.3 Hz), 107.8, 41.3, 33.8 (q,

J = 28.3 Hz), 33.7, 29.8, 28.6, 27.9, 26.7, 21.9 (q, J = 2.9 Hz).

^{19}F NMR (470 MHz, $CDCl_3$, δ): -66.41 (t, J = 10.9 Hz).

HRMS-ESI(m/z) calc'd for $C_{14}H_{19}F_3N_4O_2^+$ $[M+H]^+$, 333.1533; found, 333.1535; deviation: +0.6 ppm.

Indomethacin hydrotrifluoromethylated derivative (22)

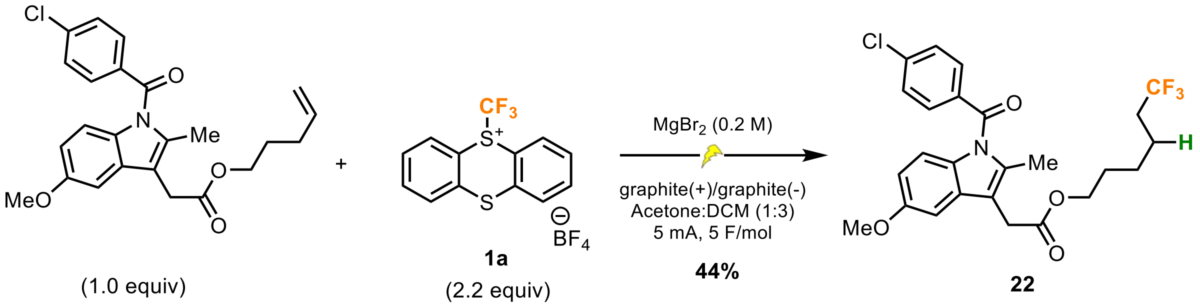

(1.0 equiv) **1a** (2.2 equiv) **22**

Under ambient conditions, to a 10 mL ElectraSyn vial equipped with a magnetic stir bar were added indomethacin ester derived olefin (0.11 g, 0.25 mmol, 1.0 equiv.), S-(Trifluoromethyl)thianthrenium tetrafluoroborate (0.20 g, 0.54 mmol, 2.2 equiv.) and magnesium bromide (0.37 g, 2.0 mmol, 8.0 equiv.). Subsequently, dichloromethane (7.5 mL) and acetone (2.5 mL) (3:1) (c = 0.025 M) were added at 25 °C. The ElectraSyn vial was equipped with two electrodes: graphite (+), graphite (-) and sealed with an ElectraSyn septum-cap. The reaction mixture was electrolyzed (electrolysis parameters: constant current: 5 mA, amount of charge: 5 F/mol and stirring: 1500 rpm). After reaction completion, the ElectraSyn vial cap was removed and the electrodes were rinsed with acetone, which was combined with the reaction mixture in a flask. The yield (58%) of **22** was determined by ^{19}F NMR spectroscopy using trifluorotoluene as internal standard. The reaction mixture was subsequently concentrated under reduced pressure and the residue was purified by flash column chromatography on silica gel eluting with hexane/ethyl acetate (100:0 to 4:1 (v/v)) to afford the title compound **22** as a colorless liquid (55 mg, 0.11 mmol, 44%). The product was isolated as an inseparable 33:1 mixture of regioisomers.

NMR Spectroscopy:

^{1}H NMR (500 MHz, CD_3CN, δ): 7.63 (d, J = 8.4 Hz, 2H), 7.54 (d, J = 8.5 Hz, 2H), 7.02 – 6.91 (m, 2H), 6.67 (dd, J = 9.0, 2.6 Hz, 1H), 4.07 (t, J = 6.5 Hz, 2H), 3.79 (s, 3H), 3.69 (s, 2H), 2.26 (s, 3H), 2.13 – 2.01 (m, 2H), 1.66 – 1.56 (m, 2H), 1.55 – 1.44 (m, 2H), 1.38 – 1.29 (m, 2H).

^{13}C NMR (126 MHz, $CDCl_3$, δ): 171.4, 168.9, 156.6, 139.0 (d, J = 1.7 Hz), 136.4, 135.0, 131.7, 131.4, 131.3, 129.6, 126.1 (d, J = 275.4 Hz), 115.6, 113.6, 112.0, 102.0, 64.8, 55.8, 33.4 (q, J = 27.9 Hz), 30.2, 28.5, 25.2, 21.8 (q, J = 3.1 Hz), 13.3.

^{19}F NMR (470 MHz, $CDCl_3$, δ): -66.94 (t, J = 11.3 Hz).

Probenecid hydrotrifluoromethylated derivative (23)

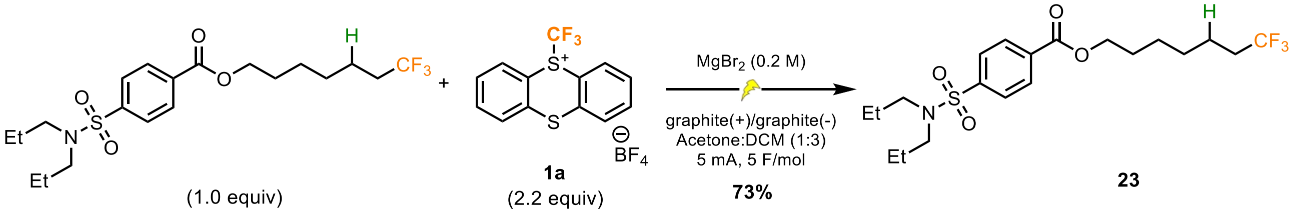

Under ambient conditions, to a 10 mL ElectraSyn vial equipped with a magnetic stir bar were added probenecid ester derived olefin (92.0 mg, 0.250 mmol, 1.00 equiv.), S-(Trifluoromethyl)thianthrenium tetrafluoroborate (0.21 g, 0.56 mmol, 2.2 equiv.) and magnesium bromide (0.36 g, 2.0 mmol, 8.0 equiv.). Subsequently, dichloromethane (7.5 mL) and acetone (2.5 mL) (3:1) (c = 0.025 M) were added at 25 °C. The ElectraSyn vial was equipped with two electrodes: graphite (+), graphite (-) and sealed with an ElectraSyn septum-cap. The reaction mixture was electrolyzed (electrolysis parameters: constant current: 5 mA, amount of charge: 5 F/mol and stirring: 1500 rpm). After reaction completion, the ElectraSyn vial cap was removed and the electrodes were rinsed with acetone, which was combined with the reaction mixture in a round bottom flask. The reaction mixture was subsequently concentrated under reduced pressure and the residue was purified by flash column chromatography on silica gel eluting with hexane/ethyl acetate (100:0 to 9:1 (v/v)) to afford the title compound **23** as a colorless oil (80 mg, 0.18 mmol, 73%). The product was isolated as an inseparable 30:1 mixture of regioisomers.

NMR Spectroscopy:

^{1}H NMR (500 MHz, $CDCl_3$, δ): 8.14 (d, *J* = 8.4 Hz, 2H), 7.87 (d, *J* = 8.4 Hz, 2H), 4.34 (t, *J* = 6.6 Hz, 2H), 3.09 (dd, *J* = 8.7, 6.6 Hz, 4H), 2.21 – 1.96 (m, 2H), 1.79 (p, *J* = 6.7 Hz, 2H), 1.65 – 1.32 (m, 10H), 0.86 (t, *J* = 7.4 Hz, 6H).

^{13}C NMR (126 MHz, $CDCl_3$, δ): 165.4, 144.4, 133.8, 130.3, 127.3 (q, *J* = 276.3 Hz), 127.1, 65.6, 50.1, 33.7 (q, *J* = 28.4 Hz), 28.5, 28.4, 25.8, 22.1, 21.9 (q, *J* = 2.9 Hz), 11.3.

^{19}F NMR (470 MHz, $CDCl_3$, δ): -66.37 (t, *J* = 10.9 Hz).

Optimization

Example of protocol for optimization

Under an ambient atmosphere, a 5 mL Electrasyn vial was equipped with a magnetic stir bar. The vial was charged with $TT^{+}CF_3BF_4^{-}$ (81 mg, 0.22 mmol, 2.2 equiv.) and magnesium bromide (0.15 mg, 0.8 mmol, 8.0 equiv.). Subsequently, dichloromethane (3.0 mL), acetone (1.0 mL) (3:1) (c = 0.025 M), and 4-allylanisole (15.0 µL, 0.10 mmol, 1.0 equiv.) were added at 25 °C. The Electrasyn vial was placed in the Electrasyn 2.0 with the following settings: 5.0 mA, 5 F/mol and maximum stirring (1500 rpm). After conclusion, mesitylene (10 µL, 0.072 mmol, 1.0 equiv.) was added as an internal standard and the reaction was diluted with 1 mL $CDCl_3$. The yield of hydrotrifluorinated product (δ 6.85 ppm) was determined by ^{1}H NMR integration relative to the internal

standard mesitylene (δ 6.80 (s) ppm).

Initial Experiments

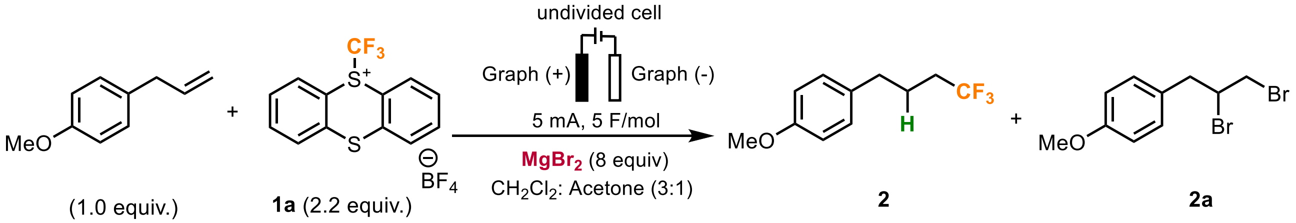

Entry	Deviation from standard conditions	Yield of 2 (%)	Yield of 2a (%)
1	None	62%	traces
2	No electricity	n.d	n.d
3	MeCN and NaCl (2.0 equiv.)	<5%	-
4	MeCN and $MgBr_2$ (0.2 M)	30%	70%
5	DCM	traces	n.d
6	Acetone	41%	n.d
7	No $MgBr_2$	traces	n.d
8	$TBABr_3$ (3.0 equiv)	60%	traces
9	DIPEA as a sacrifitial reductant	traces	-
10	Umemoto reagent instead of **1a**	42%	6%
11	$TT^+CF_3OTf^-$ instead of **1a**	n.d	71%
12	$TBABF_4$ (5.0 equiv) and $TT^+CF_3OTf^-$	69%	traces
13	MeCN and NaBr instead of $MgBr_2$	traces	detected
14	$ZnBr_2$ instead of $MgBr_2$	traces	n.d
15	$FeBr_2$ instead of $MgBr_2$	n.d	n.d
16	TBABr instead of $MgBr_2$	n.d	n.d
17	MgI_2 instead of $MgBr_2$	traces	-
18	TBABr instead of $MgBr_2$	traces	n.d
19	$Mg(OAc)_2$ instead of $MgBr_2$	traces	n.d

Evidence of dibromination product (Entry 4)

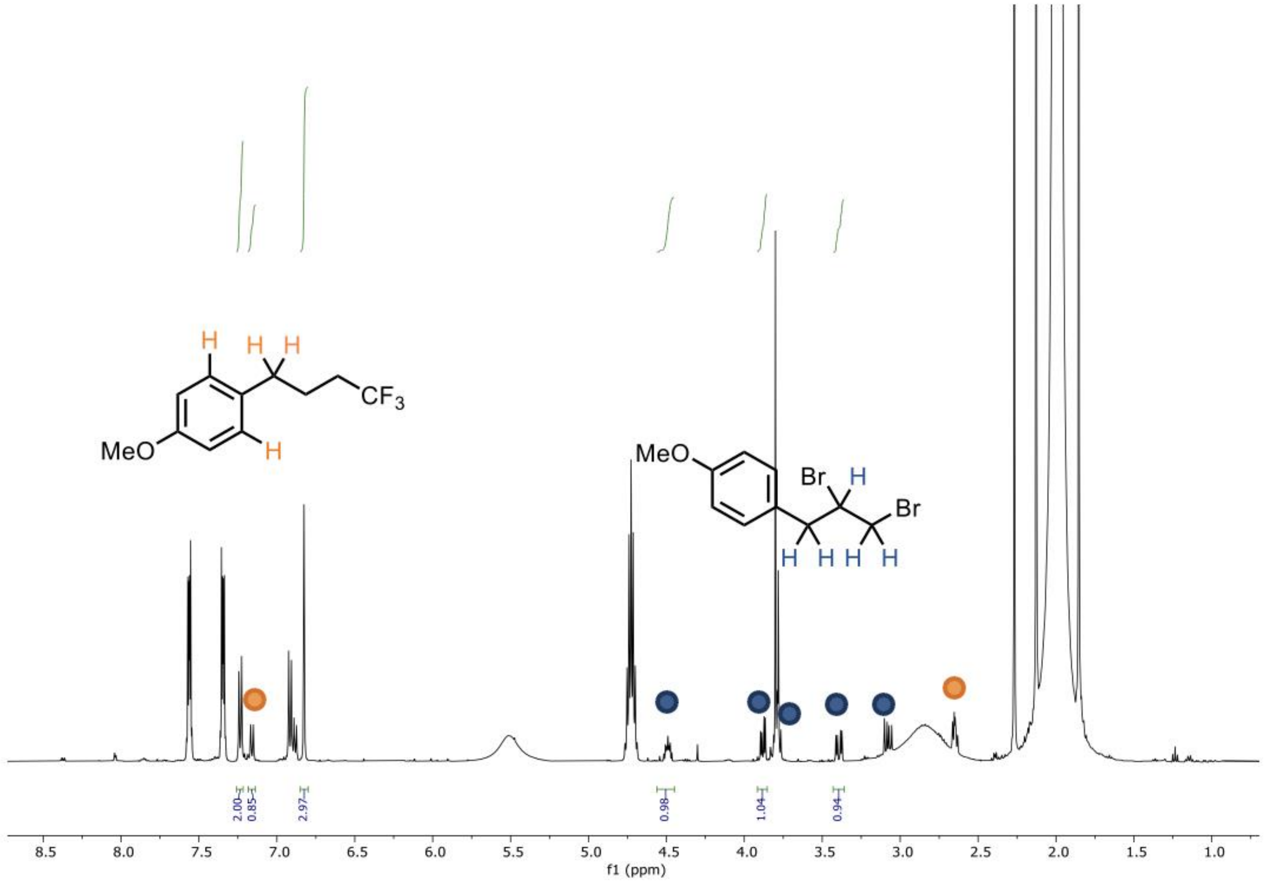

Figure S7: ^{1}H-NMR of the reaction mixture of electrochemical hydrotrifluoromethylation of 4-allylanisole (CD_3CN, RT).

Electrode Screening

Anode	Cathode	Hydrotrifluorinated product (%)	4-allylanisole (%)
Graphite	Graphite	62%	9
Zn	Graphite	Traces	n.d
Pt	Graphite	25%	66%
Graphite	Pt	Traces	93%
RVC	Graphite	45%	35%
Graphite	RVC	30%	41%
RVC	RVC	32%	39%
Mg	Graphite	Traces	n.d

Solvent Screening

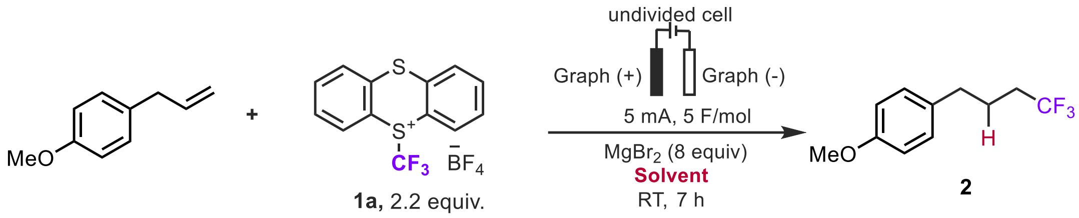

Entry	Solvent	yield of 2
1	Acetone: Alkyl halide (1:3)	<10%
2	DCM : Alkyl halide (3:1)	<10%
3	DCM : DMF (3:1)	messy, <5%
4	Acetone : DMF (1:3)	traces
5	Acetone: Ethyl acetate (1:3)	13%
6	Acetone: MeCN (1:3)	25%

Current Screening

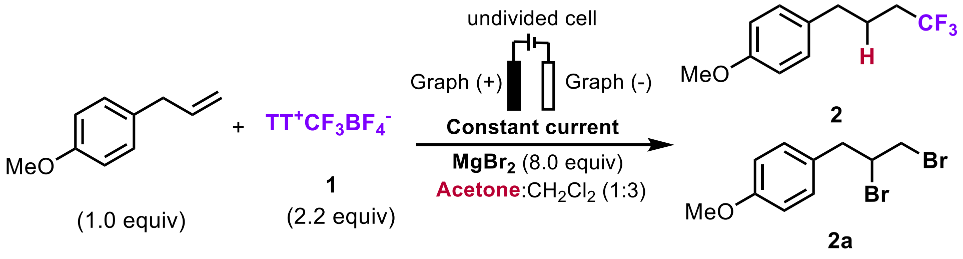

Entry	Current	Yield of 2 (%)	Yield of 2a (%)	Unreacted 4-allylanisole
1	1 mA	63%	traces	8%
2	5 mA	62%	traces	traces
3	10 mA	58%	traces	5%

Mechanistic investigations

Control experiments

A) Radical trapping experiment:

Under an ambient atmosphere, a 5 mL Electrasyn vial was equipped with a magnetic stir bar. The vial was charged with S-(Trifluoromethyl)thianthrenium tetrafluoroborate (81.0 mg, 0.22 mmol, 2.00 equiv.), magnesium bromide (0.15 g, 0.80 mmol, 8.0 equiv.) and TEMPO (16 mg, 0.11 mmol, 1.0 equiv.). Subsequently, dichloromethane (3.0 mL) and acetone (1.0 mL) (3:1) (c = 0.025 M), 4-allylanisole (0.015 mL, 0.10 mmol, 1.0 equiv.) were added at 25 °C. The ElectraSyn vial was equipped with two electrodes: graphite (+), graphite (-)

and sealed with an ElectraSyn septum-cap. The reaction mixture was electrolyzed (electrolysis parameters: constant current: 5 mA, amount of charge: 5 F/mol and stirring: 1500 rpm). After conclusion, mesitylene (8.6 mg, 10 µL, 0.07 mmol, 0.66 equiv.) and trifluorotoluene (12 mg, 10 µL, 0.070 mmol, 0.66 equiv.) were added as an internal standard. The mixture was diluted with $CDCl_3$, and a quantitative ^1H NMR and ^{19}F NMR spectrum was measured. The yields (40%) and (41%) of **2** were determined by ^1H and ^{19}F NMR spectroscopy, respectively. TEMPO-CF_3 adduct [^{19}F NMR (375 MHz, $CDCl_3$) δ -55.7][6] was not observed in the ^{19}F NMR spectrum.

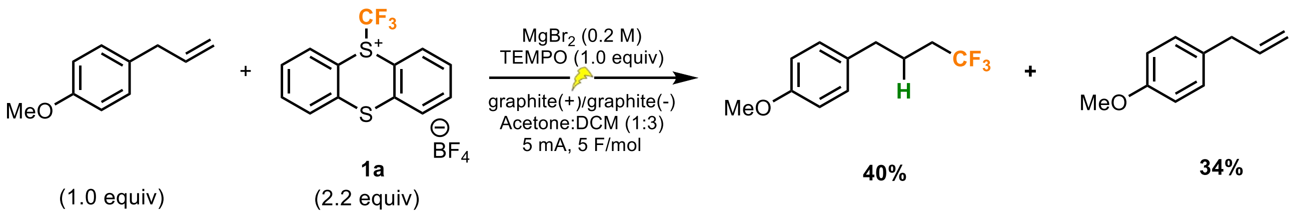

B) No $MgBr_2$

Under an ambient atmosphere, a 5 mL Electrasyn vial was equipped with a magnetic stir bar. The vial was charged with S-(Trifluoromethyl)thianthrenium tetrafluoroborate (81.0 mg, 0.220 mmol, 2.20 equiv.). Subsequently, dichloromethane (3.0 mL) and acetone (1.0 mL) (3:1) (c = 0.025 M), 4-allylanisole (0.015 mL, 0.10 mmol, 1.0 equiv.) were added at 25 °C. The ElectraSyn vial was equipped with two electrodes: graphite (+), graphite (-) and sealed with an ElectraSyn septum-cap. The reaction mixture was electrolyzed (electrolysis parameters: constant current: 5 mA, amount of charge: 5 F/mol and stirring: 1500 rpm). After conclusion, mesitylene (8.6 mg, 10 µL, 0.07 mmol, 0.66 equiv.) was added as an internal standard. The mixture was diluted with $CDCl_3$, and a quantitative ^1H NMR spectrum was measured. The yield (<1%) of **2** was determined by ^1H NMR spectroscopy.

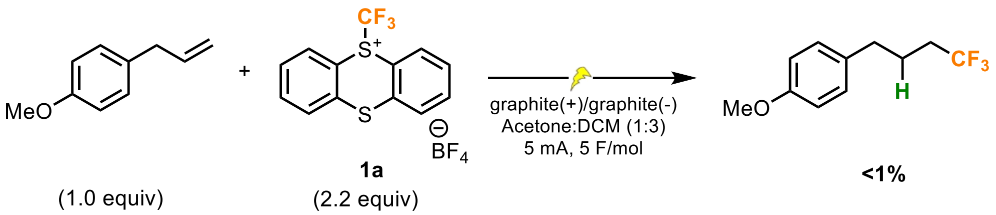

C) Dibromination of olefin: Use of $TBABr_3$ as Br_2 source in absence of electrochemistry

Under an ambient atmosphere, a 5 mL Electrasyn vial was equipped with a magnetic stir bar. The vial was charged with $TBABr_3$ (0.14 g, 0.30 mmol, 3.00 equiv.). Subsequently, dichloromethane (3.0 mL) and acetone (1.0 mL) (3:1) (c = 0.025 M), 4-allylanisole (0.015 mL, 0.10 mmol, 1.0 equiv.) were added at 25 °C. After 1 h, a qualitative ^1H NMR was prepared in $CDCl_3$ and 1:5 (dibromination: olefin) ratio was observed, which after a week did not show any notorious change in dibromination:olefin ratio.

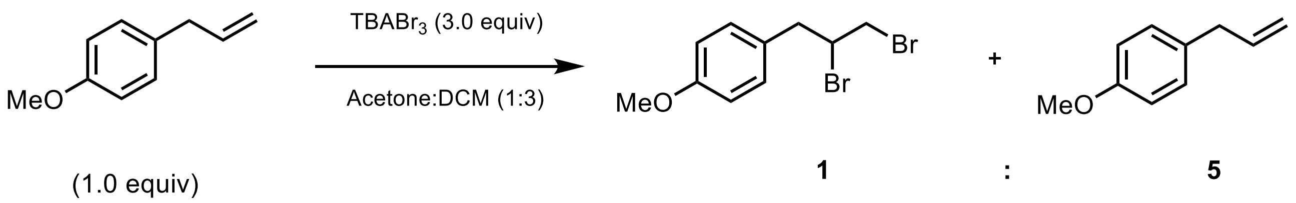

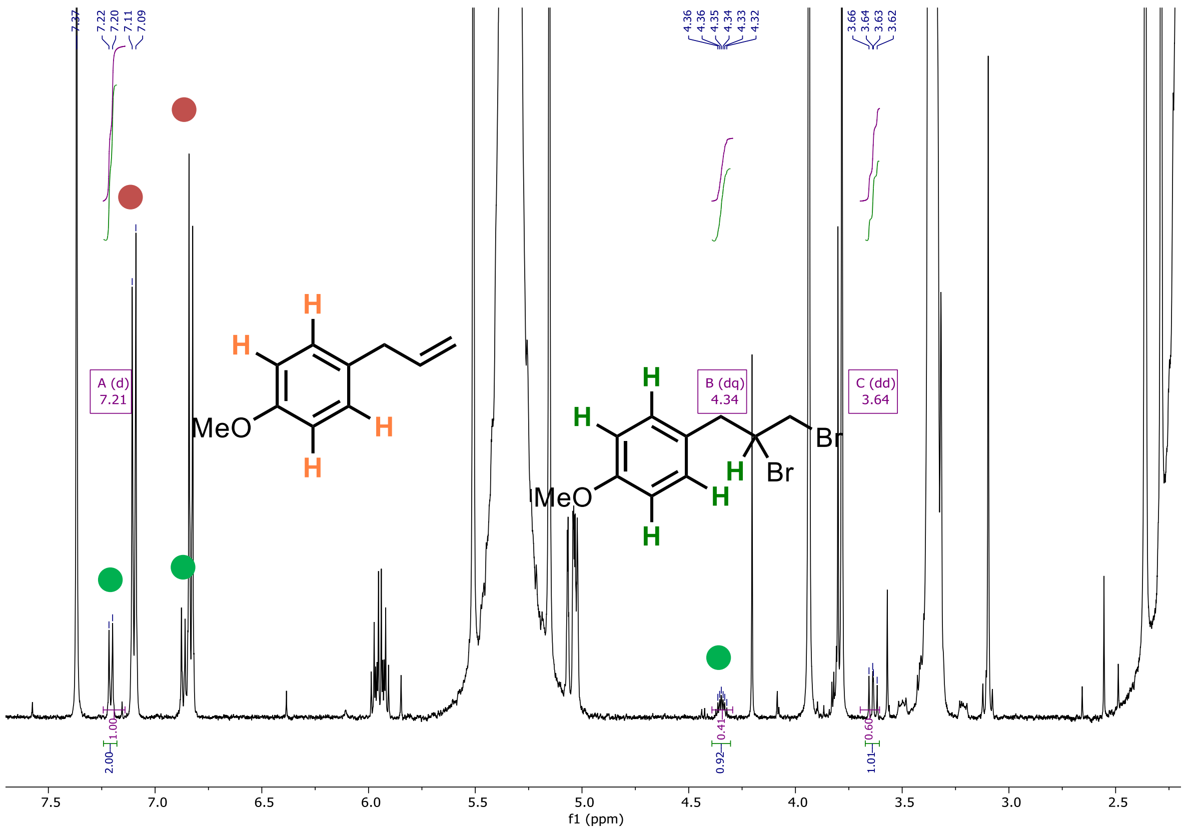

Figure S8: ^{1}H-NMR of the reaction mixture of non-electrochemical reaction of 4-allylanisole with $TBABr_3$ ($CDCl_3$, RT).

D) Evidence of bromoacetone

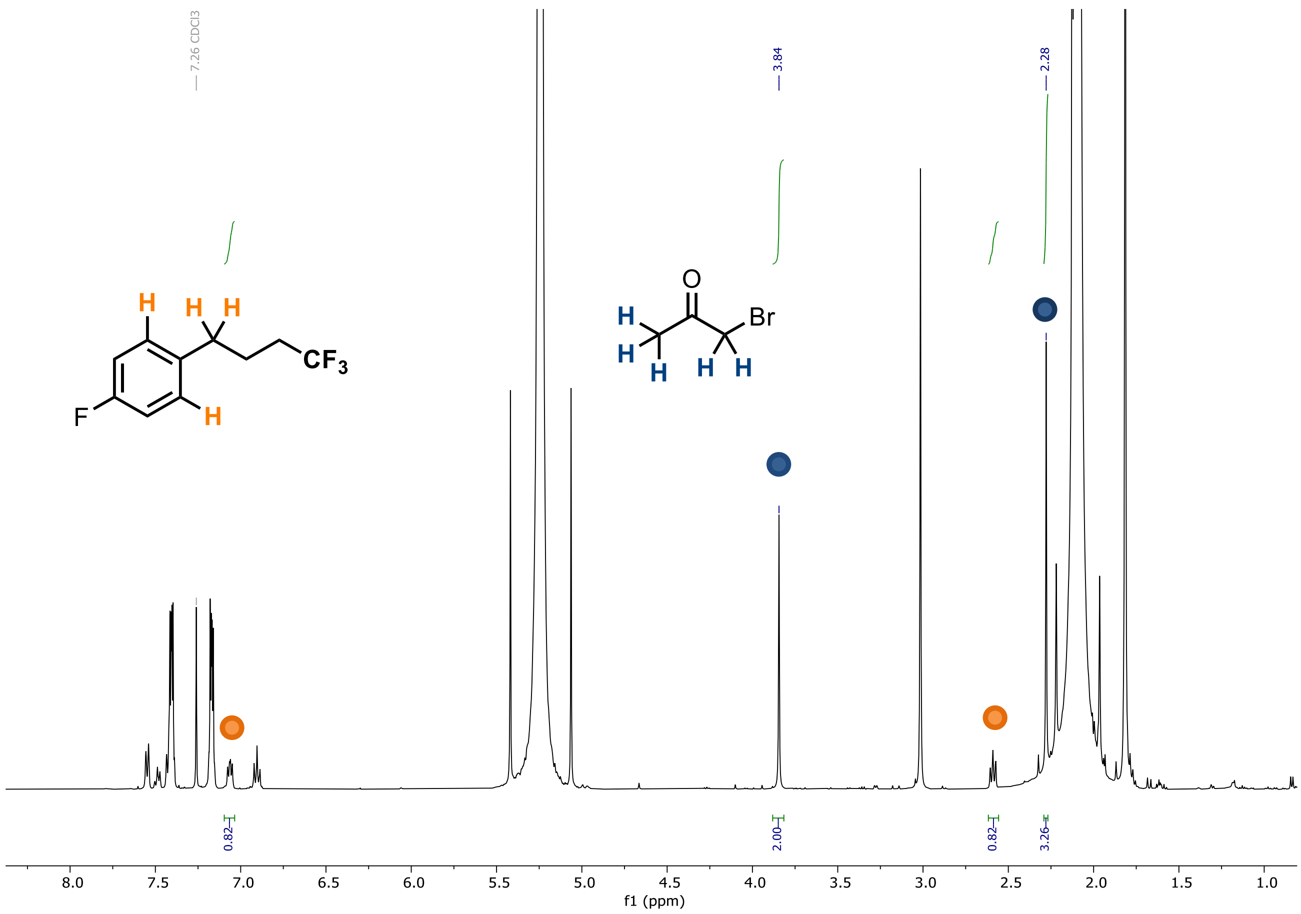

Figure S9: ^{1}H-NMR of the reaction mixture of electrochemical hydrotrifluoromethylation of 4-fluoroallylbenzene ($CDCl_3$, RT).

E) Evidence of homoaldol condensation product: 4-methyl-pent-3-en-2-one in reaction mixture.

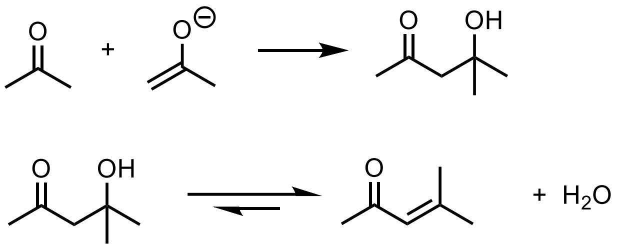

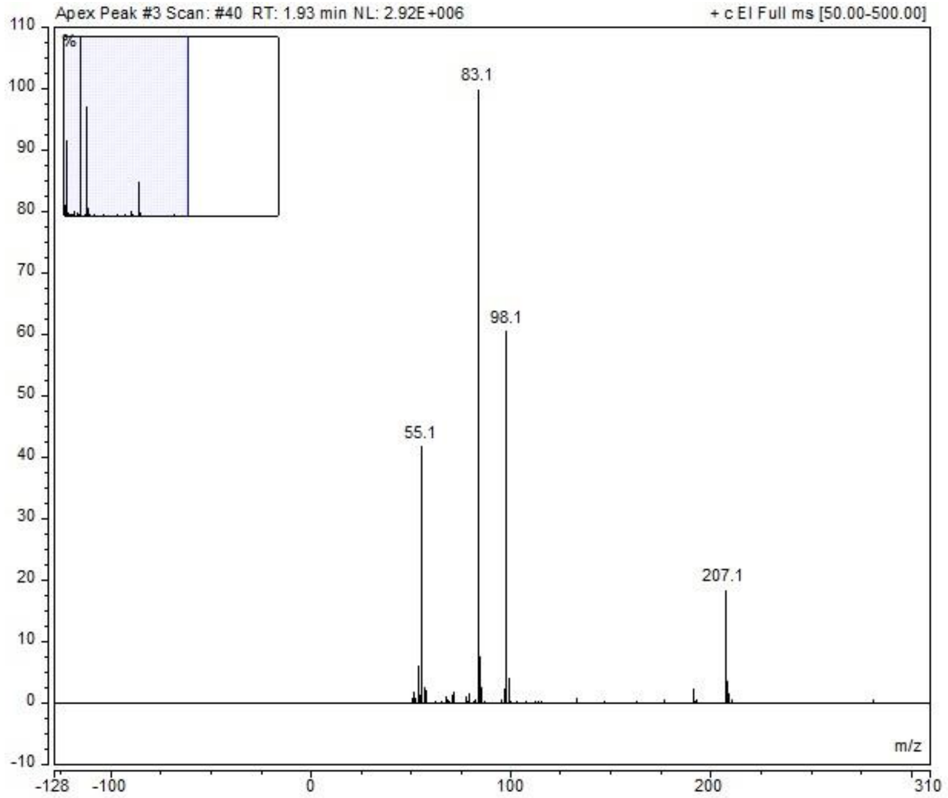

Figure S10: Mass spectrum of 4-methyl-pent-3-en-2-one.

F) Radical clock experiment

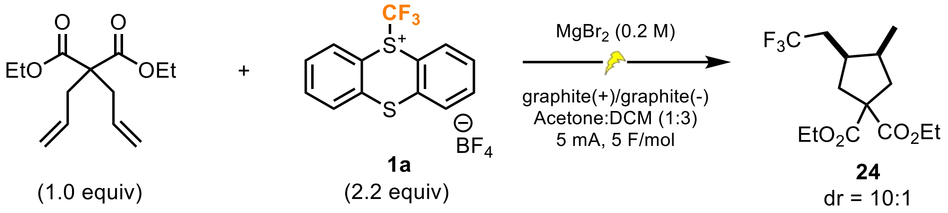

Under ambient conditions, to a 10 mL ElectraSyn vial equipped with a magnetic stir bar were added S-(Trifluoromethyl)thianthrenium tetrafluoroborate (0.20 g, 0.56 mmol, 2.2 equiv.) and magnesium bromide (0.37 g, 2.0 mmol, 8.0 equiv.). Subsequently, dichloromethane (7.5 mL) and acetone (2.5 mL) (3:1) (c = 0.025 M), diethyl diallylmalonate (60 mg, 60 µL, 0.25 mmol, 1.0 equiv.) were added at 25 °C. The ElectraSyn vial was equipped with two electrodes: graphite (+), graphite (-) and sealed with an ElectraSyn septum-cap. The reaction mixture was electrolyzed (electrolysis parameters: constant current: 5 mA, amount of charge: 5 F/mol and stirring: 1500 rpm). The yield (50%) of **24** was determined by ^{19}F NMR spectroscopy using trifluorotoluene as internal standard at δ -64.76 (t, J = 11.1 Hz). The crude product was purified by column chromatography (0-2% EtOAc in hexanes) to give **24** as a colorless oil. The product was isolated as an inseparable 10:1 mixture of diastereoisomers.

NMR Spectroscopy:

^{1}H NMR (500 MHz, $CDCl_3$, δ): 4.18 (q, *J* = 7.1 Hz, 2H), 4.18 (q, *J* = 7.2 Hz, 2H), 2.46 (ddd, *J* = 13.7, 10.7, 6.8 Hz, 2H), 2.35 – 2.23 (m, 2H), 2.21 – 1.98 (m, 4H), 1.24 (t, *J* = 7.1 Hz, 3H), 1.24 (t, *J* = 7.1 Hz, 3H), 0.86 (d, *J* = 6.8 Hz, 3H).

^{13}C NMR (126 MHz, $CDCl_3$, δ): 172.7, 172.7, 127.3 (q, *J* = 276.8 Hz), 61.7, 61.7, 58.8, 41.3, 38.1, 36.6 (t, *J* = 2.3 Hz), 36.1, 34.1 (q, *J* = 28.0 Hz), 15.0, 14.2.

^{19}F NMR (470 MHz, $CDCl_3$, δ): -64.65 (t, *J* = 10.9 Hz).

G) Deuterium Incorporation Experiments

Under an ambient atmosphere, a 5 mL Electrasyn vial was equipped with a magnetic stir bar. The vial was charged with S-(Trifluoromethyl)thianthrenium tetrafluoroborate (81.0 mg, 0.220 mmol, 2.20 equiv.), magnesium bromide (0.15 g, 0.80 mmol, 8.0 equiv.). Subsequently, dichloromethane (3.0 mL) and d_6-acetone (1.0 mL) (3:1) (c = 0.025 M), 4-allylanisole (15 µL, 0.10 mmol, 1.0 equiv.) were added at 25 °C. The ElectraSyn vial was equipped with two electrodes: graphite (+), graphite (-) and sealed with an ElectraSyn septum-cap. The reaction mixture was electrolyzed (electrolysis parameters: constant current: 5 mA, amount of charge: 5 F/mol and stirring: 1500 rpm). After conclusion, mesitylene (8.6 mg, 10 µL, 0.070 mmol, 0.66 equiv.) was added as an internal standard. The mixture was diluted with $CDCl_3$, and a quantitative ^{1}H NMR spectrum was measured. The yield (60%) of deuterated product **2-*d*** was determined by ^{1}H NMR and ^{19}F NMR spectroscopy. Non-deuterated **2** was not observed by NMR analysis. In the analysis of the ^{1}H-NMR spectrum, 9% of unreacted 4-allylanisole was also observed.

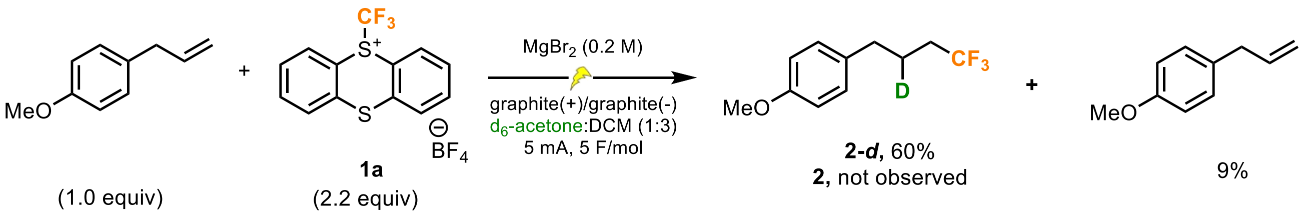

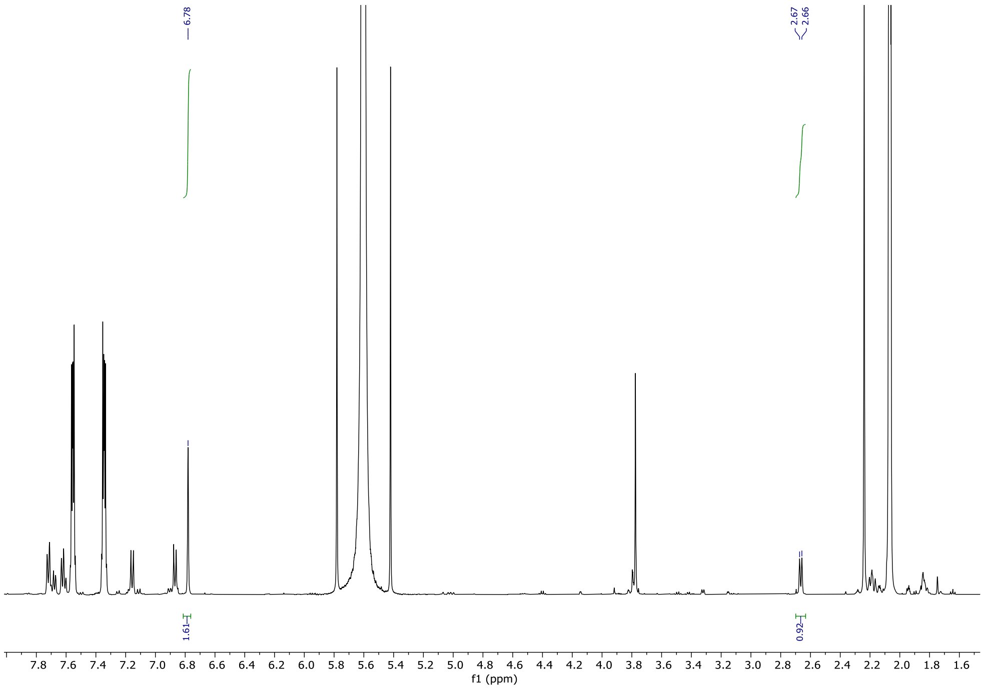

Figure S11: ^{1}H-NMR of reaction mixture of electrochemical deuterotrifluoromethylation of 4-methoxyallylbenzene using mesitylene as internal standard ($CDCl_3$, RT).

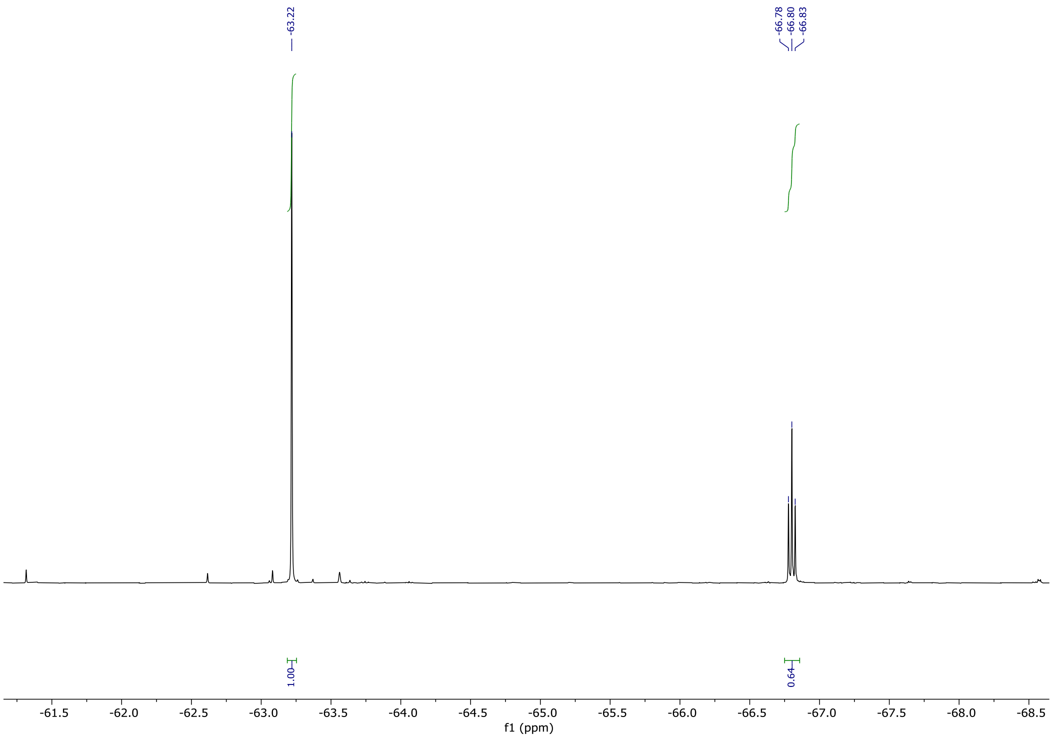

Figure S12: ^{19}F-NMR of reaction mixture of electrochemical deuterotrifluoromethylation of 4-methoxyallylbenzene using trifluorotoluene as internal standard ($CDCl_3$, RT).

H) Parallel experiments between substrates: Tertiary carbanion evidence

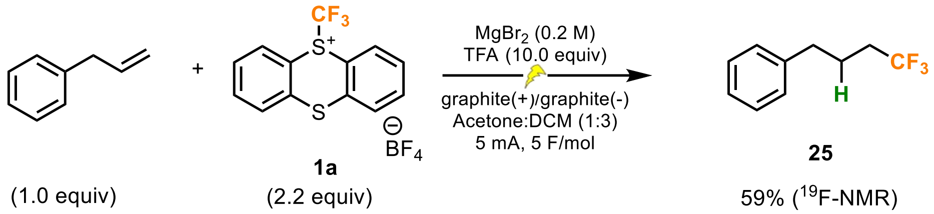

Under ambient conditions, to a 5 mL ElectraSyn vial equipped with a magnetic stir bar were added S-(Trifluoromethyl)thianthrenium tetrafluoroborate (81.0 mg, 0.218 mmol, 2.18 equiv.) and magnesium bromide (0.15 g, 0.80 mmol, 8.0 equiv.). Subsequently, dichloromethane (3.0 mL) and acetone (1.0 mL) (3:1) (c = 0.025 M), allylbenzene (13 µL, 0.10 mmol, 1.0 equiv.), and trifluoroacetic acid (80 µL, 1.0 mmol, 10 equiv.) were added at 25 °C. The ElectraSyn vial was equipped with two electrodes: graphite (+), graphite (-) and sealed with an ElectraSyn septum-cap. The reaction mixture was electrolyzed (electrolysis parameters: constant current: 5 mA, amount of charge: 5 F/mol and stirring: 1500 rpm). The yield (59%) of **25** was determined by ^{19}F NMR spectroscopy using trifluorotoluene as internal standard at δ -66.33 (t, J = 10.9 Hz).

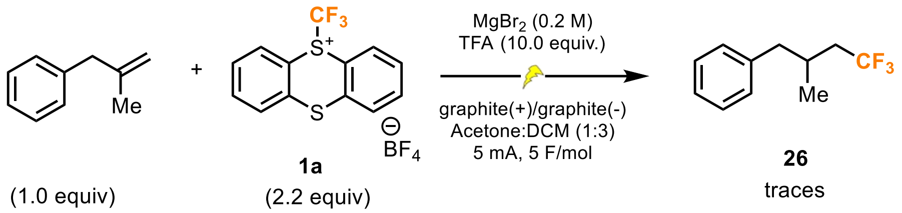

Under ambient conditions, to a 5 mL ElectraSyn vial equipped with a magnetic stir bar were added S-(Trifluoromethyl)thianthrenium tetrafluoroborate (81.0 mg, 0.218 mmol, 2.18 equiv.) and magnesium bromide (0.15 g, 0.80 mmol, 8.0 equiv.). Subsequently, dichloromethane (3.0 mL) and acetone (1.0 mL) (3:1) (c = 0.025 M), 2-methyl- 3-phenyl-1-propene (15 µL, 13 mg, 0.10 mmol, 1.0 equiv.), and trifluoroacetic acid (80 µL, 1.0 mmol, 10 equiv.) were added at 25 °C. The ElectraSyn vial was equipped with two electrodes: graphite (+), graphite (-) and sealed with an ElectraSyn septum-cap. The reaction mixture was electrolyzed (electrolysis parameters: constant current: 5 mA, amount of charge: 5 F/mol and stirring: 1500 rpm). The yield of **26** was determined by ^{19}F NMR spectroscopy using trifluorotoluene as internal standard at δ -63.67 (t, J = 11.5 Hz).

Cyclic Voltammogram studies

Cyclic voltammetry was conducted using EC-Lab and a BioLogic SP-50e potentiostat. Measurements were performed in 0.1 M $MgBr_2$ and 0.1 M $TBAPF_6$ as supporting electrolyte in anhydrous MeCN using a divided three-compartment cell (Voltammetry Cell, BASi). A glassy carbon electrode (BASi) with diameter of 3 mm was used as a working electrode. The reference electrode consisted of a Ag wire immersed in 50 mM $AgNO_3$ in 3:1 DCM/Acetone and a platinum wire was used as counter electrode. Samples containing 1 mM of $TTCF_3BF_4$,

various concentrations of olefin (1 mM, 2 mM, 3 mM and 5 mM) and 0.1 M of $MgBr_2$ were prepared in 3:1 DCM/Acetone and degassed with a stream of N_2 before measurement. Voltammograms were measured at 100 mV s^{-1} scan rate in the scan range of 0.4 to –1.0 V versus Ag^+/Ag^0. The Ag/$AgNO_3$ reference electrode was calibrated with ferrocene using a 1 mM ferrocene solution prepared in 0.1 M $MgBr_2$ and 0.1 M $TBAPF_6$ as supporting electrolyte. Analytical solution volume: 3 mL. The working electrode was polished with a synthetic rayon cloth (MicroCloth) after each measurement.

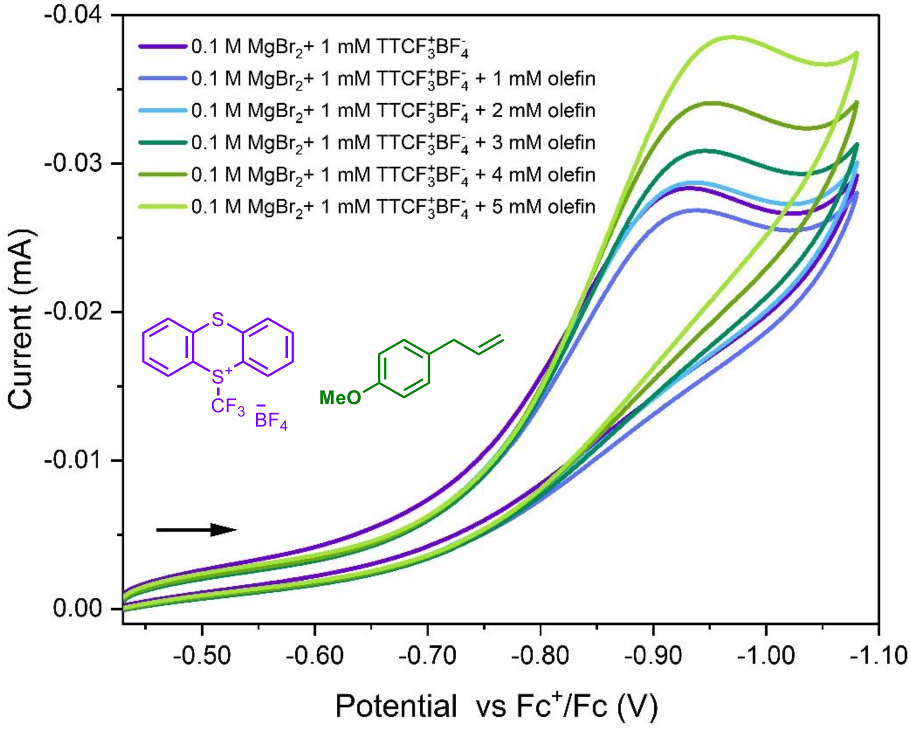

Figure S13. Cyclic voltammogram of $TTCF_3$ BF_4 (1 mM) with various concentrations of 4-allylanisole.

CV profiles

Cyclic voltammetry was conducted using EC-Lab and a BioLogic SP-50e potentiostat. Measurements were performed in 0.1 M $MgBr_2$ and 0.1 M $TBAPF_6$ as supporting electrolyte in 3:1 DCM/Acetone using a divided three-compartment cell (Voltammetry Cell, BASi). A glassy carbon disk (0.07 cm^2, BASi) was used as a working electrode. The reference electrode consisted of a Ag wire immersed in 50 mM $AgNO_3$ in 3:1 DCM/Acetone. Pt wire was used as counter electrode. Samples in 0.1 M $MgBr_2$ and 0.1 M $TBAPF_6$ as supporting electrolyte were prepared in 3:1 DCM/Acetone and degassed with a stream of N_2 before measurement. Voltammograms were measured at a scan rate of 100 mV/s. The Ag/$AgNO_3$ reference electrode was calibrated with ferrocene using a 1 mM ferrocene solution prepared in 0.1 M $MgBr_2$ and 0.1 M $TBAPF_6$ as supporting electrolyte. Analytical solution volume is 3 mL unless noted. The working electrode was polished with a synthetic rayon cloth (MicroCloth) after each measurement.

a) Cathodic CV profile

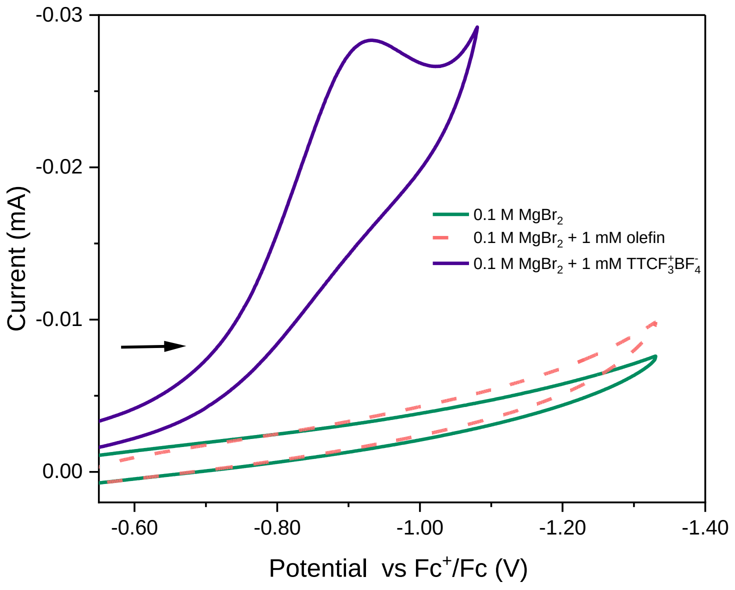

Figure S14. Cyclic Voltammogram of 4-allylanisole (1 mM), $TTCF_3BF_4$ (1 mM) and blank (0.1 M $MgBr_2$ and 0.1 M $TBAPF_6$ solution as supporting electrolyte.).

b) Anodic CV profile

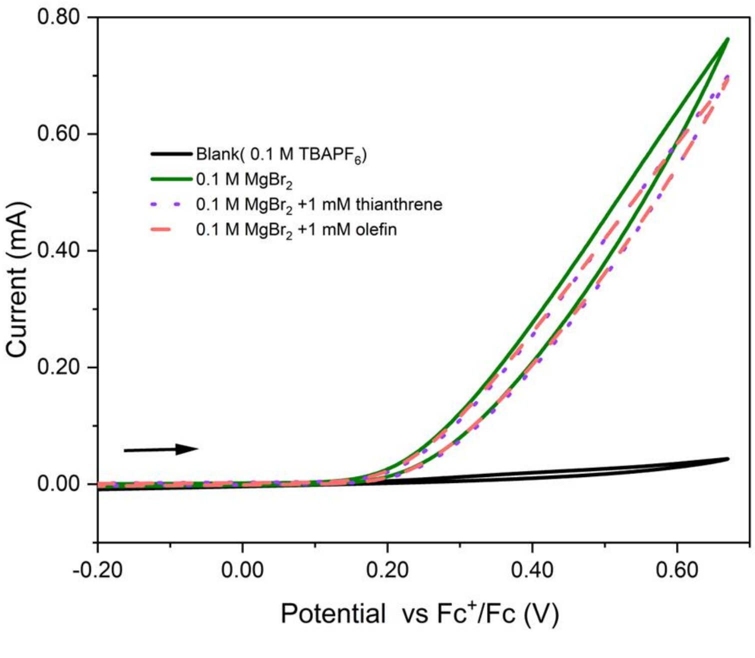

Figure S15. Cyclic Voltammogram of 4-allylanisole (1 mM), thianthrene (1 mM) in 0.1 M $MgBr_2$ and 0.1 M $TBAPF_6$ solution as supporting electrolyte.

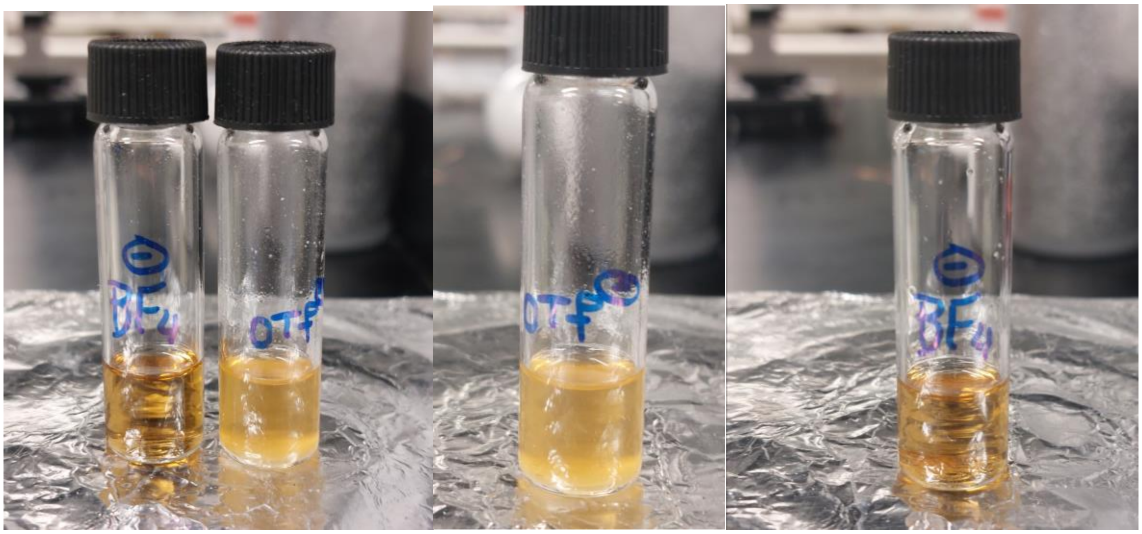

Figure S16. Pictures of the reaction mixtures of (a) $TTCF_3OTf$ with $MgBr_2$ in DCM/Acetone (3:1) and (b) $TTCF_3BF_4$ with $MgBr_2$ in DCM/Acetone (3:1).

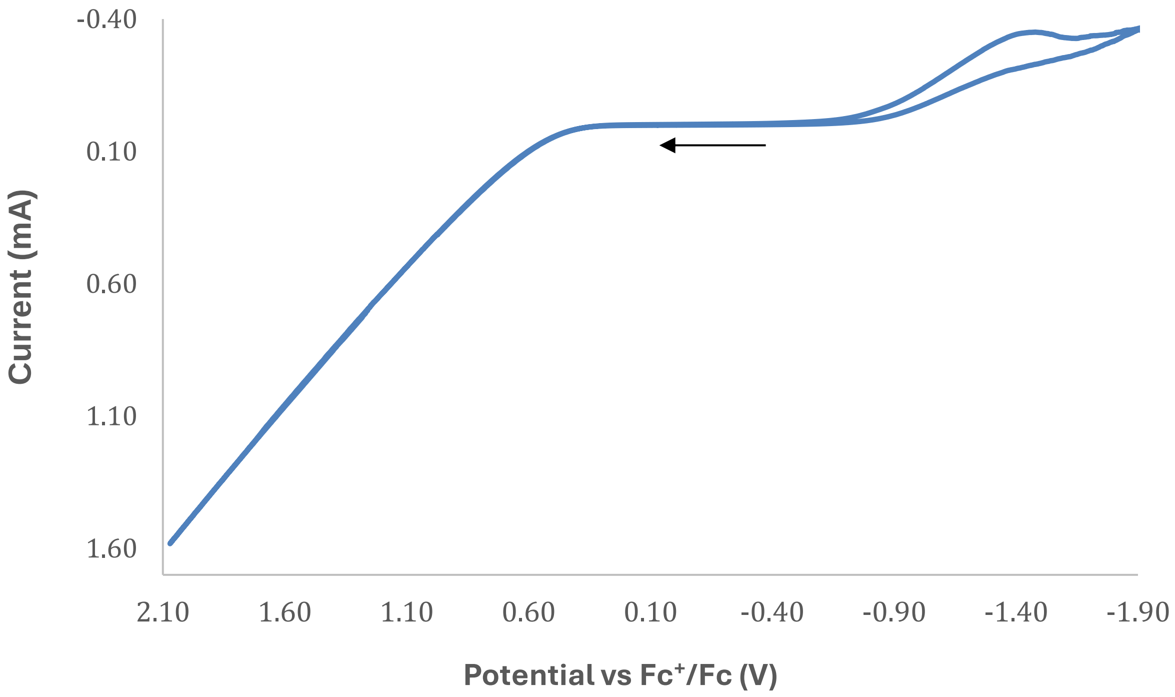

Figure S17. Cyclic Voltammogram of a mixture of $TTCF_3BF_4$ and $MgBr_2$ in DCM:Acetone (3:1). Conditions: $MgBr_2$ (0.2 M), $TTCF_3BF_4$ (54 mM). Scan rate: 50 mV/s.

Constant Potential Experiment

An oven-dried, 20 mL glass tube was equipped with a magnetic stir bar, a threaded Teflon cap fitted with

electrical feed-throughs, a graphite electrode as cathode and anode (purchased from IKA), and a Ag/Ag^+ reference electrode with 0.1 M $TBAPF_6$ in DCM/Acetone and 50 mM $AgNO_3$ in MeCN. The reference electrode was calibrated against ferrocene (Fc) as an internal standard. To the reaction vessel, S-(Trifluoromethyl)thianthrenium tetrafluoroborate (81.0 mg, 0.220 mmol, 2.2 equiv.) and magnesium bromide (0.15 g, 0.80 mmol, 8.0 equiv.). Subsequently, dichloromethane (3.0 mL), acetone (1.0 mL) (3:1) (c = 0.025 M), and 4-allylanisole (15 µL, 0.10 mmol, 1.0 equiv.) were added at 25 °C. The vessel was properly sealed, and the electrolysis was performed at ambient conditions and constant cathodic potential of -1.2 V vs Fc/Fc^+ at room temperature for 2 h and consuming half capacity (7 mAh).

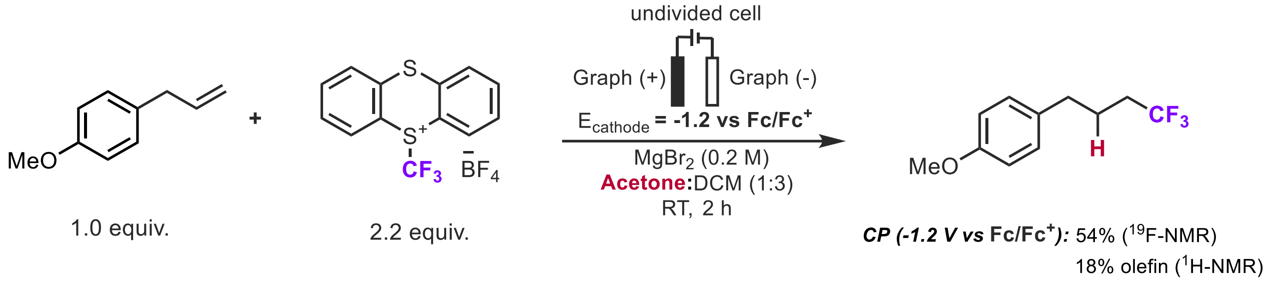

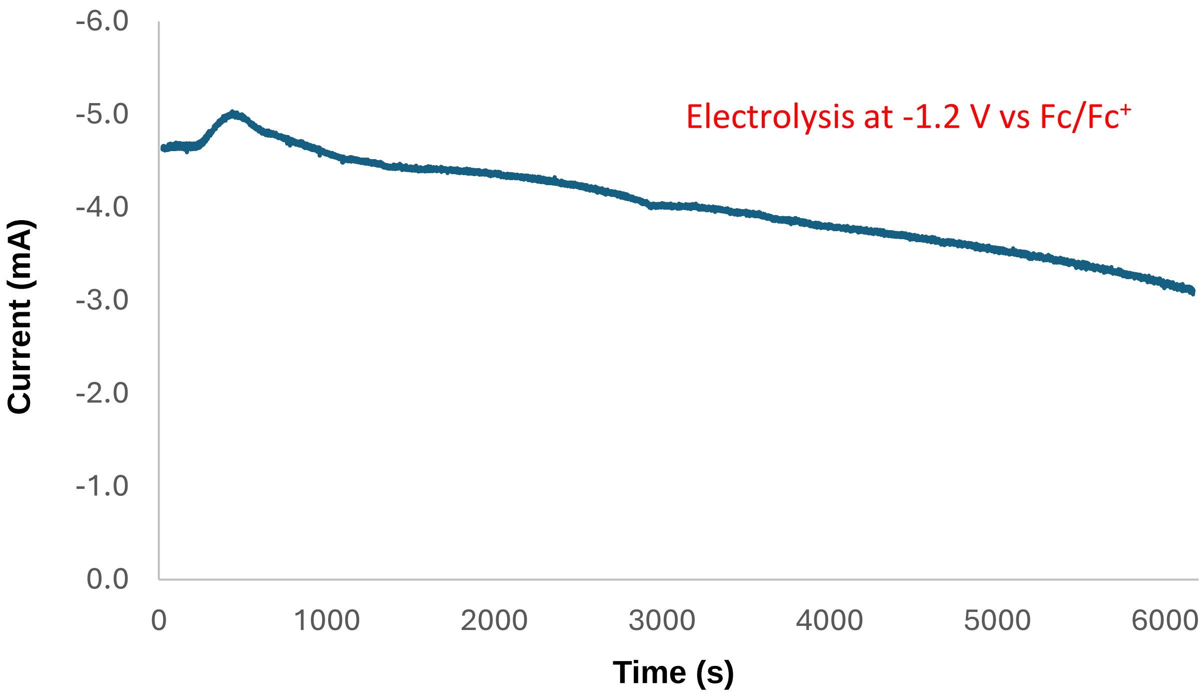

Figure S18. Current profile under constant cathodic voltage of -1.20 V vs Fc/Fc^+.

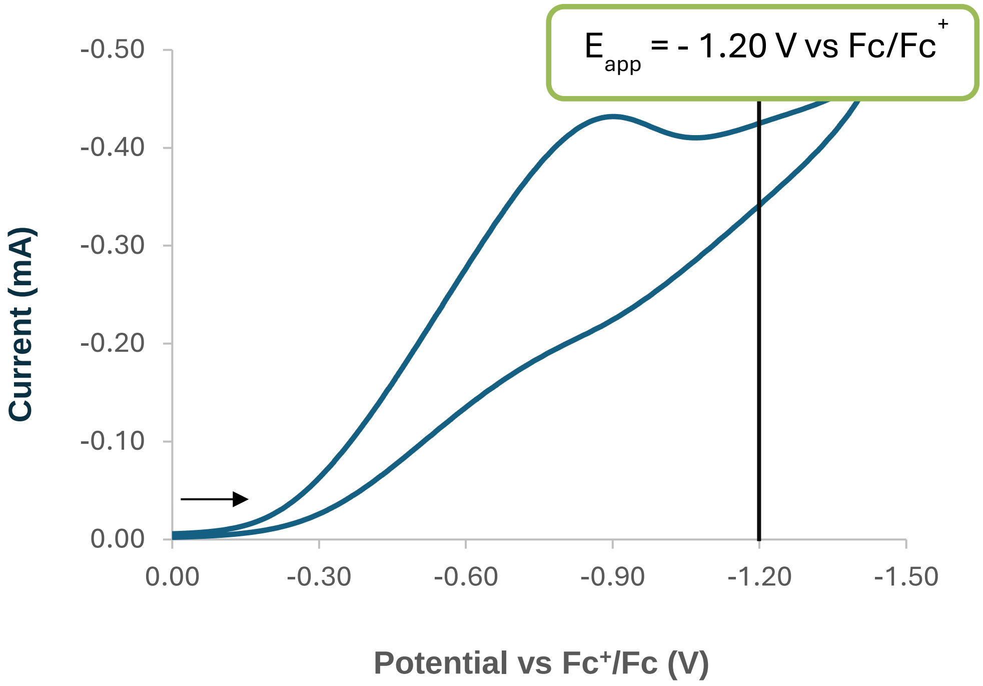

Figure S19. Cyclic voltammogram for the constant potential electrolysis (CPE) at – 1.20 V vs Fc/Fc$^+$. CV conditions: 25 mM 4-allylanisole, 100 mM $TBAPF_6$ in DCM/Acetone using a GC disk working electrode and Pt counter electrode with a $Ag/AgNO_3$ reference electrode at 50 mV/s. CPE conditions: 0.10 mmol of 4-allylanisole, 0.22 mmol of S-(Trifluoromethyl)thianthrenium tetrafluoroborate, 200 mM $MgBr_2$, and Grap(+)|Grap(–) with a $Ag/AgNO_3$ reference electrode in an undivided cell (2.6 e^- passed).

Voltage Profile Measurement

All experiments were conducted under ambient conditions considering our reaction is insensitive to air. Electrochemical hydrotrifluoromethylation was performed in a three electrode configuration using EC-Lab and a BioLogic SP-50e potentiostat. An oven-dried, 20 mL glass tube was equipped with a magnetic stir bar, a threaded Teflon cap fitted with electrical feed-throughs, a graphite electrode as cathode and anode (purchased from IKA), and a Ag/Ag^+ reference electrode with 0.1 M $TBAPF_6$ in DCM/Acetone and 50 mM $AgNO_3$ in DCM/Acetone. The working and counter electrode were connected to the potentiostat via copper wire. The reference electrode was calibrated against ferrocene (Fc) as an internal standard. To the reaction vessel, S-(Trifluoromethyl)thianthrenium tetrafluoroborate (0.16 g, 0.44 mmol, 2.2 equiv.) and magnesium bromide (0.29 g, 1.6 mmol, 8.0 equiv.). Subsequently, dichloromethane (6.0 mL), acetone (2.0 mL) (3:1) (c = 0.025 M), and 4-allylanisole (32 µL, 0.20 mmol, 1.0 equiv.) were added at 25 °C. The vessel was properly sealed, and the electrolysis was performed at ambient conditions and electrolyzed at a constant current of -5.0 mA until passing 2.5 F/mol charge. For the experiment using S-(Trifluoromethyl)thianthrenium triflate (0.44 mmol), the reaction ran until exceeding the compliance limits of the potentiostat (- 10.0 V) and stopped early.

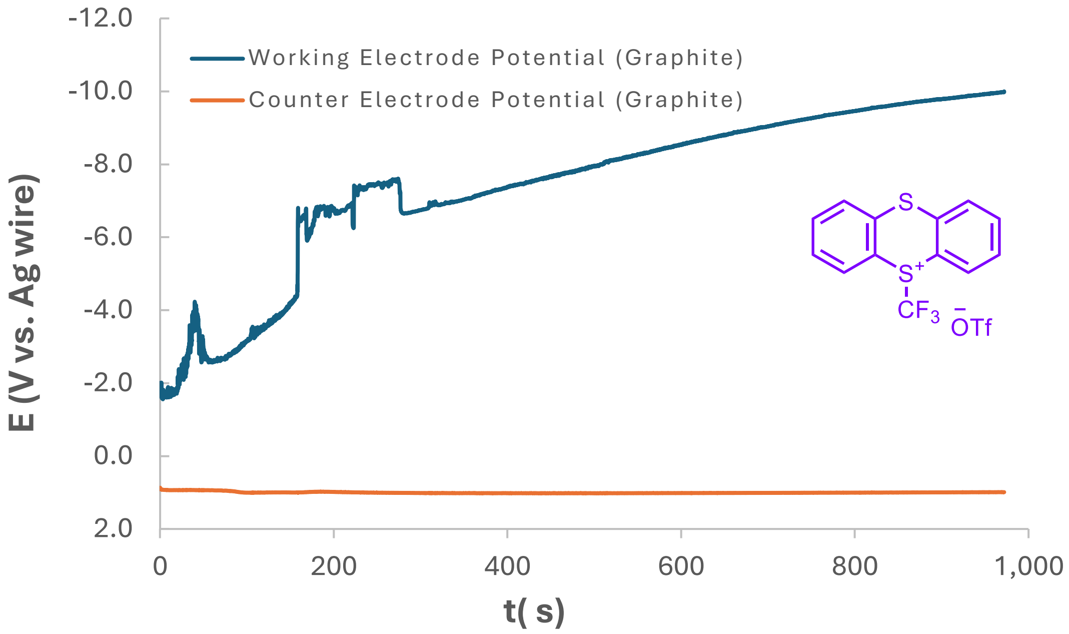

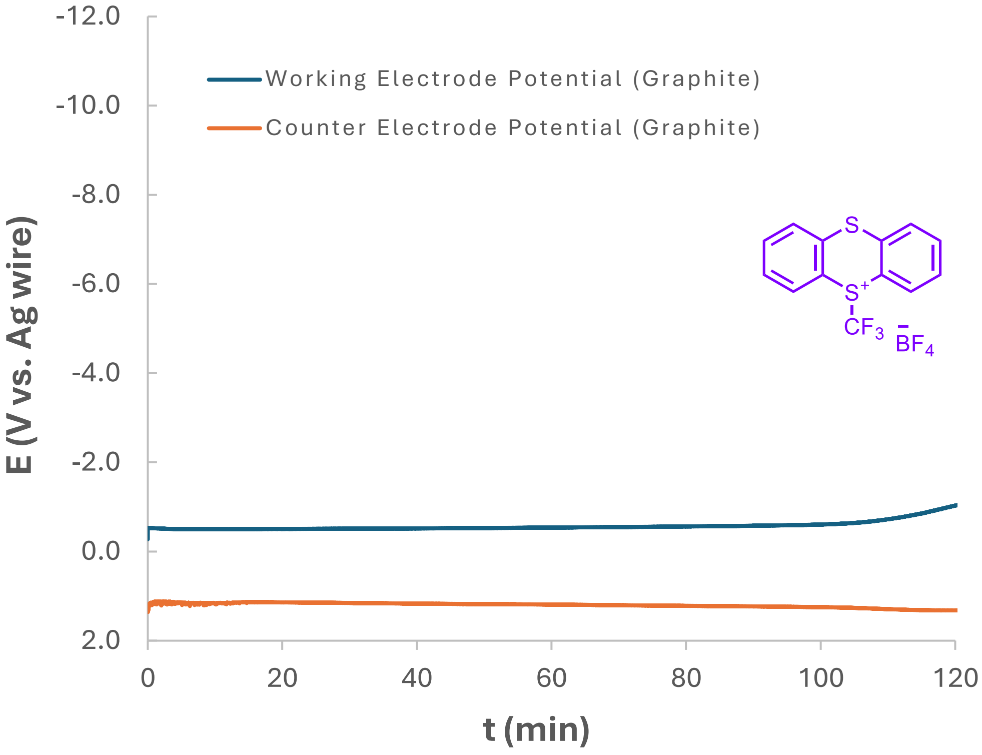

Figure S20. Voltage profile of the graphite counter electrode (E_{CE}) and graphite working electrode (E_{WE}) during the electrochemical hydrotrifluoromethylation of unactivated olefins using (a) $TTCF_3OTf$ and (b) $TTCF_3BF_4$.

a) b)

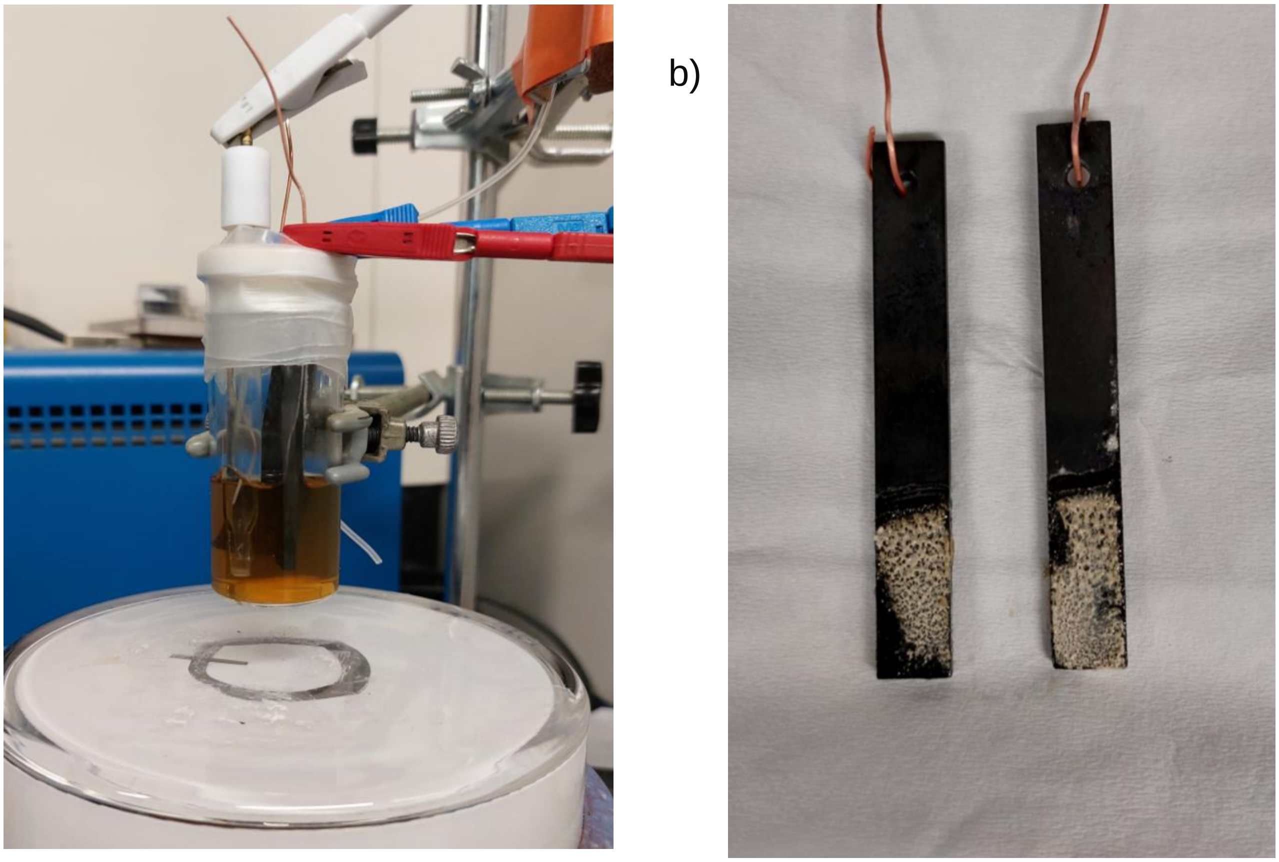

Figure S21. a) Electrochemical setup for voltage profile measurements using $TTCF_3BF_4$. b) Graphite electrodes (counter and working) after electrolysis.

Redox potential substrates (Functional group tolerance)

Cyclic voltammograms of substrates 1-boc-indole (1 mM), 4-hydroxybenzyl alcohol (1 mM), bis(4-bromophenyl) amine (5mM), quinuclidine (5 mM), 2-(methylthio)benzothiazole (5 mM), N-methyl imidazole (5 mM), and 2-methoxypyridine (5 mM) were run in DCM: Acetone (3:1) in $TBAPF_6$ as supporting electrolyte at a scan rate of 100 mV/s (Oxidation Profile). Cyclic voltammograms of substrates N-allyl phthalimide (5 mM) and 1,2-epoxy-9-decene (100 mM) were run in DCM: Acetone (3:1) in $TBAPF_6$ as supporting electrolyte at a scan rate of 100 mV/s (Reduction Profile). The samples were degassed before measurement. The Ag/AgCl reference electrode was calibrated with ferrocene (5 mM). Potentials for irreversible waves were estimated at half the maximum current ($E_{p/2}$), as previously described by Nicewicz.[7]

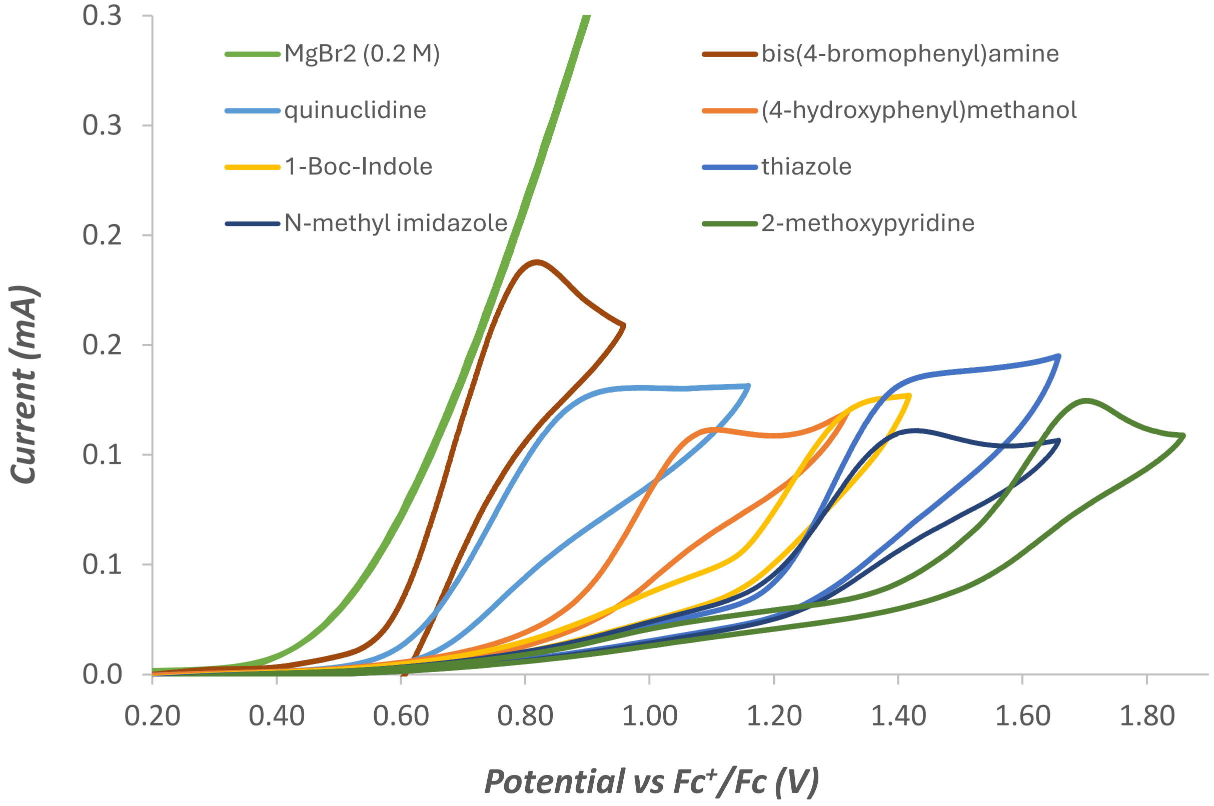

Figure S22. Functional group tolerance of substrates with the redox potential of the electroreductive hydrotrifluoromethylation (Oxidation profile).

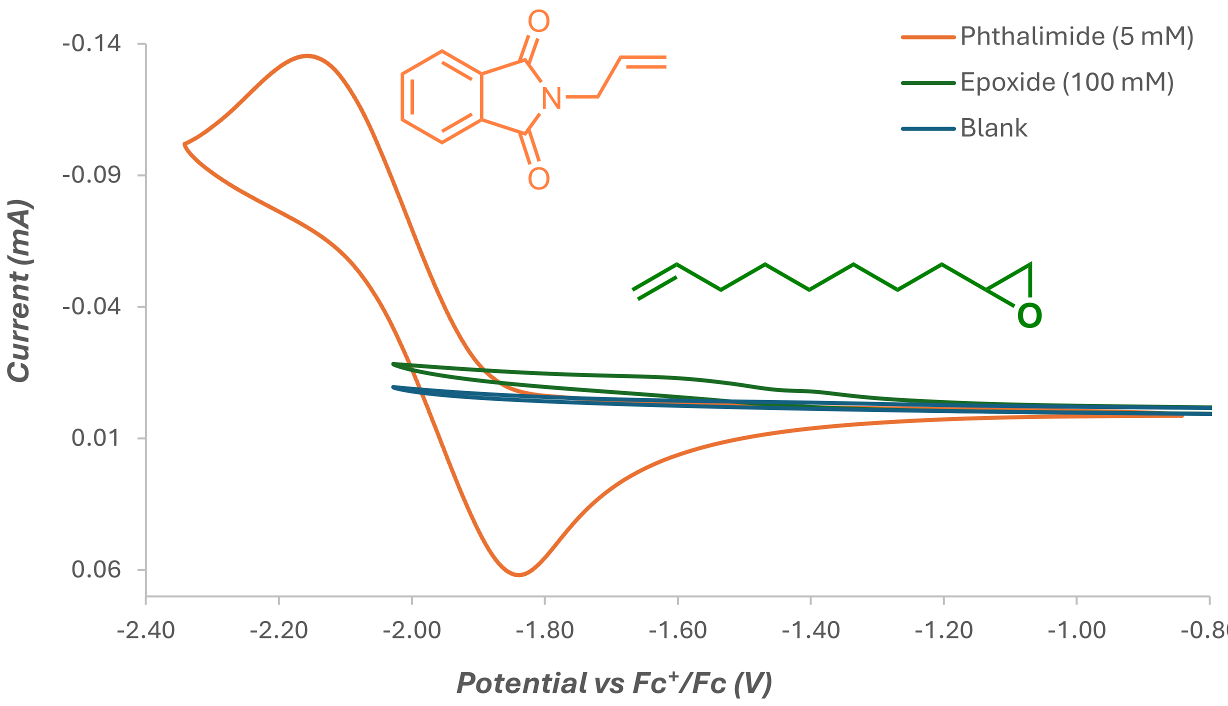

Figure S23. Functional group tolerance of substrates with the redox potential of the electroreductive hydrotrifluoromethylation (Reduction profile).

SPECTROSCOPIC DATA

^{1}H NMR of nicotinic ester hydrotrifluoromethylated derivative (S1)

$CDCl_3$

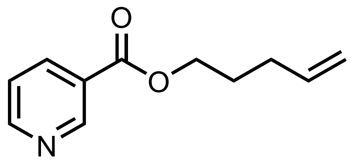

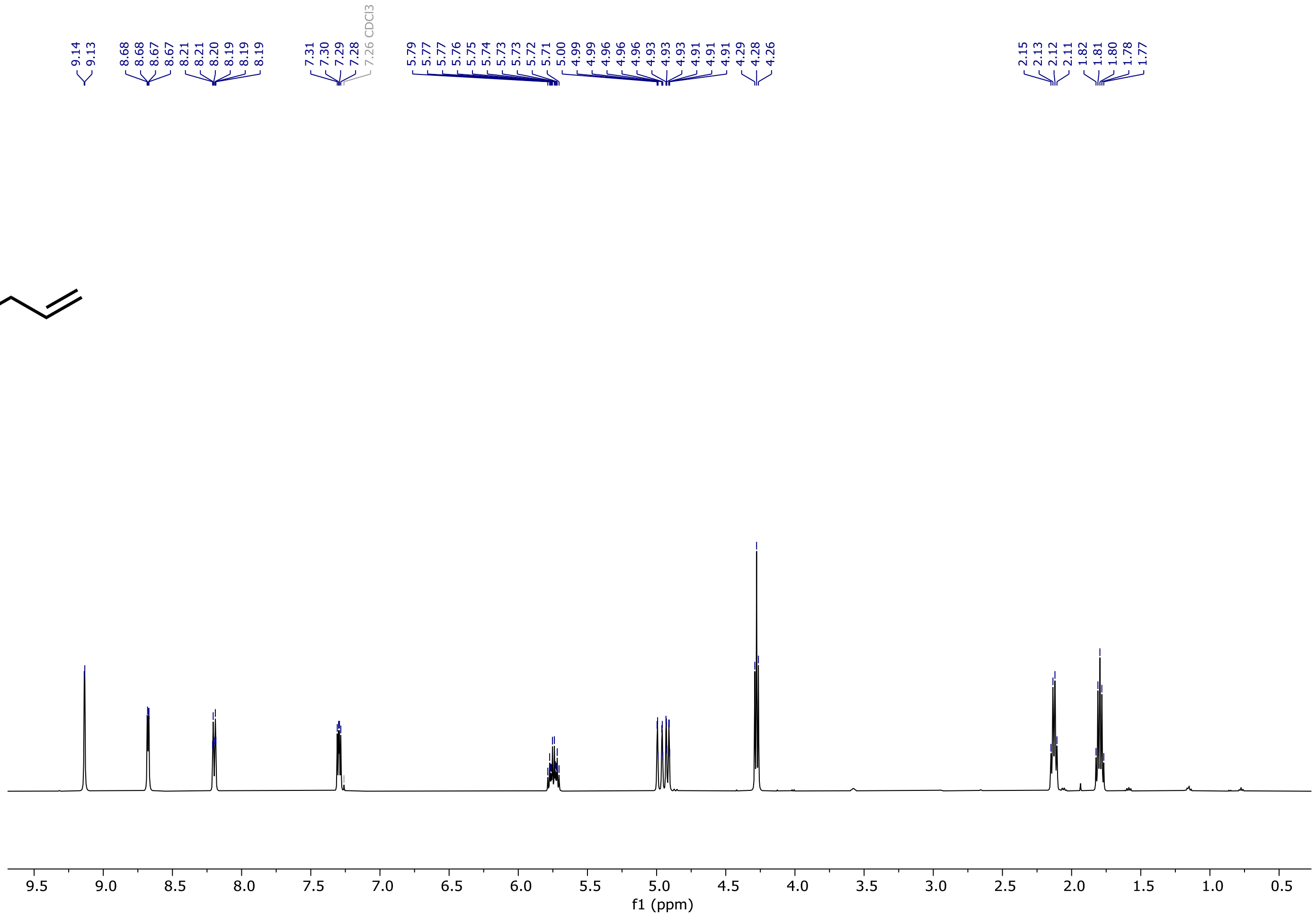

^{13}C NMR of nicotinic ester hydrotrifluoromethylated derivative (S1)

CDCl$_3$

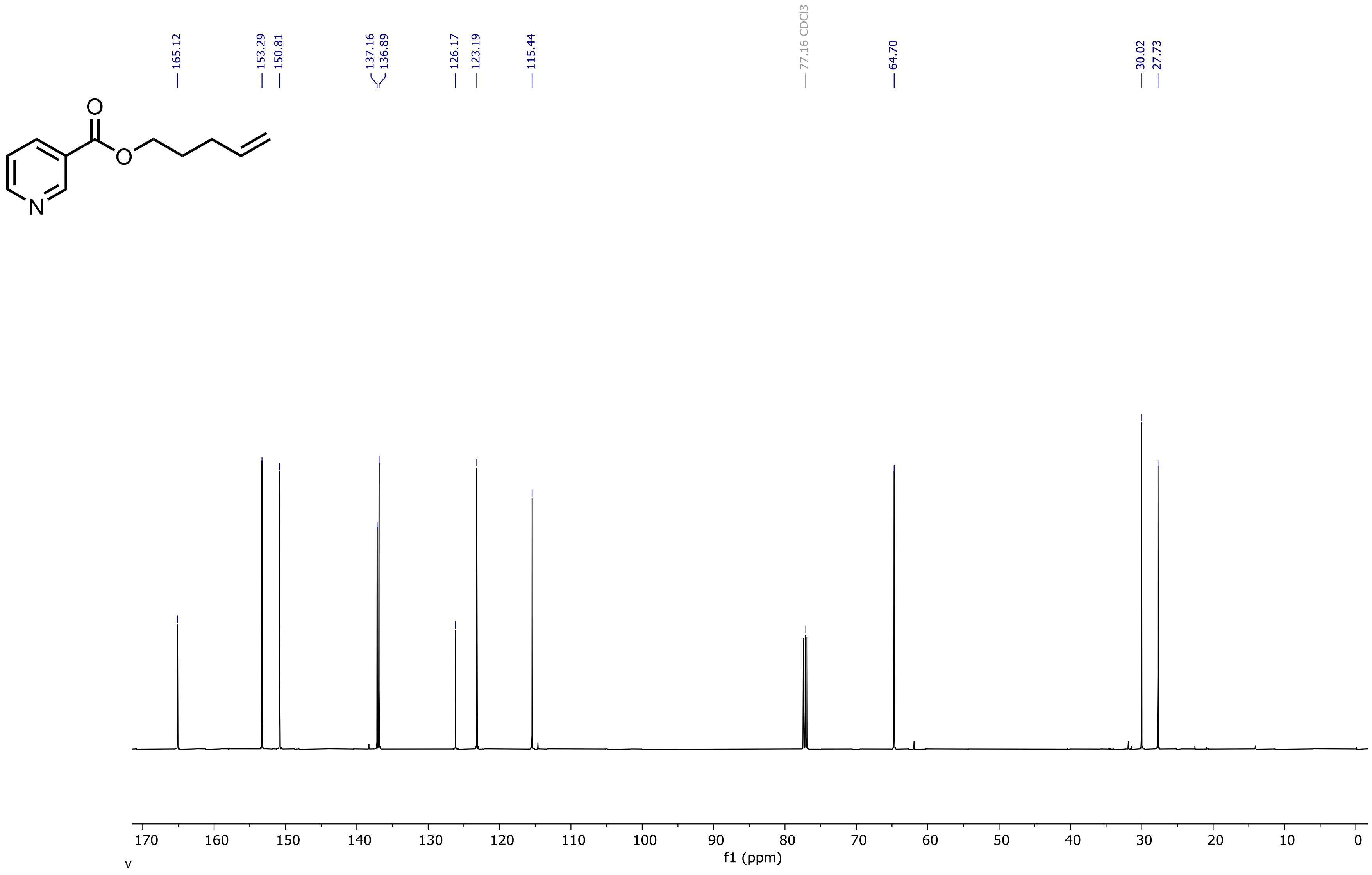

^{1}H NMR of 1-methoxy-4-(4,4,4-trifluorobutyl)benzene (2)

$CDCl_3$

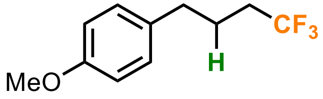

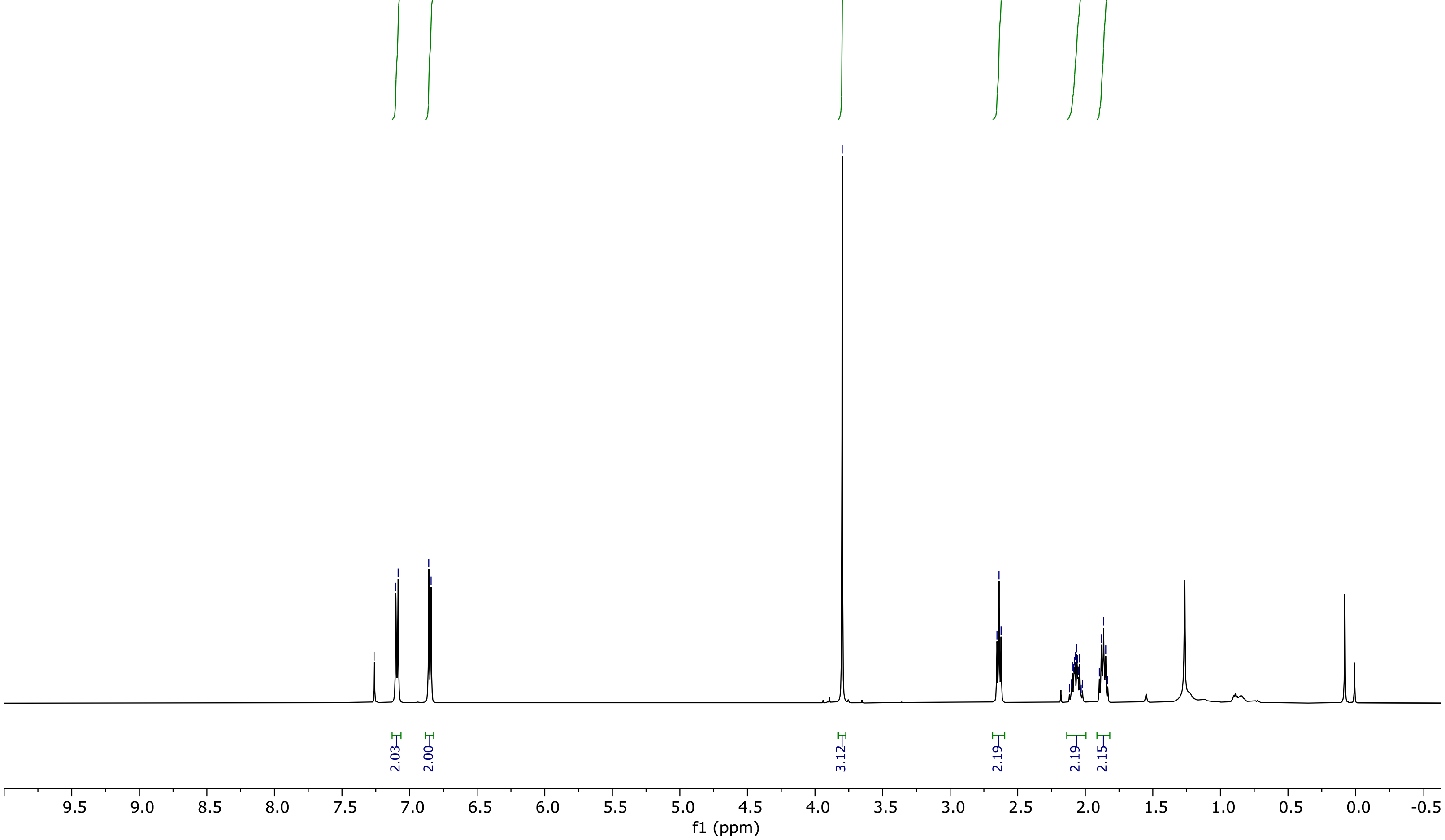

^{13}C NMR of 1-methoxy-4-(4,4,4-trifluorobutyl)benzene (2)

$CDCl_3$

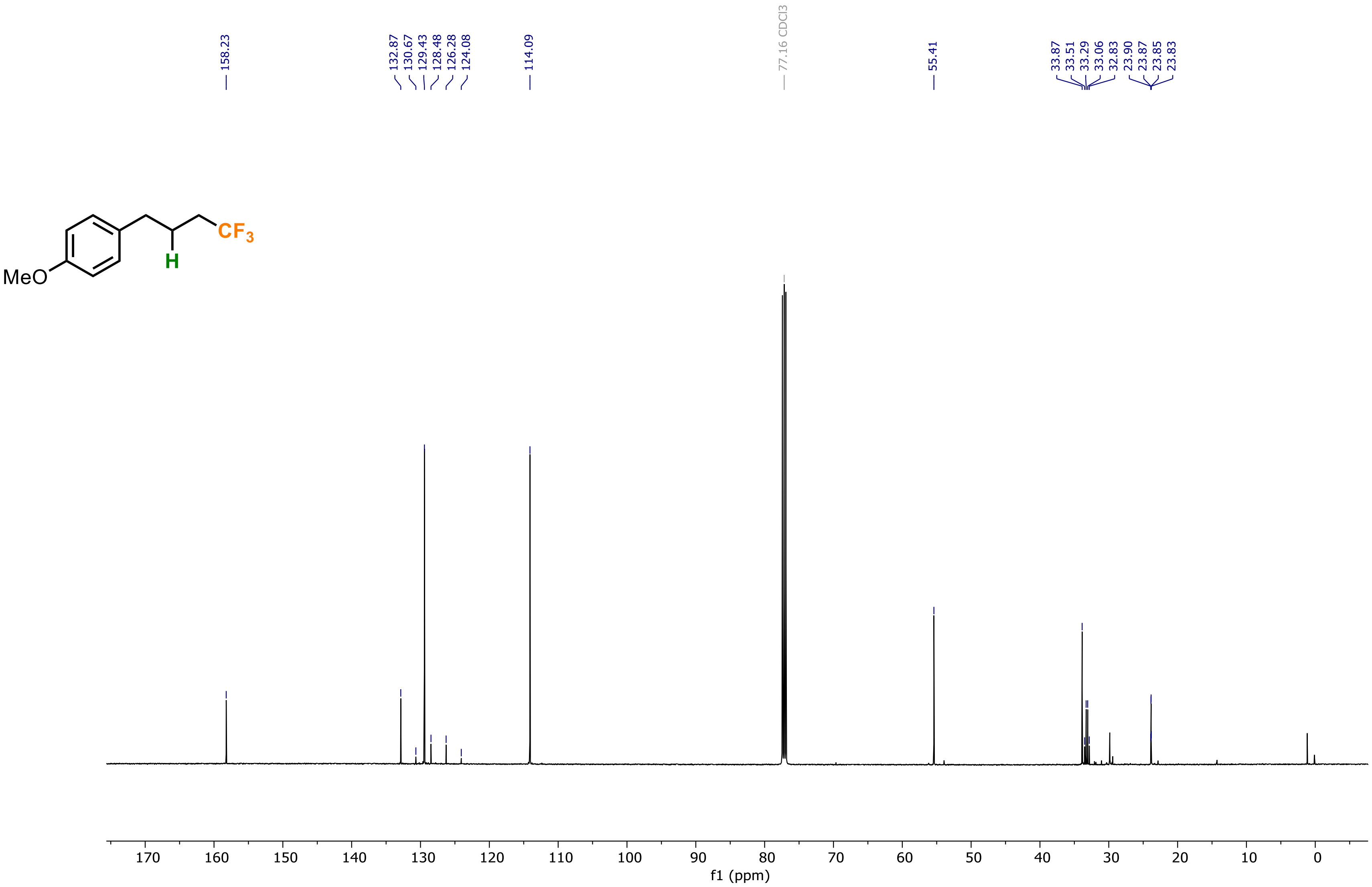

^{19}F NMR of 1-methoxy-4-(4,4,4-trifluorobutyl)benzene (2)

$CDCl_3$

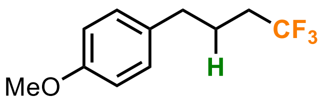

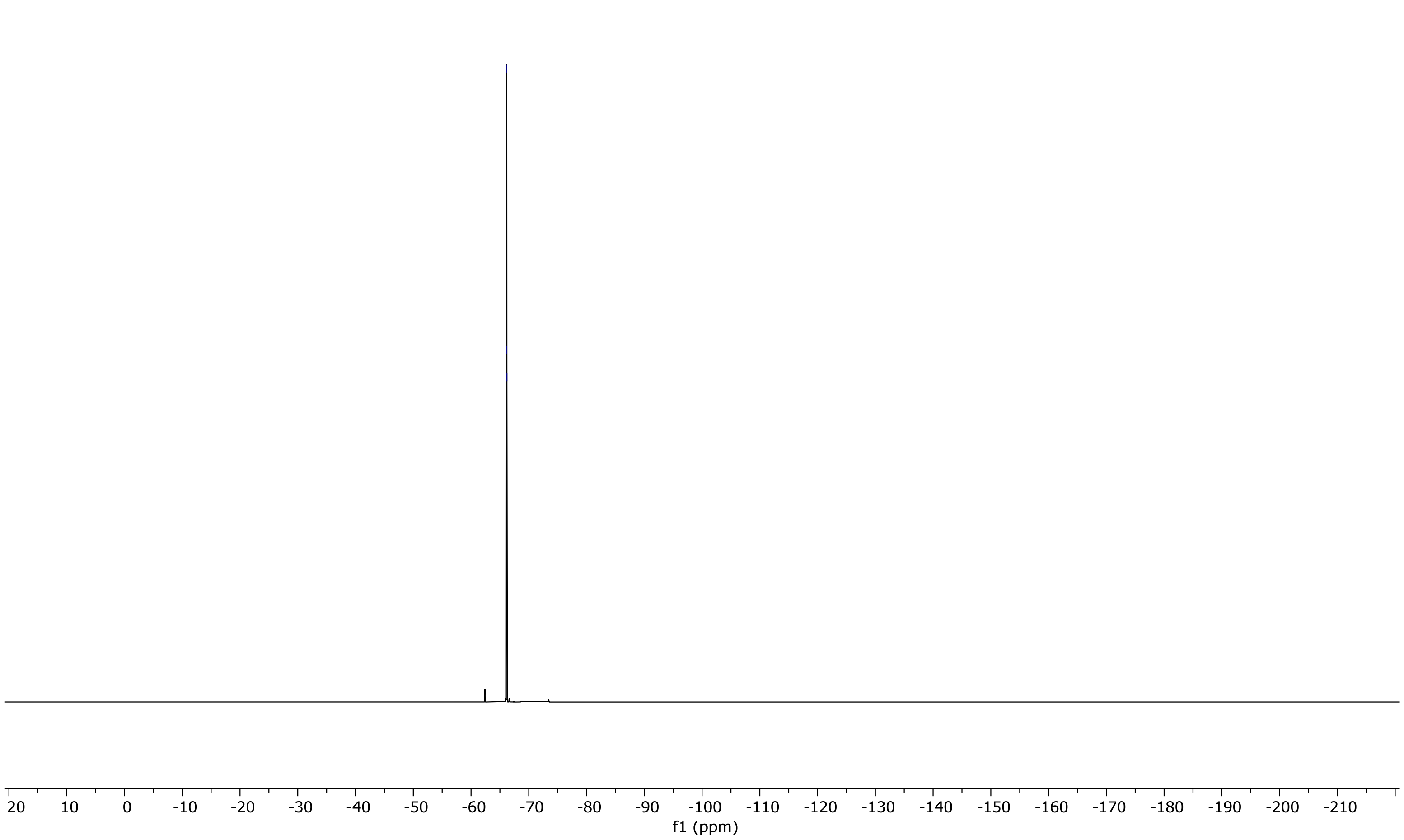

^{1}H NMR of 1-bromo-2-(4,4,4-trifluorobutyl)benzene (4)

$CDCl_3$

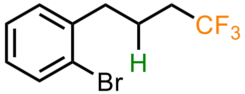

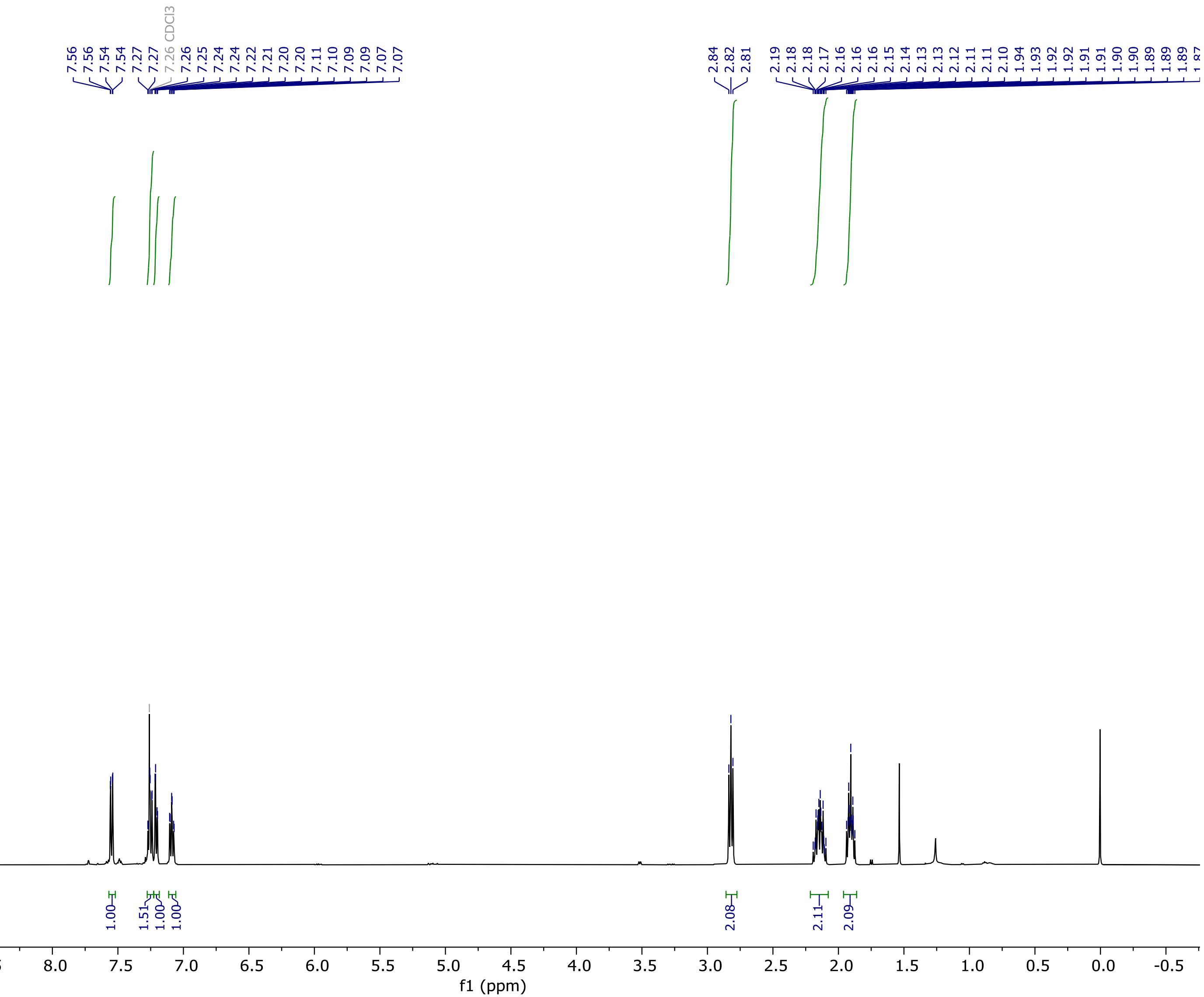

^{13}C NMR of 1-bromo-2-(4,4,4-trifluorobutyl)benzene (4)

$CDCl_3$

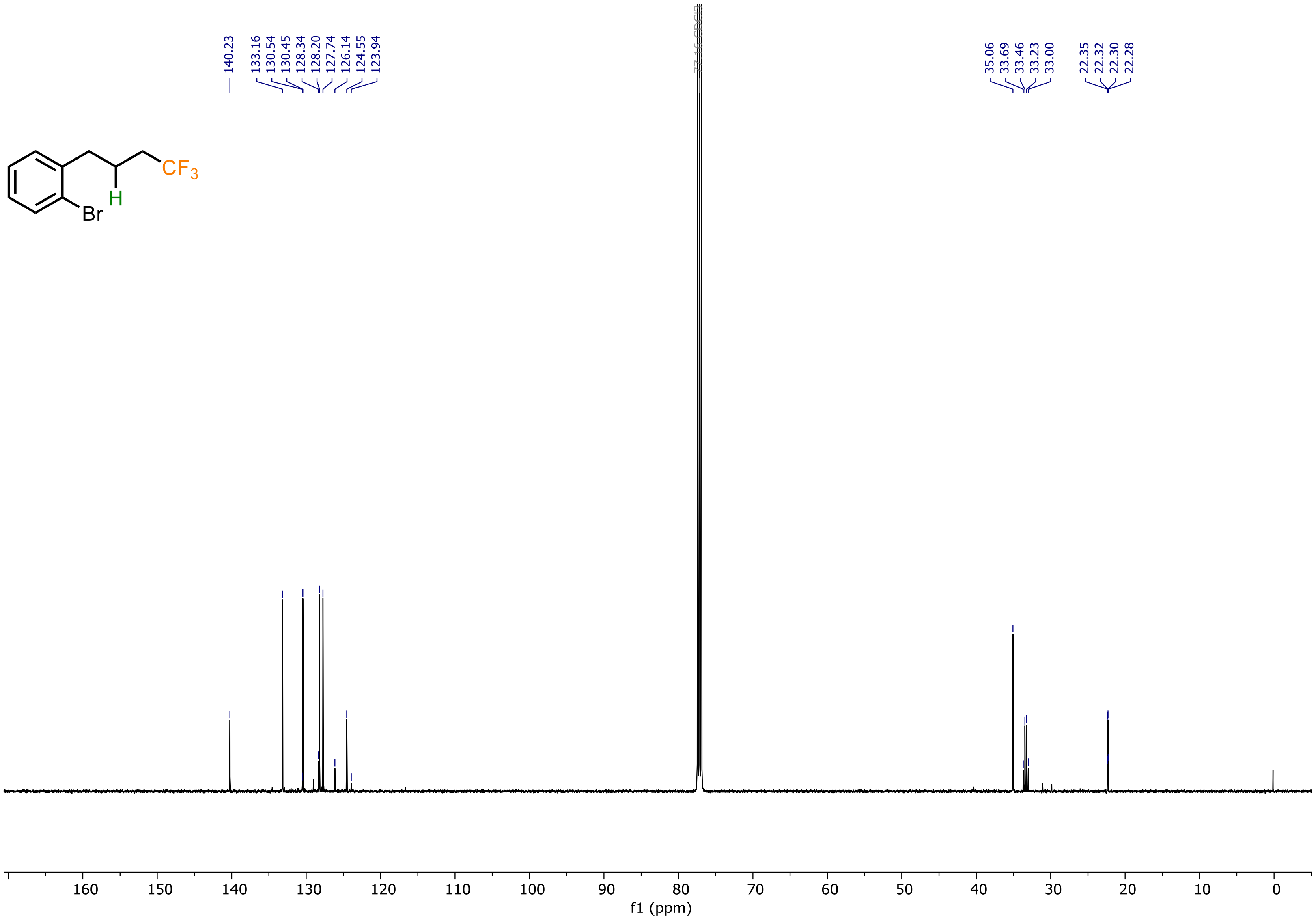

^{19}F NMR of 1-bromo-2-(4,4,4-trifluorobutyl)benzene (4)

$CDCl_3$

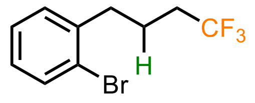

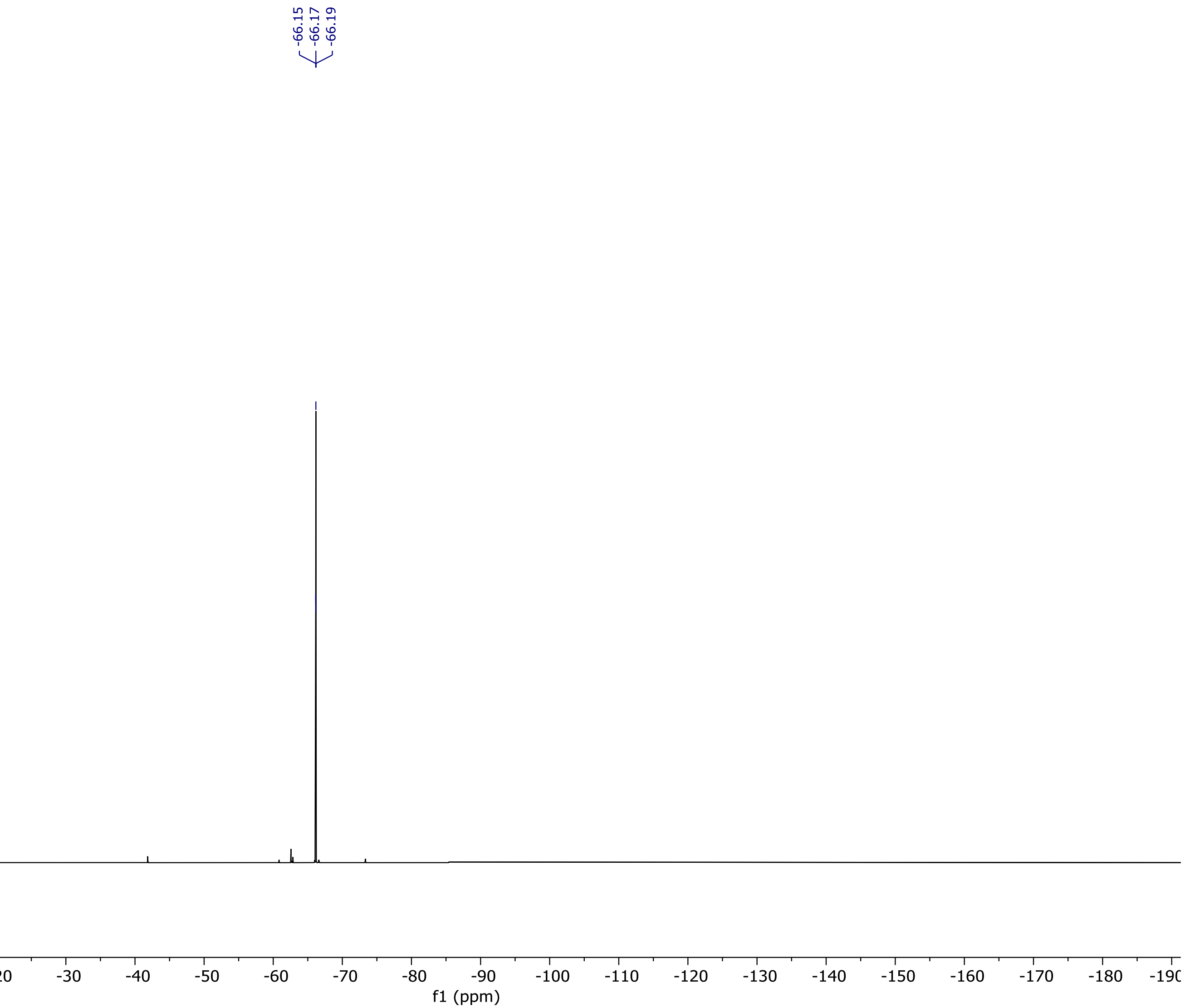

^{1}H NMR of (5,5,5-trifluoropentyl)benzene (5)

CDCl3

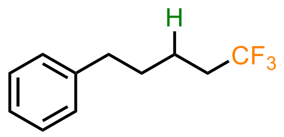

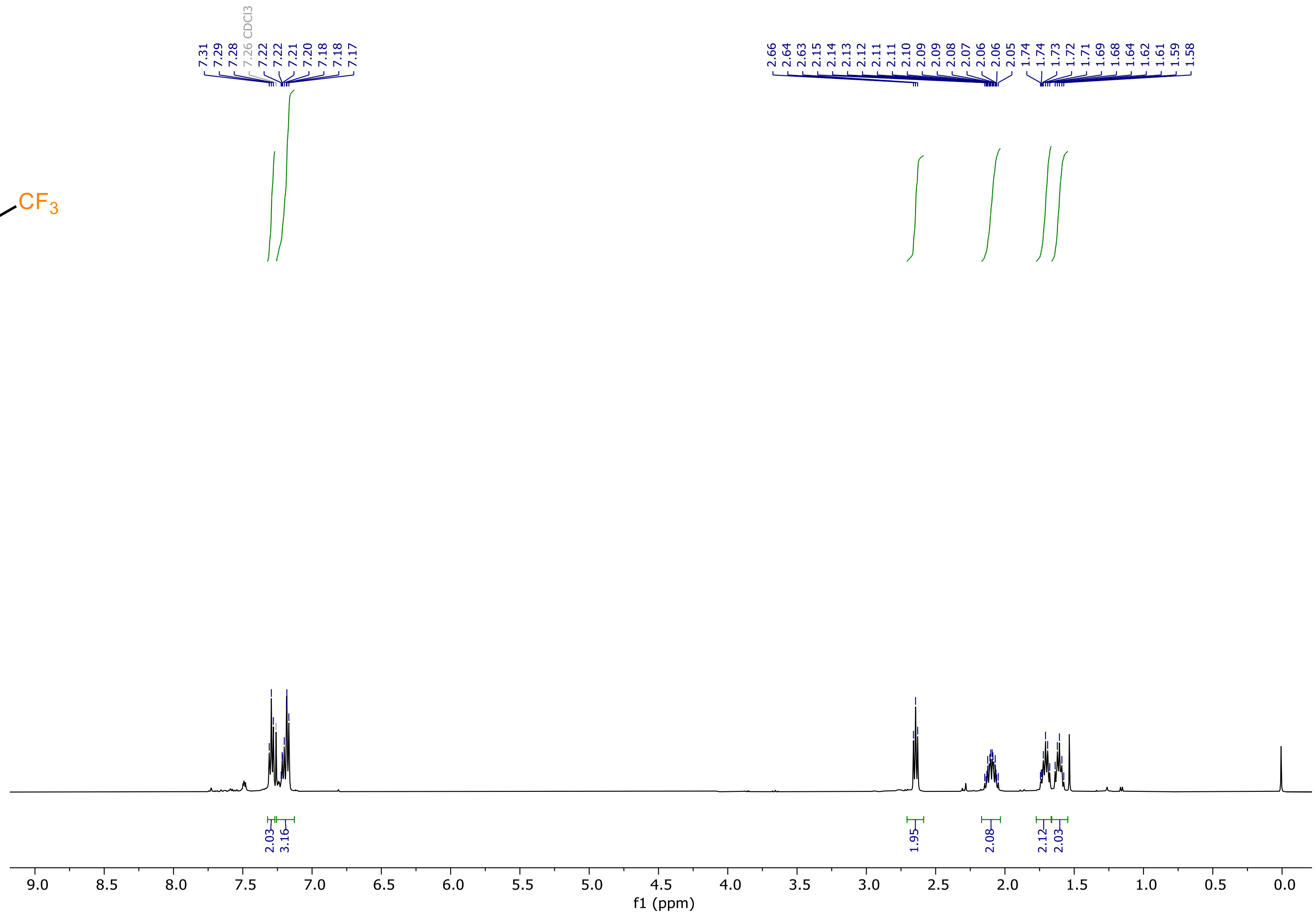

^{19}F NMR of (5,5,5-trifluoropentyl)benzene (5)

$CDCl_3$

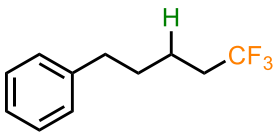

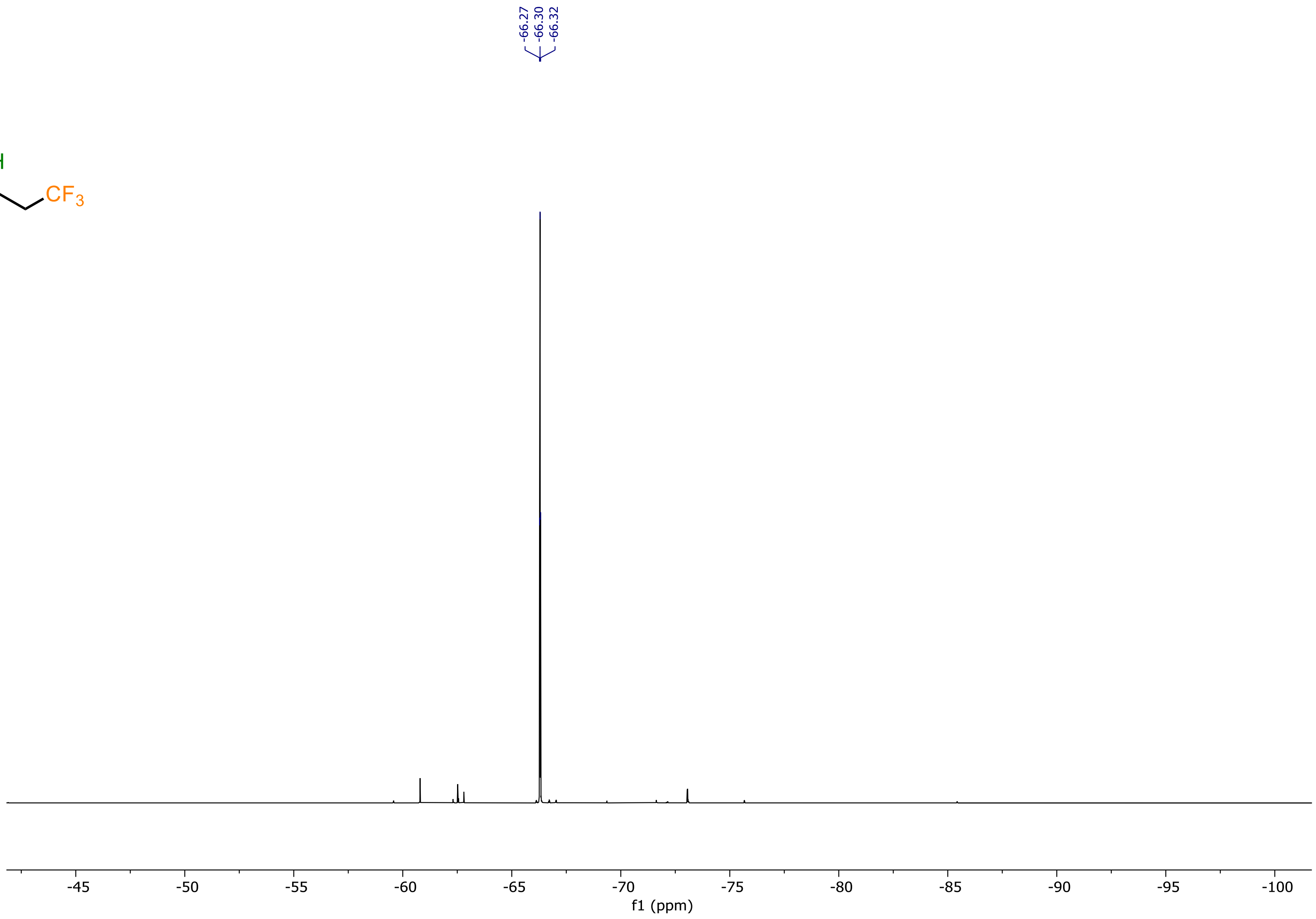

^{1}H NMR of (4,4,4-trifluorobutoxy)benzene (6)

$CDCl_3$

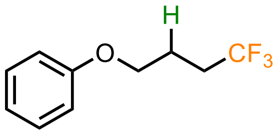

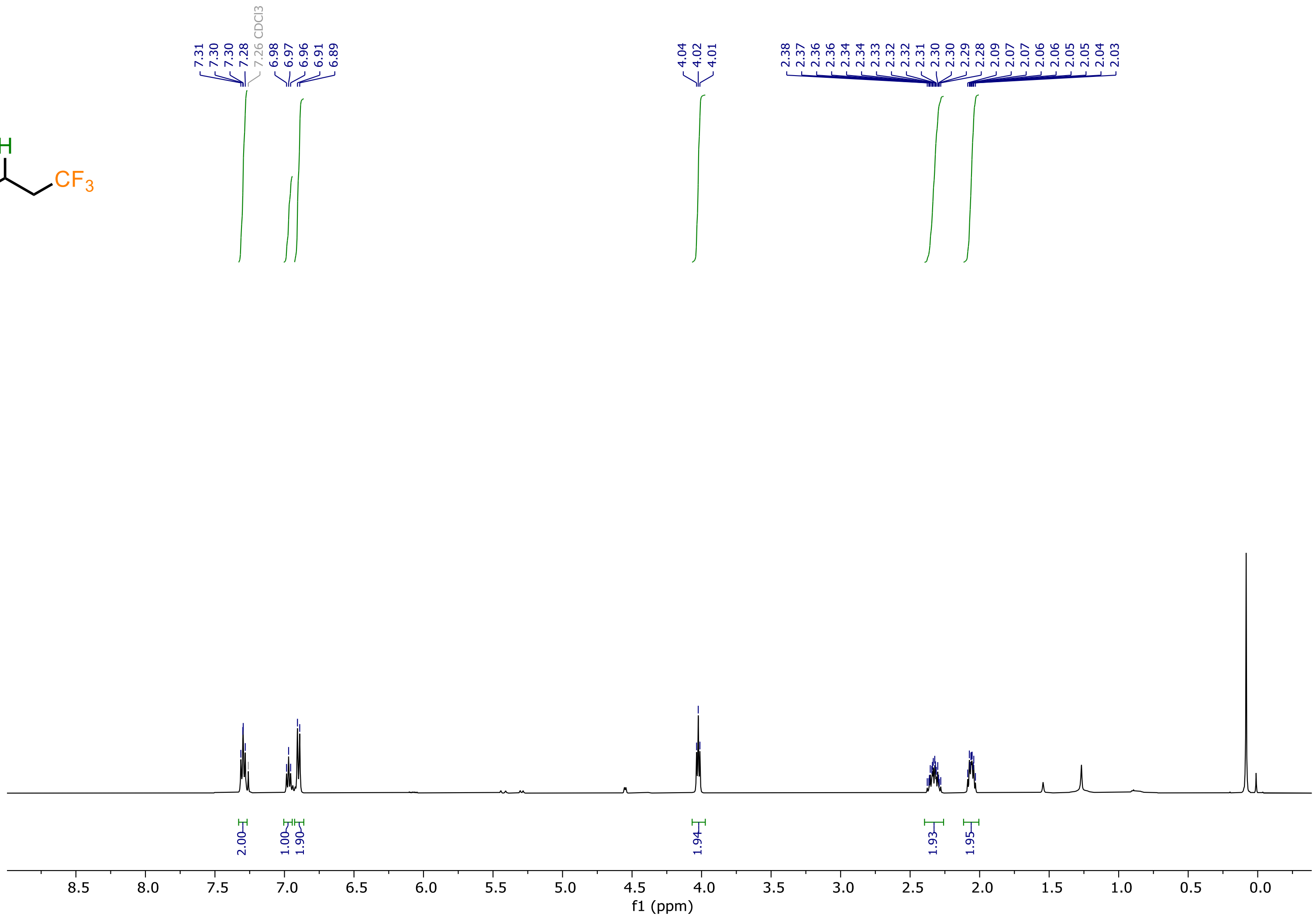

^{13}C NMR of (4,4,4-trifluorobutoxy)benzene (6)

$CDCl_3$

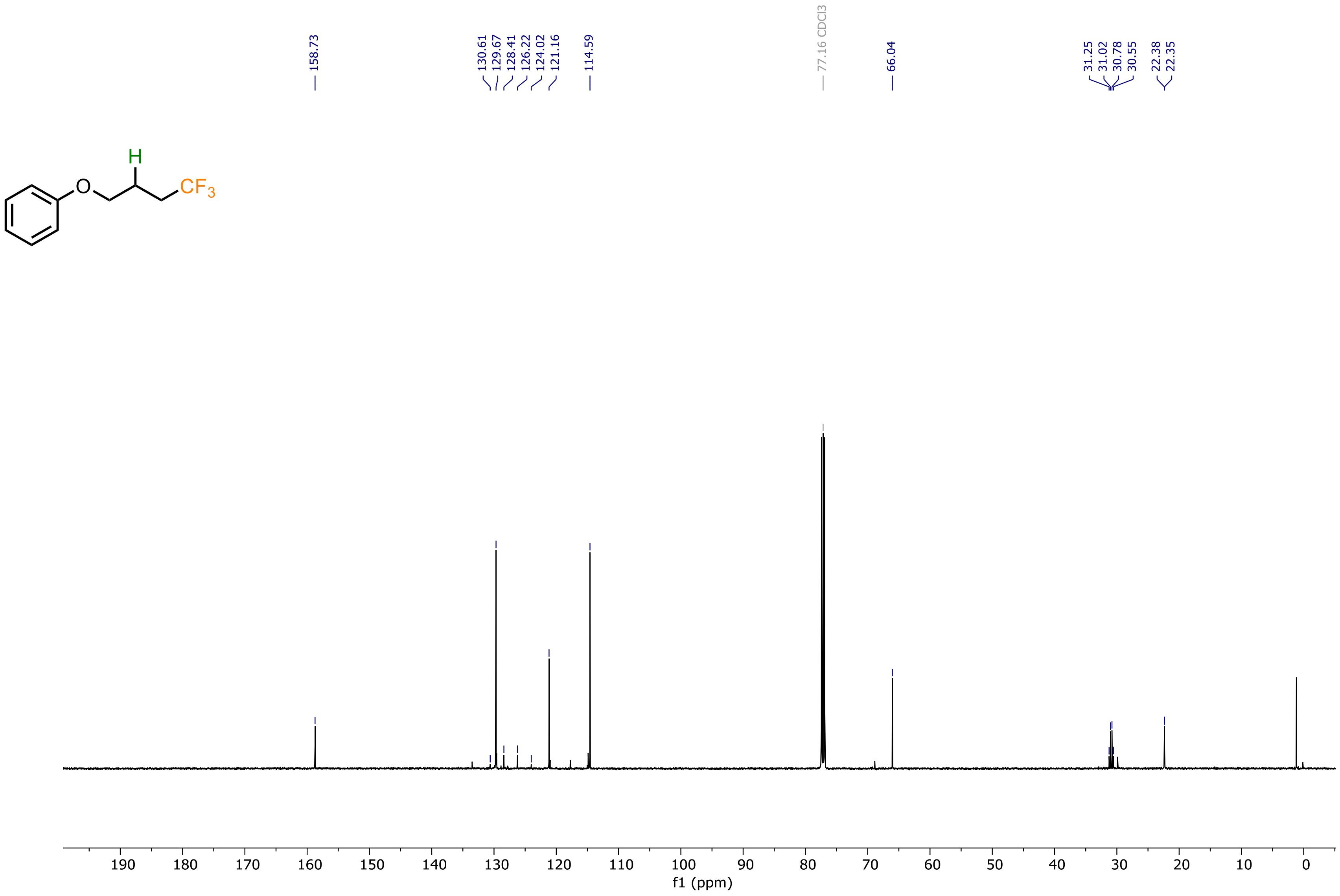

^{19}F NMR of (4,4,4-trifluorobutoxy)benzene (6)

$CDCl_3$

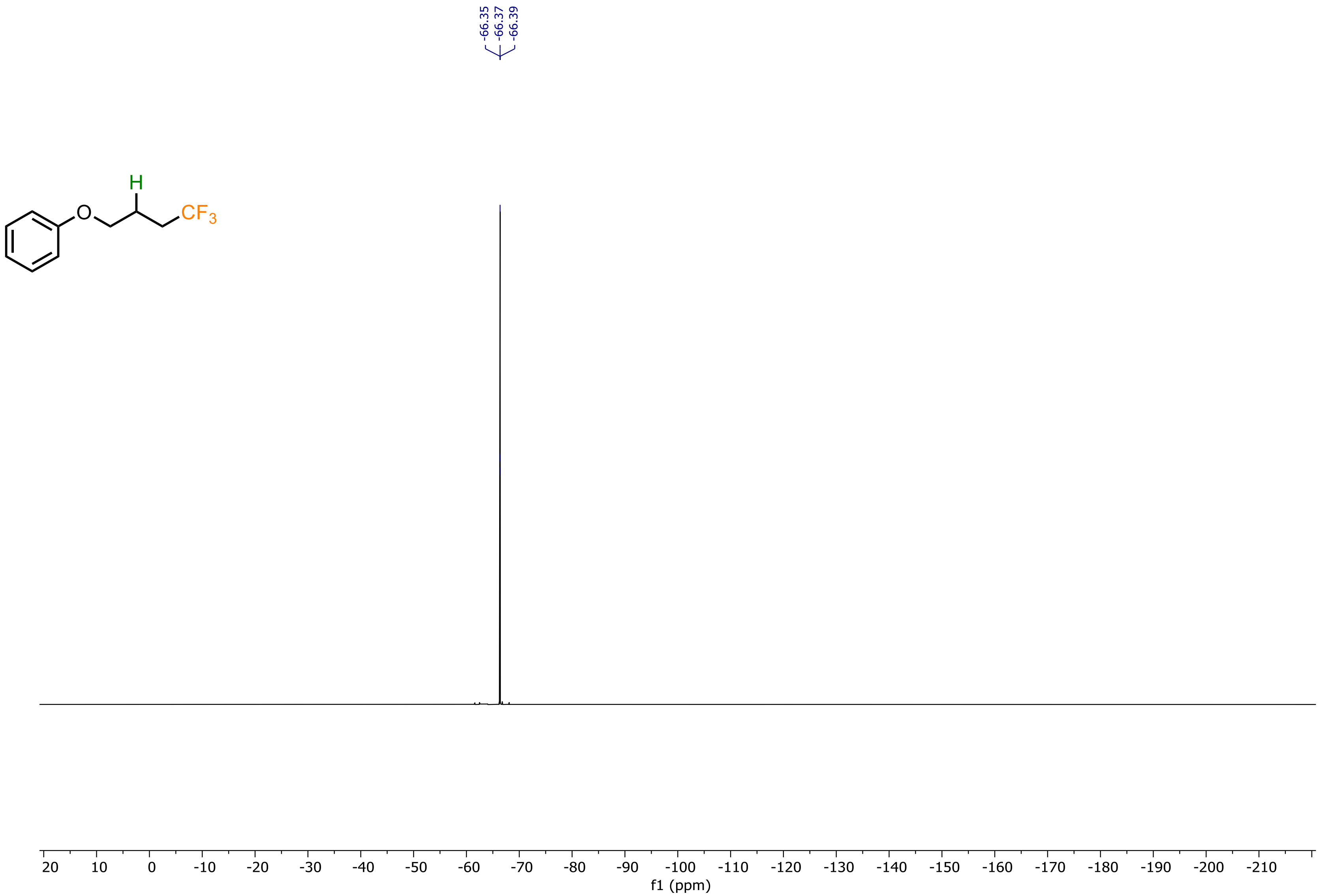

^{1}H NMR of 2-(9,9,9-Trifluorononyl)oxirane (8)

$CDCl_3$

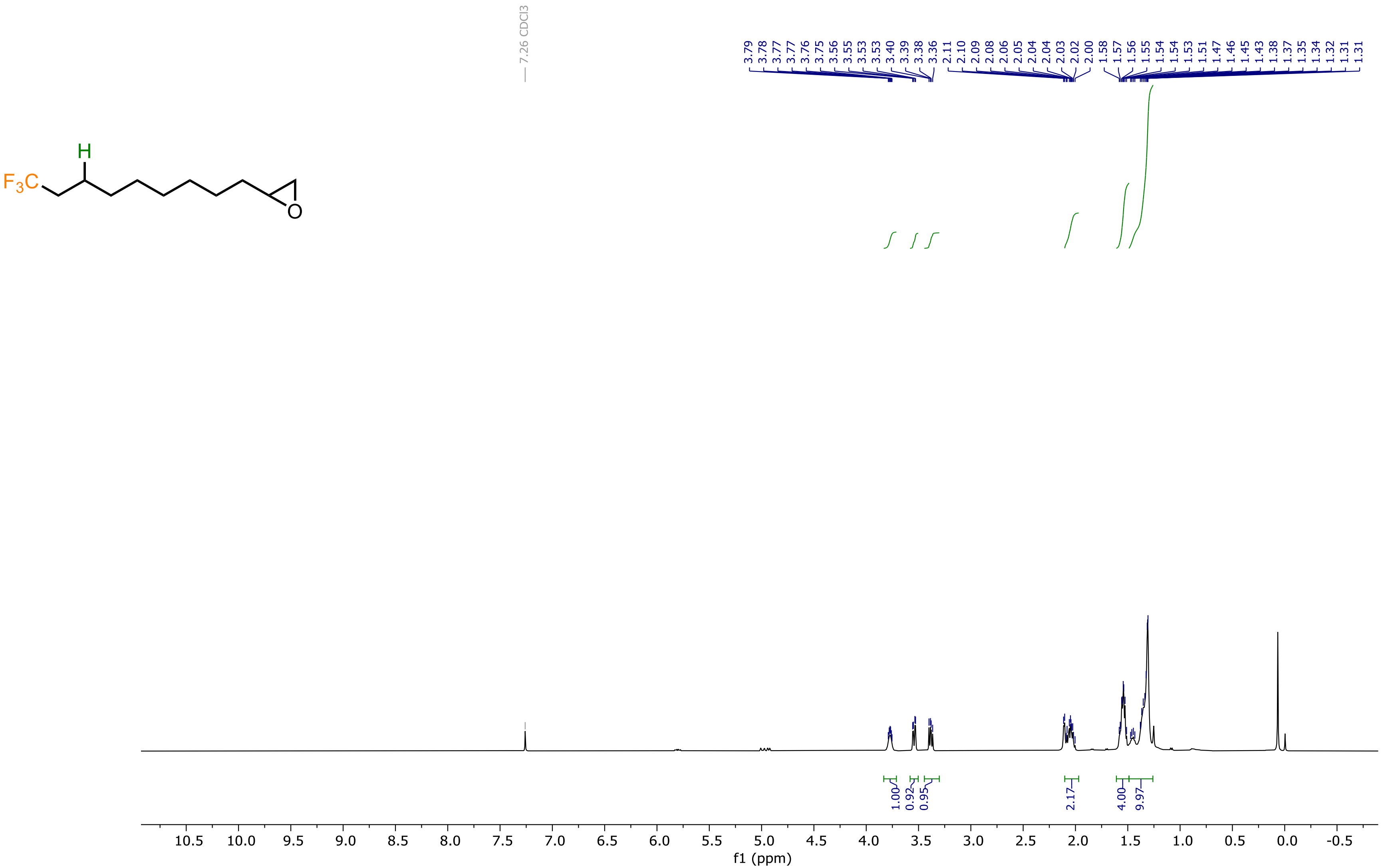

^{13}C NMR of 2-(9,9,9-Trifluorononyl)oxirane (8)

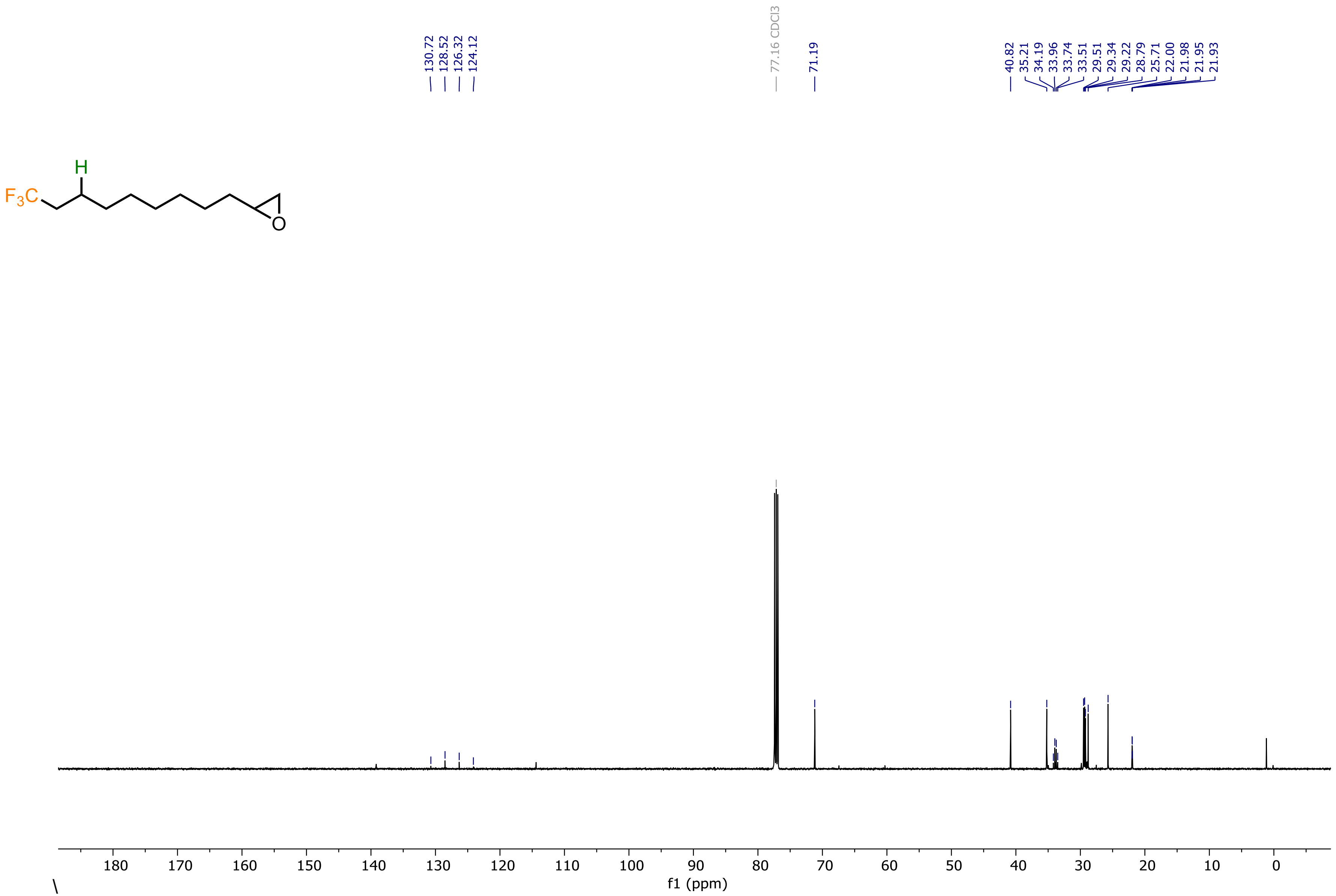

^{19}F NMR of 2-(9,9,9-Trifluorononyl)oxirane (8)

$CDCl_3$

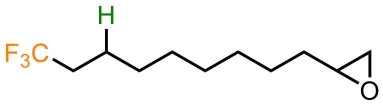

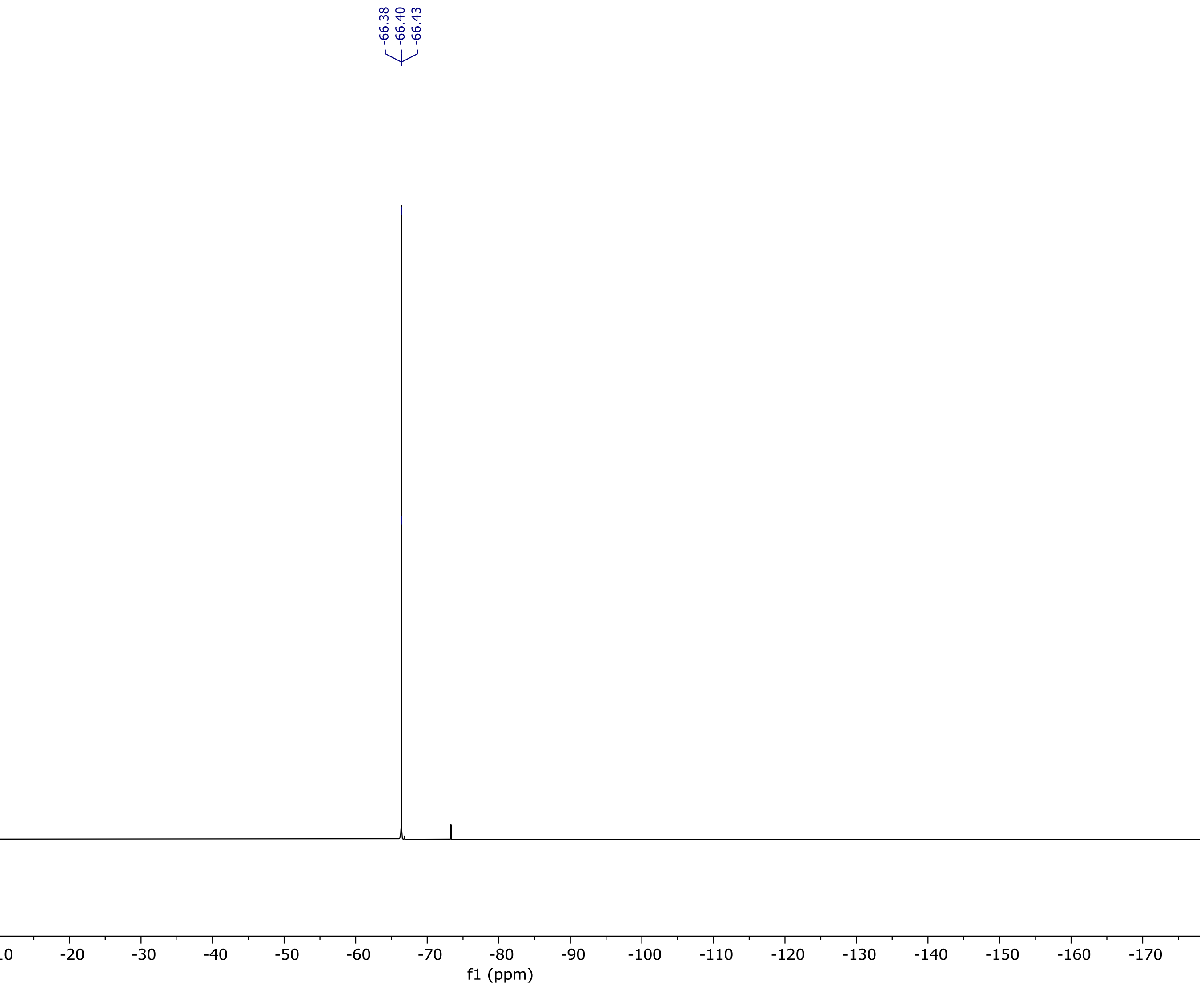

^1H NMR of 2-(4,4,4-trifluorobutyl)isoindoline-1,3-dione (9)

CD_3CN

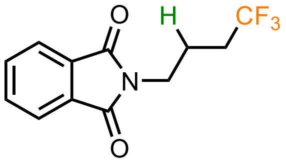

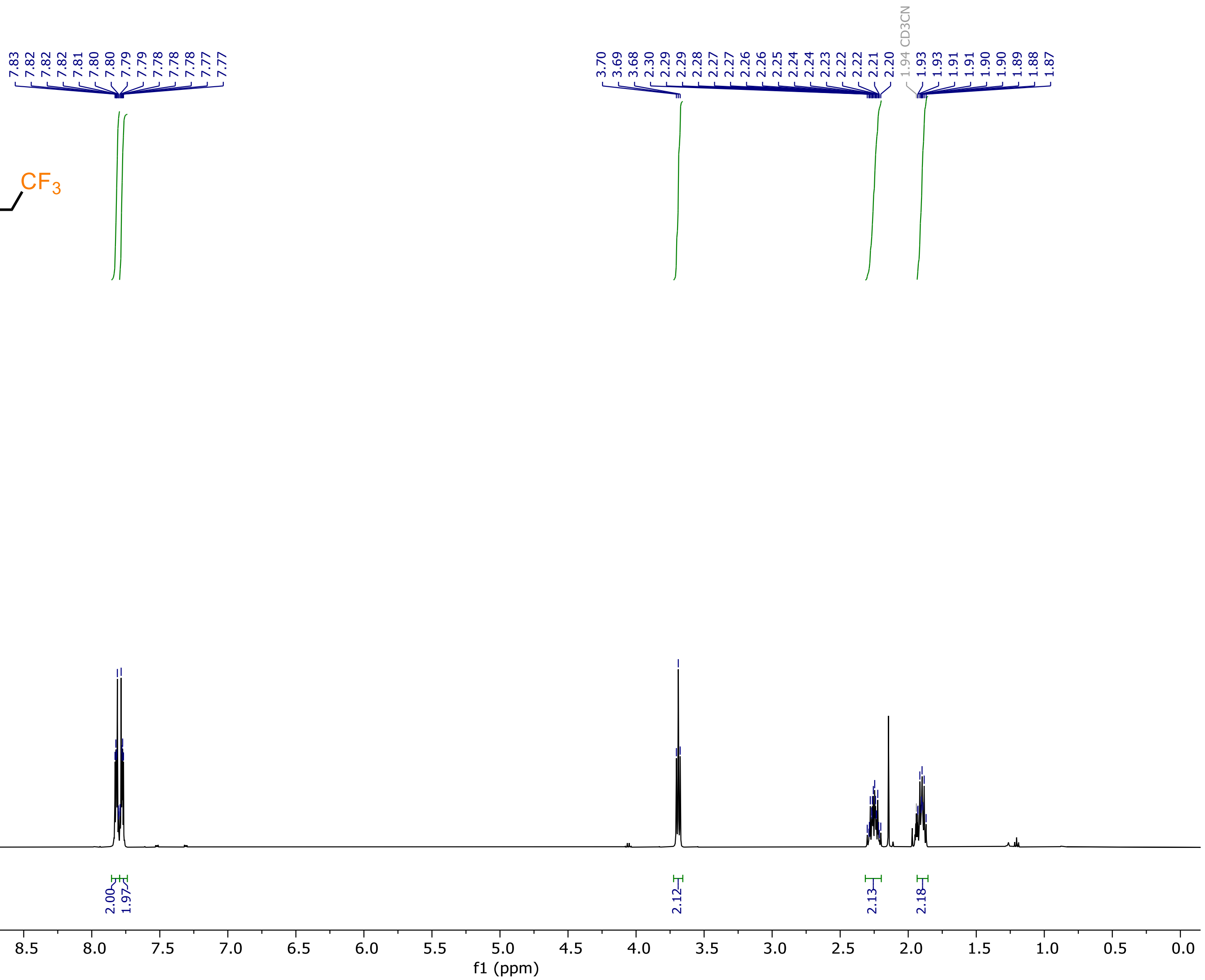

^{13}C NMR of 2-(4,4,4-trifluorobutyl)isoindoline-1,3-dione (9)

CD_3CN

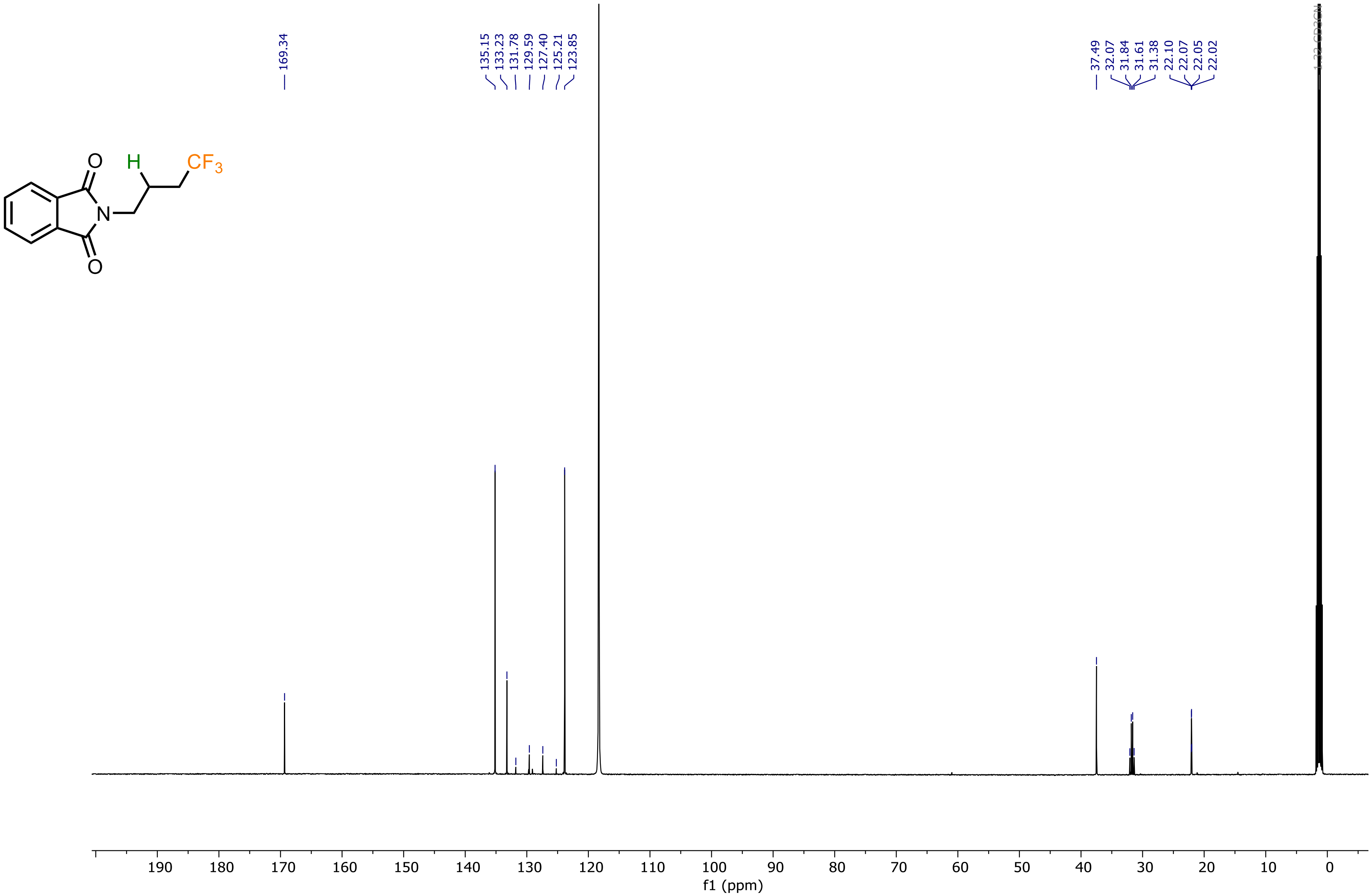

^{19}F NMR of 2-(4,4,4-trifluorobutyl)isoindoline-1,3-dione (9)

CD_3CN

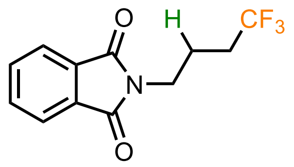

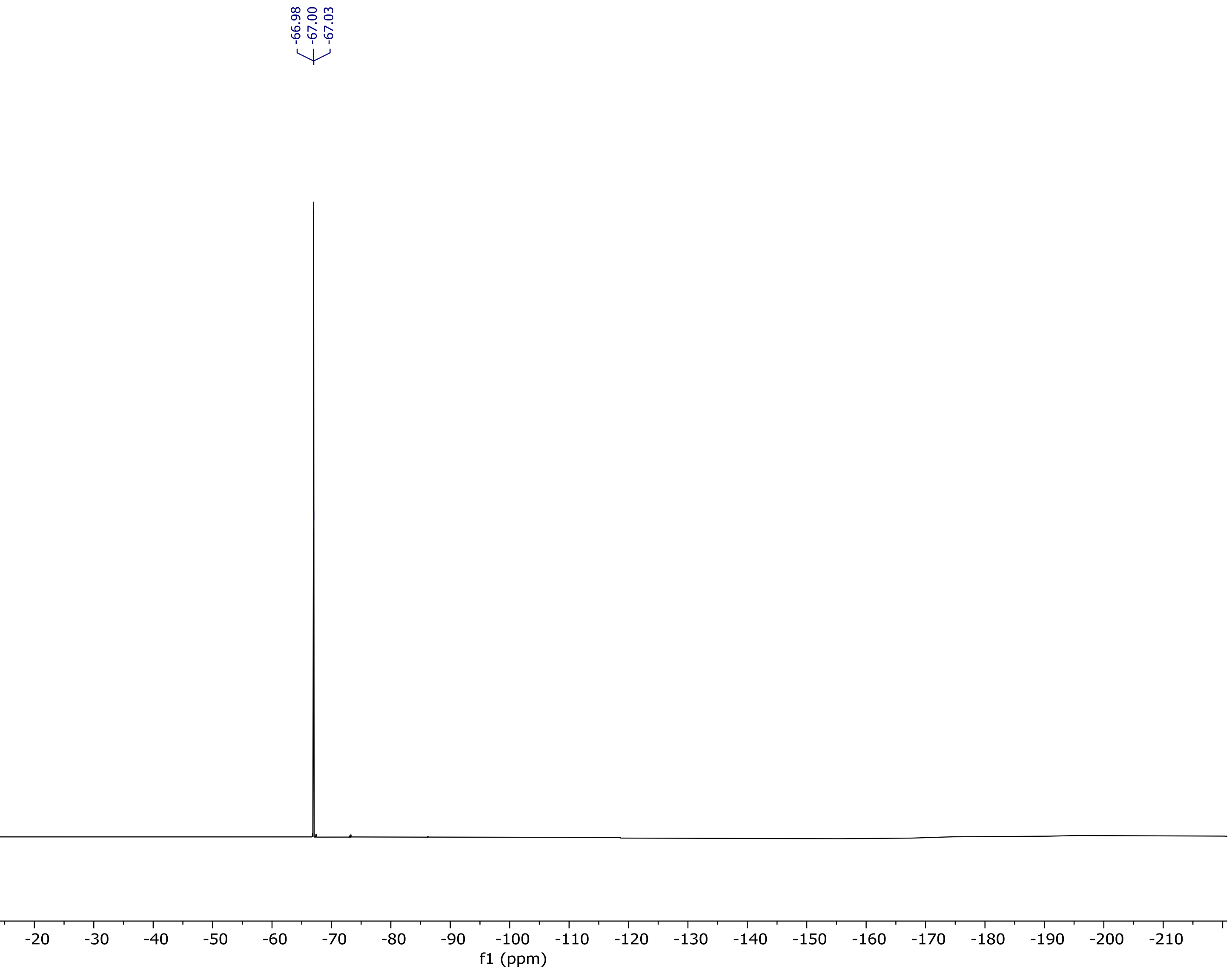

^{1}H NMR of nicotinic ester hydrotrifluoromethylated derivative (10)

CD_3CN

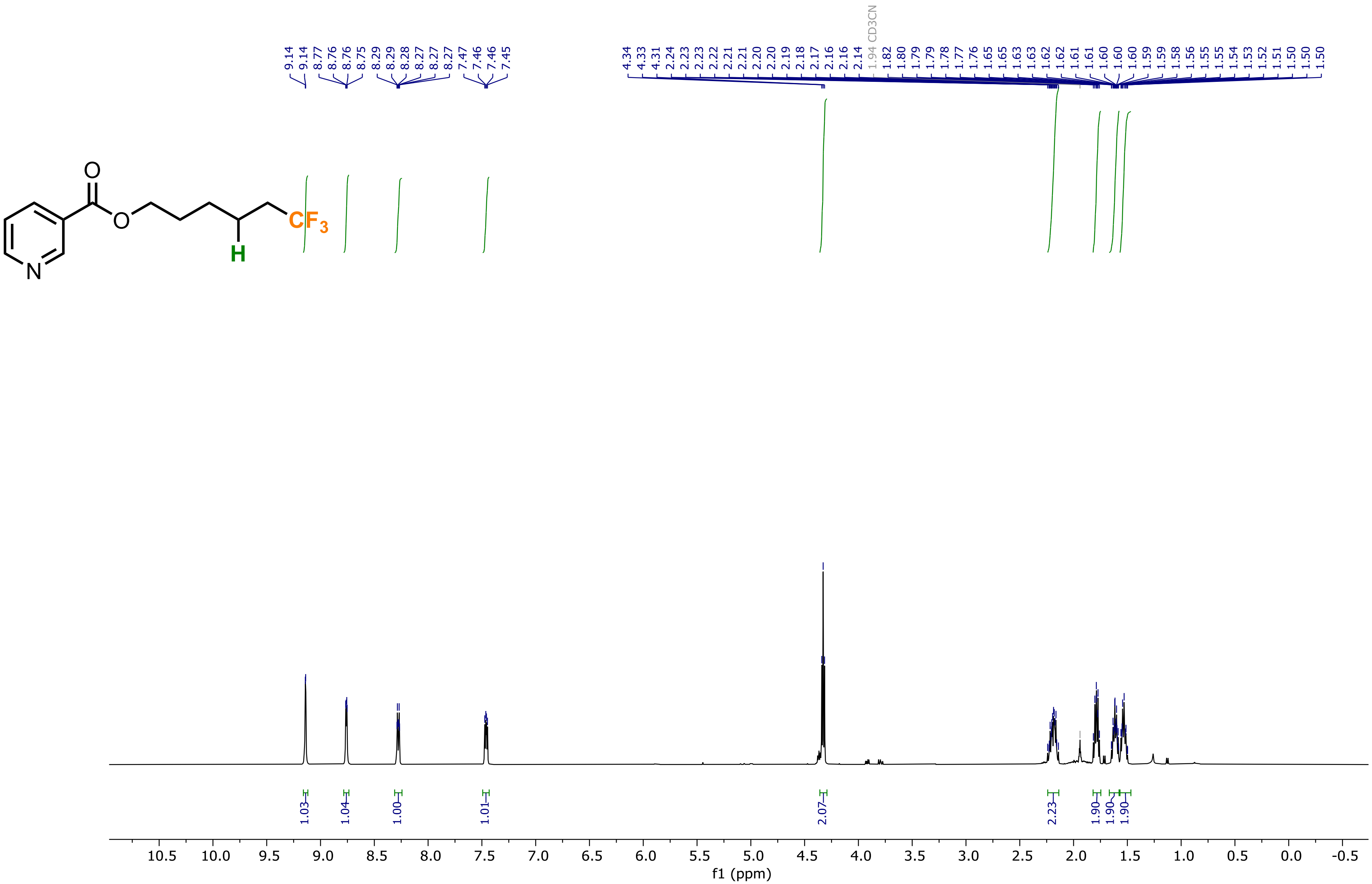

^{13}C NMR of nicotinic ester hydrotrifluoromethylated derivative (10)

CD_3CN

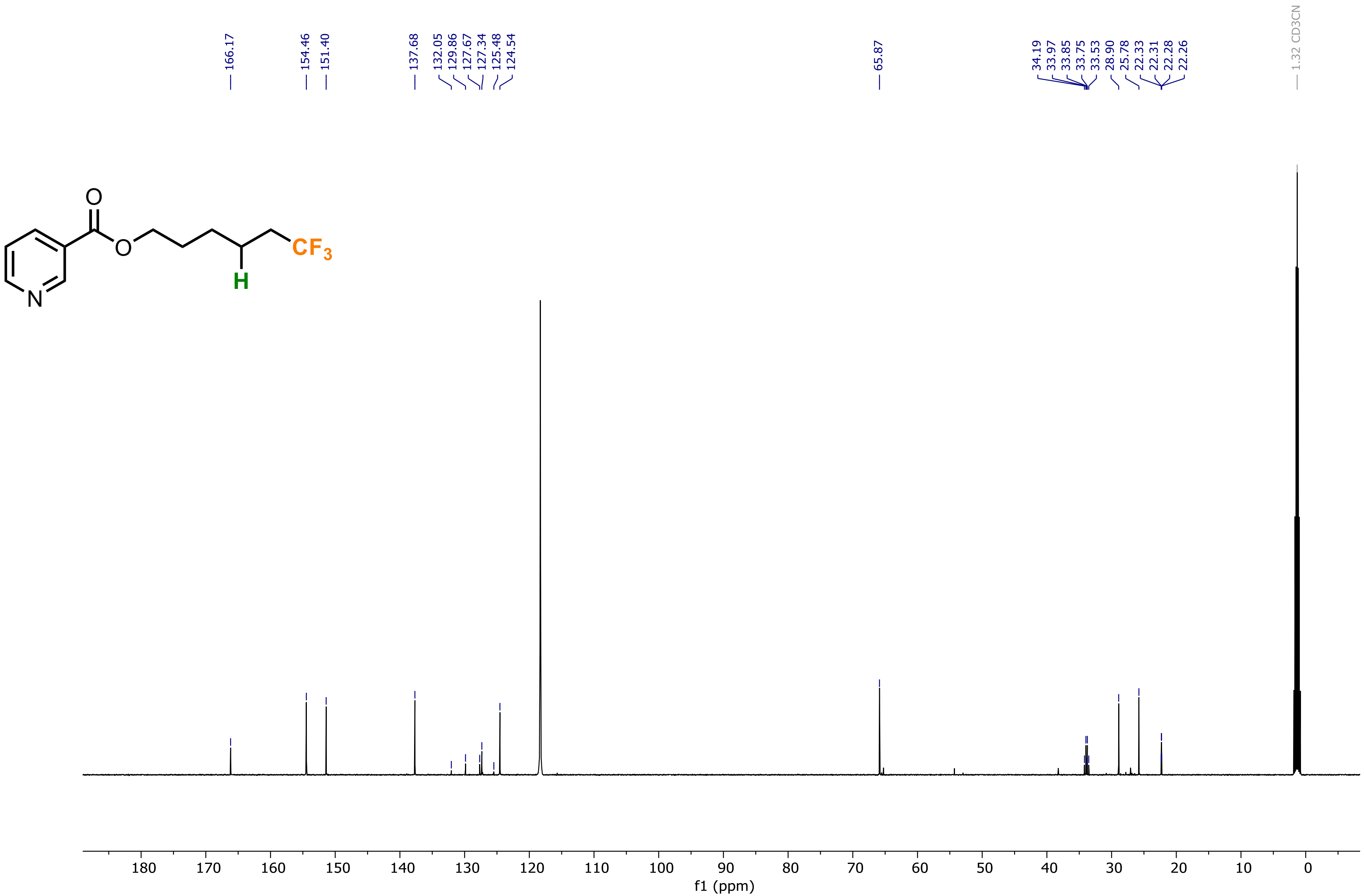

^{19}F NMR of nicotinic ester hydrotrifluoromethylated derivative (10)

$CDCl_3$

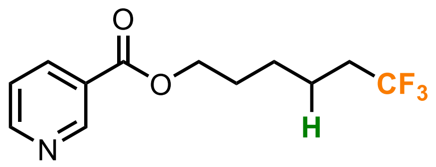

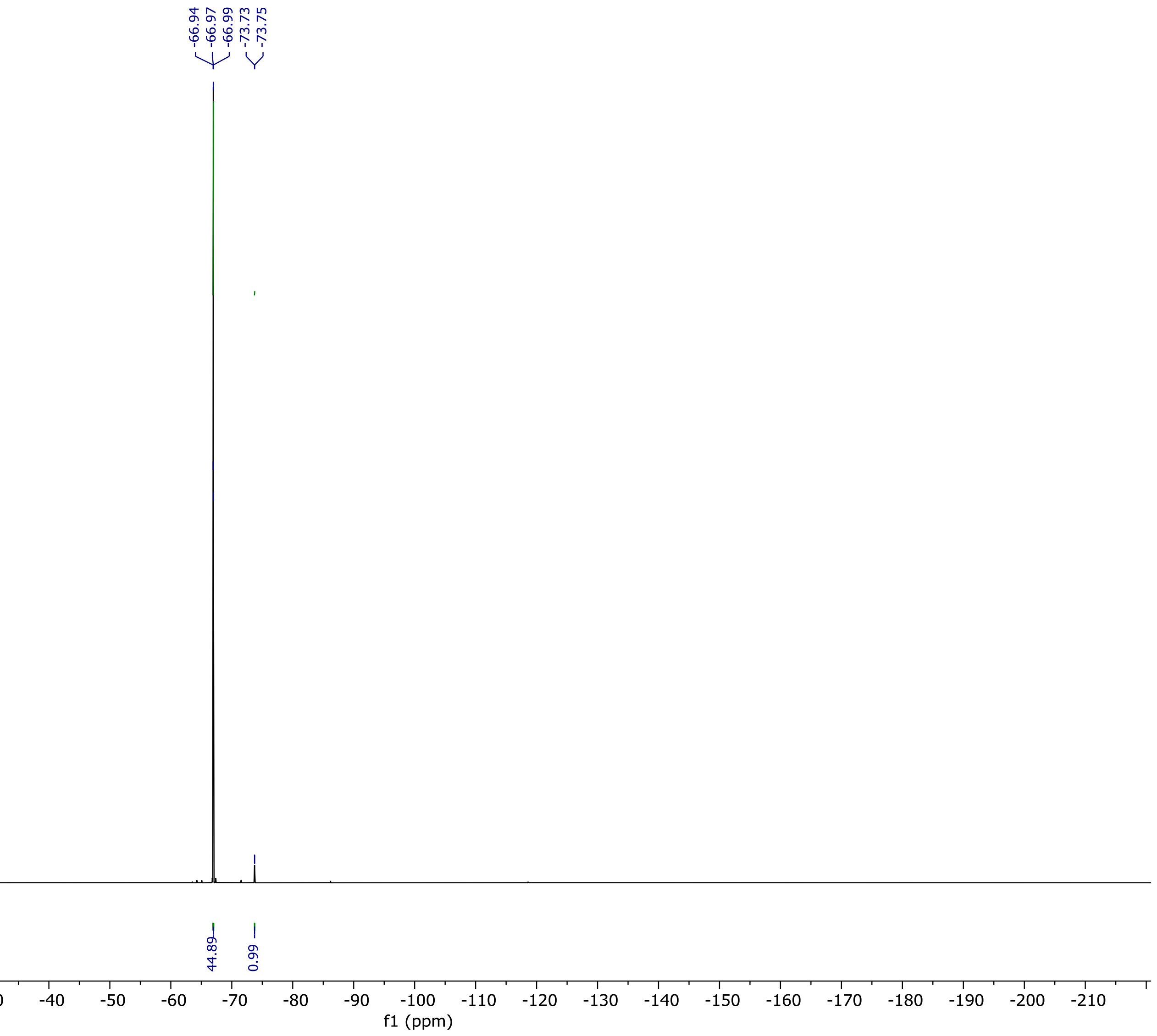

^{1}H NMR of 5,5,5-trifluoropentyl 1-benzoylpiperidine-4-carboxylate (11)

$CDCl_3$

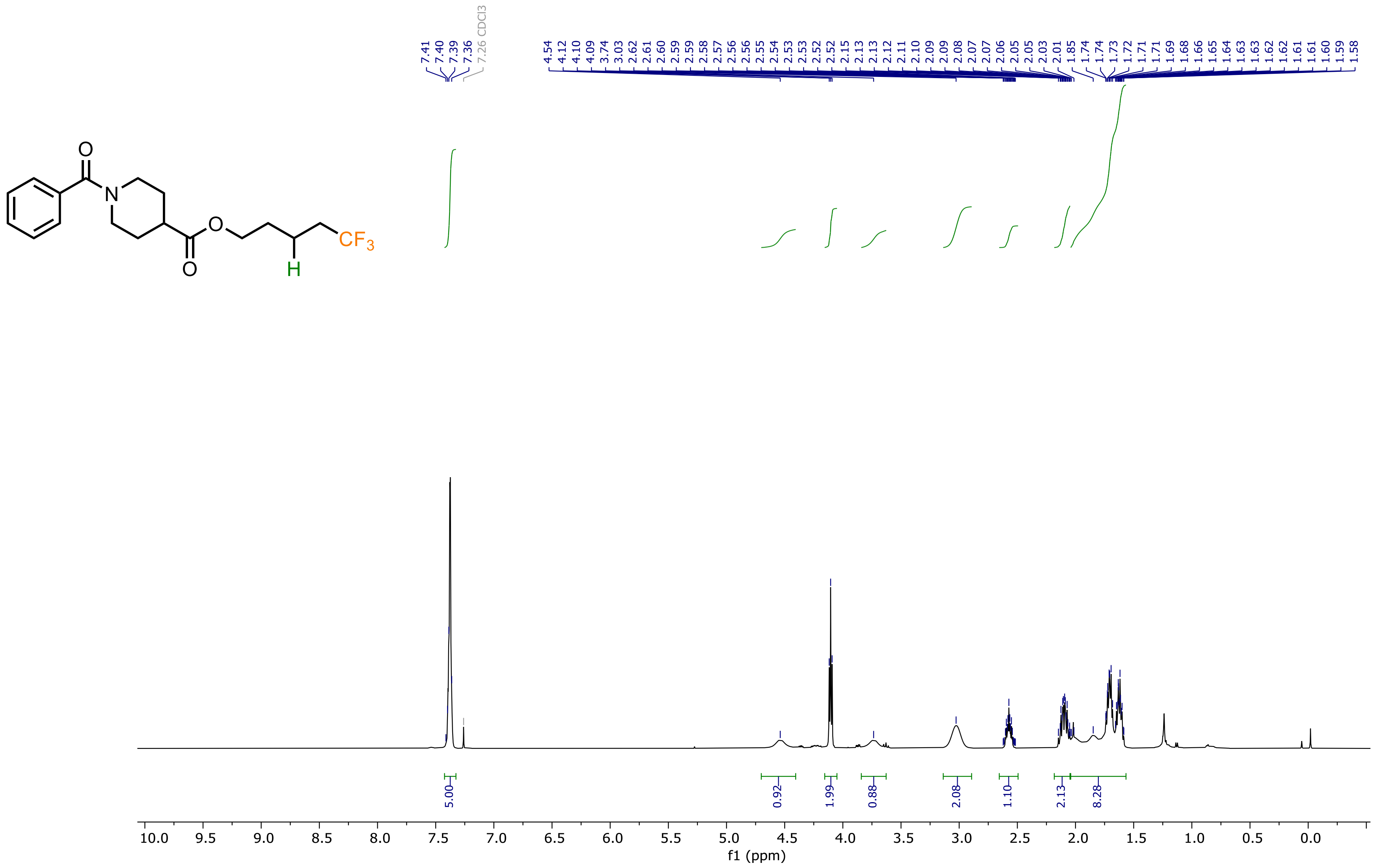

^{13}C NMR of 5,5,5-trifluoropentyl 1-benzoylpiperidine-4-carboxylate (11)

CDCl3

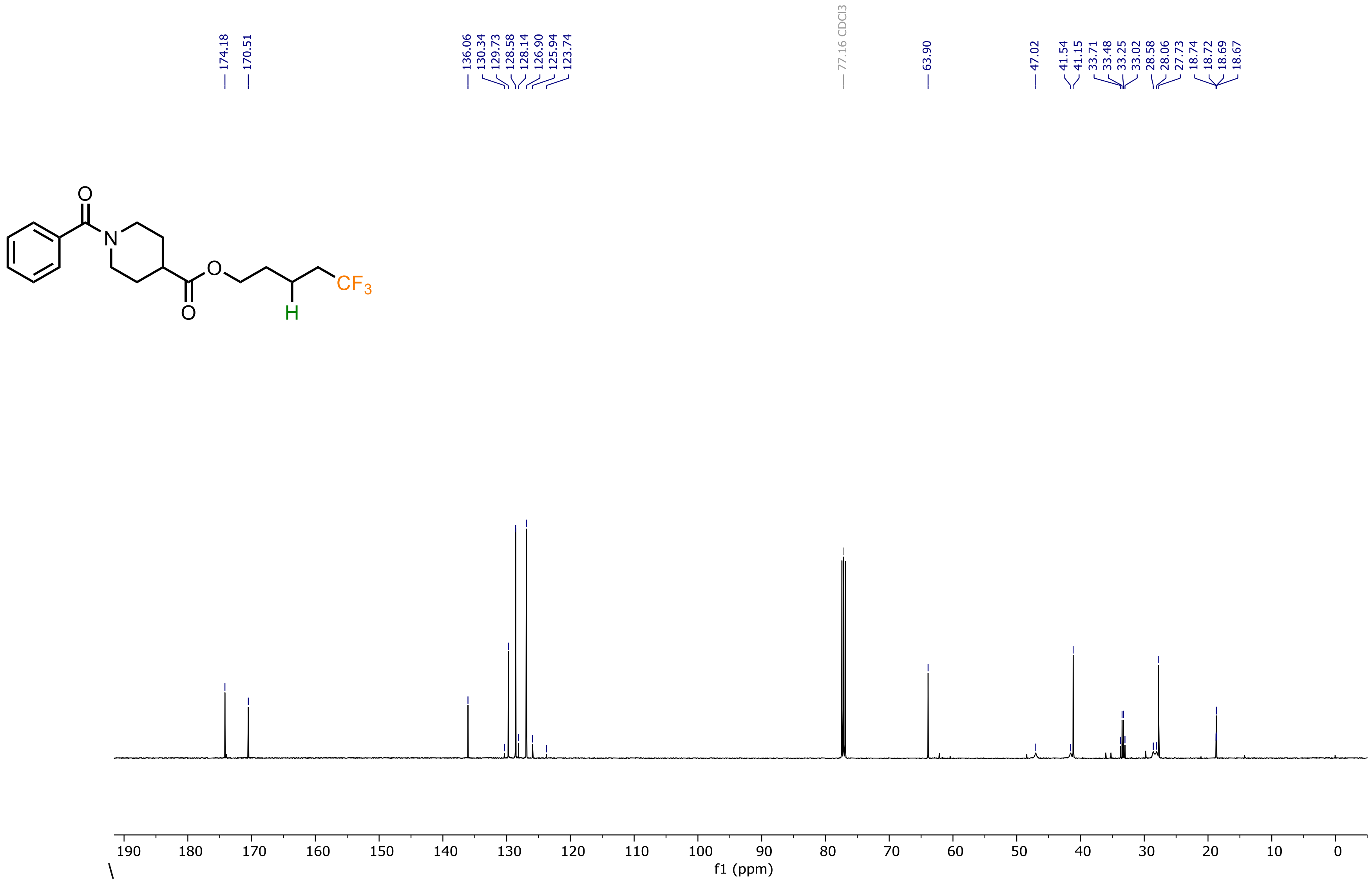

^{19}F NMR of 5,5,5-trifluoropentyl 1-benzoylpiperidine-4-carboxylate (11)

$CDCl_3$

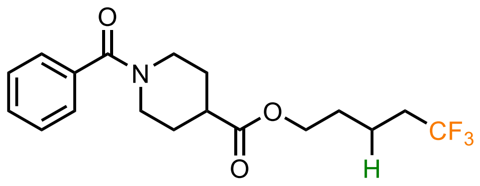

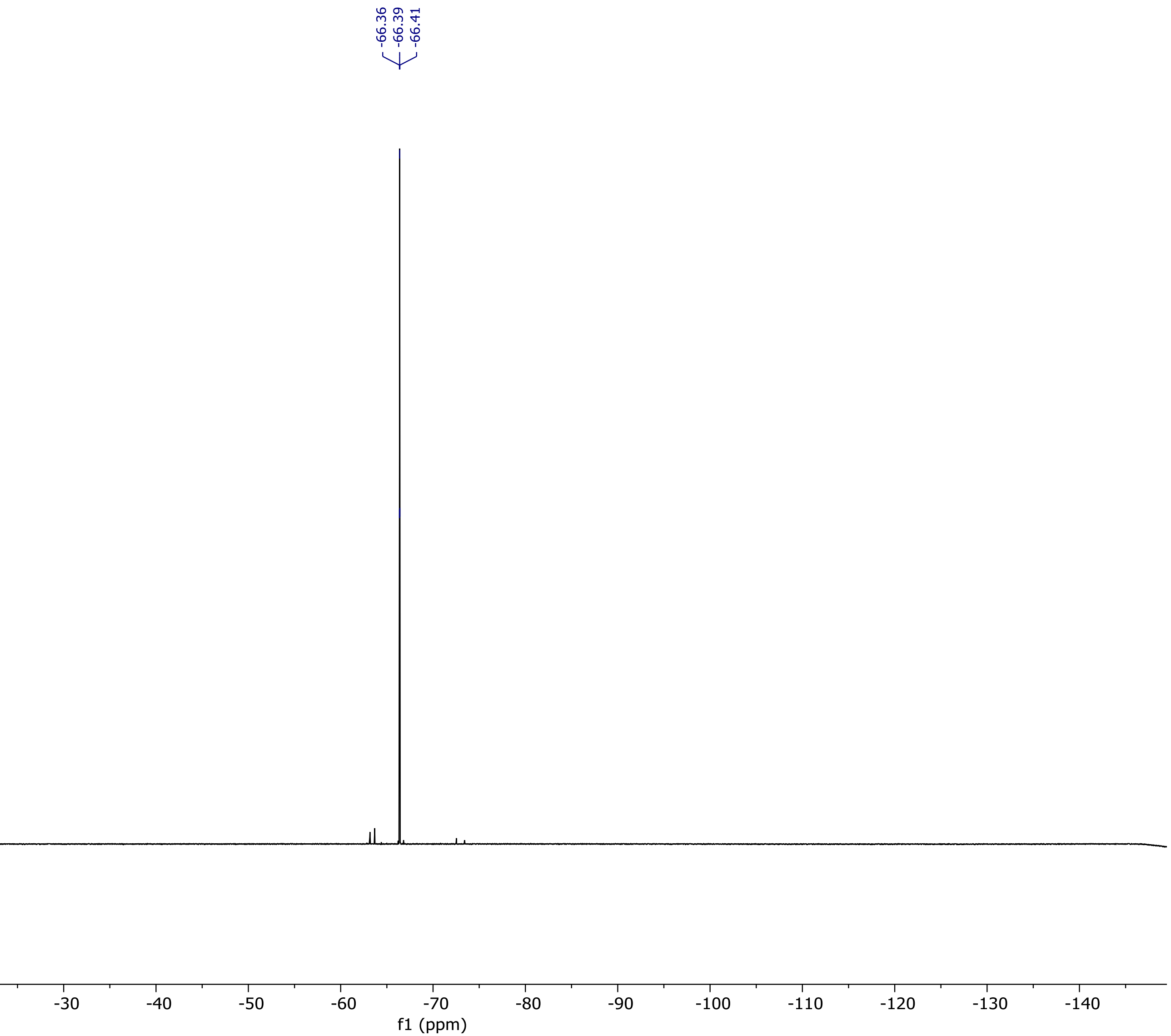

^{1}H NMR of 2-methoxy-4-(4,4,4-trifluorobutyl)phenol (12)

$CDCl_3$

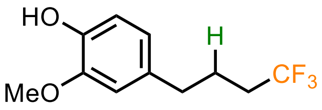

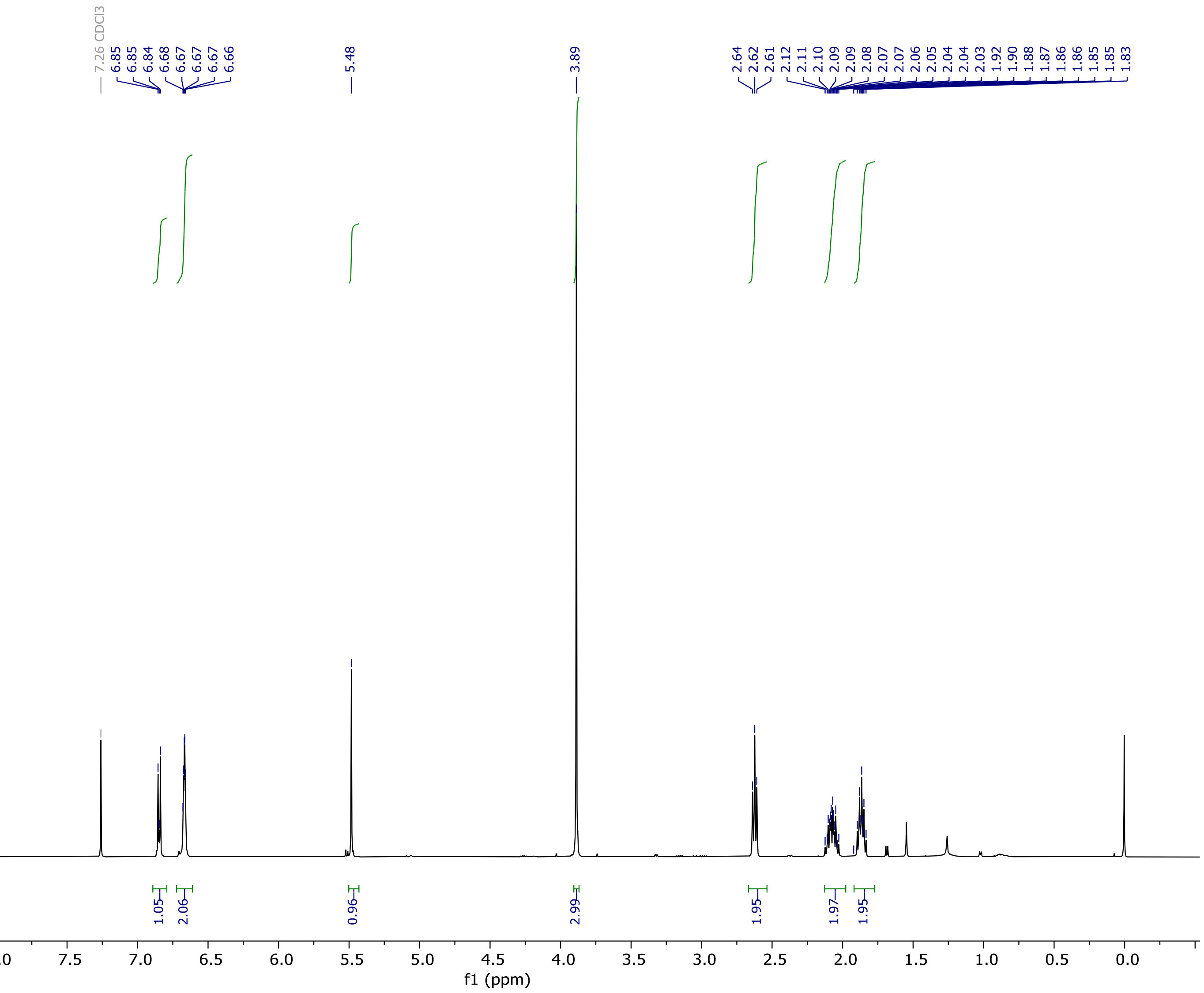

^{19}F NMR of 2-methoxy-4-(4,4,4-trifluorobutyl)phenol (12)

CD_3CN

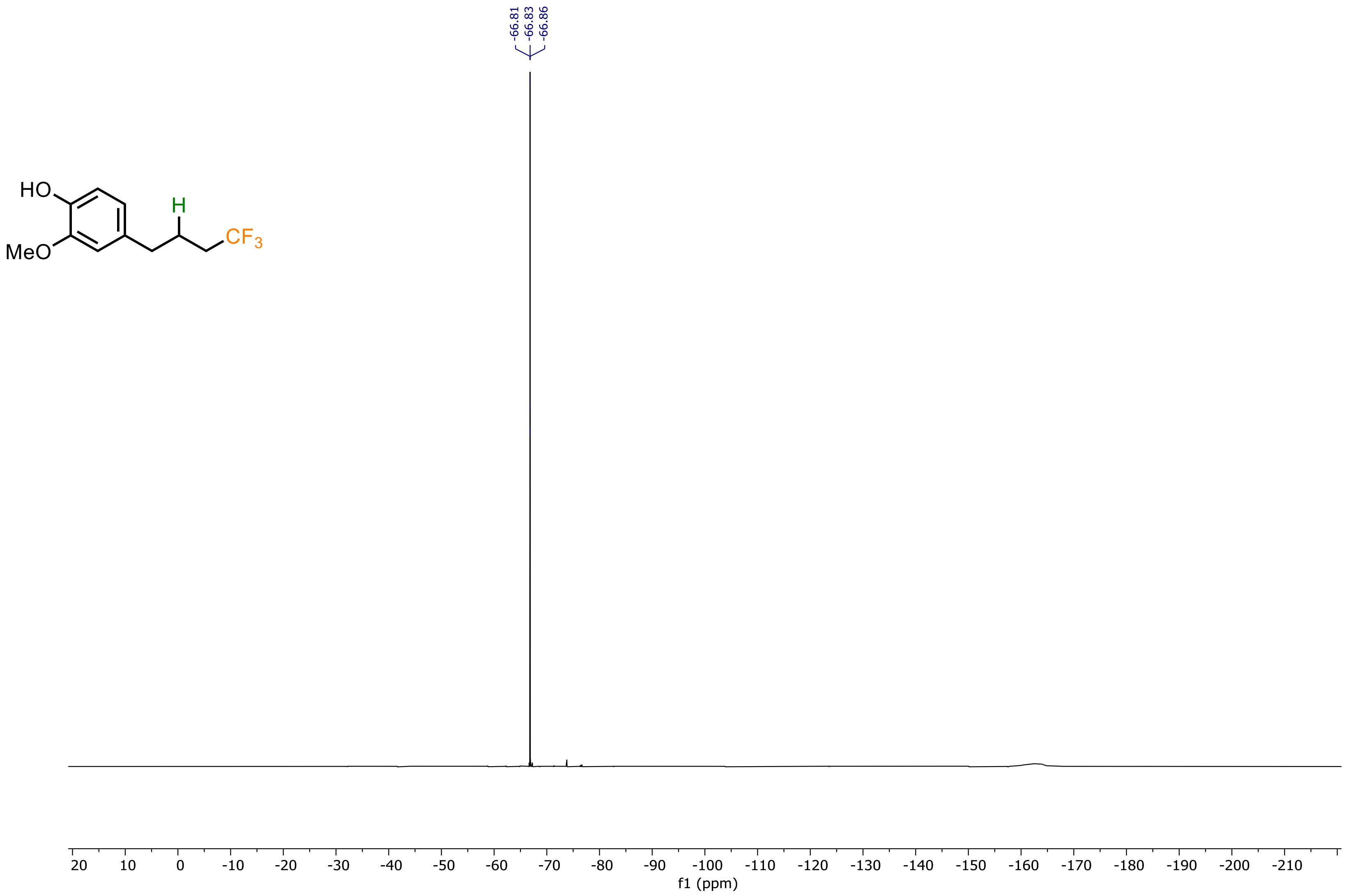

^{13}C NMR of 2-methoxy-4-(4,4,4-trifluorobutyl)phenol (12)

$CDCl_3$

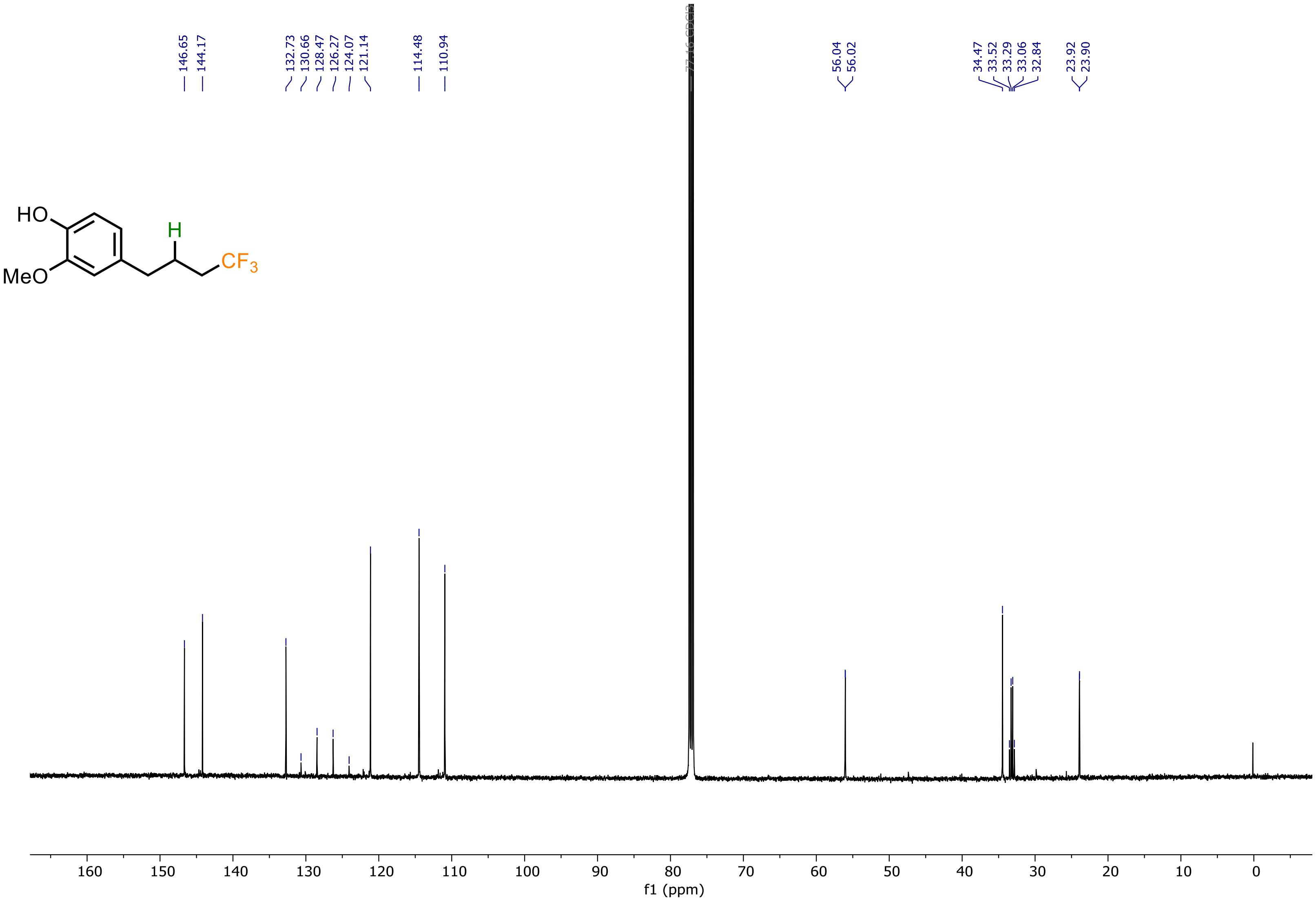

^{1}H NMR of 2-(2H-benzo[d][1,2,3]triazol-2-yl)-4-methyl-6-(4,4,4-trifluorobutyl)phenol (13)

$CDCl_3$

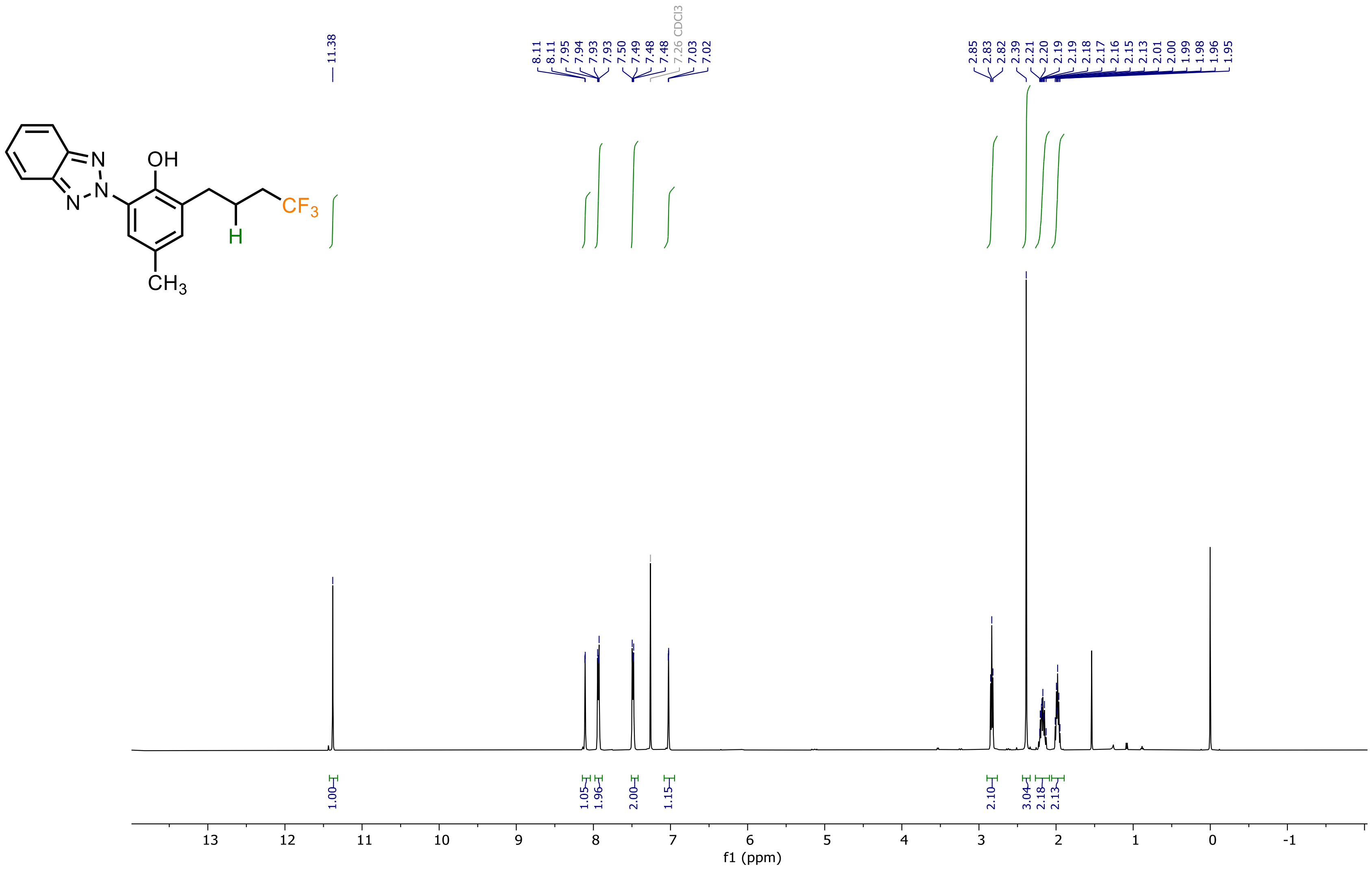

^{13}C NMR of 2-(2H-benzo[d][1,2,3]triazol-2-yl)-4-methyl-6-(4,4,4-trifluorobutyl)phenol (13)

CDCl3

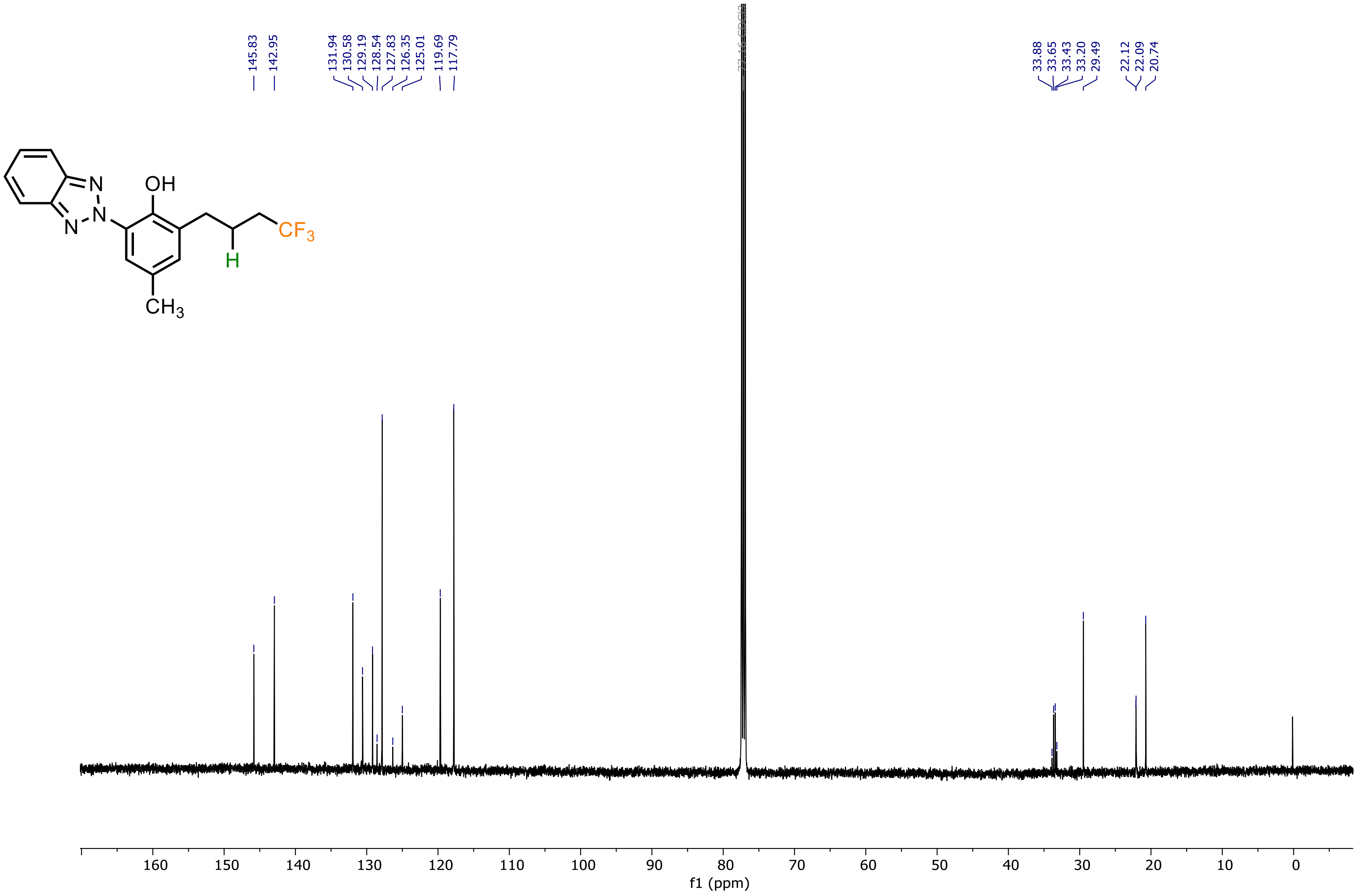

^{19}F NMR of 2-(2H-benzo[d][1,2,3]triazol-2-yl)-4-methyl-6-(4,4,4-trifluorobutyl)phenol (13)

$CDCl_3$

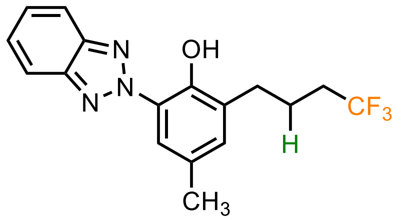

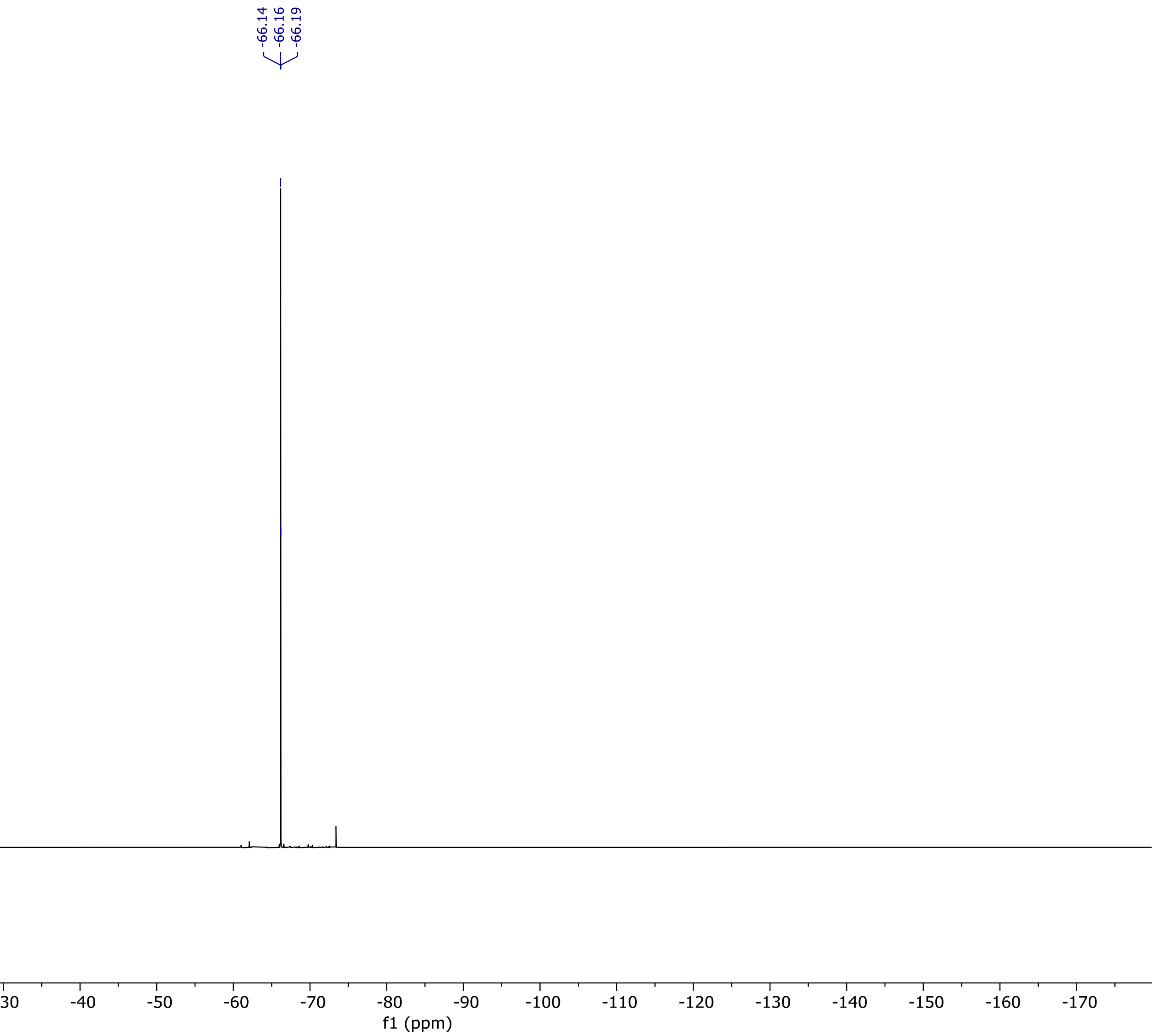

^{1}H NMR of ethyl 4-((6,6,6-trifluorohexanoyl)oxy)benzoate (14)

$CDCl_3$

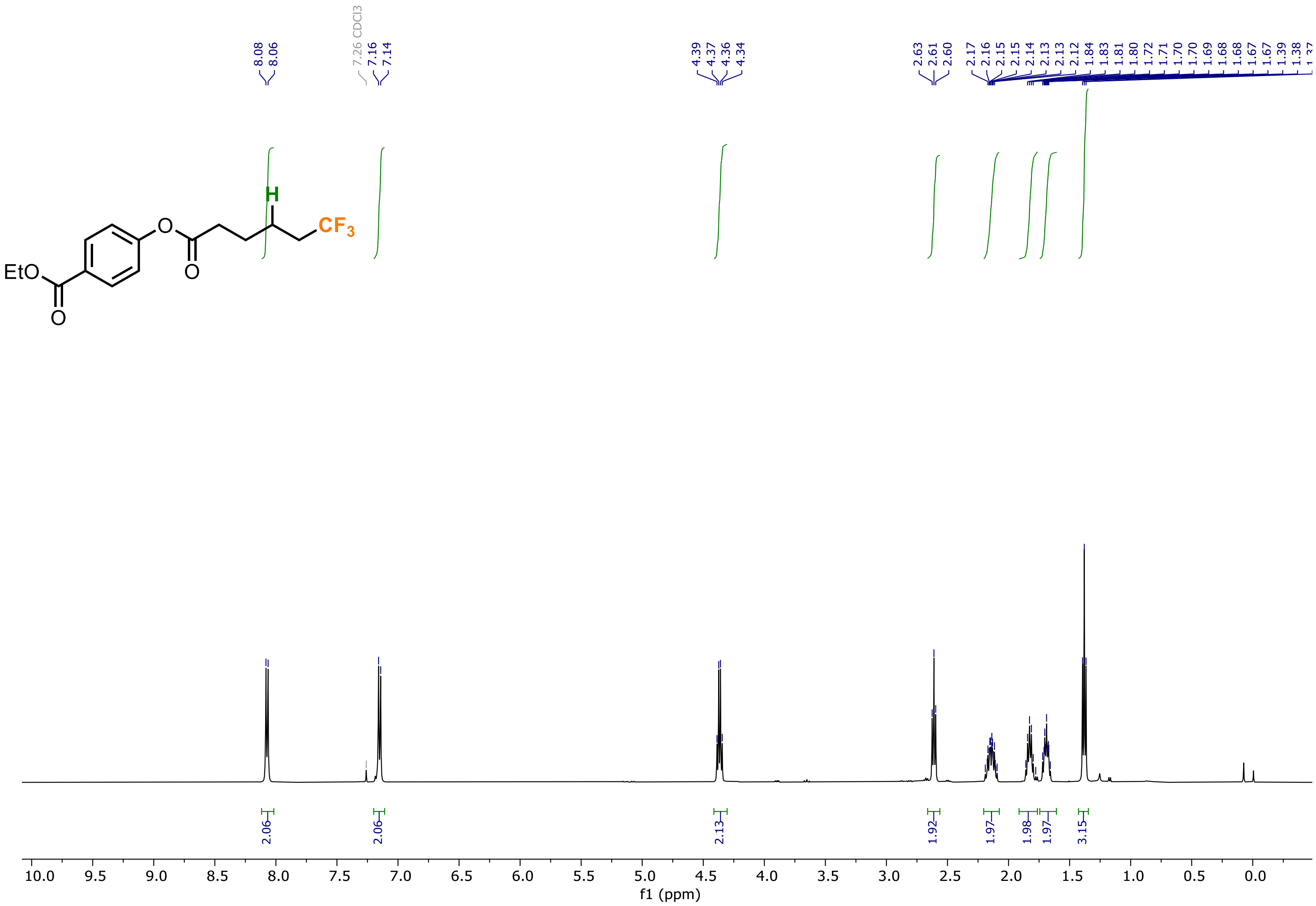

^{13}C NMR of ethyl 4-((6,6,6-trifluorohexanoyl)oxy)benzoate (14)

$CDCl_3$

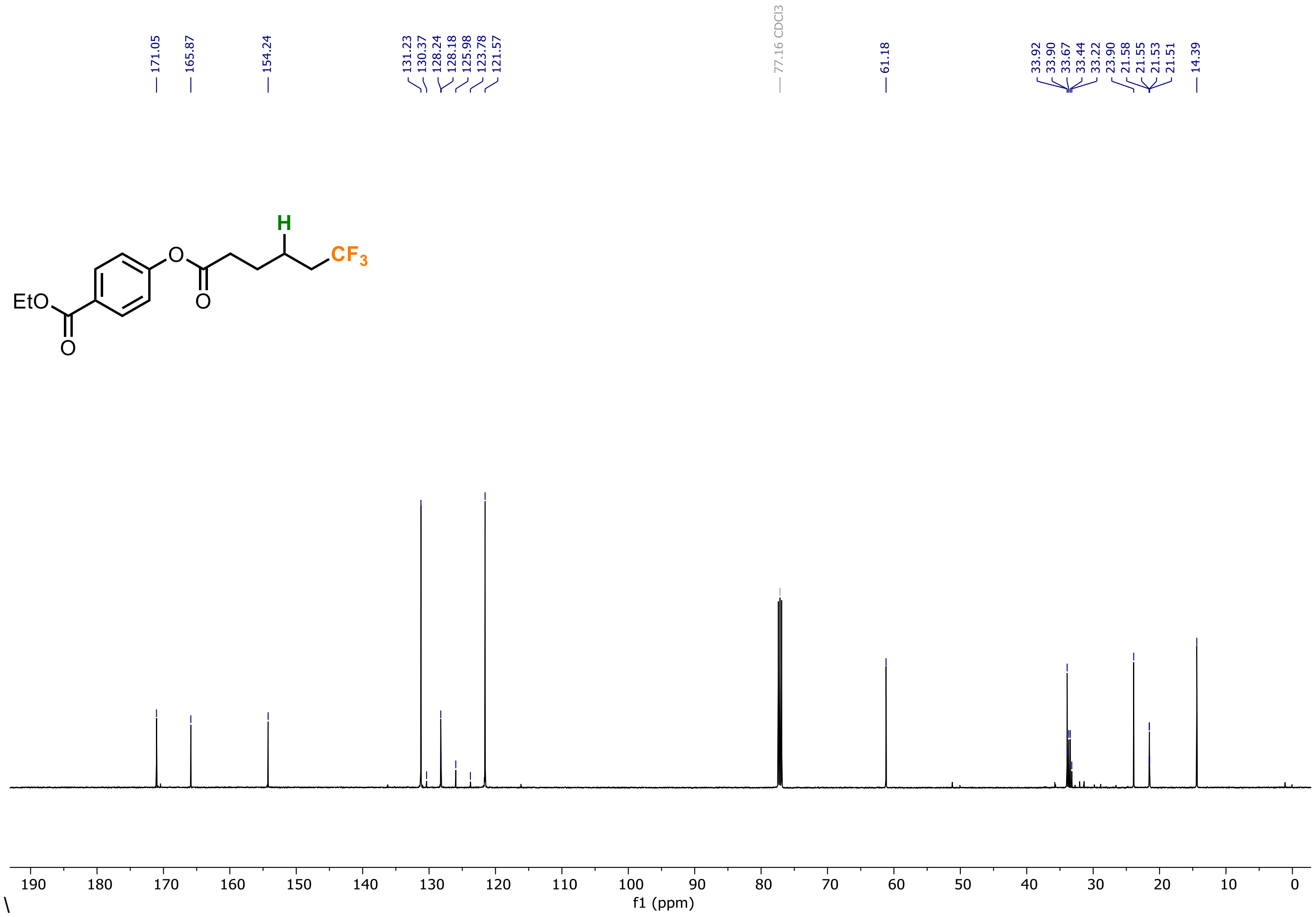

^{19}F NMR of ethyl 4-((6,6,6-trifluorohexanoyl)oxy)benzoate (14)

CDCl3

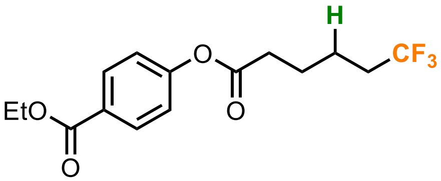

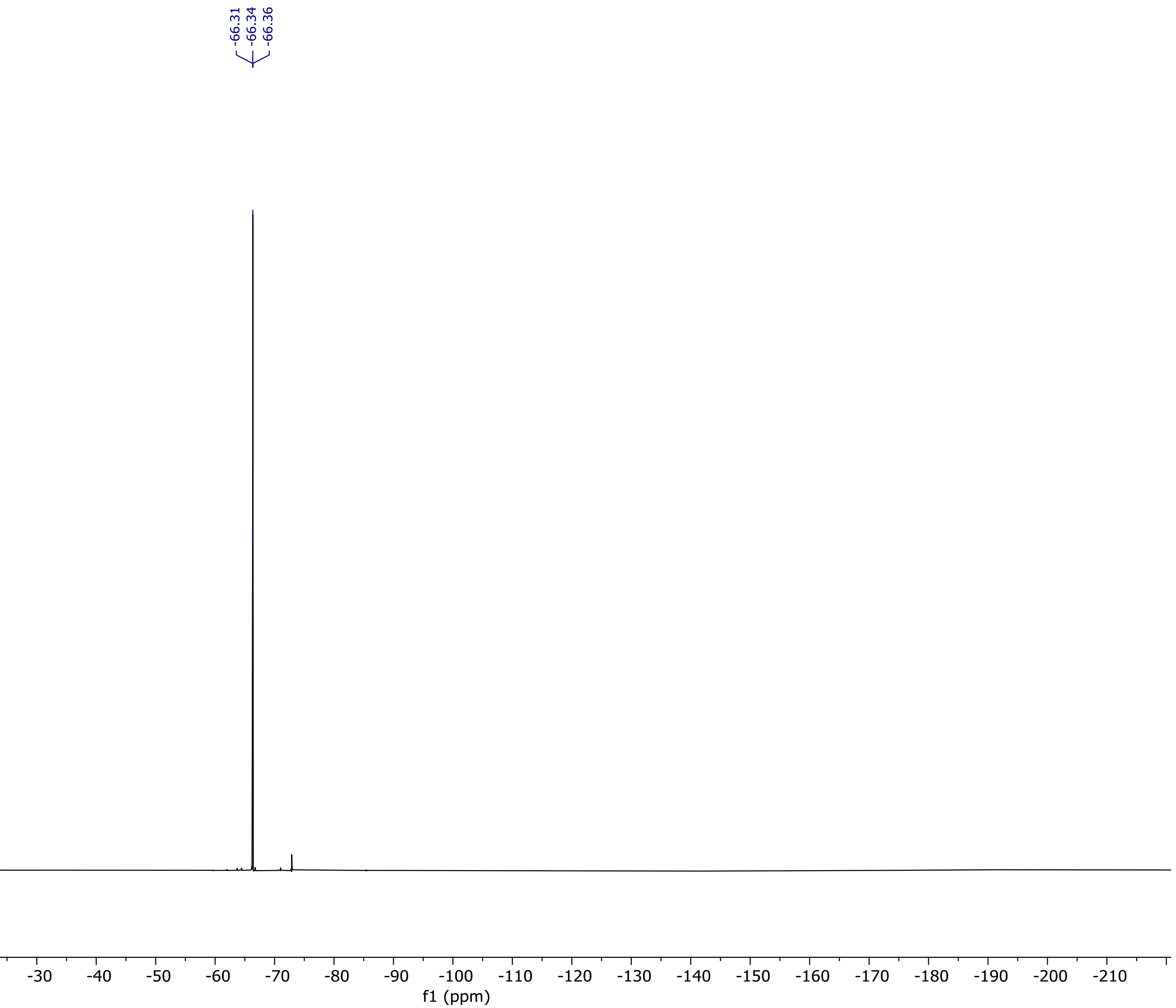

^{1}H NMR of febuxostat hydrotrifluoromethylated derivative (18)

$CDCl_3$

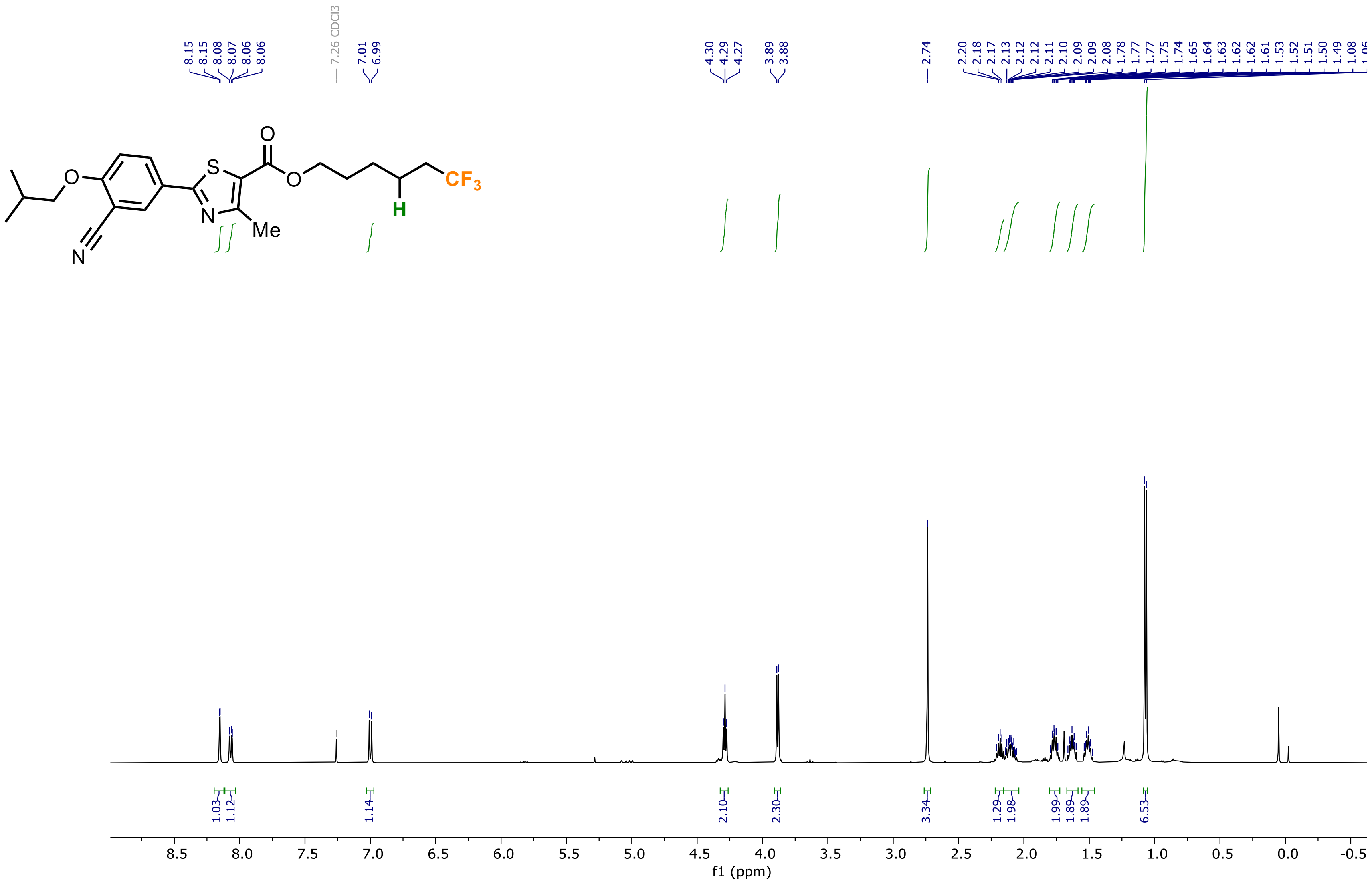

^{13}C NMR of febuxostat hydrotrifluoromethylated derivative (18)

$CDCl_3$

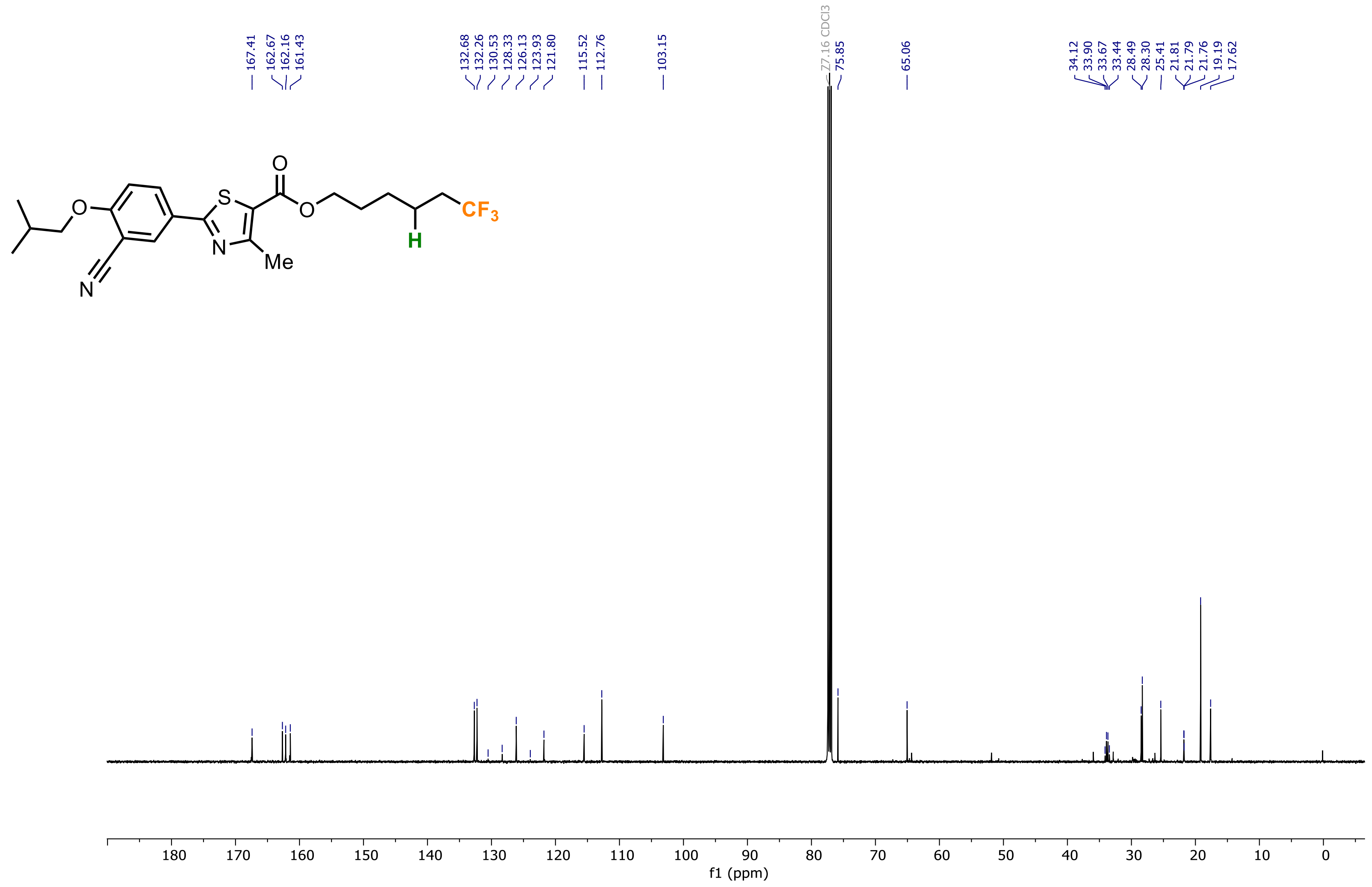

^{19}F NMR of febuxostat hydrotrifluoromethylated derivative (18)

CD_3CN

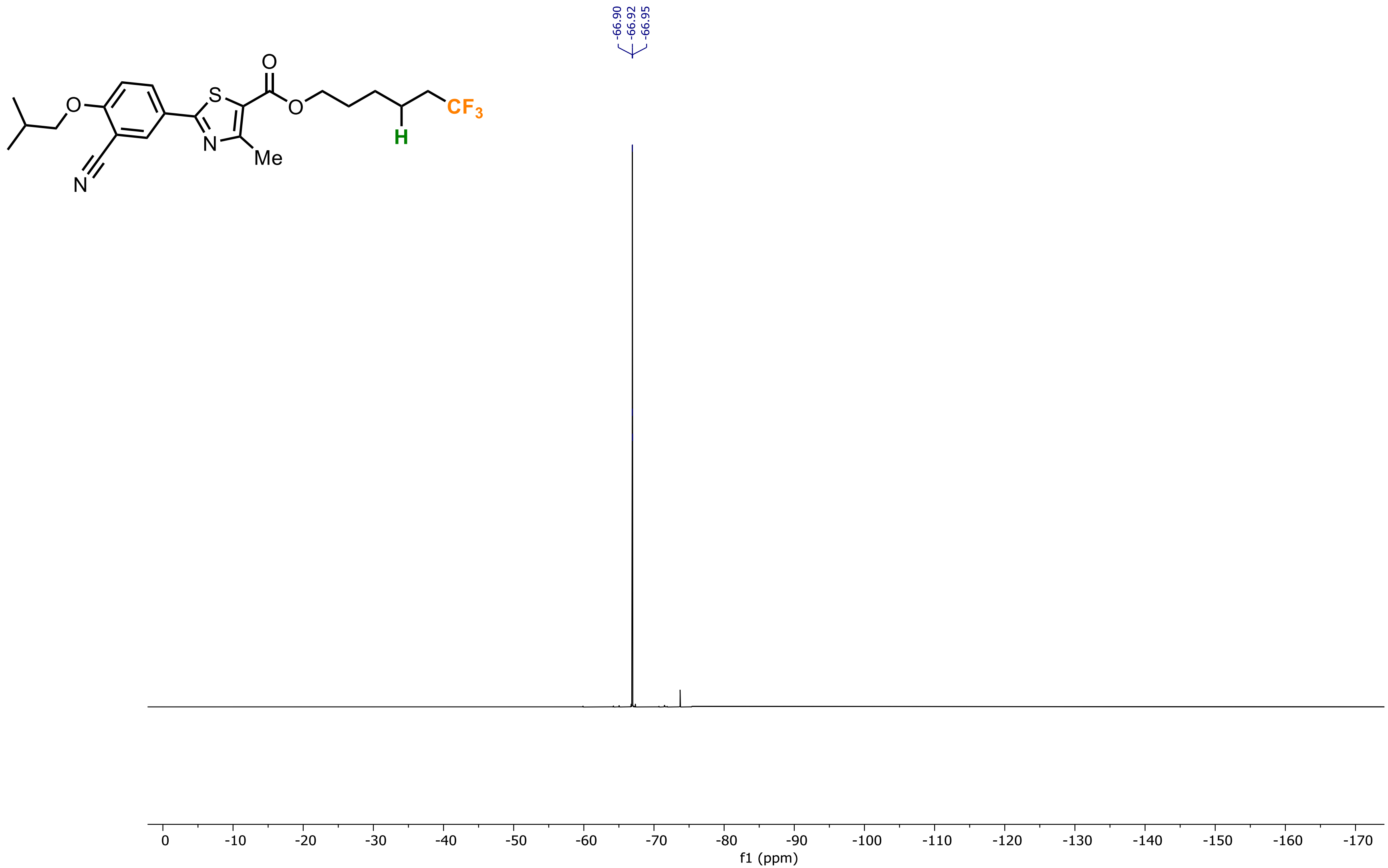

^{1}H NMR of quinine hydrotrifluoromethylated derivative (19)

$CDCl_3$

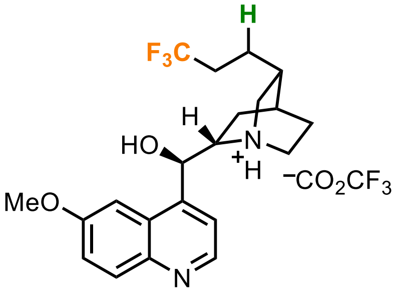

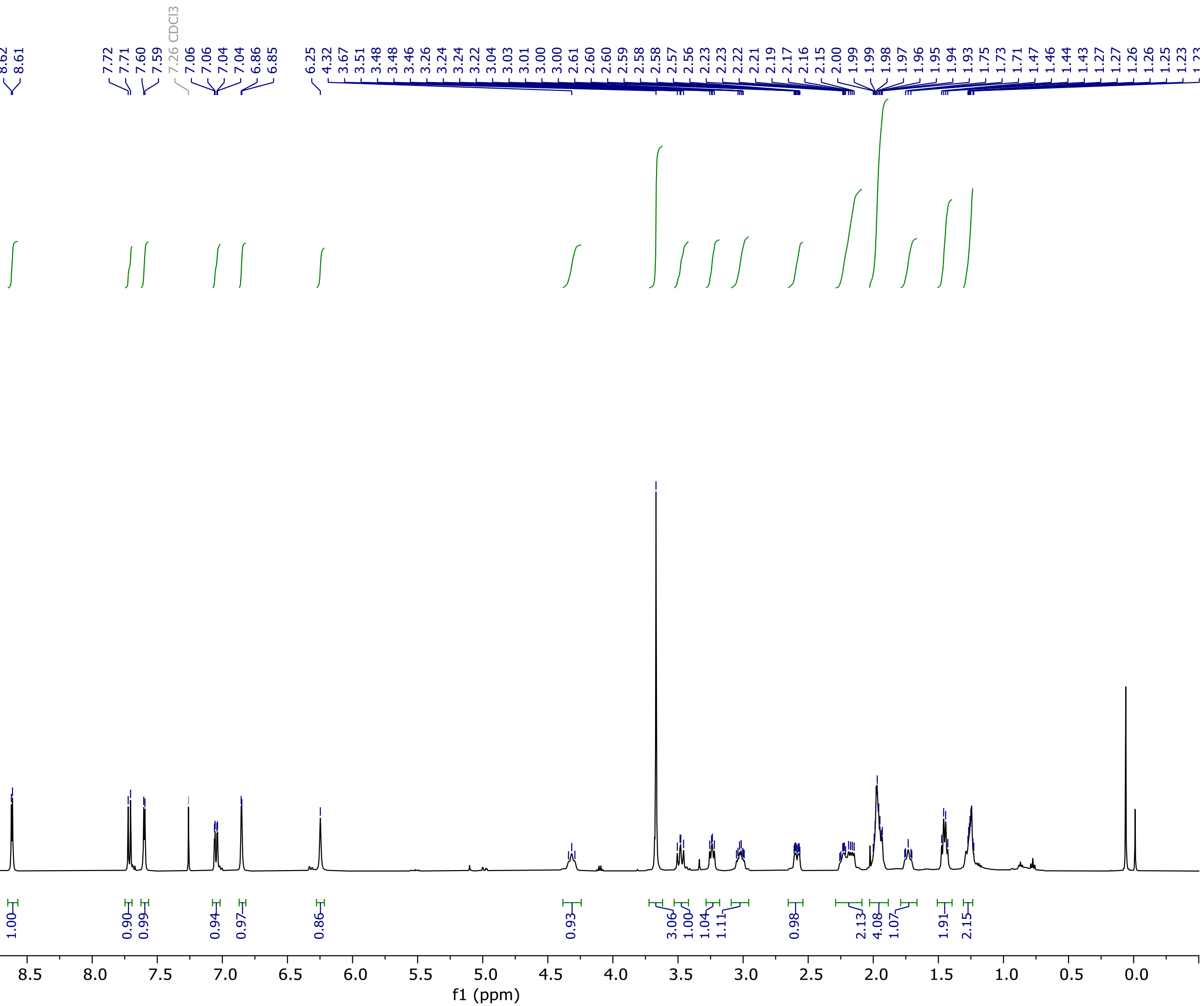

^{13}C NMR of quinine hydrotrifluoromethylated derivative (19)

$CDCl_3$

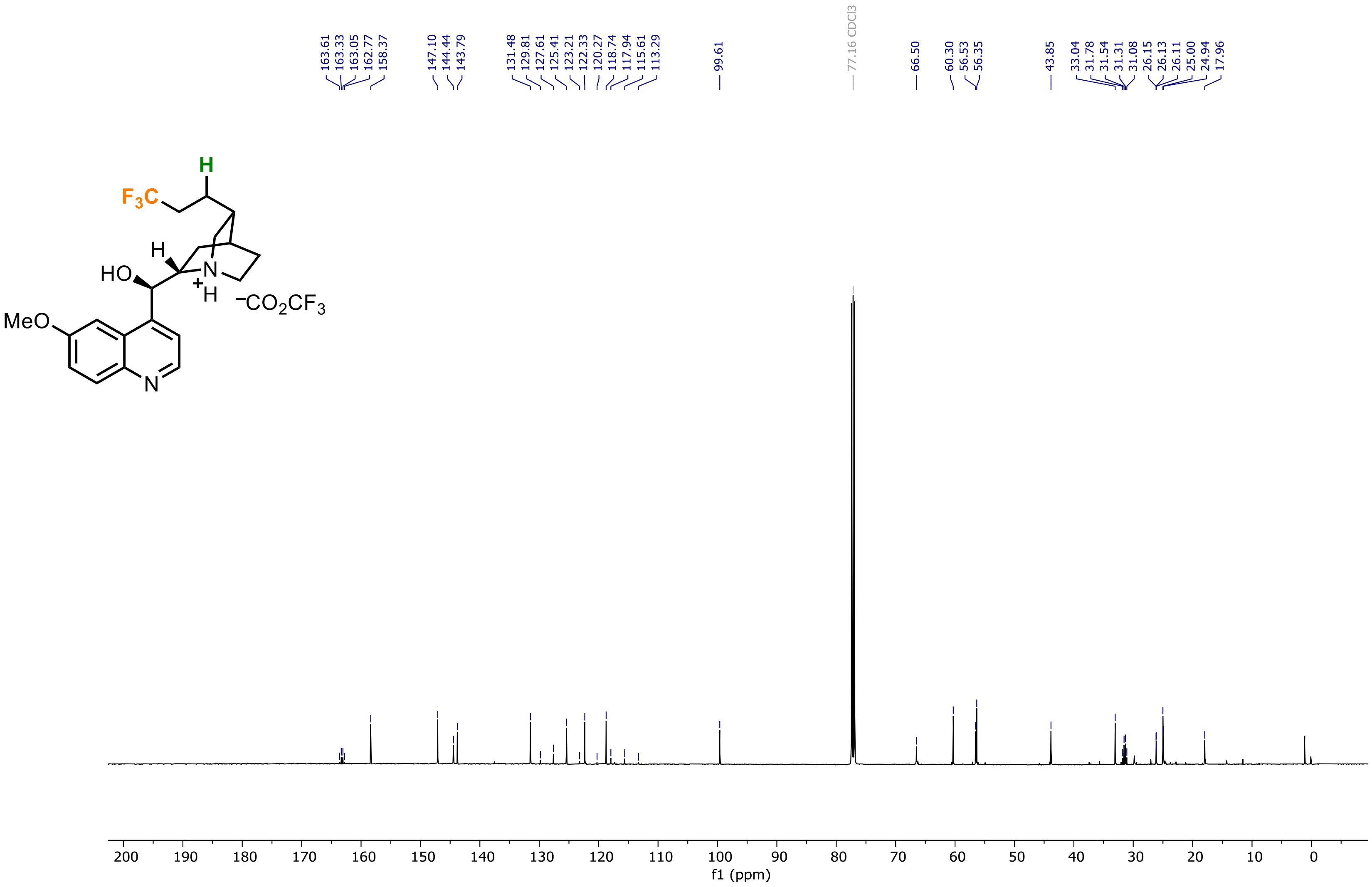

^{19}F NMR of quinine hydrotrifluoromethylated derivative (19)

$CDCl_3$

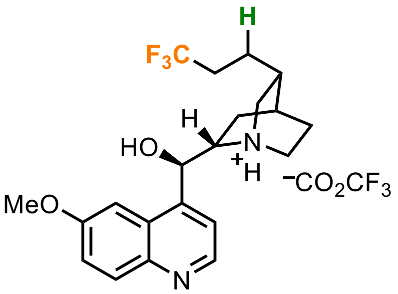

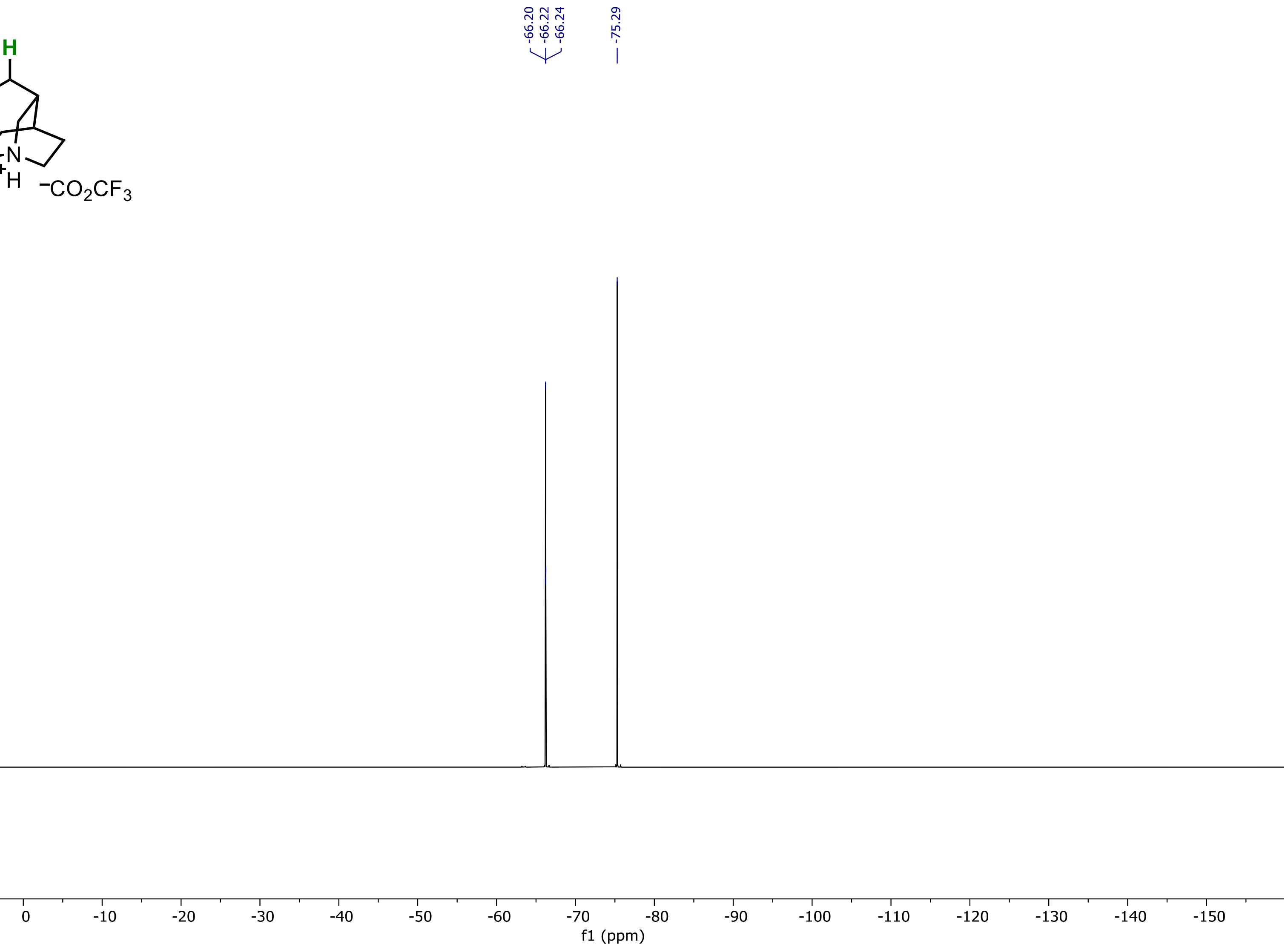

^{1}H NMR of vinclozolin hydrotrifluoromethylated derivative (20)

$CDCl_3$

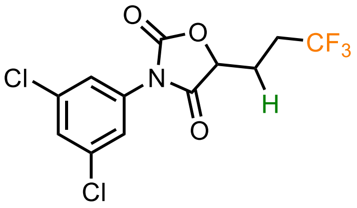

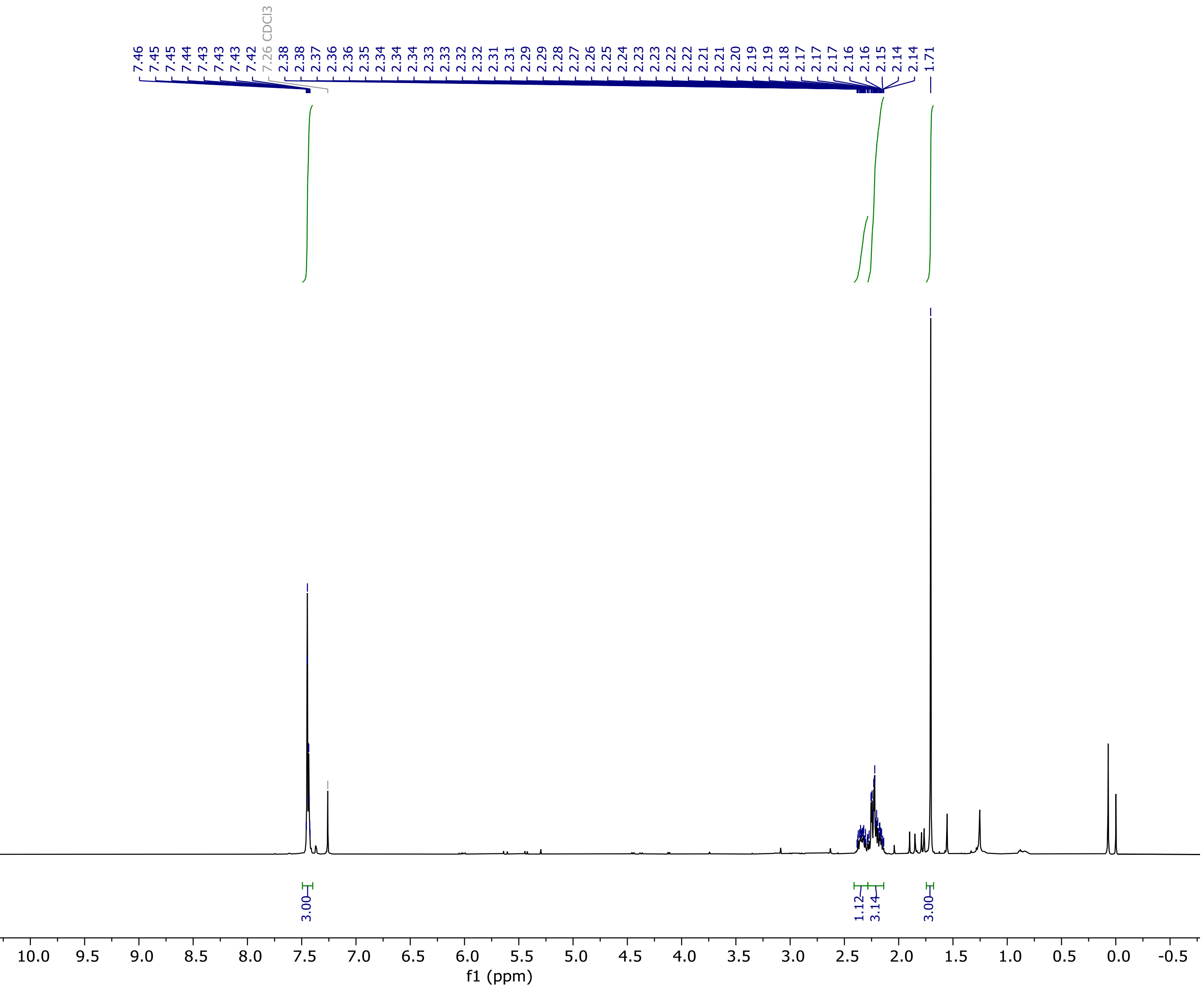

^{13}C NMR of vinclozolin hydrotrifluoromethylated derivative (20)

$CDCl_3$

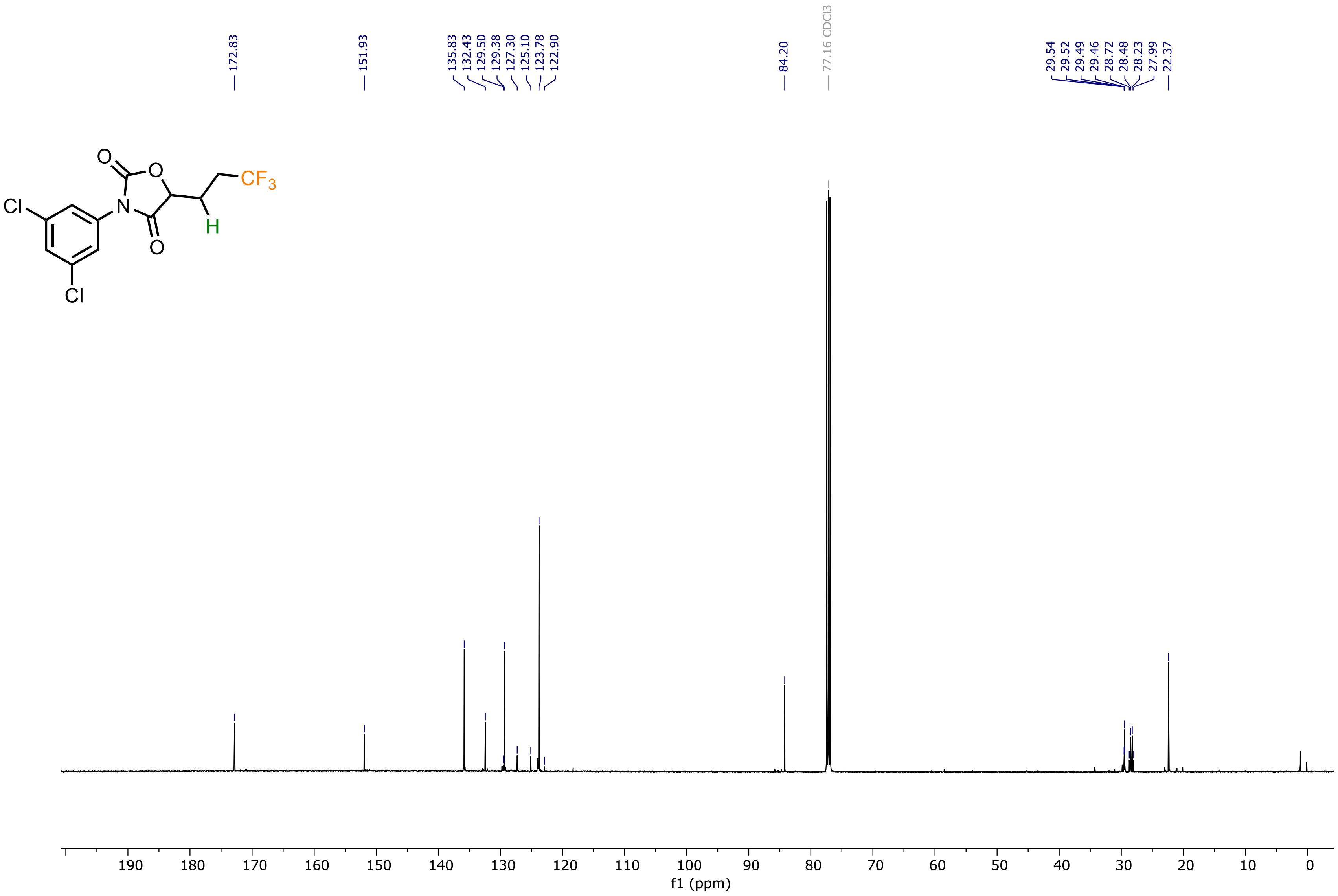

^{19}F NMR of vinclozolin hydrotrifluoromethylated derivative (20)

$CDCl_3$

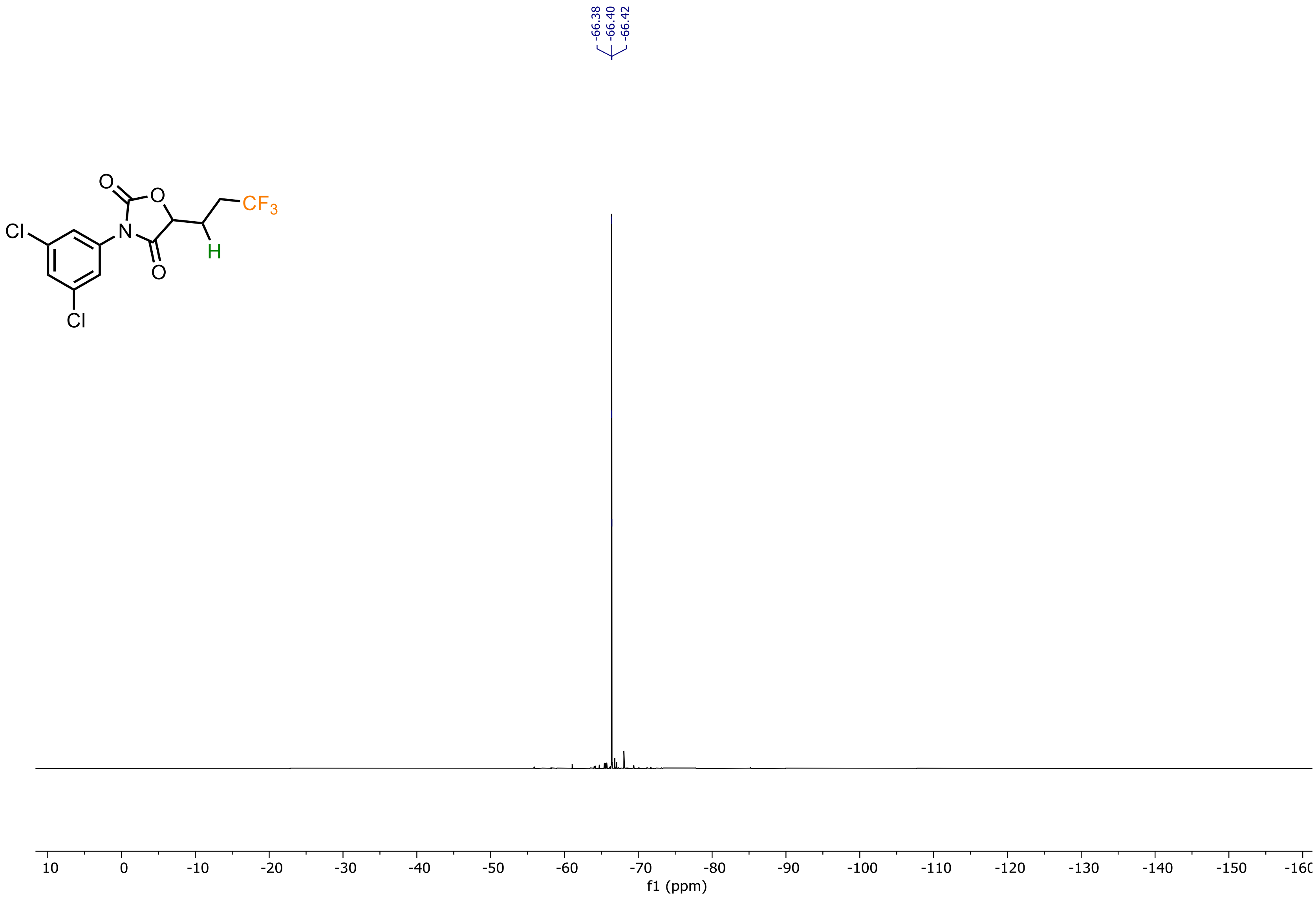

^{1}H NMR of theobromine hydrotrifluoromethylated derivative (21)

$CDCl_3$

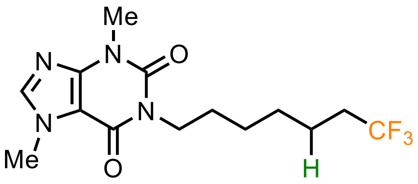

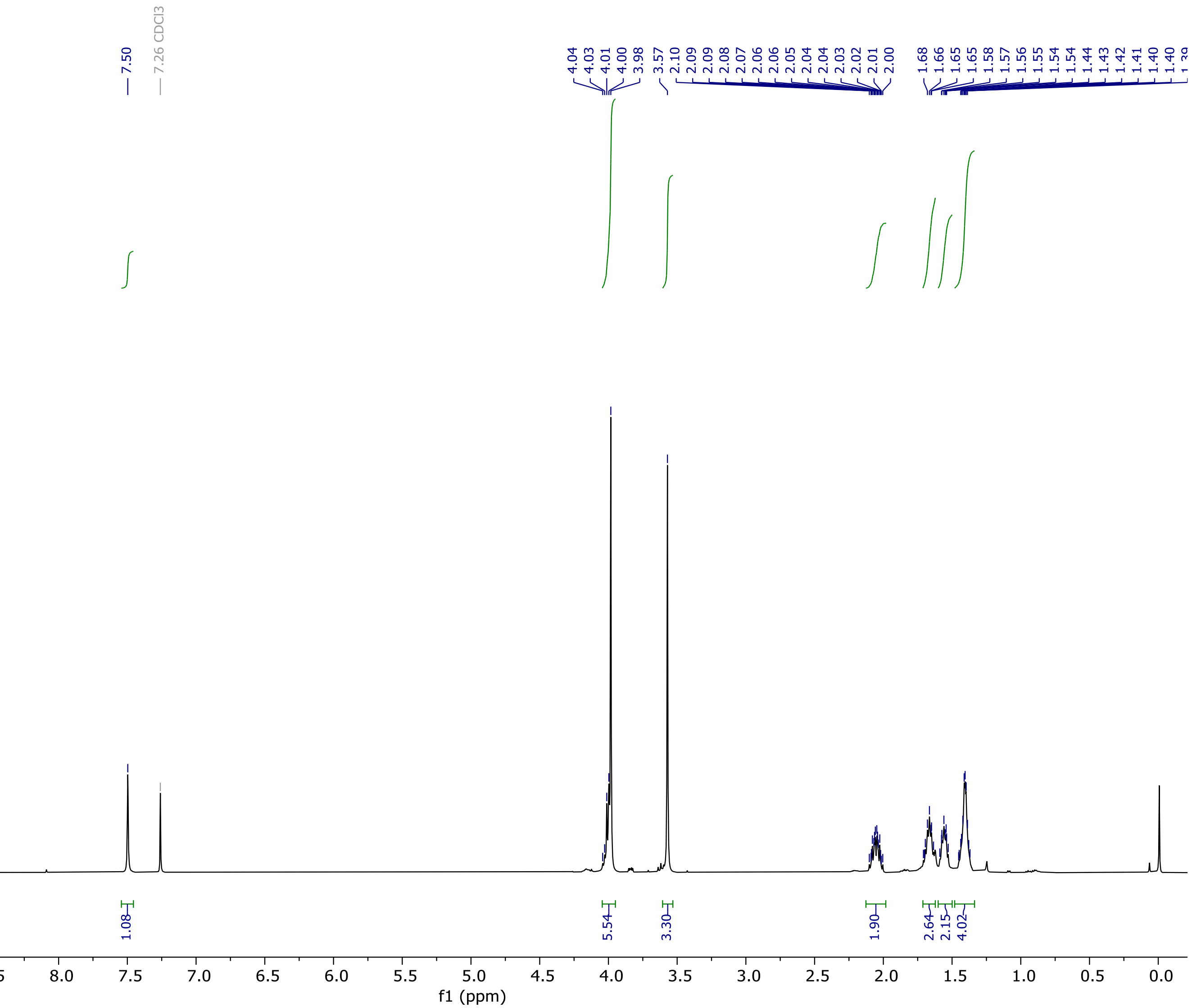

^{13}C NMR of theobromine hydrotrifluoromethylated derivative (21)

CDCl3

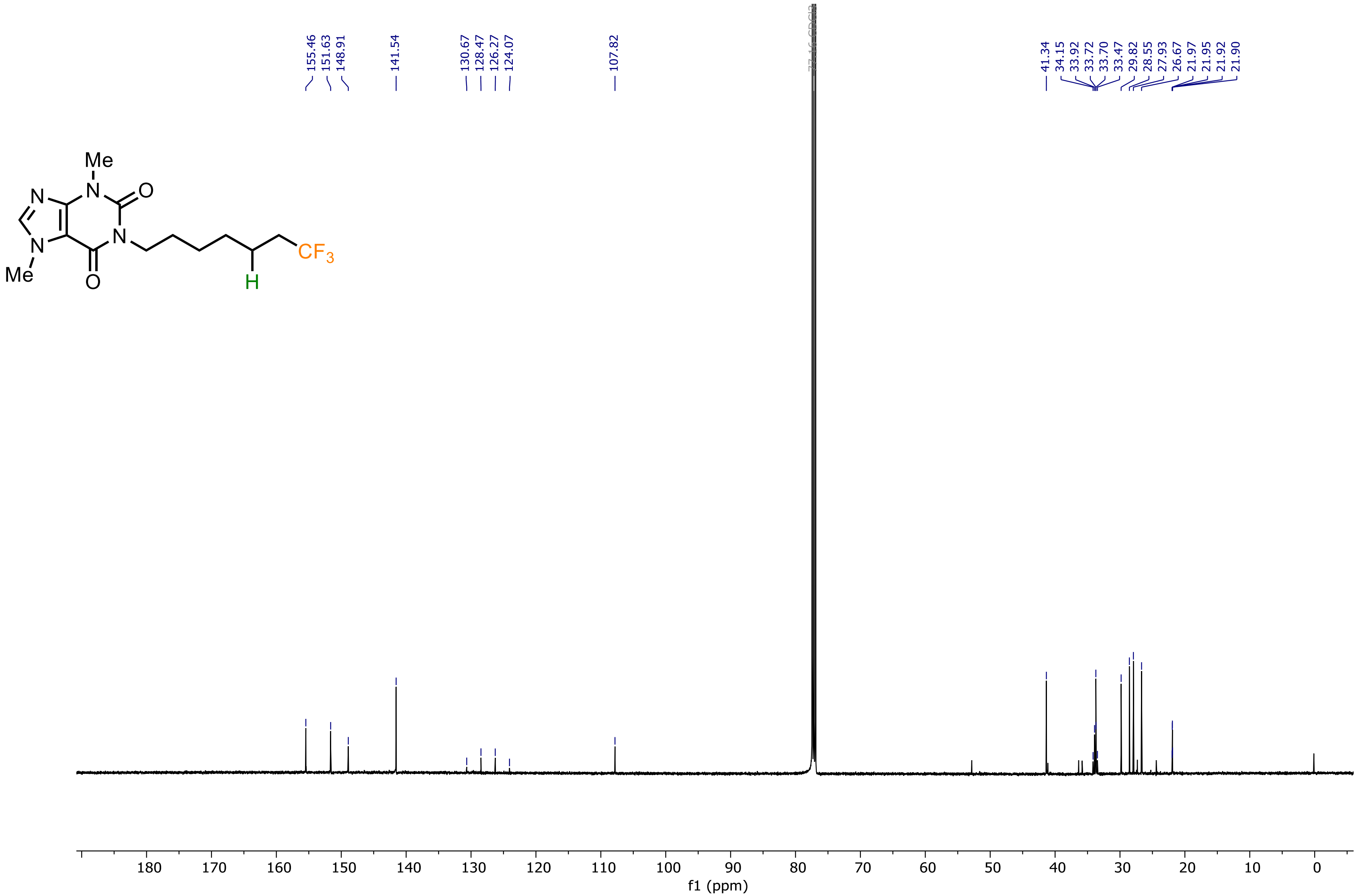

^{19}F NMR of theobromine hydrotrifluoromethylated derivative (21)

$CDCl_3$

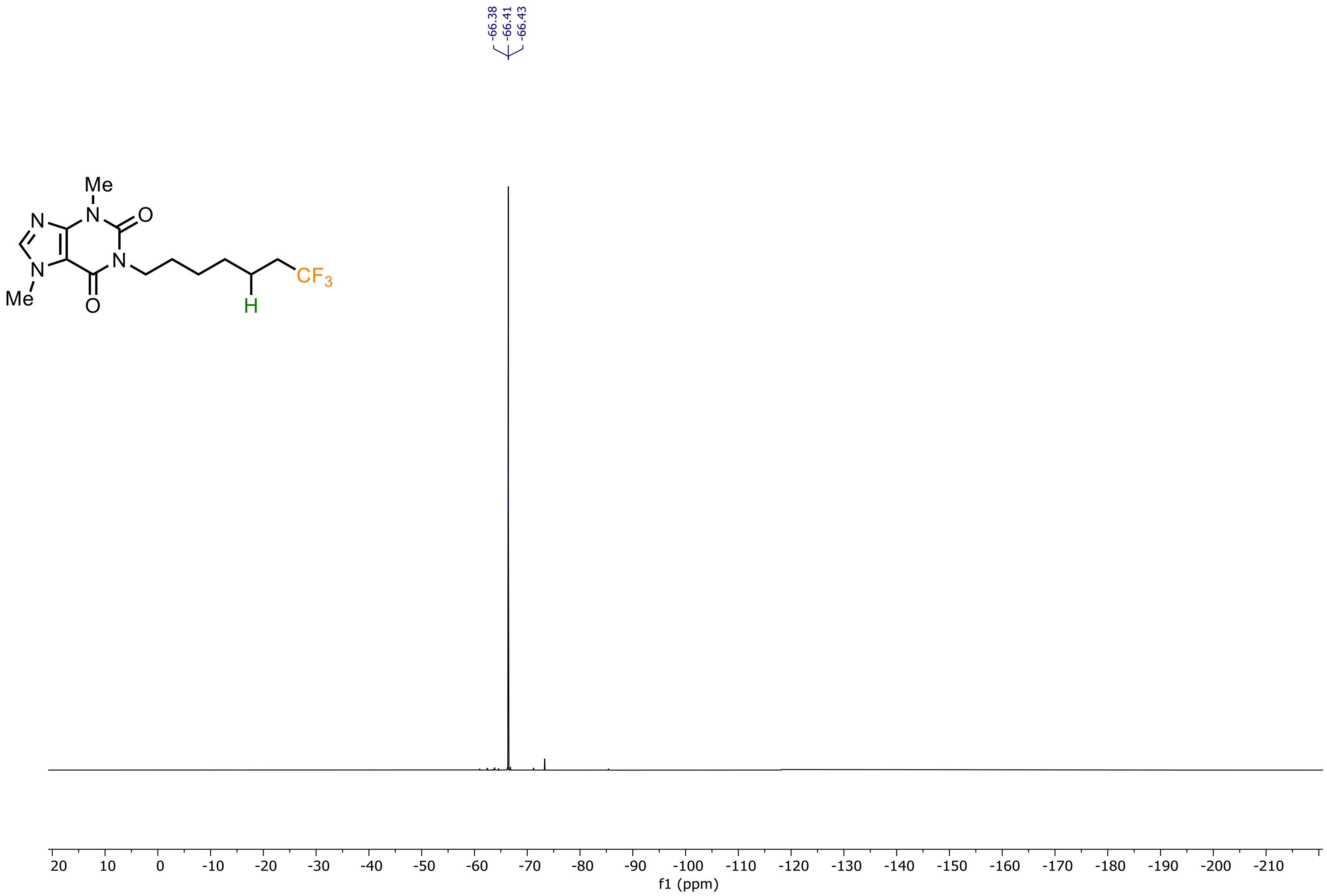

^{1}H NMR of indomethacin hydrotrifluoromethylated derivative (22)

$CDCl_3$

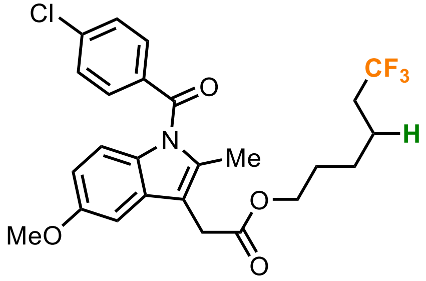

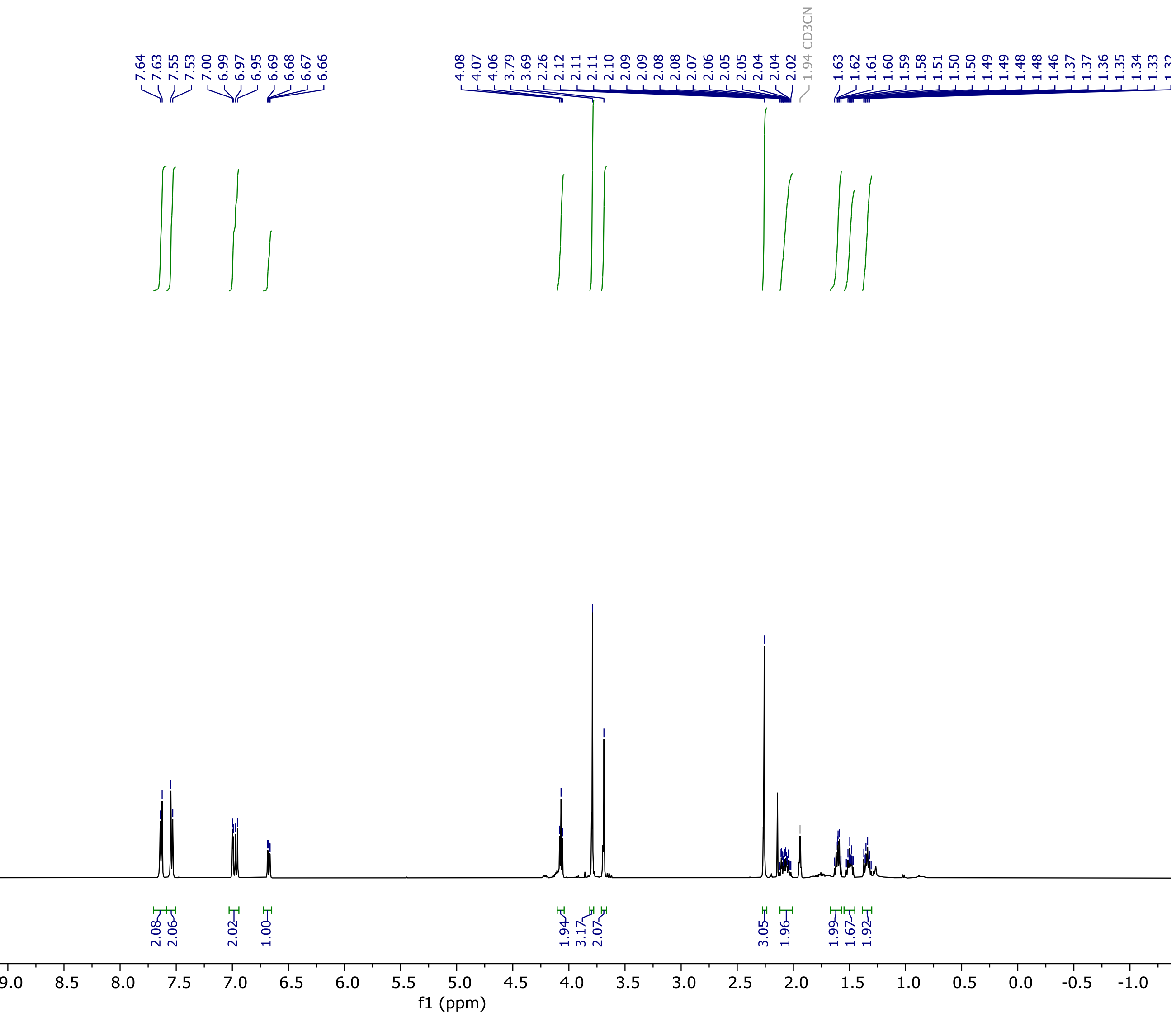

^{13}C NMR of indomethacin hydrotrifluoromethylated derivative (22)

CD_3CN

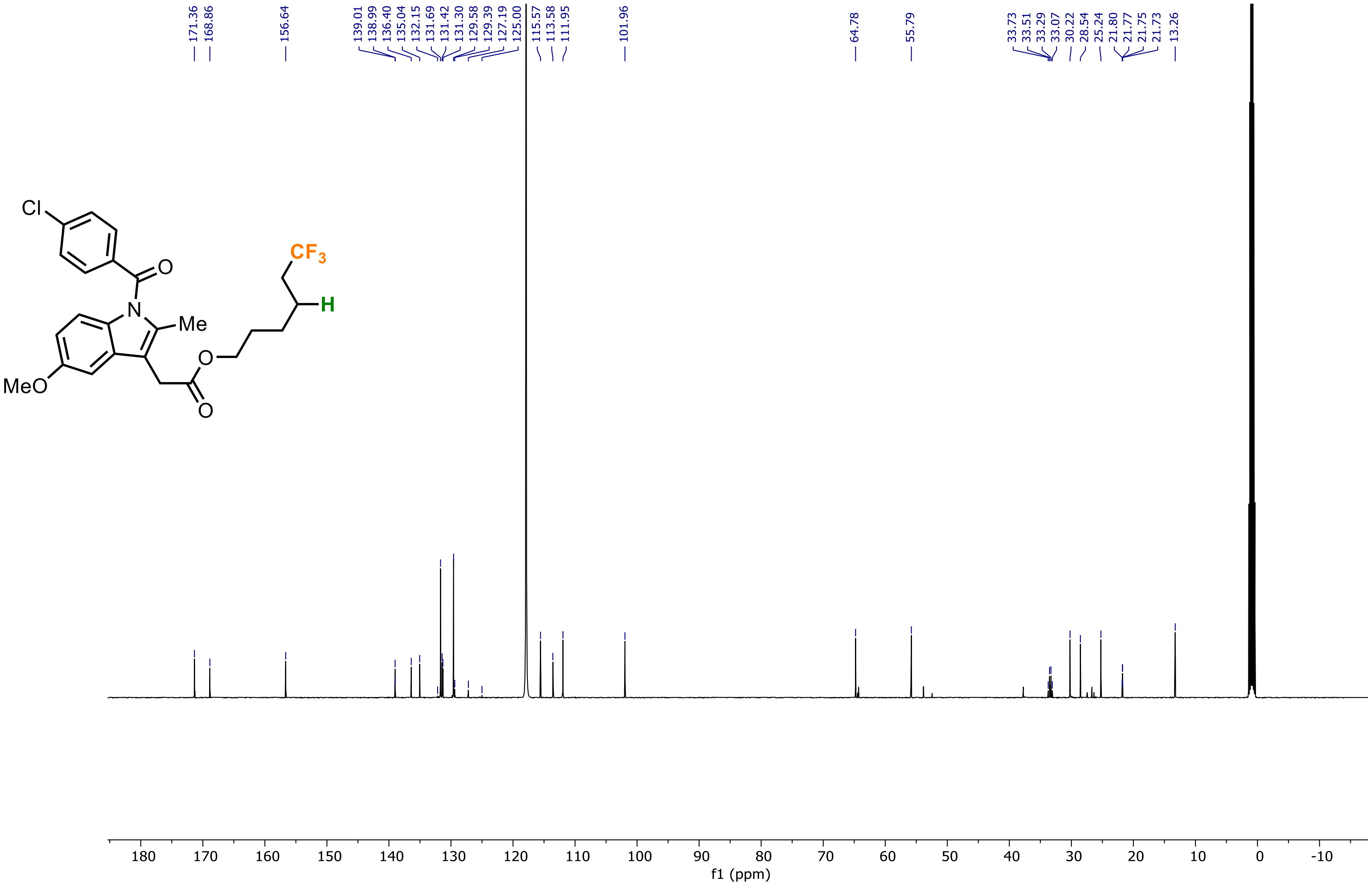

^{19}F NMR of indomethacin hydrotrifluoromethylated derivative (22)

CD_3CN

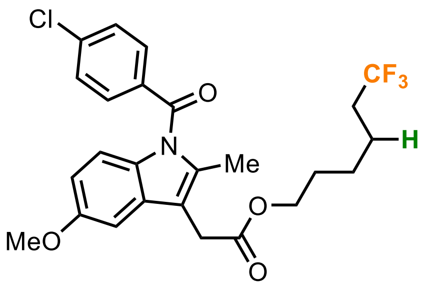

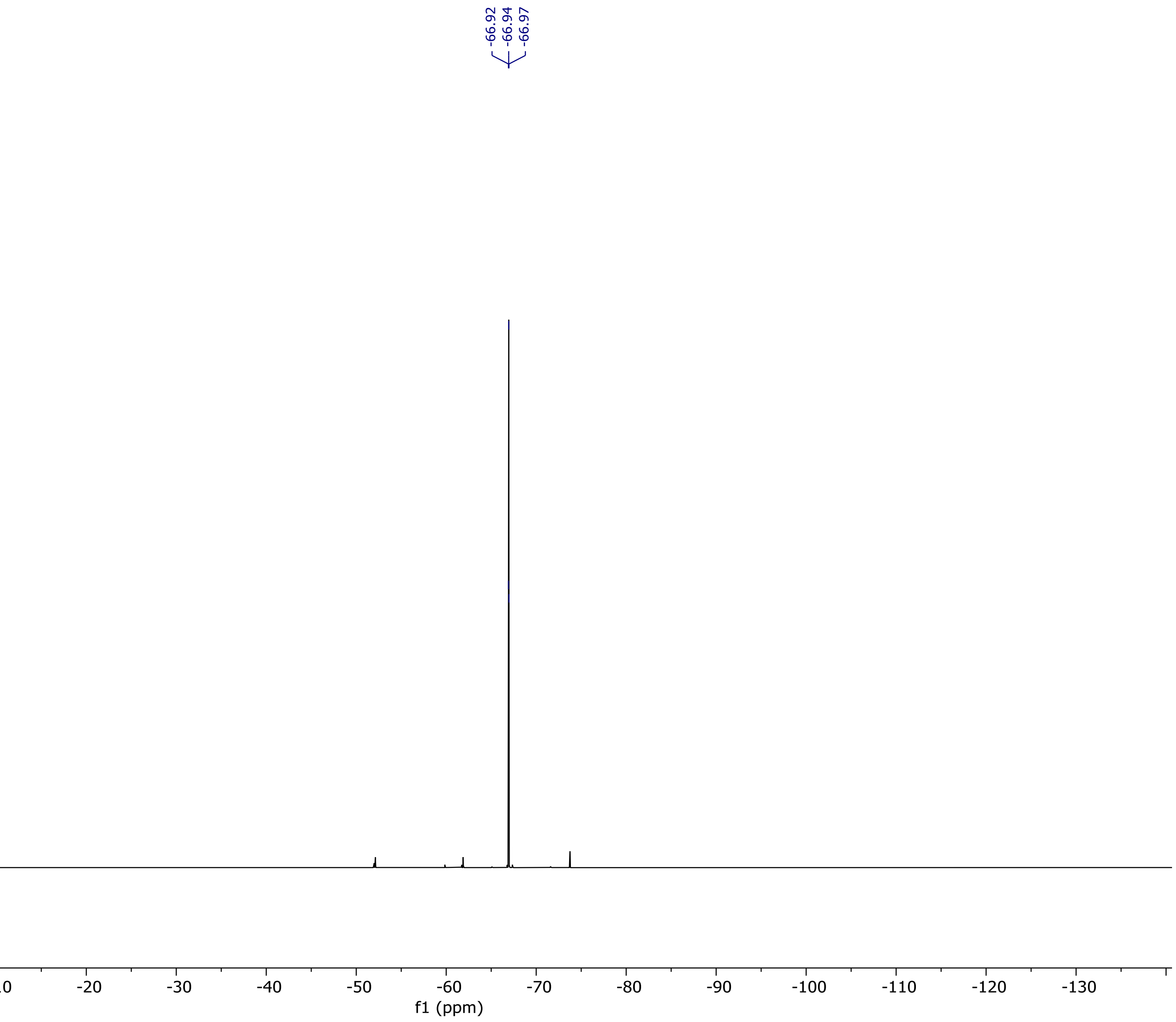

^{1}H NMR of probenecid hydrotrifluoromethylated derivative (23)

$CDCl_3$

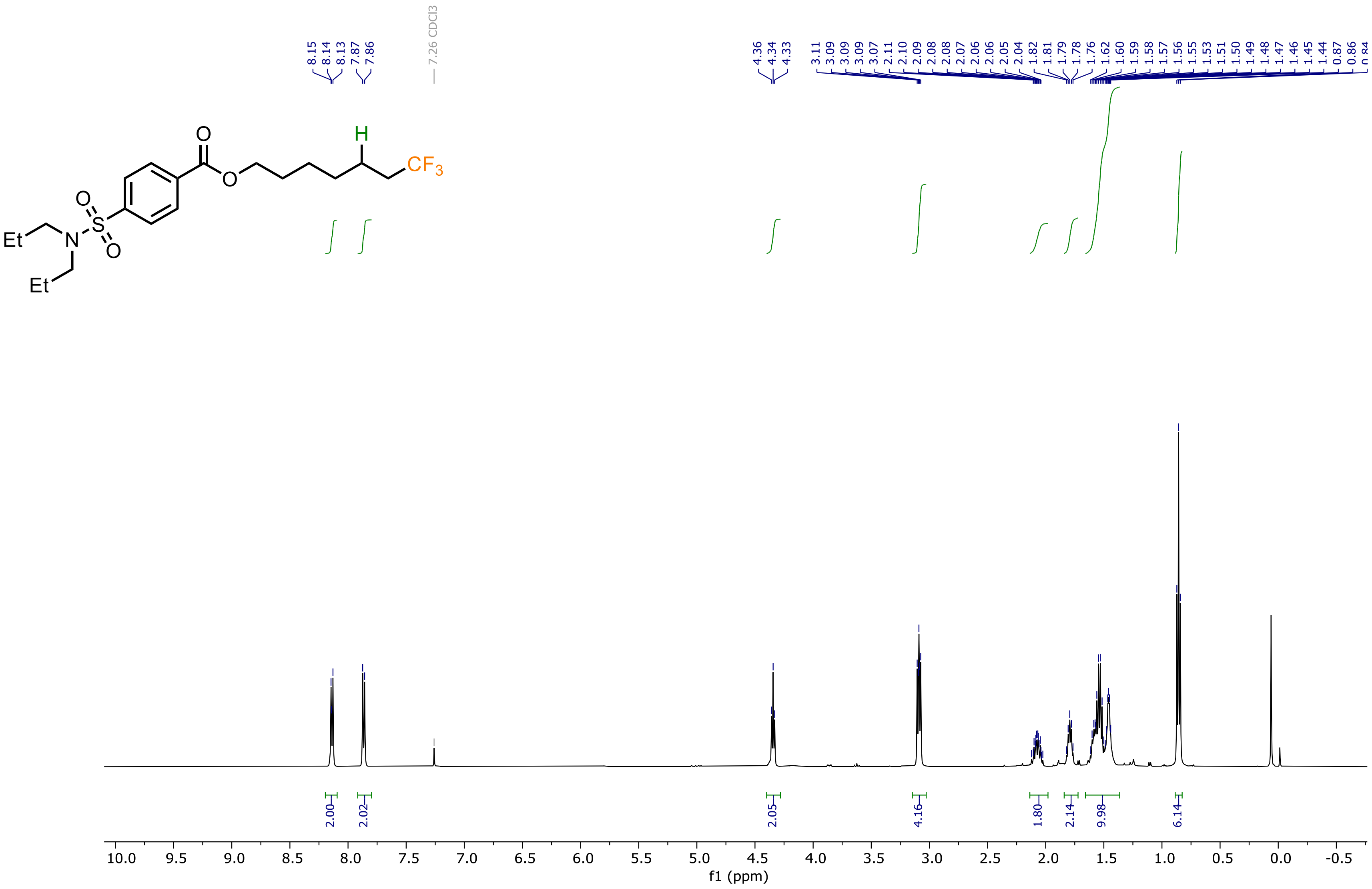

^{13}C NMR of probenecid hydrotrifluoromethylated derivative (23)

CDCl3

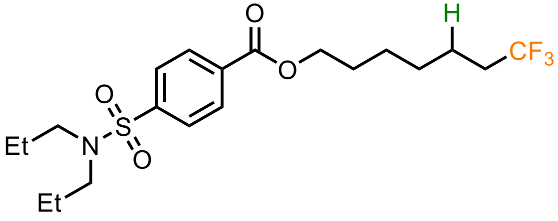

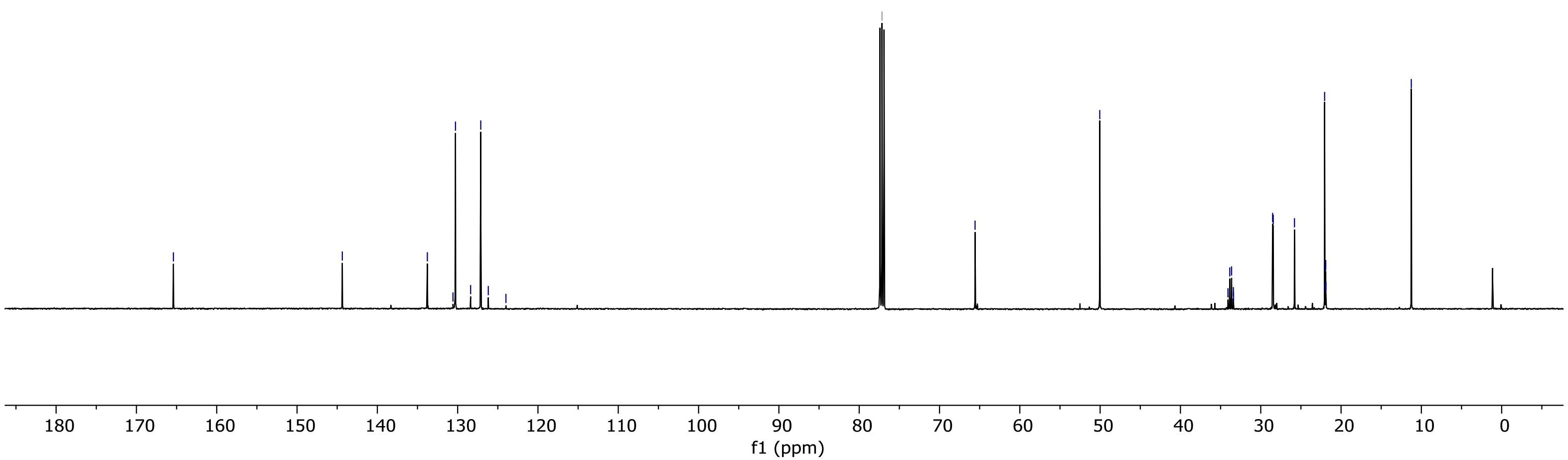

^{19}F NMR of probenecid hydrotrifluoromethylated derivative (23)

$CDCl_3$

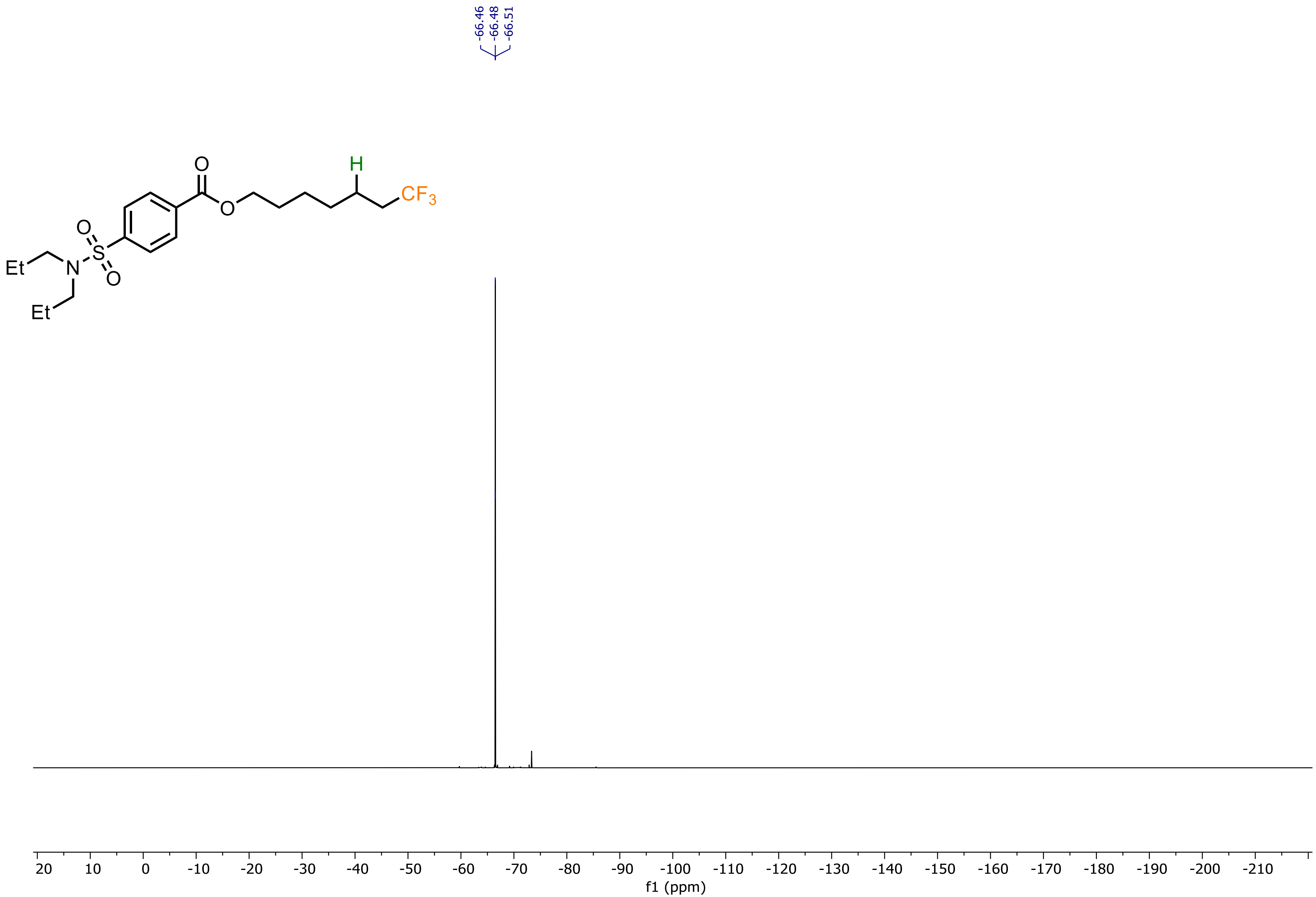

^{1}H NMR of diethyl 3-methyl-4-(2,2,2-trifluoroethyl)cyclopentane-1,1-dicarboxylate (24)

$CDCl_3$

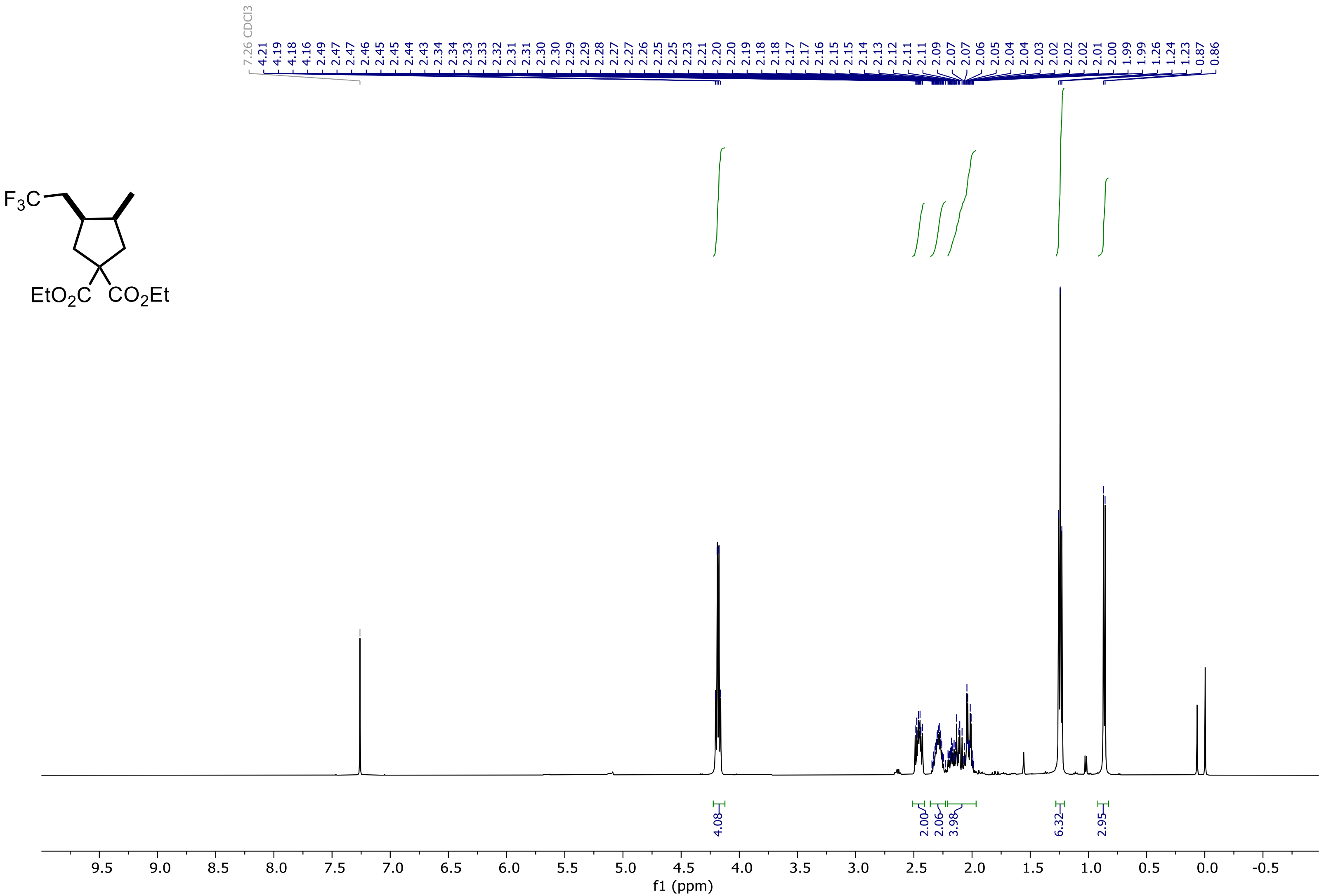

^{13}C NMR of diethyl 3-methyl-4-(2,2,2-trifluoroethyl)cyclopentane-1,1-dicarboxylate (24)

$CDCl_3$

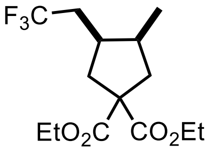

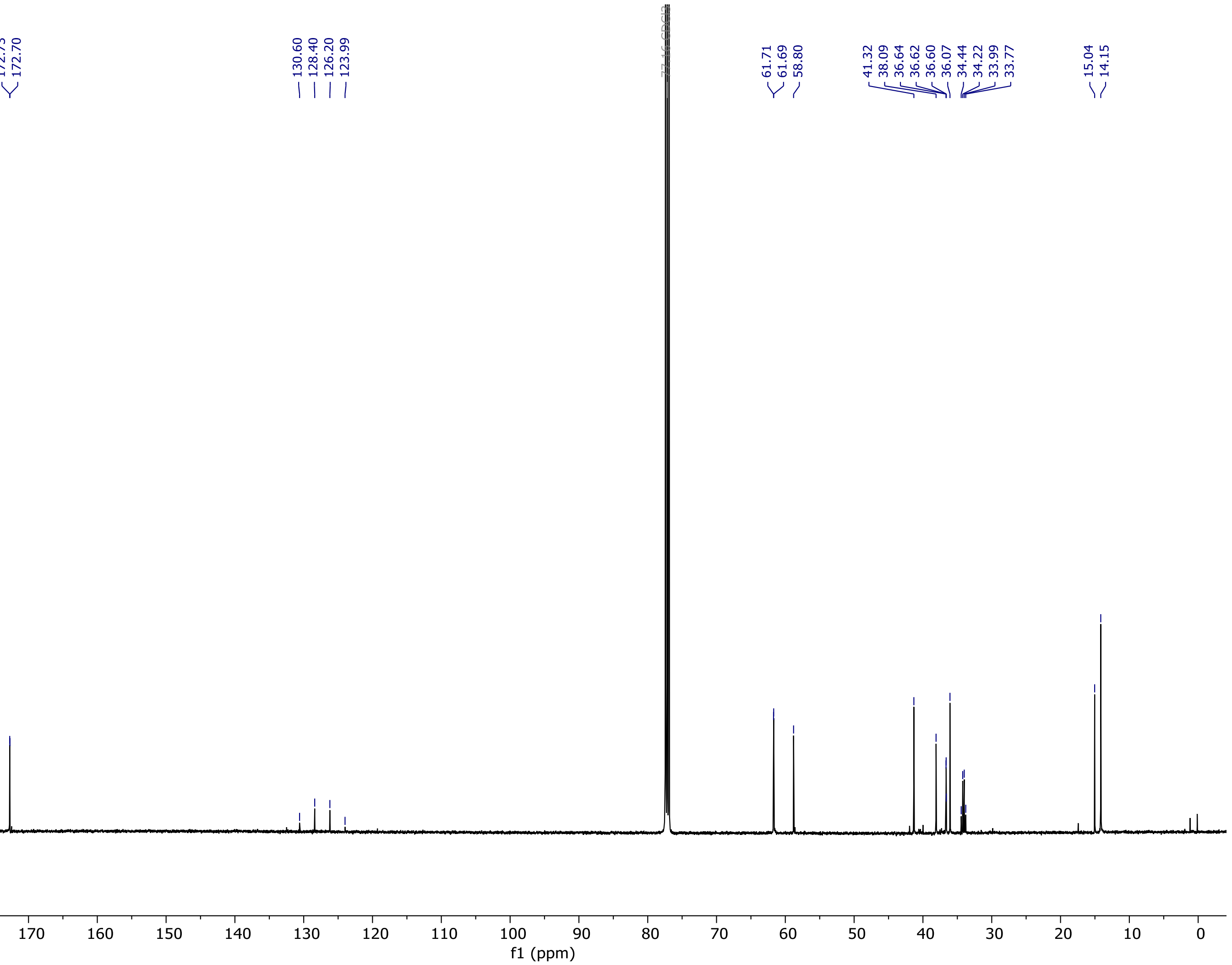

^{19}F NMR of diethyl 3-methyl-4-(2,2,2-trifluoroethyl)cyclopentane-1,1-dicarboxylate (24)

$CDCl_3$

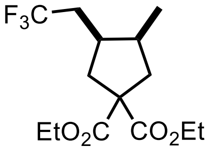

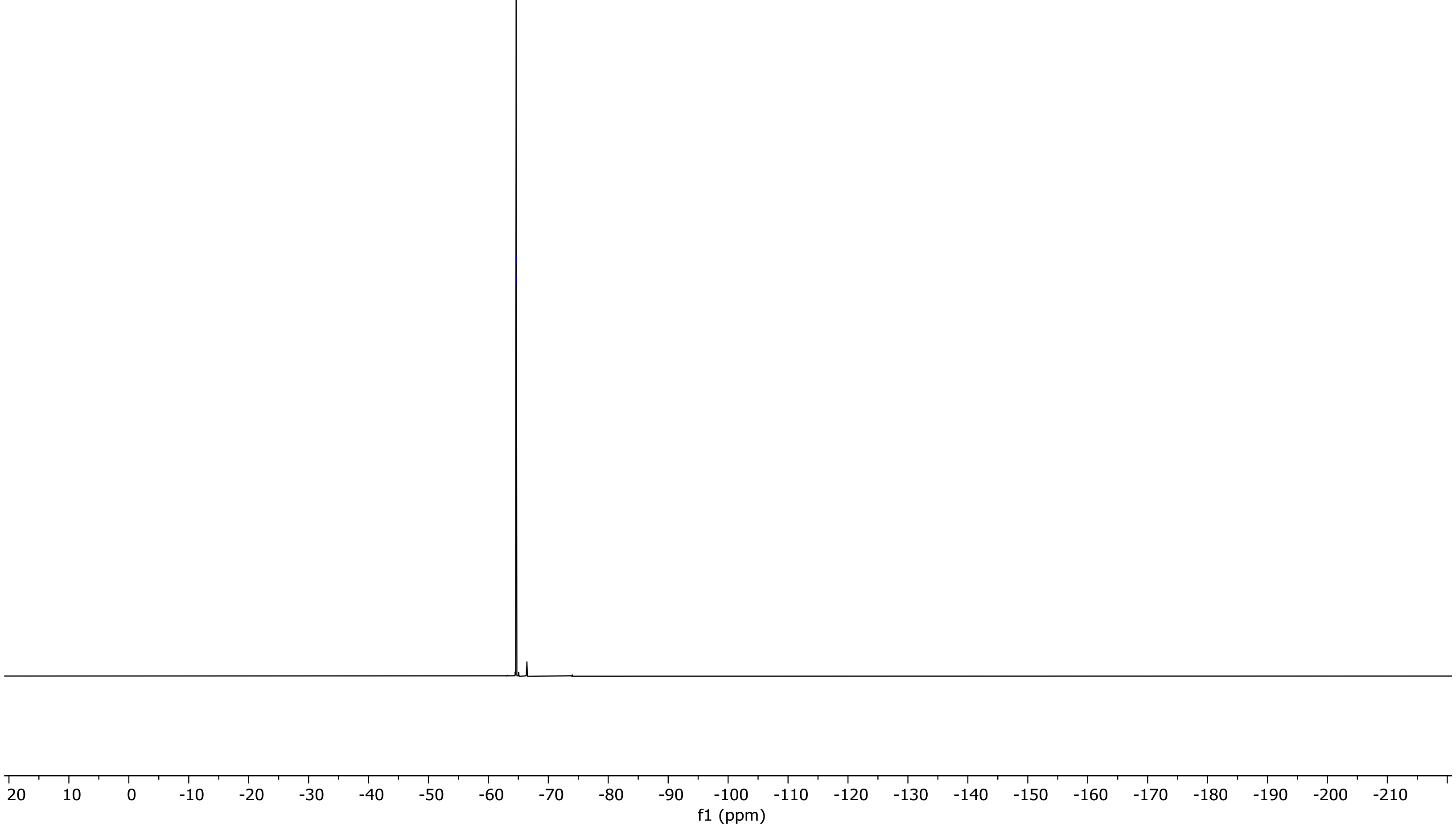

REFERENCES

1. Lindner, H.; Amberg, W. M.; Carreira, E. M. Iron-Mediated Photochemical Anti-Markovnikov Hydroazidation of Unactivated Olefins. *J. Am. Chem. Soc*. **2023,** *145*, 22347–22353.
2. Chen, J.; Li, J.; Plutschack, M. B.; Berger, F.; Ritter, T. Regio- and Stereoselective Thianthrenation of Olefins to Access Versatile Alkenyl Electrophiles. *Angew. Chem. Int. Ed.***2020***,59,* 5616 –5620.
3. Jia, H.; Haering, A. P.; Berger, F.; Zhang, L.; Ritter, T. Trifluoromethyl Thianthrenium Triflate: A Readily Available Trifluoromethylating Reagent with Formal CF3+, CF3•, and CF3– Reactivity *J. Am. Chem. Soc*. **2021**, *143*, 7623–7628.
4. Paeth, M.; Tyndall, S. B.; Chen, L-Y.; Hong, J.-C.; Carson, W. P.; Liu, X.; Sun, X.; Liu, J.; Yang, K.; Hale, E. M.; Tierney, D. L.; Liu, B.; Cao, Z.; Cheng, M.-J.; Goddard III, W. A.; Liu, W. Csp^3–Csp^3 Bond-Forming Reductive Elimination from Well-Defined Copper(III) Complexes Regio- and Stereoselective. *J. Am. Chem. Soc.* **2019**, *141*, 3153–3159.
5. Lu. C.; Qiu, Z.; Xuan, M.; Huang, Y.; Lou, Y.; Zhu, Y.; Shen, H.; Lin, B-L. Direct N-Alkylation/Fluoroalkylation of Amines Using Carboxylic Acids via Transition-Metal-Free Catalysis. *Adv. Synth.Catal*. **2020**, *362*, 4151–4158.
6. Rodrigo, S.; Hazra, A.; Mahajan, J. P.; Nguyen, H. M.; Luo, L. Overcoming the Potential Window-Limited Functional Group Compatibility by Alternating Current Electrolysis. *J. Am. Chem. Soc.* **2023,** *145,* 21851–21859.
7. Roth, H. G.; Romero, N. A.; Nicewicz, D. A. Experimental and calculated electrochemical potentials of common organic molecules for applications to single-electron redox chemistry. *Synlett.* **2016,** *27*, 714–723.
